# A divergent synthetic approach to tricyclic Furo[3,2-*e*]indolizines *via* base-mediated tandem annulations of diethyl malonate with aromatic bromomethyls

**DOI:** 10.1039/d6ra00540c

**Published:** 2026-02-19

**Authors:** Sandya Tambi Dorai, Sandeep Chandrashekharappa

**Affiliations:** a Department of Medicinal Chemistry, National Institute of Pharmaceutical Education and Research-Raebareli, Transit Campus Bijnor-Sisendi Road, Sarojini Nagar, Near CRPF Base Camp Lucknow UP-226002 India c.sandeep@niperraebareli.edu.in c.sandeep@niperrbl.ac.in +91-522-2975587 +91-522-2497903

## Abstract

A base-promoted, transition-metal-free one-pot method for the divergent synthesis of tricyclic furo[3,2-*e*]indolizines is described. The reaction of 1-(2-oxo-2-phenylethyl)-1*H*-pyrrole-2-carbaldehyde, diethyl malonate, and aromatic bromomethyl derivatives proceeds through a tandem Knoevenagel–aldol annulation, *O*-alkylation, and intramolecular cyclization to afford the corresponding furoindolizine products in good to excellent yields. The protocol employs readily accessible starting materials, operates under simple and transition-metal-free conditions, and enables the rapid construction of structurally diverse furo[3,2-*e*]indolizine frameworks. These features highlight the synthetic efficiency and practical applicability of the approach, making it a valuable platform for heterocyclic synthesis and potential applications in medicinal chemistry.

## Abstract 2

A base-promoted, transition-metal-free one-pot method for the divergent synthesis of tricyclic furo[3,2-e]indolizines is described.

## Graphical abstract

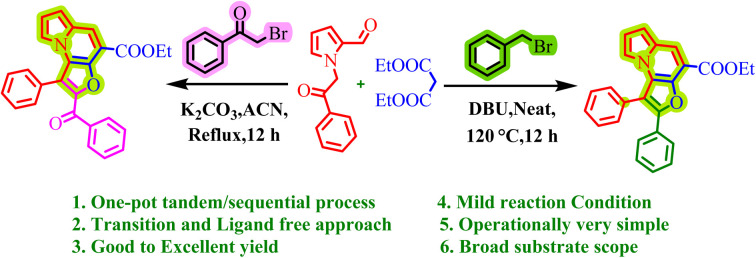

## Introduction

**Fig. 1:**
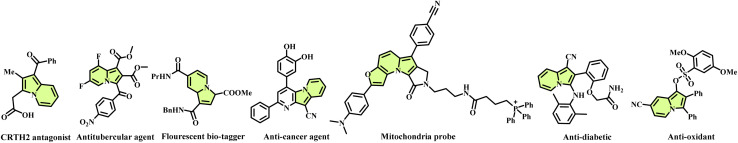
Biologically active indolizine and furoindolizine scaffold.

The indolizine scaffold is recognized as a privileged structural motif and it serves as a bioisostere of indole, a well-established pharmacophore which is found in natural products, pharmaceuticals, and clinically approved drugs.^1,2^ Owing to its structural resemblance to indoles, indolizine has emerged as a promising framework in medicinal chemistry, offering valuable alternatives for drug discovery and synthetic applications.^3–7^ Indolizine derivatives have attracted significant attention due to their diverse therapeutic potential against cancer, inflammation conditions,^8,9^ tuberculosis,^10,11^ and other infectious diseases. Additionally, these compounds exhibit diverse biological and pharmacological properties such as antimicrobial,^12^ antioxidant,^13^ antifungal and larvicidal activities,^14^ and show use as NmeNANAS inhibitors,^15^ fluorescent biotaggers and probes,^16^ as represented in Fig. 1. Notably, the indolizine scaffold displays intrinsic fluorescence properties, commonly referred to as Seoul-Fluor.^17^ Structural modification of substituents and their positions enables fine-tuning of photophysical properties, including emission wavelength and quantum yield. Such variations in structure can influence π-conjugation and intramolecular charge transfer, leading to environment-sensitive fluorescence across the visible spectrum. Its modularity supports bioorthogonal labelling applications, with indolizine-based probes demonstrating strong fluorescence turn-on behaviour in live-cell imaging.^16–18^

**Scheme 1:**
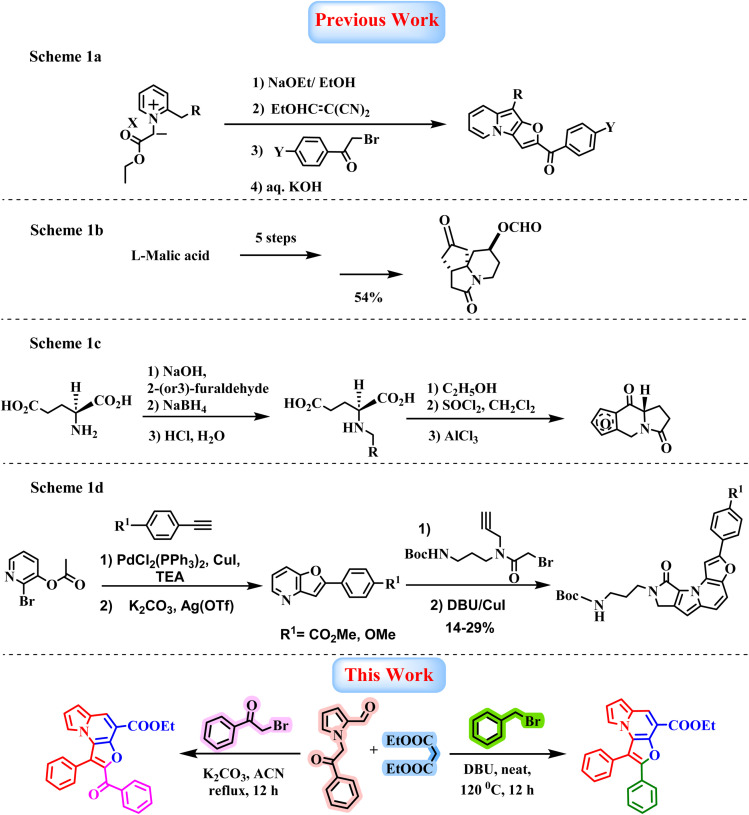
Reactions associated with previous studies (1a–d) and present work.

Indolizine-fused heterocycles have recently garnered significant attention across diverse research areas, particularly those incorporating a furan moiety due to its inherent fluorescence properties.^16–18^ The presence of multiple reactive sites within these frameworks renders them highly versatile, enabling extensive structural diversification. As a result, substantial efforts have been directed towards developing efficient synthetic methodologies for their construction. Among these, a notable strategy involves the Michael addition of various vinylating agents to 2(3*H*)-indolizinones, generated *in situ* from the corresponding 1-(ethoxycarbonylmethyl)-2-picolinium salts, followed by an alkaline treatment (Scheme 1a).^19^ While this strategy provides access to indolizine-based architecture, its applicability is limited by the narrow substrate scope of suitable vinylating agents. In parallel, several chiral furoindolizine derivatives have been reported. For instance, a multistep asymmetric synthesis of the furoindolizine framework starting from l-malic acid relies on a key *N*-acyliminium ion cyclization step (Scheme 1b).^20^ Similarly, condensation of (*S*)-glutamic acid with 2-(or 3)-furaldehyde to form Schiff base, followed by sodium borohydride reduction, affords (*S*)-*n*-(furylmethyl)-glutamic acid, which undergoes cyclization in ethanol to yield oxoproline. Subsequently, conversion to the corresponding acid chlorides using thionyl chloride and Friedel–Crafts cyclization using aluminium trichloride furnishes the desired furoindolizine product (Scheme 1c).^21^ Additionally, Lee and co-workers reported a metal-catalyzed coupling strategy for constructing the furan scaffold, which, upon further reaction with *tert-*butyl (3-(2-bromo-*N*-(prop-2-yn-1-yl)acetamido)propyl)carbamate, delivers the furo[3,2-*e*]indolizine core (Scheme 1d).^17^ Although several approaches for the construction of furoindolizine scaffolds have been reported, many suffer from limitations such as multistep procedures, pre-functionalized substrates, transition-metal catalysts, harsh reaction conditions, limited substrate scope, and lower yields, which restrict their synthetic utility. A key challenge in this area is developing a simple, high-yielding, base catalyzed, transition-metal-free one-pot methodology^22–24^ that enables the divergent synthesis of tricyclic heterocyclic frameworks is highly desirable. Herein, we report a base-promoted tandem, Knoevenagel–Aldol annulation involving 1-(2-oxo-2-phenylethyl)-1*H*-pyrrole-2-carbaldehyde and diethyl malonate, followed by *O*-alkylation of bromomethyl substrates and subsequent intramolecular cyclization. This sequence efficiently furnishes structurally diverse furo[3,2-*e*]indolizine derivatives under mild conditions, providing a versatile and divergent synthetic methodology (Scheme 1).

## Result and discussion

Initially, to identify the optimal reaction conditions, 1-(2-oxo-2-phenylethyl)-1*H*-pyrrole-2-carbaldehyde (1a), diethyl malonate (2), and 1-bromo-4-(bromomethyl)benzene (3a) were selected as model substrates (Table 1). Preliminary attempts employing the weak organic base triethylamine under neat conditions (110 °C) failed to yield the desired product (Table 1, Entry 1). Substitution with DIPEA likewise did not promote the reaction (Table 1, Entry 2 and Table S1, Entry 4). To enhance reactivity, the base strength was increased by using piperidine. However, conducting the reaction neat at room temperature for 24 h did not yield the target compound; instead, ethyl 5-benzoyl-6-hydroxyindolizine-7-carboxylate^25^ was obtained (Table S1, Entry 5). Elevating the temperature to 100 °C did not enhance product formation, and most of the starting materials, particularly the benzyl bromide, remained unreacted, with only trace amounts of compound 4.1 detected. Similarly, refluxing the reaction mixture in ethanol proved ineffective (Table S1, Entries 6 and 7). These observations suggested that moderate base strength was insufficient to drive the desired transformation.

**Table 1:** Optimization of reaction conditions for the synthesis of ethyl 2-(4-nitrophenyl)-1-phenylfuro[3,2-*e*]indolizine-4-carboxylate (4a)^a^

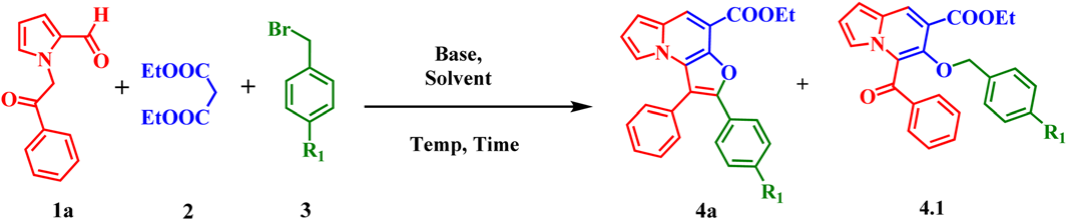							
Entry	Base (equiv.)	Solvent	Time (h)	Temp. (°C)	*R*_1_	Yield^b^ % (4.1)	Yield^b^ % (4a)
1	Et_3_N (3.0)	—	12	110	Br	—	—
2	DIPEA (2.0)	—	24	100	Br	—	—
3	Piperidine (Cat.)	—	24	100	Br	Trace	—
4	K_2_CO_3_ (1.0)	CH_3_CN	48	rt	Br	20	—
5	K_2_CO_3_ (1.5)	DMF	12	120	Br	72	—
6	Cs_2_CO_3_ (2.0)	CH_3_CN	12	85	Br	78	—
7	*t*-BuOK (2.0)	DMF	12	120	Br	67	—
8	NaH (2.5)	CH_3_CN	12	85	Br	61	
9	DBU (3.0)	CH_3_CN	24	85	Br	68	—
10	*t*-BuOK (4.0)	DMSO	12	120	NO_2_	52	Trace
11	DBU (2.0)	DMF	24	120	NO_2_	—	20
12	DBU (2.5)	DMF	24	120	NO_2_	—	58
**13**	**DBU (3.0)**	**—**	**12**	**120**	**NO**_**2**_	**—**	**80**
14	DBU (3.0)	CH_3_CN	24	85	NO_2_	—	72

a Reaction condition: 1a (0.9 mmol), 2 (1.8 mmol), 3a (0.9 mmol), DBU (2.8 mmol), 120 °C, 12 h.

b Isolated yields.

Subsequently, stronger inorganic bases were investigated. Use of potassium carbonate (K_2_CO_3_) in a polar aprotic solvent such as acetonitrile resulted in the formation of compound 4.1 in 20% yield after 48 h at room temperature, with no detectable formation of compound 4a. (Table 1, Entry 4). Increasing the base equivalents and screening solvents, including acetonitrile and dimethylformamide, led to improved yields of 4.1, with no observation of 4a (Table S1, Entries 9–11). Further screening of bases such as cesium carbonate, potassium *tert*-butoxide, sodium hydride, and DBU were carried out in various solvents. However, these conditions did not enhance the formation of compound 4a (Table 1, Entries 6–9 and Table S1, Entries 12–19). Reactions employing benzyl bromides bearing electron-donating substituents and other halogen groups consistently resulted in the formation of *O*-alkylated indolizine intermediate 4.1.

To promote cyclization, the effect of electron-withdrawing substituents on the benzyl bromide was examined. Benzyl bromides containing nitro and cyano groups were subjected to the reaction under different conditions. Notably, in the presence of potassium *tert*-butoxide and polar aprotic solvents such as DMF and DMSO, trace amount of the desired cyclized product 4a was detected (Table 1, Entry 10 and Table S1, Entries 20–22), indicating that electron-withdrawing groups promote the cyclization process.

To improve the yield of the cyclized product, stronger bases and reaction parameters were systematically evaluated. Optimal results were obtained using 3.0 equivalents of DBU under neat condition at 120 °C, affording compound 4a in 80% yield (Table 1, Entries 11–14). These results highlight the importance of base strength and electronic effects in facilitating cyclization, and the optimized reaction condition (Table 1, Entry 13) was subsequently taken for further studies.

**Scheme 2:**
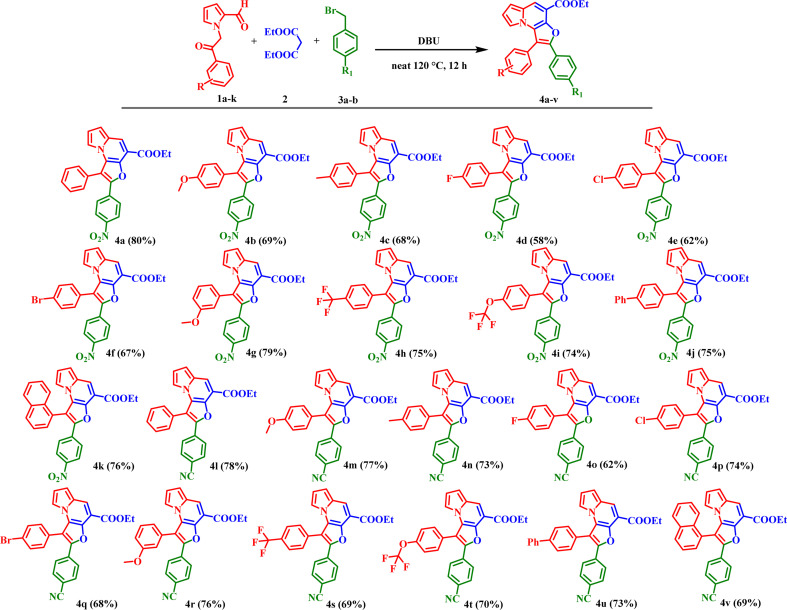
Substrate scope for 4-nitro- and 4-cyanophenyl furo[3,2-*e*]indolizine-4-carboxylates (4a–v); reaction condition: 1 (0.9 mmol), 2 (1.8 mmol), 3 (0.9 mmol), DBU (2.8 mmol), 120 °C, 12 h. Isolated yields.

As illustrated in Scheme 2, the substrate scope was explored with respect to the benzoyl group substitutions at the *meta-*and *para-*positions of 1-(2-oxo-2-phenylethyl)-1*H*-pyrrole-2-carbaldehyde (1a–k) in reaction with aromatic bromomethyls (3a–b), affording the corresponding furo[3,2-*e*]indolizines (4a–4v) in moderate to good yields. Pyrrole substrates containing electron-donating groups (EDGs) on the benzoyl moiety, such as OCH_3_, CH_3_, phenyl, and naphthyl substituents, provided the desired products (4b–c, 4j–n, 4u–v) in good yields ranging from 68–77%. Notably, electron-withdrawing groups (EWGs), including Br, Cl, and F, were also well tolerated under the optimized conditions, delivering the corresponding products (4d–f, 4o–q) in moderate yields (58–74%). These results demonstrate the broad functional-group compatibility of the protocol, although EDG-substituted substrates generally afforded slightly higher yields than EWG-substituted substrates.

**Scheme 3:**
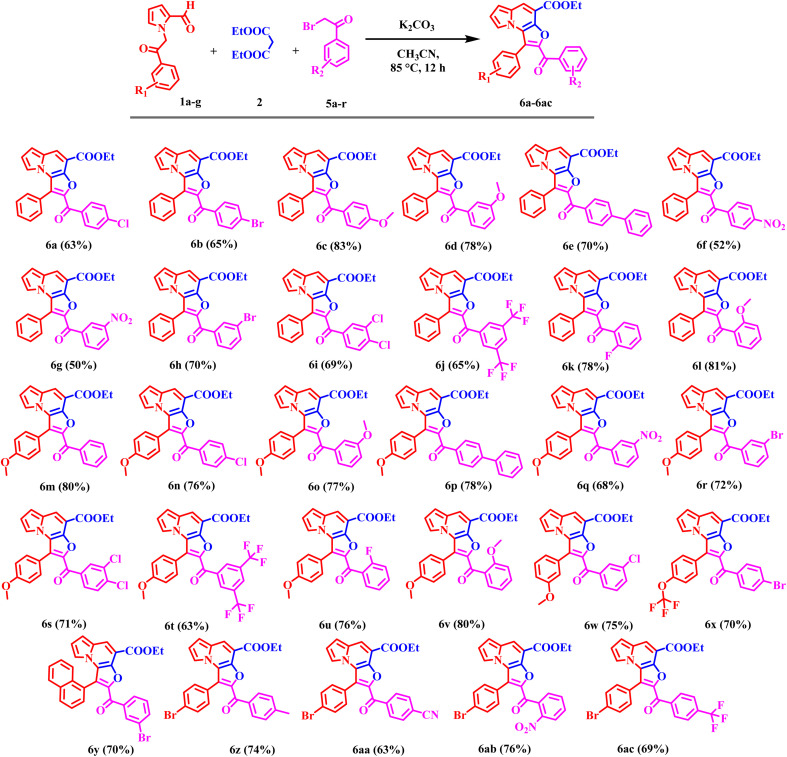
Substrate scope of ethyl 2-benzoyl-1-phenylfuro[3,2-*e*]indolizine-4-carboxylate derivatives (6a–ac); reaction condition: 1 (0.9 mmol), 2 (1.8 mmol), 5 (0.9 mmol), K_2_CO_3_ (1.4 mmol), 85 °C, 12 h. Isolated yields.

**Scheme 4:**
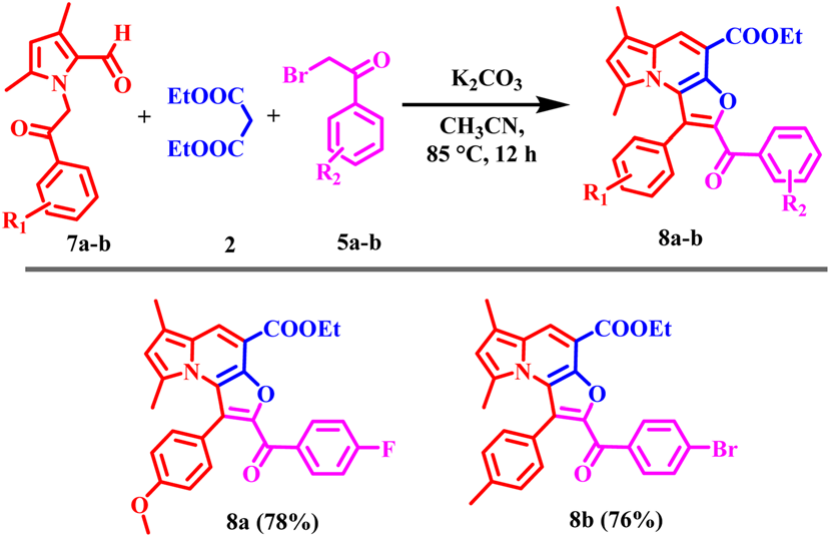
Substrate scope of 6,8-dimethyl furo[3,2-*e*]indolizine derivatives (8a–b); reaction condition: 7 (0.7 mmol), 2 (1.5 mmol), 5 (0.7 mmol), K_2_CO_3_ (1.1 mmol), 85 °C, 12 h, isolated yields.

Encouraged by these results, the scope of the tandem Knoevenagel–Aldol annulation was further extended to phenacyl bromide derivatives (5a–r) and pyrrole substituted intermediate (7a–b) as depicted in Schemes 3 and 4. Unlike benzyl bromide substrates, initial reactions using piperidine under neat or ethanol conditions were ineffective, yielding no or only trace amount of product (Table S2, Entries 1–3). This outcome is likely due to insufficient activation of the phenacyl bromide towards C–C bond formation under mild basic conditions. In contrast, the use of K_2_CO_3_ in acetonitrile significantly improved the reaction outcome, yielding 20% at room temperature and increasing to 80% at 85 °C (Table S2, Entries 4–6). Other bases, including Cs_2_CO_3_, NaH, potassium *tert*-butoxide, and DBU, yielded only moderate results, likely due to competing side reactions under strongly basic conditions (Table S2, Entries 7–11).

Overall, K_2_CO_3_ in acetonitrile was identified as the optimal condition for phenacyl bromide substrate. Under optimized conditions, phenacyl bromide derivatives bearing substituents at the *para*-, *meta*-, and *ortho*-positions of the phenyl ring yielded the desired products (6a–6ac) in good to excellent yields. *Para*-substituted electron-donating groups, such as CH_3_ and OCH_3_, phenyl provided compounds 6c, 6e, 6p, and 6z in high yield (70–83%). Corresponding *meta*-substituted derivatives 6d and 6o were obtained in 78% and 77% yields, respectively, while *ortho*-substituted analogues 6l and 6v furnished the products in similarly high yields (81% and 80%) respectively. In contrast, electron-withdrawing groups including F, Cl, Br, CF_3_, NO_2_, and CN at the *para*-position resulted in comparatively lower yields, ranging from 52–76%. *Meta*-substituted substrates afforded the corresponding products (6g, 6h, 6q, 6r, 6w, and 6y) in moderate yields (50–70%). While the *ortho*-substituted derivative 6u was obtained in 76% yield. Disubstituted phenacyl bromides were also compatible under the optimized conditions; 3,5-di-CF_3_ substituted substrate furnished products 6j and 6t in 65% and 63% yields, respectively, whereas 3,4-dichloro analogue provided products 6i and 6s in 69% and 71% yields. Overall, these results indicate that the electron-donating substituents, particularly at the para position, favor higher reaction efficiency. In contrast, the electron-withdrawing and disubstituted substrates moderately reduce yields, consistent with electronic modulation of the annulation process.

**Scheme 5:**
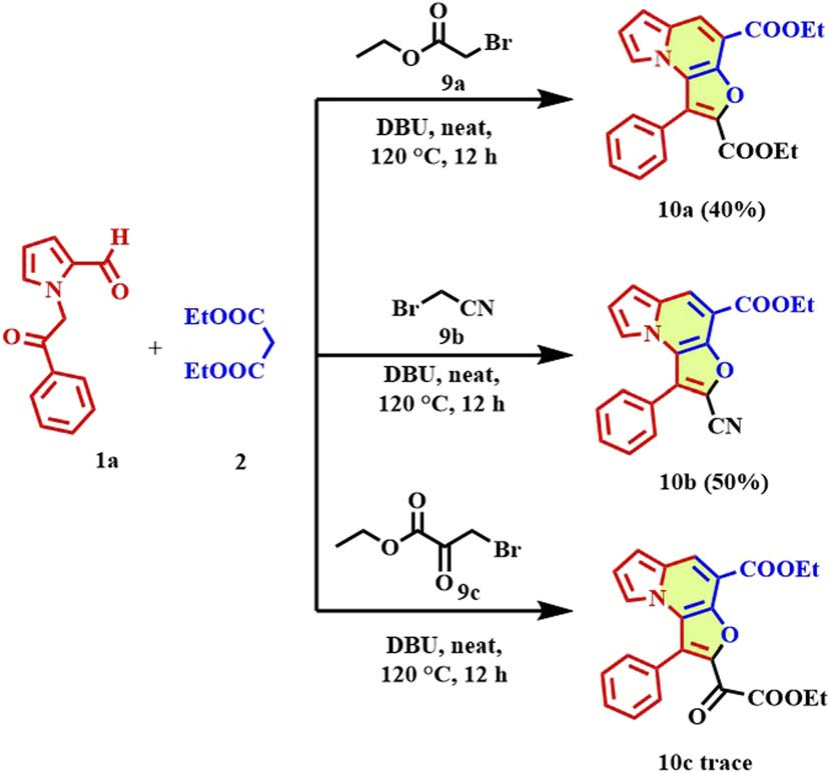
Synthesis of furo[3,2-*e*]indolizine analogues (10a–c) using aliphatic bromomethyls (9a–c); reaction condition: 1 (0.9 mmol), 2 (1.8 mmol), 9 (0.9 mmol), DBU (2.8 mmol), 120 °C, 12 h. Isolated yields.

To further assess the scope and applicability of the developed protocol, the reaction was extended to aliphatic bromomethyl derivatives, including ethyl bromoacetate, bromoacetonitrile, and ethyl 3-bromopyruvate. Under the optimized neat conditions in the presence of DBU (3.0 equiv) at 120 °C for 12 h, these substrates successfully underwent the tandem Knoevenagel-*O*-alkylation-cyclization sequence to afford the corresponding furo[3,2-*e*]indolizine derivatives (Scheme 5). The reaction with ethyl bromoacetate, bromoacetonitrile, and ethyl 3-bromopyruvate afforded products 10a–b in 40%, 50% respectively, and compound 10c was found in trace amount. The successful incorporation of ester, nitrile functionalities demonstrates the tolerance of the protocol towards diverse aliphatic electrophiles. Importantly, this expanded substrate scope highlights the versatility of the base-mediated, transition-metal-free one-pot strategy.

### Plausible mechanism

**Scheme 6:**
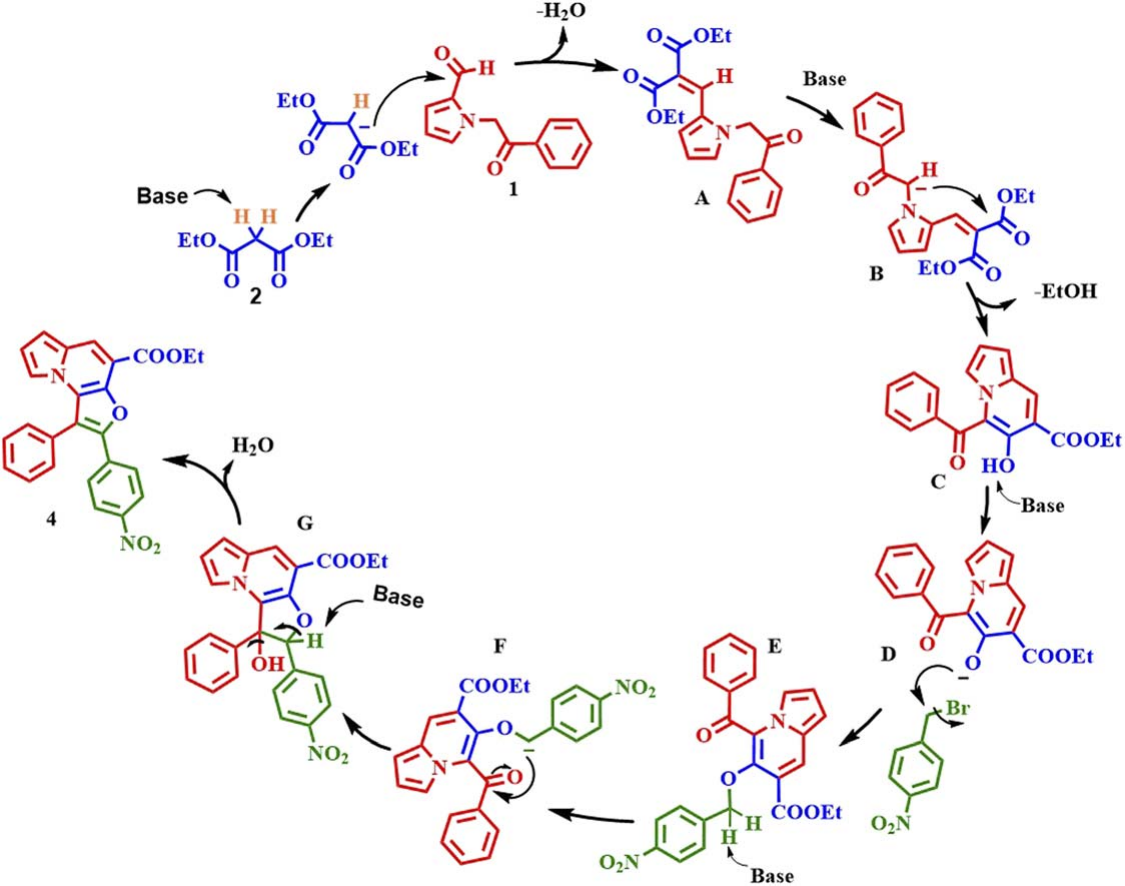
Plausible reaction pathway for the base-mediated formation of furo[3,2-*e*]indolizines from 1-(2-oxo-2-phenylethyl)-1*H*-pyrrole-2-carbaldehyde and 4-nitro benzyl bromide.

Based on the preliminary results and the previous literature,^25^ a plausible mechanism is proposed in Scheme 6. The reaction proceeds through a series of steps, beginning with a base-mediated Knoevenagel condensation reaction between compounds 1 and 2, to form intermediate (A). This intermediate then undergoes cyclization to produce ethyl 5-benzoyl-6-hydroxyindolizine-7-carboxylate (C). Next, the β-carbon, which bears a hydroxyl group and is situated between the ester and ketone functional groups, undergoes deprotonation to generate intermediate (D), which acts as a nucleophile and attacks the alkyl halide, forming the *O*-alkylated intermediate (E). After alkylation, the base deprotonates the active methylene proton, which then acts on the carbonyl carbon of the benzoyl group, forming intermediate (G). Finally, this intermediate undergoes dehydrogenation to yield compound 4.

## Conclusion

In summary, an efficient and transition metal-free strategy for the synthesis of furo[3,2-*e*]indolizine derivatives has been developed. This method employs readily accessible starting materials and proceeds under mild, base-mediated conditions through a tandem Knoevenagel condensation, *O*-alkylation, and cyclization sequence. A broad range of *para*-, *meta*-, and *ortho*-substituted 1-(2-oxo-2-phenylethyl)-1*H*-pyrrole-2-carbaldehyde were successfully converted to the corresponding furoindolizine in good to excellent yields. The operational simplicity, metal-free nature, and broad substrate scope highlights the utility of this method for rapid access to structurally diverse furoindolizine frameworks.

## Conflicts of interest

The authors declare no competing financial interest.

## Supplementary Material

RA-016-D6RA00540C-s001

## Data Availability

The authors declare that all the required spectral data available in supporting information (SI). Supplementary information: the synthetic procedure, characterisation details, and spectral information. See DOI: https://doi.org/10.1039/d6ra00540c.
