# Supplementary material for: A divergent synthetic approach to tricyclic Furo[3,2-e]indolizines via base-mediated tandem annulations of diethyl malonate with aromatic bromomethyls
Source: RSC Adv. 2026 Feb 19;16(11):9851–7. doi: 10.1039/d6ra00540c (PMC12917731; doi:10.1039/d6ra00540c)
Supplement: RA-016-D6RA00540C-s001 [file RA-016-D6RA00540C-s001.pdf]

Supplementary Information (SI) for RSC Advances.
This journal is © The Royal Society of Chemistry 2026

Supporting Information
A Divergent Synthetic Approach to Tricyclic Furo[3,2-*e*]indolizines *via* Base-Mediated Tandem Annulations of Diethyl Malonate with Aromatic Bromomethyls

Sandya Tambi Dorai, **Sandeep Chandrashekharappa***

Affiliation: Department of Medicinal Chemistry, National Institute of Pharmaceutical Education and Research Raebareli, Transit Campus, Bijnor-Sisendi Road, Sarojini Nagar, Near CRPF Base Camp, Lucknow (UP)-226002,

*Corresponding author: **Dr. Sandeep Chandrashekharappa**,
E-mail: c.sandeep@niperraebareli.edu.in and c.sandeepniper@gmail.com
Fax: 91-522-2975587; Tel: 91-522-2499703

Experimental section

The required chemicals are received from Sigma-Aldrich and TCI, India, and are used as such without further purification. All the reactions are carried out in hot-air-dried glassware at room temperature. Chemical reactions are monitored on thin layer chromatography (TLC). TLC is performed on sigma-Aldrich silica gel 60 F_{254} on TLC aluminum foils with ethyl acetate and hexane (2:8) as the solvent system and visualization with UV-light chamber. Flash chromatography using silica gel (230-400 mesh size) is used for the purification of compounds. NMR spectra are recorded using Jeol Nuclear Magnetic Resonance-ECZR series spectrometers, operating at 500 MHz and 125 MHz respectively, using tetramethyl silane (TMS) as internal standard at ambient temperature using DMSO-d_6 and $CDCl_3$ as a solvent for products. Chemical shift values are measured in δ parts per million. The peak multiplicities are given as follows; s, singlet; d, doublet; dd, double doublet; t, triplet; q, quartet; m, multiplet. *J* values are given in Hertz. High-resolution mass spectrometry (HRMS) analyses were performed using an AGILENT mass spectrometer equipped with a Quadrupole Time of Flight (QTOF) detector.

1. Synthesis of ethyl 2-(4-nitrophenyl)-1-phenylfuro[3,2-*e*]indolizine-4-carboxylate and ethyl 2-(4-cyanophenyl)-1-phenylfuro[3,2-*e*]indolizine-4-carboxylate using various substituted intermediates **(1a-k)**

1.1 General reaction

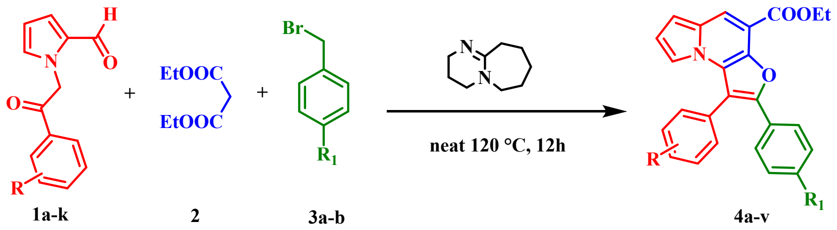

Table S1. Optimization of reaction conditions for the synthesis of ethyl 2-(4-nitrophenyl)-1-phenylfuro[3,2-*e*]indolizine-4-carboxylate (4a)

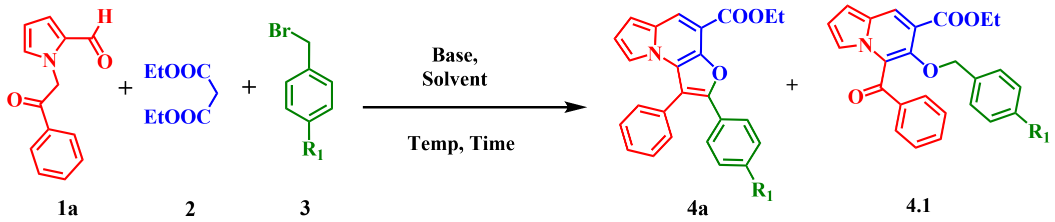

Entry	Base (equiv)	Solvent	Time (h)	Temp (°C)	R_1	Yield[b] % (4.1)	Yield[b] % (4a)
1	Et_3N (3.0)	-	12	110	Br	-	-
2	Et_3N (3.0)	Ethanol	12	80	Br	-	-
3	DIPEA (2.0)	-	24	100	Br	-	-
4	DIPEA (3.0)	Ethanol	24	80	Br	-	-
5	Piperidine (Cat.)	-	24	rt	Br	-	-
6	Piperidine (1.0)	-	24	100	Br	trace	-
7	Piperidine (2.0)	Ethanol	24	80	Br	-	-
8	K_2CO_3 (1.0)	CH_3CN	48	rt	Br	20	-
9	K_2CO_3 (1.5)	CH_3CN	24	85	Br	70	-
10	K_2CO_3 (1.5)	DMF	24	120	Br	72	-
11	K_2CO_3 (2.0)	CH_3CN	12	85	Br	69	-
12	Cs_2CO_3 (2.0)	CH_3CN	12	85	Br	78	-
13	KtBuO (1.0)	CH_3CN	24	85	Br	58	-
14	KtBuO (2.0)	DMF	12	120	Br	67	-
15	KtBuO (2.0)	DMSO	12	120	Br	54	-
16	NaH (2.0)	DMF	24	120	Br	58	-
17	NaH (2.5)	CH_3CN	12	85	Br	61	-
18	NaH (2.5)	DMSO	12	120	Br	50	-
19	DBU (3.0)	CH_3CN	24	85	Br	68	-
20	KtBuO (1.0)	CH_3CN	24	85	NO_2	10	-
21	KtBuO (2.0)	DMF	12	120	NO_2	42	trace
22	KtBuO (4.0)	DMSO	12	120	NO_2	52	trace
23	DBU (2.0)	DMF	24	120	NO_2	-	20
24	DBU (2.5)	DMF	24	120	NO_2	-	58
25	DBU (3.0)	-	12	120	NO_2	-	80
26	DBU (3.0)	CH_3CN	24	85	NO_2	-	72

1.1 General procedure for the synthesis of ethyl 2-(4-nitrophenyl)-1-phenylfuro[3,2-e]indolizine-4-carboxylate and ethyl 2-(4-cyanophenyl)-1-phenylfuro[3,2-*e*]indolizine-4-carboxylate analogues **(4a-v)**

In a clean, dry sealed tube, compound **1a** (0.2 g, 0.93 mmol) and diethylmalonate **2** (0.3 g, 1.88 mmol) was taken. To this mixture, DBU (0.42 g, 2.81 mmol) was added dropwise, and the

reaction mixture was stirred at room temperature for 5 min. Subsequently, compound **3** (0.2 g, 0.93 mmol) was added to the reaction mixture, and the sealed tube was placed in a preheated oil bath at 120 °C for 12 h, and the progress of the reaction was monitored by thin-layer chromatography (TLC). After completion, the reaction mixture was allowed to cool to room temperature and then diluted with ethyl acetate (20 mL). The organic content was extracted with ethyl acetate (25 mL×2) and washed with water (50 mL×2) and brine (10 mL), dried over anhydrous sodium sulphate, filtered, and concentrated under reduced pressure using rotary evaporator. The resulting crude residue was purified by flash column chromatography using ethyl acetate and hexane (1:9) as eluent to afford the desired product **4a** with 80% yield. The purified compound was characterized by ^{1}H NMR, ^{13}C NMR, and HRMS. The remaining derivatives (**4b-v**) were prepared by using the same protocol.

2. Synthesis of Ethyl 2-benzoyl-1-phenylfuro[3,2-*e*]indolizine-4-carboxylate analogues using different substituted compounds (1a-g) and phenacyl bromides (5a-r)

2.1 General reaction

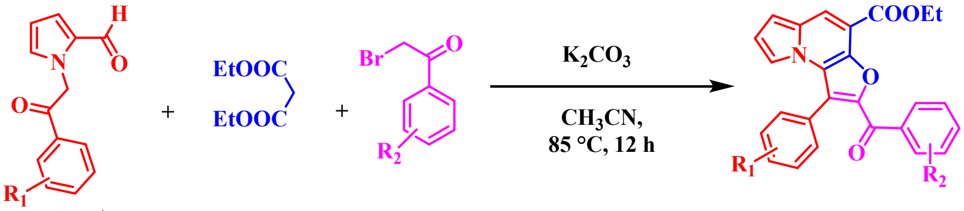

Table S2. Optimization of reaction conditions for the synthesis of ethyl 2-benzoyl-1-phenylfuro[3,2-*e*]indolizine-4-carboxylate analogue **(6m)**

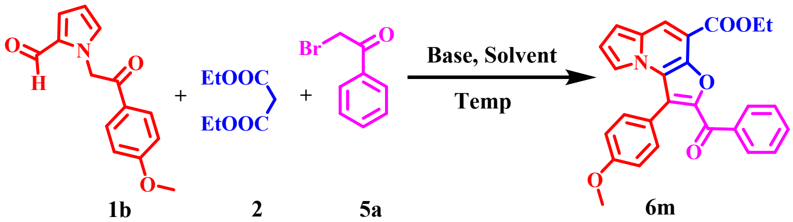

Entry	Base (equiv.)	Solvent	Time (h)	Temp (°C)	Yield %
1	Piperidine (Cat.)	-	24	rt	-
2	Piperidine (Cat.)	-	12	100	-
3	Piperidine (Cat.)	Ethanol	24	80	trace
4	K_2CO_3 (1.0)	CH_3CN	12	rt	20
**5**	**K_2CO_3 (1.5)**	**CH_3CN**	**12**	**85**	**80**
6	K_2CO_3 (2.5)	DMF	8	110	50
7	Cs_2CO_3 (1.5)	CH_3CN	12	85	67
8	Cs_2CO_3 (2.5)	DMF	24	100	62
9	NaH (2.0)	DMF	6	100	30
10	*t*-BuOK (2.0)	CH_3CN	12	85	40
11	DBU (1.5)	DMF	12	100	52

2.2 General procedure for the synthesis of ethyl 2-benzoyl-1-phenylfuro[3,2-e]indolizine-4-carboxylate analogues (6a-ac)

In a clean, oven-dried round-bottom flask, compound **1a** (0.2 g, 0.93 mmol), diethylmalonate **2** (0.3 g, 1.88 mmol) was dissolved in acetonitrile (5 mL), and K_2CO_3 (0.19 g, 1.41 mmol) was added. The reaction mixture was stirred at room temperature for 10 min, followed by the addition of compound **5a** (0.21 g, 0.93 mmol). Then the mixture was refluxed at 80 °C for 12 h, and the progress of the reaction was monitored by TLC. After completion, the reaction mixture was allowed to cool to room temperature, the solvent was removed under reduced pressure, and the resulting crude was extracted with ethyl acetate (25 mL×2) and washed with water (50 mL×2) and brine (10 mL), dried over anhydrous sodium sulphate, filtered, and concentrated under reduced pressure using rotary evaporator. The resulting crude residue was purified by flash column chromatography using ethyl acetate and hexane (1:9) as eluent to afford the desired product **6a** with 63% yield. The purified compound was characterized by ^{1}H NMR, ^{13}C NMR, and HRMS. The remaining derivatives (**6b-ac**) were prepared by using the same protocol.

3. Synthesis of ethyl 2-benzoyl-6,8-dimethyl-1-phenylfuro[3,2-*e*]indolizine-4-carboxylate analogues using 3,5-dimethyl substituted pyrrole-2-carbaldehyde (8a-b)

3.1 General reaction

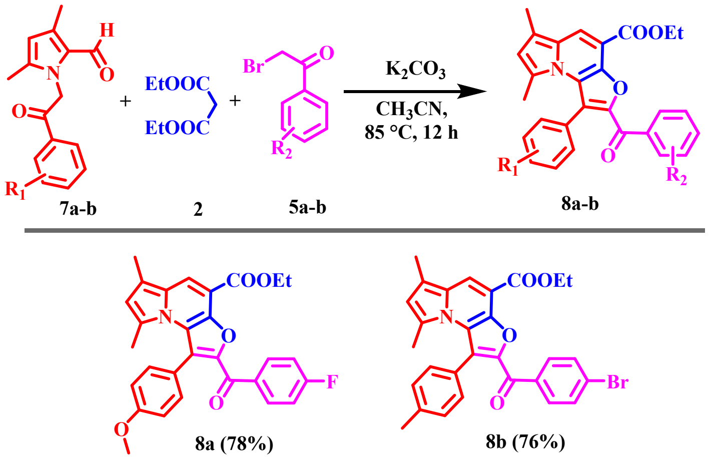

3.2 General procedure for the synthesis of ethyl 2-benzoyl-6,8-dimethyl-1-phenylfuro[3,2-*e*]indolizine-4-carboxylate analogues (8a-b)

In a clean, oven-dried round-bottom flask, compound **7a** (0.2 g, 0.74 mmol), diethylmalonate **2** (0.23 g, 1.49 mmol) was dissolved in acetonitrile (5 mL), and K_2CO_3 (0.15 g, 1.11 mmol) was added. The reaction mixture was stirred at room temperature for 10 min, followed by the addition of compound **5a** (0.17 g, 0.74 mmol). Then the mixture was refluxed at 80 °C for 12 h, and the progress of the reaction was monitored by TLC. After completion, the reaction

mixture was allowed to cool to room temperature, the solvent was removed under reduced pressure, and the resulting crude was extracted with ethyl acetate (25 mL×2) and washed with water (50 mL×2) and brine (10 mL), dried over anhydrous sodium sulphate, filtered, and concentrated under reduced pressure using rotary evaporator. The resulting crude residue was purified by flash column chromatography using ethyl acetate and hexane (1:9) as eluent to afford the desired product **8a** with 78% yield. The purified compound was characterized by ^{1}H NMR, ^{13}C NMR, and HRMS.

4. Plausible mechanism

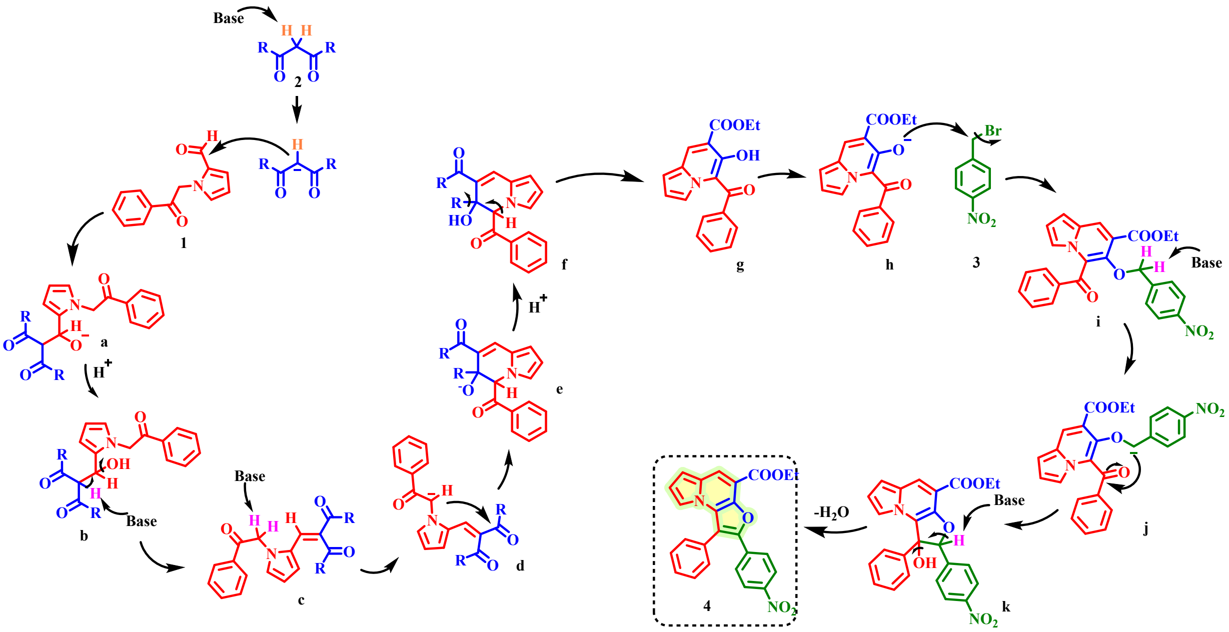

4.1 Density Function Theory (DFT) study

DFT calculations were performed using the Gaussian 16 program package. All structures were optimized in the gas phase using the B3LYP functional with the 6-31G(d) basis set. Harmonic vibrational frequency calculations confirmed all optimized structures are true minima and provided zero-point energy and thermal corrections to Gibbs free energies at 298.15 K and 1 atm. Reported Gibbs free energies include electronic, zero-point, and thermal contributions. Relative Gibbs free energies (ΔG) were calculated with respect to the reference reactant.

The computed Gibbs free energy profile shows that the formation of intermediate **M1** is associated with a positive free energy change (ΔG = +13.87 kcal/mol), indicating reduced thermodynamic stability relative to the reactant complex **1a+2** (ΔG = 0 kcal mol^{-1}). The free energy profile suggests that **M2** is thermodynamically stabilized relative to the reactant complex, with a negative Gibbs free energy of ΔG = −4.46 kcal mol^{-1}, suggesting that **M2** can

form readily and acts as a relatively stable intermediate. However, further progression along the **M2→M3A→M4A** pathway is thermodynamically unfavorable. In particular, the formation of **M3A** requires an increase in free energy to ΔG = +18.45 kcal mol^{-1}, and the subsequent intermediate **M4A** is exceptionally high in energy (ΔG = +196.45 kcal mol^{-1}), rendering this pathway inaccessible, as shown in **Figure S1**. In contrast, the alternative pathway *via* **M3B** (ΔG = +17.08 kcal mol^{-1}) leads to the formation of **M4B**, which has a thermodynamically favorable negative free energy (ΔG = −14.71 kcal mol^{-1}), identifying it as the most stable and energetically preferred product, as shown in **Figure S2**.

4.2 Plausible mechanism based on the DFT study

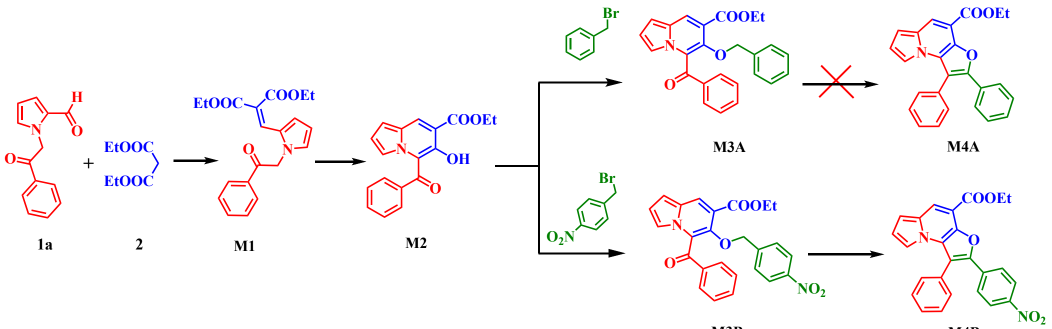

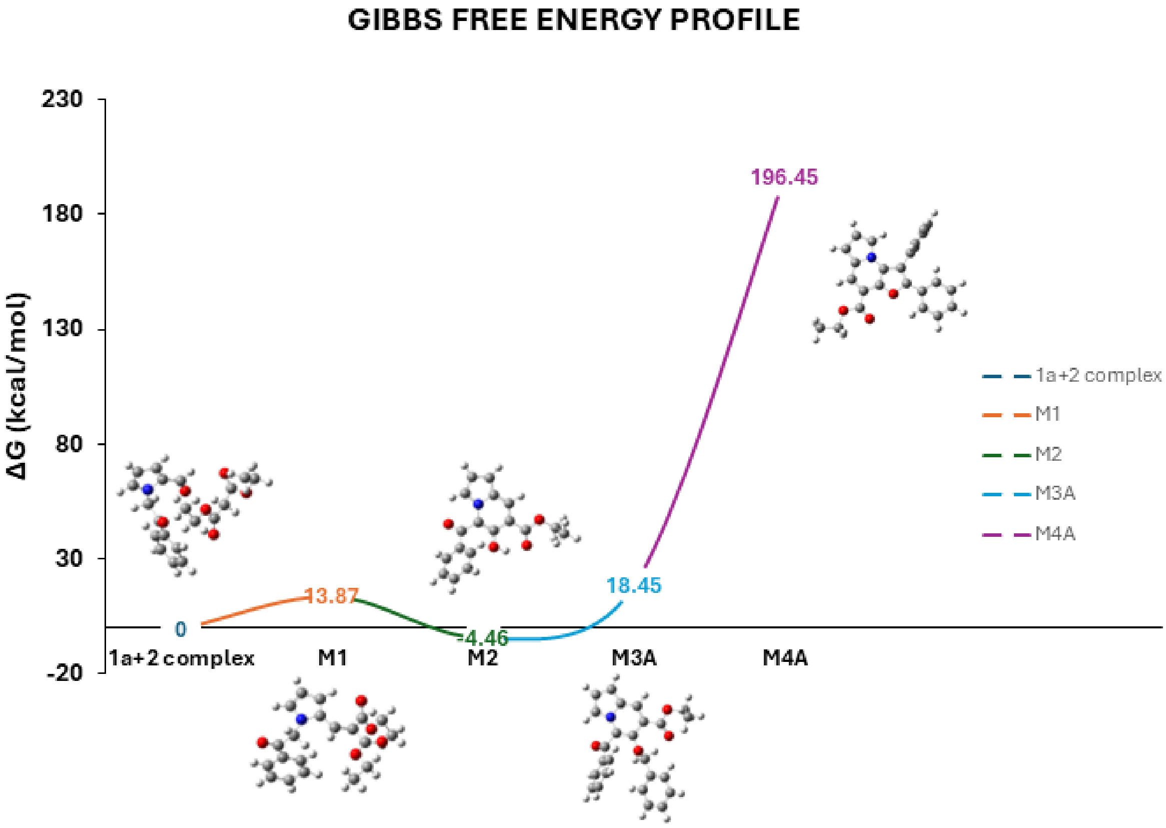

Figure S1. Computed Gibbs free energy profile (ΔG, kcal mol^{-1}) for the formation of furo[3,2-*e*]indolizine using benzyl bromide as the reactant.

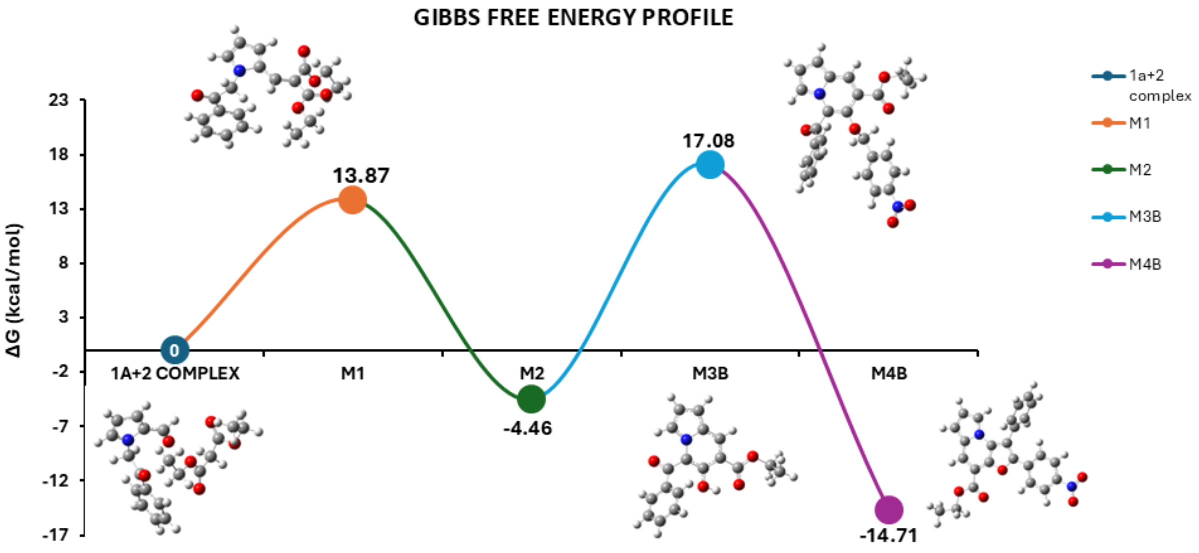

Figure S2. Computed Gibbs free energy profile (ΔG, kcal mol^{-1}) for the formation of furo[3,2-*e*]indolizine using 4-NO_2 benzyl bromide as the reactant.

Table S3. Calculated electronic and Gibbs free energies at the B3LYP level with 6-31G(d) basis set for all structures in synthesis of furo[3,2-*e*]indolizine

Structure	SCF Energy (Hartree)	ZPE (Hartree)	Thermal Corr. to G (Hartree)	Gibbs Free Energy (Hartree)	ΔG vs Reactant (Hartree)	ΔG vs Reactant (kcal mol⁻¹)
**1a+2**	-1282.0949	0.405042	0.340938	-1281.7539	0	0
**M1**	-1205.6443	0.379422	0.317887	-1205.3264	0.022096	13.87
**M2**	-1050.6024	0.296834	0.247671	-1050.3547	0.007105	-4.46
**M3A**	-1320.9419	0.40573	0.346115	-1320.5958	0.029394	18.45
**M3B**	-1525.4448	0.408366	0.344361	-1525.1005	0.027223	17.08
**M4A**	-1244.2026	0.382251	0.325263	-1243.8773	0.313066	196.45
**M4B**	-1449.0398	0.382156	0.3213	-1448.7185	-0.023444	-14.71

4.3 Cartesian coordinates correspond to fully optimized gas-phase geometries at the B3LYP/6-31G(d) level of theory.

4.1.1 Cartesian coordinates (Å) of optimized structure **1a+2** complex (gas phase, B3LYP/6-31G(d))

C 0.977743 0.691574 0.000000

C	2.724213	-0.129408	1.674041
C	5.103660	0.025873	1.440639
H	5.767142	0.882364	1.583918
H	5.115327	-0.598903	2.337345
C	5.480821	-0.770788	0.200518
H	6.503706	-1.152862	0.306838
H	4.808267	-1.622906	0.064006
H	5.440472	-0.141130	-0.694938
O	3.786639	0.620488	1.306727
O	2.810234	-1.249839	2.135263
C	0.932633	-0.471257	-2.080840
H	-0.159497	-0.495327	-2.073995
H	1.265047	0.425927	-2.612811
C	1.523467	-1.739116	-2.667235
H	1.199899	-1.842971	-3.709690
H	2.618711	-1.714272	-2.647790
H	1.181985	-2.619675	-2.113572
O	0.292928	1.587537	-0.451441
O	1.387399	-0.376630	-0.701781
C	-1.051306	-3.687972	1.179262
C	-0.486634	-4.881817	0.722535
C	-1.465048	-5.562301	-0.027787
C	-2.605336	-4.767289	-0.013100
N	-2.364145	-3.644867	0.721091
H	0.527948	-5.201927	0.923414
H	-1.366565	-6.517726	-0.525079
H	-3.572659	-4.925285	-0.471903
C	-2.987471	-1.325208	0.057570
C	-3.738965	-0.067092	0.339115
C	-4.892182	-0.042908	1.142489
C	-3.272333	1.130422	-0.230219
C	-5.575367	1.154495	1.359572
H	-5.271246	-0.953278	1.597874
C	-3.948107	2.326797	0.000336
H	-2.366591	1.113063	-0.827327
C	-5.103077	2.340723	0.790237
H	-6.470817	1.162294	1.975362
H	-3.569608	3.249736	-0.430460
H	-5.630625	3.274716	0.966939
C	-0.413070	-2.653046	1.957086
H	0.644135	-2.853765	2.208555
O	-0.947979	-1.602955	2.321995
O	-2.153334	-1.398255	-0.829698
C	-3.307856	-2.561016	0.921879
H	-4.304808	-2.934090	0.658678
H	-3.317979	-2.274367	1.975180
C	1.427500	0.625883	1.454130
H	0.635697	0.115675	2.015169
H	1.517241	1.653645	1.814195

4.1.2 Cartesian coordinates (Å) of optimized structure **M1** (gas phase, B3LYP/6-31G(d))

C	1.600655	3.016459	0.318240
C	2.861306	3.798277	0.731904
C	3.094558	4.087424	2.083166
C	3.775247	4.220585	-0.242924
C	4.241752	4.798878	2.459600
H	2.396745	3.764982	2.827469
C	4.922438	4.932043	0.133509
H	3.597154	3.999815	-1.274643
C	5.155691	5.221188	1.484772
H	4.419846	5.019645	3.491319
H	5.620250	5.254487	-0.610794
H	6.031598	5.764401	1.772187
O	1.391205	2.756815	-0.895138
C	0.596323	2.552386	1.389480
H	0.869374	1.577534	1.735900
H	-0.385970	2.518447	0.966576
N	0.612022	3.495316	2.517104
C	0.203206	4.867637	2.130925
C	-0.361055	3.144299	3.579854
C	-0.607927	5.390112	3.075535
H	0.502962	5.368185	1.233987
C	-0.978233	4.259143	4.026418
H	-0.941552	6.404989	3.135696
H	-1.620514	4.331337	4.879157
C	-0.632465	1.718150	4.093697
H	-1.256292	1.200178	3.395523
C	-1.340556	1.790633	5.459330
C	-1.465024	0.373408	6.048890
C	-2.744784	2.396313	5.277957
C	-0.147375	-1.312709	7.369903
H	-0.462609	-0.893048	8.302326
H	-0.854297	-2.051193	7.053988
C	1.235875	-1.967120	7.543013
H	1.178673	-2.729271	8.291848
H	1.543636	-2.401457	6.614824
H	1.946405	-1.225319	7.842645
O	-0.059483	-0.199432	6.309484
C	-3.777537	3.942476	6.971199
H	-4.658636	3.391067	6.717205
H	-3.350601	3.540179	7.866063
C	-4.144535	5.420940	7.197154
H	-4.569628	5.823790	6.301661
H	-4.855204	5.494307	7.993688
H	-3.263764	5.971967	7.453108
O	-2.756883	3.836634	5.822868
O	-2.104662	0.424106	7.131417
O	-3.585273	1.702676	5.907254

4.1.3 Cartesian coordinates (Å) of optimized structure **M2** (gas phase, B3LYP/6-31G(d))

C	-0.812786	3.366546	0.228117
C	0.119277	4.349189	0.586081

C	1.386370	3.766225	0.618883
C	1.225066	2.409885	0.279847
N	-0.155859	2.186307	0.047686
H	-1.873915	3.442528	0.063092
H	-0.127145	5.384452	0.782436
H	2.331179	4.241258	0.846659
C	2.119046	1.354222	0.129395
H	3.175574	1.536948	0.289697
C	-0.652037	0.900517	-0.250505
C	0.265030	-0.128477	-0.427043
C	1.682145	0.087547	-0.231841
C	-2.113356	0.761088	-0.496002
C	-2.846983	-0.470650	-0.069238
C	-4.012930	-0.823910	-0.766340
C	-2.472754	-1.208449	1.063482
C	-4.776399	-1.914903	-0.353720
H	-4.305819	-0.234510	-1.629797
C	-3.250815	-2.284439	1.490815
H	-1.579689	-0.932884	1.616885
C	-4.398677	-2.645359	0.778343
H	-5.668654	-2.192478	-0.909033
H	-2.960724	-2.843105	2.376967
H	-4.998657	-3.490766	1.106020
O	-2.739893	1.693555	-1.004527
C	2.621818	-1.023835	-0.418109
C	4.908524	-1.735923	-0.365609
H	5.812119	-1.187494	-0.641871
H	4.609458	-2.386559	-1.190362
C	5.100819	-2.516957	0.925312
H	4.193541	-3.068060	1.190480
H	5.914435	-3.240901	0.795164
H	5.366168	-1.848693	1.751747
O	2.279187	-2.167471	-0.740236
O	3.907596	-0.692572	-0.209512
O	-0.188650	-1.332817	-0.824074
H	0.589061	-1.943349	-0.886295

4.1.4 Cartesian coordinates (Å) of optimized structure **M3A** (Benzyl bromide) (gas phase, B3LYP/6-31G(d))

C	-2.4664	-3.4434	-0.6612
C	-3.8542	-3.5741	-0.6318
C	-4.4199	-2.3038	-0.4468
C	-3.3623	-1.3885	-0.3614
N	-2.1568	-2.1208	-0.4949
H	-1.6979	-4.1820	-0.8216
H	-4.3860	-4.5095	-0.7475
H	-5.4697	-2.0510	-0.3844
C	-3.2916	-0.0009	-0.1842
H	-4.2172	0.5528	-0.0794
C	-0.9188	-1.4826	-0.4014

C	-0.8688	-0.1119	-0.2692
C	-2.0799	0.6695	-0.1527
C	0.3064	-2.3521	-0.5403
C	1.3570	-2.3478	0.5166
C	2.5679	-3.0109	0.2488
C	1.1533	-1.7712	1.7804
C	3.5610	-3.0821	1.2223
H	2.7101	-3.4635	-0.7277
C	2.1439	-1.8551	2.7586
H	0.2227	-1.2579	1.9986
C	3.3497	-2.5052	2.4805
H	4.4984	-3.5872	1.0048
H	1.9775	-1.4080	3.7349
H	4.1239	-2.5624	3.2414
O	0.3834	-3.1038	-1.5093
C	-2.0557	2.1454	0.0097
C	-3.3749	4.0538	0.6132
H	-4.4412	4.2440	0.4646
H	-2.8028	4.5469	-0.1766
C	-2.9184	4.5026	1.9939
H	-1.8496	4.3138	2.1330
H	-3.0941	5.5796	2.1068
H	-3.4761	3.9799	2.7788
O	-1.1097	2.8760	-0.2314
O	-3.2491	2.6187	0.4554
O	0.3683	0.4689	-0.1724
C	0.9328	0.9625	-1.4208
H	1.0449	0.1176	-2.1120
H	0.2359	1.6901	-1.8471
C	2.2654	1.5979	-1.1272
C	3.4558	0.9853	-1.5368
C	2.3269	2.8227	-0.4448
C	4.6920	1.5846	-1.2758
H	3.4167	0.0343	-2.0640
C	3.5602	3.4187	-0.1774
H	1.4021	3.2963	-0.1280
C	4.7458	2.8023	-0.5938
H	5.6086	1.0998	-1.6026
H	3.5979	4.3681	0.3514
H	5.7055	3.2707	-0.3888

4.1.5 Cartesian coordinates (Å) of optimized structure **M3B** (4-NO_2 Benzyl bromide) (gas phase, B3LYP/6-31G(d))

C	-4.363764	-2.141508	-0.765261
C	-5.674343	-1.666347	-0.787390
C	-5.650011	-0.276108	-0.602372
C	-4.307327	0.100118	-0.464900
N	-3.525874	-1.078365	-0.567289
H	-3.979227	-3.138347	-0.908898

H	-6.549976	-2.284670	-0.936050
H	-6.493227	0.400634	-0.572436
C	-3.657935	1.324181	-0.262675
H	-4.261357	2.220565	-0.182233
C	-2.139447	-1.030352	-0.424559
C	-1.516699	0.186888	-0.263843
C	-2.277893	1.412287	-0.173821
C	-1.393038	-2.337010	-0.553343
C	-0.484553	-2.794343	0.534383
C	0.349467	-3.898328	0.281232
C	-0.480851	-2.206733	1.809795
C	1.182649	-4.393521	1.280914
H	0.329360	-4.352838	-0.704448
C	0.344079	-2.713294	2.813883
H	-1.122233	-1.356312	2.017118
C	1.180097	-3.802201	2.550252
H	1.832543	-5.239993	1.075565
H	0.338757	-2.255557	3.799329
H	1.828719	-4.190326	3.331472
O	-1.597603	-3.028626	-1.547705
C	-1.628750	2.732642	0.023028
C	-2.028265	5.035423	0.560012
H	-2.885116	5.669524	0.318175
H	-1.225761	5.216297	-0.159161
C	-1.557364	5.259206	1.989497
H	-0.696460	4.625295	2.222522
H	-1.255918	6.306219	2.116533
H	-2.359800	5.044434	2.703724
O	-0.439639	2.970525	-0.117310
O	-2.527600	3.686438	0.373185
O	-0.151023	0.179175	-0.117842
C	0.609391	0.405508	-1.327939
H	0.399406	-0.400451	-2.042315
H	0.302986	1.362594	-1.763110
C	2.071810	0.434973	-0.964942
C	2.967782	-0.479065	-1.533903
C	2.549867	1.395118	-0.058348
C	4.325086	-0.443904	-1.214517
H	2.604446	-1.229597	-2.231280
C	3.899747	1.439643	0.277786
H	1.852649	2.104885	0.374824
C	4.769714	0.516614	-0.307769
H	5.028141	-1.145517	-1.647242
H	4.284969	2.174060	0.975145
N	6.196659	0.557643	0.043008
O	6.868129	-0.378940	-0.679169
O	6.697100	1.790782	-0.237217

4.1.6 Cartesian coordinates (Å) of optimized structure **M4A** (Benzyl bromide) (gas phase, B3LYP/6-31G(d))

C	-0.485350	-0.114414	-0.175875
C	0.773252	-0.462215	-0.691716
C	1.288138	0.662287	-1.367186
C	0.363408	1.703909	-1.276396
N	-0.718805	1.247522	-0.555885
H	1.245159	-1.426039	-0.579587
H	2.238481	0.723910	-1.875578
H	0.400712	2.706773	-1.663154
C	-1.456599	-0.809474	0.575439
H	-1.262452	-1.836633	0.852816
C	-1.904575	1.861247	-0.174790
C	-2.828428	1.134870	0.563763
C	-2.650975	-0.217883	0.969060
C	-3.663826	-0.938807	1.751127
C	-4.228794	-3.054922	2.815784
H	-4.411781	-2.550653	3.769555
H	-5.177240	-3.099874	2.271833
C	-3.590529	-4.420775	2.990440
H	-2.637371	-4.340214	3.522142
H	-4.254932	-5.072576	3.495053
C	-1.974308	-4.930165	2.337476
H	-1.279087	-5.591923	2.863258
O	-0.408498	-1.297194	-0.006907
O	-4.746044	-0.524627	0.006956
O	-3.157426	-2.180161	0.021197

4.1.7 Cartesian coordinates (Å) of optimized structure **M4B** (4-NO_2 Benzyl bromide) (gas phase, B3LYP/6-31G(d))

C	0.149300	0.707500	0.003900
C	-0.234400	-0.624700	0.005500
C	-1.641300	-0.853800	0.007500
C	-2.591900	0.153200	0.007800
C	-0.789300	1.762600	0.002600
H	1.202600	0.961500	0.002500
C	-0.664500	3.159000	-0.001400
H	0.272300	3.699700	-0.003400
C	-2.878900	2.652100	-0.000200
H	-3.957300	2.660900	-0.000900
C	-1.961400	3.700400	-0.003100
H	-2.223900	4.750300	-0.006400
N	-2.177900	1.475100	0.003600
C	-3.885700	-0.465300	0.008900
C	-3.629900	-1.826700	0.010000
O	-2.273900	-2.054200	0.009100
C	-4.455800	-3.033100	0.012500
C	-3.832800	-4.298100	-0.032900
C	-5.863100	-2.977900	0.061400
C	-4.596000	-5.464300	-0.031700
H	-2.750200	-4.357600	-0.068600
C	-6.618200	-4.150300	0.061800

H	-6.370300	-2.021300	0.101200
C	-5.991900	-5.399200	0.014900
H	-4.095400	-6.428600	-0.067400
H	-7.702700	-4.084700	0.100400
C	-5.198300	0.233400	0.002600
C	-5.836800	0.544000	-1.209100
C	-5.807000	0.621800	1.207300
C	-7.058800	1.221700	-1.215800
H	-5.372400	0.248300	-2.146300
C	-7.028900	1.299700	1.200700
H	-5.319900	0.385900	2.150100
C	-7.657700	1.601500	-0.010900
H	-7.541400	1.452800	-2.162000
H	-7.488200	1.591300	2.141800
H	-8.608100	2.129000	-0.016300
C	0.738800	-1.739400	0.003400
C	3.046500	-2.322400	0.006800
H	2.908000	-2.958000	0.887500
H	2.913400	-2.947700	-0.882000
C	4.393500	-1.623100	0.015000
H	5.195900	-2.370300	0.013200
H	4.513800	-0.988600	-0.869800
H	4.508100	-0.998700	0.907700
O	0.443400	-2.921300	-0.003400
O	2.023300	-1.299500	0.009600
N	-6.793500	-6.631500	0.015900
O	-6.481600	-7.374300	1.111600
O	-6.525100	-7.347700	-1.108700

5 Characterization details

Ethyl 2-(4-nitrophenyl)-1-phenylfuro[3,2-*e*]indolizine-4-carboxylate (4a)

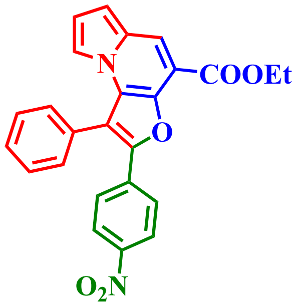

Orange solid, 80%, ^{1}H NMR (500 MHz, $CDCl_3$) δ 8.16 (s, 1H), 8.11 (d, J = 8.9 Hz, 2H), 7.68 (d, J = 8.9 Hz, 2H), 7.64 – 7.61 (m, 3H), 7.54 (dd, J = 6.5, 2.9 Hz, 2H), 6.89 (d, J = 2.0 Hz, 1H), 6.84 (d, J = 4.0 Hz, 1H), 6.69 (dd, J = 4.0, 2.7 Hz, 1H), 4.54 – 4.50 (m, 2H), 1.53 (t, J = 7.1 Hz, 3H); ^{13}C {^{1}H} NMR (125 MHz, $CDCl_3$) δ 164.2, 146.8, 146.6, 136.2, 130.9, 130.8, 130.3, 129.9, 129.7, 125.7, 125.2, 124.1, 121.7, 117.1, 115.1, 114.7, 109.4, 106.9, 61.3, 14.6; HRMS (ESI-TOF) m/z: $[M+H]^+$ calculated for $C_{25}H_{19}N_2O_5$: 427.1294; found $[M+H]^+$: 427.1297.

Ethyl 1-(4-methoxyphenyl)-2-(4-nitrophenyl)furo[3,2-*e*]indolizine-4-carboxylate (4b)

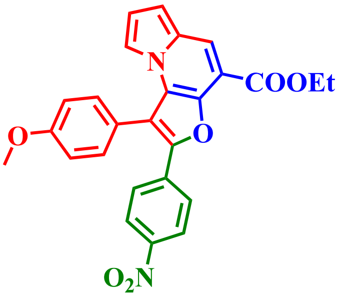

Orange solid, 69%,^{1}H NMR (500 MHz, $CDCl_3$) δ 8.15 (s, 1H), 8.12 (d, J = 9.0 Hz, 2H), 7.72 (d, J = 9.0 Hz, 2H), 7.44 (d, J = 8.6 Hz, 2H), 7.14 (d, J = 8.6 Hz, 2H), 6.99-6.96 (m, 1H), 6.83 (d, J = 3.6 Hz, 1H), 6.71 (dd, J = 3.9, 2.7 Hz, 1H), 4.52 (q, J = 7.1 Hz, 2H), 3.96 (s, 3H), 1.53 (t, J = 7.1 Hz, 3H); ^{13}C {^{1}H} NMR (125 MHz, $CDCl_3$) δ 164.3, 160.6, 146.9, 146.5, 140.4, 136.4, 131.5, 130.9, 125.7, 125.5, 124.1, 122.4, 121.6, 116.9, 115.4, 115.1, 114.8, 109.4, 106.9, 61.2, 55.5, 14.6; HRMS (ESI-TOF) m/z: $[M+H]^+$ calculated for $C_{26}H_{21}N_2O_6$: 457.1400; found $[M+H]^+$: 457.1399.

Ethyl 2-(4-nitrophenyl)-1-(p-tolyl)furo[3,2-*e*]indolizine-4-carboxylate (4c)

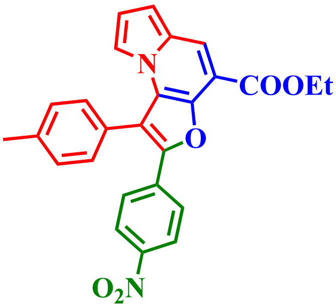

Orange solid, 68%, ^{1}H NMR (500 MHz, $CDCl_3$): δ 8.16 (s, 1H), 8.12 (d, J = 9.0 Hz, 2H), 7.71 (d, J = 9.0 Hz, 2H), 7.42 (s, 4H), 6.95 (d, J = 2.0 Hz, 1H), 6.83 (d, J = 3.6 Hz, 1H), 6.70 (dd, J = 4.0, 2.7 Hz, 1H), 4.52 (q, J = 7.1 Hz, 2H), 2.54 (s, 3H), 1.53 (t, J = 7.1 Hz, 3H); ^{13}C {^{1}H} NMR (125 MHz, $CDCl_3$) δ 164.3, 146.9, 146.5, 140.5, 139.7, 136.5, 130.9, 130.7, 130.1, 127.6, 125.7, 125.3, 124.1, 121.6, 117.2, 115.1, 114.8, 109.4, 106.9, 61.2, 21.6, 14.6; HRMS (ESI-TOF) m/z: $[M+H]^+$ calculated for $C_{26}H_{21}N_2O_5$: 441.1450; found $[M+H]^+$: 441.1451.

Ethyl 1-(4-fluorophenyl)-2-(4-nitrophenyl)furo[3,2-*e*]indolizine-4-carboxylate (4d)

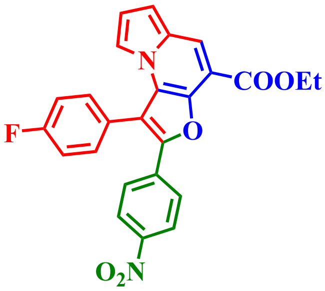

Orange solid, 58%, ^{1}H NMR (500 MHz, $CDCl_3$) δ 8.17 (s, 1H), 8.14 (d, J = 9.0 Hz, 2H), 7.68 (d, J = 9.0 Hz, 2H), 7.54 (dd, J = 8.5, 5.3 Hz, 2H), 7.34 (t, J = 8.5 Hz, 2H), 6.90 (d, J = 2.1 Hz, 1H), 6.85 (d, J = 4.0 Hz, 1H), 6.72 (dd, J = 4.0, 2.7 Hz, 1H), 4.52 (q, J = 7.1 Hz, 2H), 1.53 (t, J = 7.1 Hz, 3H); ^{13}C {^{1}H} NMR (125 MHz, $CDCl_3$) δ 164.13, 163.53 (d, J = 250.4 Hz), 147.02, 146.69, 140.56, 136.00, 132.30 (d, J = 8.1 Hz), 130.96, 126.69 (d, J = 3.5 Hz), 125.74, 125.12, 124.15, 121.75, 117.26 (d, J = 21.6 Hz), 115.96, 115.26, 114.53, 109.40, 107.11, 61.29, 14.59; HRMS (ESI-TOF) m/z: $[M+H]^+$ calculated for $C_{25}H_{18}FN_2O_5$: 445.1199; found $[M+H]^+$: 445.1205.

Ethyl 1-(4-chlorophenyl)-2-(4-nitrophenyl)furo[3,2-*e*]indolizine-4-carboxylate (4e)

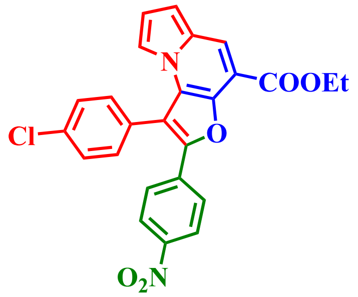

Orange solid, 62%, ^{1}H NMR (500 MHz, $CDCl_3$) δ 8.17 (s, 1H), 8.14 (d, J = 9.1 Hz, 2H), 7.68 (d, J = 9.1 Hz, 2H), 7.62 (d, J = 8.4 Hz, 2H), 7.50 (d, J = 8.4 Hz, 2H), 6.92 (d, J = 2.1 Hz, 1H), 6.85 (d, J = 4.1 Hz, 1H), 6.73 (dd, J = 4.1, 2.7 Hz, 1H), 4.52 (q, J = 7.1 Hz, 2H), 1.53 (t, J = 7.1 Hz, 3H); ^{13}C {^{1}H} NMR (125 MHz, $CDCl_3$) δ 164.1, 146.9, 146.8, 140.6, 136.0, 135.9, 131.8, 130.9, 130.3, 129.3, 125.8, 124.9, 124.2, 121.8, 115.8, 115.3, 114.6, 109.4, 107.2, 61.3, 14.6; HRMS (ESI-TOF) m/z: $[M+H]^+$ calculated for $C_{25}H_{18}ClN_2O_5$: 461.0904; found $[M+H]^+$: 461.0910.

Ethyl 1-(4-bromophenyl)-2-(4-nitrophenyl)furo[3,2-*e*]indolizine-4-carboxylate (4f)

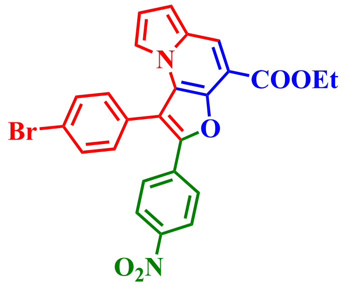

Orange solid, 67%, ^{1}H NMR (500 MHz, $CDCl_3$) δ 8.17 (s, 1H), 8.15 (d, J = 9.1 Hz, 2H), 7.77 (d, J = 8.4 Hz, 2H), 7.68 (d, J = 9.1 Hz, 2H), 7.44 (d, J = 8.4 Hz, 2H), 6.92 (d, J = 2.1 Hz, 1H), 6.85 (d, J = 4.1 Hz, 1H), 6.73 (dd, J = 4.1, 2.7 Hz, 1H), 4.52 (q, J = 7.1 Hz, 2H), 1.53 (t, J = 7.1 Hz, 3H); ^{13}C {^{1}H} NMR (125 MHz, $CDCl_3$) δ 164.1, 146.9, 146.7, 140.7, 135.9, 133.3, 132.1, 132.1, 132.1, 130.9, 129.8, 125.8, 124.9, 124.2, 124.2, 121.8, 121.8, 115.8, 115.3, 109.4, 107.2, 61.3, 14.6; HRMS (ESI-TOF) m/z: $[M+H]^+$ calculated for $C_{25}H_{18}BrN_2O_5$; 505.0399; found $[M+H+2]^+$: 507.0379.

Ethyl 1-(3-methoxyphenyl)-2-(4-nitrophenyl)furo[3,2-*e*]indolizine-4-carboxylate (4g)

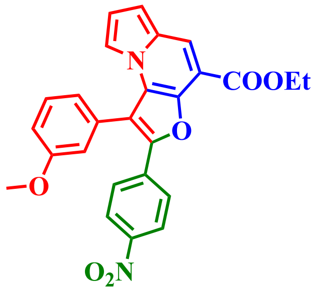

Orange solid, 79%, ^{1}H NMR (500 MHz, CDCl$_3$) δ 8.17 (s, 1H), 8.13 (d, J = 9.1 Hz, 2H), 7.72 (d, J = 9.1 Hz, 2H), 7.53 (d, J = 8.2 Hz, 1H), 7.16 (m, 1H), 7.12 (m, 1H), 7.06-7.03 (m, 1H), 6.95 (d, J = 2.5 Hz, 1H), 6.84 (d, J = 4.0 Hz, 1H), 6.71 (dd, J = 4.1, 2.7 Hz, 1H), 4.52 (q, J = 7.1 Hz, 2H), 3.86 (s, 3H), 1.53 (t, J = 7.1 Hz, 3H); ^{13}C {^{1}H} NMR (125 MHz, CDCl$_3$) δ 164.2, 160.7, 146.8, 146.6, 136.2, 132.1, 131.1, 130.9, 125.7, 124.1, 122.4, 121.7, 116.9, 115.5, 115.3, 115.2, 114.8, 109.4, 106.9, 61.3, 55.5, 14.6; HRMS (ESI-TOF) m/z: [M+H]$^+$ calculated for $C_{26}H_{21}N_2O_6$: 457.1399; found [M+H]$^+$: 457.1401.

Ethyl 2-(4-nitrophenyl)-1-(4-(trifluoromethyl)phenyl)furo[3,2-*e*]indolizine-4-carboxylate (4h)

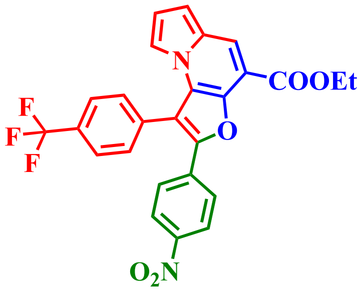

Orange solid, 75%, ^{1}H NMR (500 MHz, CDCl$_3$) δ 8.19 (s, 1H), 8.15 (d, J = 9.0 Hz, 2H), 7.91 (d, J = 8.1 Hz, 2H), 7.72 (d, J = 7.9 Hz, 2H), 7.65 (d, J = 9.0 Hz, 2H), 6.86 (d, J = 4.1 Hz, 1H), 6.84 (d, J = 2.3 Hz, 1H), 6.74 (dd, J = 4.0, 2.7 Hz, 1H), 4.53 (q, J = 7.1 Hz, 2H), 1.53 (t, J = 7.1 Hz, 3H); ^{13}C {^{1}H} NMR (125 MHz, CDCl$_3$) δ 164.05, 147.02, 146.89, 140.80, 135.71, 134.96, 132.11, 131.84, 131.02, 126.88, 126.06 – 125.76 (m), 124.74, 124.34, 124.15, 122.80, 122.08 – 121.78 (m), 115.46, 114.59, 114.45, 109.43, 107.36, 107.22, 61.33, 14.49; HRMS (ESI-TOF) m/z: [M+H]$^+$ calculated for $C_{26}H_{18}F_3N_2O_5$: 495.1168; found: [M+H]$^+$: 495.1168.

Ethyl 2-(4-nitrophenyl)-1-(4-(trifluoromethoxy)phenyl)furo[3,2-*e*]indolizine-4-carboxylate (4i)

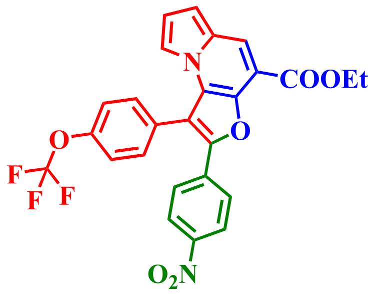

Orange solid, 74%, ^{1}H NMR (500 MHz, $CDCl_3$) δ 8.17 (s, 1H), 8.14 (d, J = 8.9 Hz, 2H), 7.66 (d, J = 8.9 Hz, 2H), 7.61 (d, J = 8.5 Hz, 2H), 7.49 (d, J = 8.1 Hz, 2H), 6.89-6.87 (m, 1H), 6.85 (d, J = 3.9 Hz, 1H), 6.75-6.72 (m, 1H), 4.52 (q, J = 7.2 Hz, 2H), 1.53 (t, J = 7.1 Hz, 3H); ^{13}C {^{1}H} NMR (125 MHz, $CDCl_3$) δ 164.1, 150.3, 147.0, 146.8, 140.7, 135.8, 132.1, 132.1, 130.9, 129.4, 126.0, 125.8, 124.9, 124.2, 122.1, 121.8, 115.5, 115.4, 114.5, 109.4, 107.2, 61.3, 14.6; HRMS (ESI-TOF) m/z: $[M+H]^+$ calculated for $C_{26}H_{18}F_3N_2O_6$: 511.1117; found $[M+H]^+$: 511.1116.

Ethyl 1-([1,1'-biphenyl]-4-yl)-2-(4-nitrophenyl)furo[3,2-*e*]indolizine-4-carboxylate (4j)

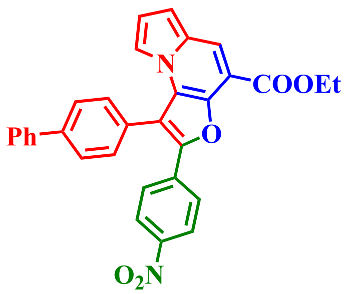

Orange solid, 75%, ^{1}H NMR (500 MHz, $CDCl_3$) δ 8.18 (s, 1H), 8.14 (d, J = 9.1 Hz, 2H), 7.87 (d, J = 8.2 Hz, 2H), 7.76 (dd, J = 8.0, 4.9 Hz, 4H), 7.62 (d, J = 8.2 Hz, 2H), 7.54 (t, J = 7.6 Hz, 2H), 7.45 (t, J = 7.4 Hz, 1H), 7.02 (d, J = 2.1 Hz, 1H), 6.85 (d, J = 4.2 Hz, 1H), 6.72 (dd, J = 4.0, 2.7 Hz, 1H), 4.53 (q, J = 7.1 Hz, 2H), 1.55 (d, J = 7.1 Hz, 3H); ^{13}C {^{1}H} NMR (125 MHz, $CDCl_3$) δ 164.2, 146.9, 146.6, 142.4, 140.6, 139.9, 136.2, 130.9, 130.8, 129.6, 129.2, 128.5, 128.2, 127.2, 125.8, 125.2, 124.1, 121.7, 116.8, 115.2, 114.8, 109.4, 107.0, 61.3, 14.6; HRMS (ESI-TOF) m/z: $[M+H]^+$ calculated for $C_{31}H_{23}N_2O_5$: 503.1607; found $[M+H]^+$: 503.1607.

Ethyl 1-(naphthalen-1-yl)-2-(4-nitrophenyl)furo[3,2-*e*]indolizine-4-carboxylate (4k)

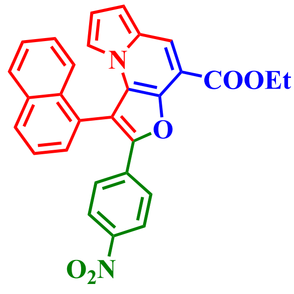

Orange solid, 76%, ^{1}H NMR (500 MHz, $CDCl_3$) δ 8.18 (s, 1H), 8.12 (d, J = 8.8 Hz, 1H), 8.09-8.01 (m, 4H), 7.91 (d, J = 7.9 Hz, 1H), 7.72-7.60 (m, 5H), 6.89-6.85 (m, 1H), 6.83 (d, J = 3.2

Hz, 1H), 6.65-6.60 (m, 1H), 4.54 (q, J = 7.2 Hz, 2H), 1.55 (t, J = 7.1 Hz, 3H); ^{13}C {^{1}H} NMR (125 MHz, $CDCl_3$) δ 164.2, 147.0, 146.6, 140.6, 136.2, 133.7, 133.6, 130.9, 129.9, 129.8, 128.4, 128.2, 128.1, 127.5, 127.4, 127.3, 125.8, 125.3, 124.1, 121.7, 117.1, 115.2, 114.9, 109.4, 107.0, 61.3, 14.6; HRMS (ESI-TOF) m/z: $[M+H]^+$ calculated for $C_{29}H_{21}N_2O_5$: 477.1450; found $[M+H]^+$: 477.1452.

Ethyl 2-(4-cyanophenyl)-1-phenylfuro[3,2-*e*]indolizine-4-carboxylate (4l)

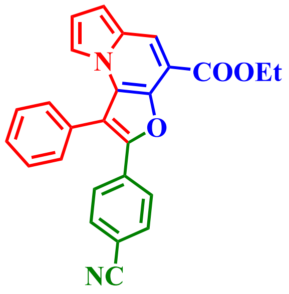

Orange solid, 78%, ^{1}H NMR (500 MHz, $CDCl_3$) δ 8.14 (s, 1H), 7.64-7.60 (m, 5H), 7.53-7.51 (m, 4H), 6.88 (d, J = 2.2 Hz, 1H), 6.82 (d, J = 4.1 Hz, 1H), 6.68 (dd, J = 4.0, 2.7 Hz, 1H), 4.51 (q, J = 7.1 Hz, 2H), 1.52 (t, J = 7.1 Hz, 3H); ^{13}C {^{1}H} NMR (125 MHz, $CDCl_3$) δ 164.3, 147.0, 140.1,134.4, 132.4, 130.9, 130.9, 130.4, 129.9, 129.6, 125.7, 125.1, 121.4, 118.9, 116.5, 115.0, 114.6, 110.8, 109.4, 106.8, 61.2, 14.6; HRMS (ESI-TOF) m/z: $[M+H]^+$ calculated for $C_{26}H_{19}N_2O_3$: 407.1395; found $[M+H]^+$: 407.1390.

Ethyl 2-(4-cyanophenyl)-1-(4-methoxyphenyl)furo[3,2-*e*]indolizine-4-carboxylate (4m)

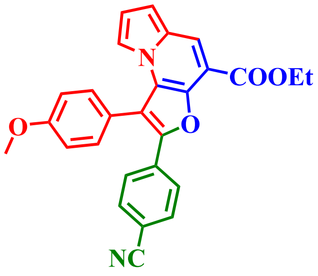

Orange solid, 77%, ^{1}H NMR (500 MHz, $CDCl_3$) δ 8.13 (s, 1H), 7.67 (d, J = 8.5 Hz, 2H), 7.54 (d, J = 8.5 Hz, 2H), 7.43 (d, J = 8.6 Hz, 2H), 7.13 (d, J = 8.6 Hz, 2H), 6.96 (d, J = 1.9 Hz, 1H), 6.82 (d, J = 3.9 Hz, 1H), 6.70 (dd, J = 4.0, 2.7 Hz, 1H), 4.51 (q, J = 7.1 Hz, 2H), 3.95 (s, 3H), 1.52 (t, J = 7.1 Hz, 3H); ^{13}C {^{1}H} NMR (125 MHz, $CDCl_3$) δ 164.3, 160.5, 147.2 , 140.1, 134.5, 132.4, 131.6, 130.9, 125.6, 125.4, 122.5, 121.3, 118.9, 116.3, 115.3, 115.0, 114.6, 110.7, 109.4, 106.7, 61.2, 55.5, 14.6; HRMS (ESI-TOF) m/z: $[M+H]^+$ calculated for $C_{27}H_{21}N_2O_4$: 437.1501; found $[M+H]^+$: 437.1509.

Ethyl 2-(4-cyanophenyl)-1-(p-tolyl)furo[3,2-*e*]indolizine-4-carboxylate (4n)

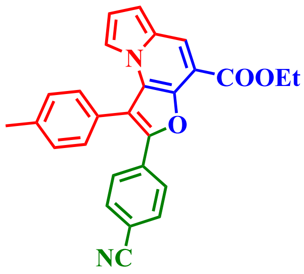

Orange solid, 73%, ^1H NMR (500 MHz, $CDCl_3$) δ 8.13 (s, 1H), 7.66 (d, J = 8.6 Hz, 2H), 7.53 (d, J = 8.6 Hz, 2H), 7.40 (s, 4H), 6.93 (d, J = 2.1 Hz, 1H), 6.82 (d, J = 4.0 Hz, 1H), 6.69 (dd, J = 4.1, 2.7 Hz, 1H), 4.51 (q, J = 7.1 Hz, 2H), 2.53 (s, 3H), 1.52 (t, J = 7.1 Hz, 3H); ^{13}C {^1H} NMR (125 MHz, $CDCl_3$) δ 164.3, 147.0, 140.1, 139.5, 134.5, 132.4, 130.9, 130.6, 130.2, 127.7, 125.6, 125.3, 121.3, 118.9, 116.6, 114.9, 114.7, 110.7, 109.4, 106.7, 61.2, 21.6, 14.6; HRMS (ESI-TOF) m/z: $[M+H]^+$ calculated for $C_{27}H_{21}N_2O_3$: 421.1552; found $[M+H]^+$: 421.1555.

Ethyl 2-(4-cyanophenyl)-1-(4-fluorophenyl)furo[3,2-*e*]indolizine-4-carboxylate (4o)

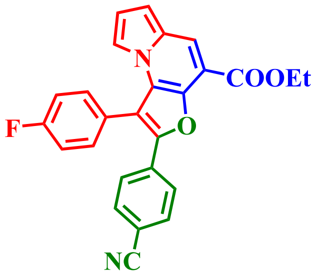

Orange solid, 62%, ^1H NMR (500 MHz, $CDCl_3$) δ 8.15 (s, 1H), 7.63 (d, J = 8.5 Hz, 2H), 7.56-7.51 (m, 4H), 7.32 (t, J = 8.5 Hz, 2H), 6.89 (d, J = 2.2 Hz, 1H), 6.84 (d, J = 4.1 Hz, 1H), 6.72 (dd, J = 4.1, 2.7 Hz, 1H), 4.51 (q, J = 7.1 Hz, 2H), 1.52 (t, J = 7.1 Hz, 3H); ^{13}C {^1H} NMR (125 MHz, $CDCl_3$) δ 164.2, 163.5 (d, $J_{C\text{-}F}$ = 250.2 Hz), 147.2, 140.2, 134.2, 132.5, 132.3 (d, $J_{C\text{-}F}$ = 8.1 Hz), 130.9, 126.8 (d, $J_{C\text{-}F}$ = 3.5 Hz), 125.7, 125.1, 121.5, 118.8, 117.2 (d, $J_{C\text{-}F}$= 21.6 Hz), 115.4, 115.2, 114.4, 111.1, 109.4, 106.9, 61.3, 14.6; HRMS (ESI-TOF) m/z: $[M+H]^+$ calculated for $C_{26}H_{18}FN_2O_3$: 425.1301; found $[M+H]^+$: 425.1350.

Ethyl 1-(4-chlorophenyl)-2-(4-cyanophenyl)furo[3,2-*e*]indolizine-4-carboxylate (4p)

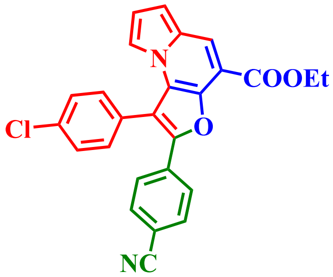

Orange solid, 74%, ^1H NMR (500 MHz, $CDCl_3$) δ 8.15 (s, 1H), 7.63 (d, J = 8.6 Hz, 2H), 7.61 (d, J = 8.4 Hz, 2H), 7.56 (d, J = 8.3 Hz, 2H), 7.49 (d, J = 8.2 Hz, 2H), 6.92 – 6.90 (m, 1H), 6.84 (d, J = 4.1 Hz, 1H), 6.72 (dd, J = 4.1, 2.7 Hz, 1H), 4.51 (q, J = 7.1 Hz, 3H), 1.53 (d, J = 7.1 Hz, 3H);

$^{13}C\ \{^1H\}$ NMR (125 MHz, $CDCl_3$) δ 164.2, 147.2, 140.3, 135.9, 134.1, 132.5, 131.8, 130.9, 130.3, 129.4, 125.7, 124.9, 121.5, 118.8, 115.2, 115.2, 114.5, 111.2, 109.4, 106.9, 61.3, 14.6; HRMS (ESI-TOF) m/z: $[M+H]^+$ calculated for $C_{26}H_{18}ClN_2O_3$: 441.1006; found $[M+H]^+$: 441.1009.

Ethyl 1-(4-bromophenyl)-2-(4-cyanophenyl)furo[3,2-*e*]indolizine-4-carboxylate (4q)

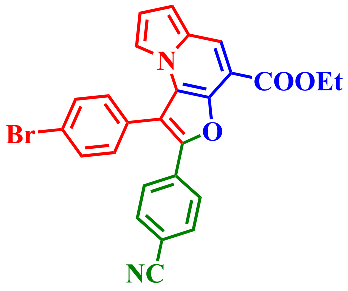

Orange solid, 68%, ^1H NMR (500 MHz, $CDCl_3$) δ 8.15 (s, 1H), 7.76 (d, J = 8.3 Hz, 2H), 7.63 (d, J = 8.6 Hz, 2H), 7.56 (d, J = 8.6 Hz, 2H), 7.42 (d, J = 8.3 Hz, 2H), 6.91 (d, J = 2.2 Hz, 1H), 6.84 (d, J = 3.9 Hz, 1H), 6.73 (dd, J = 4.0, 2.7 Hz, 1H), 4.51 (q, J = 7.1 Hz, 2H), 1.52 (t, J = 7.1 Hz, 3H); $^{13}C\ \{^1H\}$ NMR (125 MHz, $CDCl_3$) δ 164.1, 147.1, 140.3, 134.1, 133.2, 132.5, 132.1, 130.9, 129.9, 125.8, 124.8, 124.0, 121.5, 118.7, 115.2, 115.2, 114.5, 111.2, 109.4, 106.9, 77.3, 61.3, 14.6; HRMS (ESI-TOF) m/z: $[M+H]^+$ calculated for $C_{26}H_{18}BrN_2O_3$: 485.0501; found $[M+H]^+$: 485.0514.

Ethyl 2-(4-cyanophenyl)-1-(3-methoxyphenyl)furo[3,2-*e*]indolizine-4-carboxylate (4r)

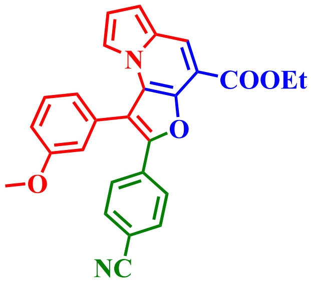

Orange solid, 76%, ^1H NMR (500 MHz, $CDCl_3$) δ 8.14 (s, 1H), 7.67 (d, J = 8.9 Hz, 2H), 7.54 (d, J = 8.8 Hz, 2H), 7.52 (d, J = 8.4 Hz, 1H), 7.15 (dd, J = 8.4, 2.6 Hz, 1H), 7.11 (d, J = 7.5 Hz, 1H), 7.05 – 7.03 (m, 1H), 6.94 (d, J = 1.9 Hz, 1H), 6.83 (d, J = 4.2 Hz, 1H), 6.70 (dd, J = 4.1, 2.6 Hz, 1H), 4.51 (q, J = 7.1, 7.1, 7.1 Hz, 2H), 3.86 (s, 3H), 1.52 (t, J = 7.1, 7.1 Hz, 3H); $^{13}C\ \{^1H\}$ NMR (125 MHz, $CDCl_3$) δ 164.3, 160.6, 146.9, 140.1, 134.4, 132.4, 132.2, 131.1, 130.9, 125.7, 125.1, 122.5, 121.3, 118.9, 116.4, 115.60, 115.5, 115.1, 114.7, 110.9, 109.4, 106.8, 61.2, 55.5, 14.6; HRMS (ESI-TOF) m/z: $[M+H]^+$ calculated for $C_{27}H_{21}N_2O_4$: 437.1501; found $[M+H]^+$: 37.1501.

Ethyl 2-(4-cyanophenyl)-1-(4-(trifluoromethyl)phenyl)furo[3,2-*e*]indolizine-4-carboxylate (4s)

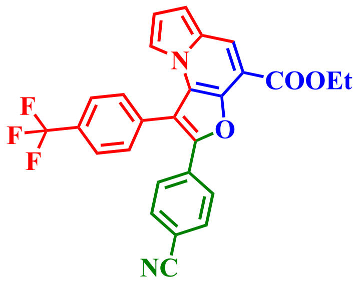

Orange solid, 69%,^{1}H NMR (500 MHz, $CDCl_3$) δ 8.17 (s, 1H), 7.89 (d, J = 8.1 Hz, 2H), 7.70 (d, J = 8.0 Hz, 2H), 7.61 – 7.55 (m, 5H), 6.85 (d, J = 4.1 Hz, 1H), 6.82 (d, J = 2.5 Hz, 1H), 6.73 (dd, J = 2.7, 4.1 Hz, 1H), 4.52 (q, J = 7.1 Hz, 2H), 1.52 (t, J = 7.1 Hz, 3H); ^{13}C {^{1}H} NMR (125 MHz, $CDCl_3$) δ 164.1, 147.3, 140.5, 139.7, 138.5, 135.1, 133.9, 132.6 (d, J = 6.2 Hz), 131.1, 131.0, 126.9 – 126.7 (m), 125.9 (d, J = 5.5 Hz), 124.7, 122.6, 121.6, 118.6, 115.4, 114.9, 114.1, 111.4, 109.5, 107.2, 101.5, 61.3, 14.6; HRMS (ESI-TOF) m/z: $[M+H]^+$ calculated for $C_{27}H_{18}F_3N_2O_3$: 475.1269; found $[M+H]^+$: 475.1275.

Ethyl 2-(4-cyanophenyl)-1-(4-(trifluoromethoxy)phenyl)furo[3,2-*e*]indolizine-4-carboxylate (4t)

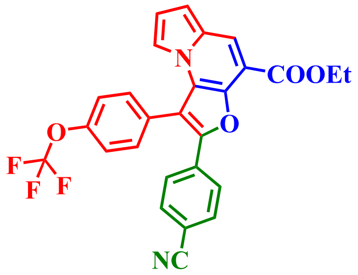

Orange solid, 70%, ^{1}H NMR (500 MHz, $CDCl_3$) δ 8.16 (s, 1H), 7.61 (d, J = 8.8 Hz, 3H), 7.58 (d, J = 5.7 Hz, 2H), 7.55 (s, 1H), 7.47 (d, J = 8.0 Hz, 2H), 6.87 (d, J = 2.1 Hz, 1H), 6.85 (d, J = 4.0 Hz, 1H), 6.73 (dd, J = 2.7, 4.1 Hz, 1H), 4.51 (q, J = 7.1 Hz, 2H), 1.52 (t, J = 7.1 Hz, 3H); ^{13}C {^{1}H} NMR (125 MHz, $CDCl_3$) δ 164.1, 155.8, 147.3, 142.4, 140.3, 139.7, 134.1, 132.5, 132.2, 130.9, 129.5, 125.8, 125.1 – 124.8 (m), 122.1, 121.6, 119.0 – 118.6 (m), 115.3, 114.9, 114.4, 111.2, 109.4, 107.0, 104.7, 61.3, 14.6; HRMS (ESI-TOF) m/z: $[M+H]^+$ calculated for $C_{27}H_{18}F_3N_2O_4$: 491.1218; found $[M+H]^+$: 491.1220.

Ethyl 1-([1,1'-biphenyl]-4-yl)-2-(4-cyanophenyl)furo[3,2-*e*]indolizine-4-carboxylate (4u)

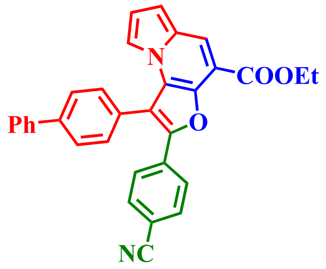

Orange solid, 73%, ^{1}H NMR (500 MHz, $CDCl_3$) δ 8.16 (s, 1H), 7.86 (d, J = 8.2 Hz, 2H), 7.76 (d, J = 7.3 Hz, 2H), 7.70 (d, J = 8.5 Hz, 2H), 7.61 (d, J = 8.1 Hz, 2H), 7.54 (dd, J = 12.3, 8.1 Hz, 4H), 7.44 (t, J = 7.4 Hz, 1H), 7.02 -7.00 (m, 1H), 6.84 (d, J = 4.0 Hz, 1H), 6.71 (dd, J = 4.0, 2.7 Hz, 1H), 4.52 (q, J = 7.1 Hz, 2H), 1.53 (t, J = 7.1 Hz, 3H); ^{13}C {^{1}H} NMR (125 MHz, $CDCl_3$) δ 164.3, 147.1, 142.2, 140.2, 139.9, 134.4, 132.5, 130.9, 130.9, 129.7, 129.2, 128.4, 128.2, 127.2, 125.7, 125.2, 121.4, 118.9, 116.2, 115.1, 114.7, 110.9, 109.4, 106.8, 61.2, 14.6; HRMS (ESI-TOF) m/z: $[M+H]^+$ calculated for $C_{32}H_{23}N_2O_3$: 483.1708; found $[M+H]^+$: 483.1714.

Ethyl 2-(4-cyanophenyl)-1-(naphthalen-1-yl)furo[3,2-*e*]indolizine-4-carboxylate (4v)

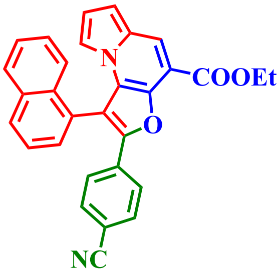

Orange solid, 69%, ^{1}H NMR (500 MHz, $CDCl_3$) δ 8.17 (s, 1H), 8.11 (s, 1H), 8.06-8.02 (m, 2H), 7.91 (d, J = 8.0 Hz, 1H), 7.65 (d, J = 8.7 Hz, 3H), 7.63-7.58 (m, 2H), 7.49 (d, J = 8.6 Hz, 2H), 6.85 (d, J = 2.3 Hz, 1H), 6.82 (d, J = 4.0 Hz, 1H), 6.62 (dd, J = 4.0, 2.7 Hz, 1H), 4.53 (q, J = 7.1 Hz, 2H), 1.54 (t, J = 7.1 Hz, 3H); ^{13}C {^{1}H} NMR (125 MHz, $CDCl_3$) δ 164.3, 147.2, 140.3, 135.4, 134.4, 133.7, 133.5, 132.5, 130.9, 129.9, 129.8, 128.4, 128.2, 128.2, 127.5, 127.4, 127.2, 125.7, 125.3, 121.4, 116.5, 115.1, 114.7, 110.9, 109.4, 106.8, 61.3, 14.6; HRMS (ESI-TOF) m/z: $[M+H]^+$ calculated for $C_{30}H_{21}N_2O_3$: 457.1552; found $[M+H]^+$: 457.1557.

Ethyl 2-(4-chlorobenzoyl)-1-phenylfuro[3,2-*e*]indolizine-4-carboxylate (6a)

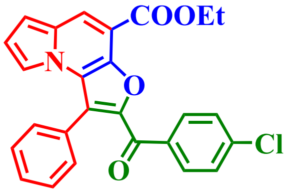

Yellow solid, 63%, ^{1}H NMR (500 MHz, $CDCl_3$) δ 8.31 (s, 1H), 8.23 (d, J = 8.2 Hz, 2H), 7.55 (s, 5H), 7.47 (d, J = 8.3 Hz, 2H), 7.08 (s, 1H), 6.90 (d, J = 3.4 Hz, 1H), 6.77 – 6.65 (m, 1H), 4.50 (q, J = 7.2, 7.2, 7.1 Hz, 2H), 1.45 (t, J = 7.2, 7.2 Hz, 3H); ^{13}C {^{1}H} NMR (125 MHz, $CDCl_3$) δ 180.8, 163.9, 146.5, 141.7, 139.3, 135.4, 131.7, 130.7, 130.2, 129.7, 129.2, 128.9, 128.7, 125.5, 124.9, 124.2, 116.3, 115.4, 109.2, 108.5, 61.5, 14.7; HRMS (ESI-TOF) m/z: $[M+H]^+$ calculated for $C_{26}H_{19}ClNO_4$: 444.1002; found $[M+H]^+$: 444.0952.

Ethyl 2-(4-bromobenzoyl)-1-phenylfuro[3,2-*e*]indolizine-4-carboxylate (6b)

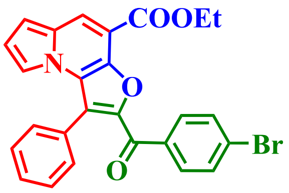

Yellow solid, 65%, ^{1}H NMR (500 MHz, $CDCl_3$) δ 8.31 (s, 1H), 8.15 (d, J = 7.1 Hz, 2H), 7.64 (d, J = 6.8 Hz, 2H), 7.55 (s, 5H), 7.08 (s, 1H), 6.90 (d, J = 3.9 Hz, 1H), 6.72 (s, 1H), 4.50 (q, J = 7.3, 7.3, 7.0 Hz, 2H), 1.50 – 1.43 (m, 3H); ^{13}C {^{1}H} NMR (125 MHz, $CDCl_3$) δ 181.8, 155.2, 149.1, 147.1, 140.2, 139.6, 135.8, 130.9, 130.8, 129.8, 129.1, 128.9, 128.3, 127.4, 127.0, 125.2, 124.7, 116.2, 115.3, 109.3, 108.3, 61.5, 14.6; HRMS (ESI-TOF) m/z: $[M+H]^+$ calculated for $C_{26}H_{19}BrNO_4$: 488.0497; found $[M+H]^+$: 488.0502.

Ethyl 2-(4-methoxybenzoyl)-1-phenylfuro[3,2-*e*]indolizine-4-carboxylate (6c)

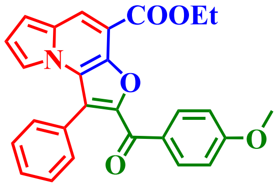

Yellow solid, 83%, ^{1}H NMR (500 MHz, $CDCl_3$) δ 8.33 (d, J = 8.9 Hz, 2H), 8.30 (s, 1H), 7.54 (s, 5H), 7.06 (s, 1H), 6.98 (d, J = 8.8 Hz, 2H), 6.89 (d, J = 3.7 Hz, 1H), 6.74 – 6.69 (m, 1H), 4.51 (q, J = 7.1, 7.1, 7.1 Hz, 2H), 3.90 (s, 3H), 1.47 (t, J = 7.1, 7.1 Hz, 3H); ^{13}C {^{1}H} NMR (125 MHz, $CDCl_3$) δ 180.7, 164.1, 163.5, 147.2, 141.1, 132.7, 130.7, 130.6, 129.8, 128.9, 128.9, 124.7, 124.3, 124.2, 116.1, 115.2, 113.7, 109.3, 108.0, 61.4, 55.6, 14.7; HRMS (ESI-TOF) m/z: $[M+H]^+$ calculated for $C_{27}H_{22}NO_4$: 440.1498; found $[M+H]^+$: 440.1439.

Ethyl 2-(3-methoxybenzoyl)-1-phenylfuro[3,2-*e*]indolizine-4-carboxylate (6d)

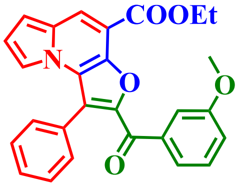

Yellow solid, 78%, ^{1}H NMR (500 MHz, $CDCl_3$) δ 8.31 (s, 1H), 7.93 (d, J = 7.4 Hz, 1H), 7.73 (s, 1H), 7.54 (s, 5H), 7.40 (t, J = 7.9 Hz, 1H), 7.13 (d, J = 5.7 Hz, 1H), 7.08 (s, 1H), 6.89 (d, J = 3.5 Hz, 1H), 6.73 – 6.69 (m, 1H), 4.49 (q, J = 7.0 Hz, 2H), 3.86 (s, 3H), 1.43 (t, J = 7.1 Hz, 3H); ^{13}C {^{1}H} NMR (125 MHz, $CDCl_3$) δ 182.00, 164.01, 159.57, 146.80, 141.53, 138.28, 130.76, 130.43, 129.79, 129.31, 129.06, 128.91, 125.24, 124.64, 124.21, 123.02, 119.59, 116.21, 115.24, 114.42, 109.40, 108.24, 61.43, 55.50, 14.50; HRMS (ESI-TOF) m/z: $[M+H]^+$ calculated for $C_{27}H_{22}NO_4$: 440.1498; found $[M+H]^+$: 440.1471.

Ethyl 2-([1,1'-biphenyl]-4-carbonyl)-1-phenylfuro[3,2-*e*]indolizine-4-carboxylate (6e)

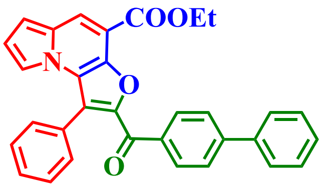

Yellow solid, 70%, ^{1}H NMR (500 MHz, $CDCl_3$) δ 8.42 – 8.28 (m, 3H), 7.68 (dd, J = 29.8, 7.2 Hz, 4H), 7.55 (s, 5H), 7.49 (d, J = 7.1 Hz, 2H), 7.44 – 7.39 (m, 1H), 7.10 (s, 1H), 6.90 (d, J = 4.0 Hz, 1H), 6.77 – 6.69 (m, 1H), 4.51 (q, J = 6.9, 6.9, 6.9 Hz, 2H), 1.46 (t, J = 7.1, 7.1 Hz, 3H); ^{13}C {^{1}H} NMR (125 MHz, $CDCl_3$) δ 181.8, 164.1, 146.9, 145.4, 141.5, 140.2, 135.8, 130.9, 130.8, 130.4, 129.8, 129.1, 128.9, 128.3, 127.4, 127.0, 125.2, 124.7, 124.2, 116.3, 115.3, 109.3, 108.3, 61.5, 14.7; HRMS (ESI-TOF) m/z: $[M+H]^+$ calculated for $C_{32}H_{24}NO_4$: 486.1705; found $[M+H]^+$: 486.1726.

Ethyl 2-(4-nitrobenzoyl)-1-phenylfuro[3,2-*e*]indolizine-4-carboxylate (6f)

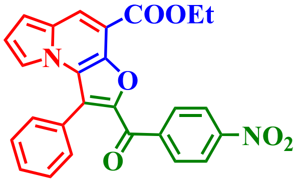

Yellow solid, 52%, ^{1}H NMR (500 MHz, $CDCl_3$) δ 8.39 (d, J = 8.9 Hz, 2H), 8.33 (d, J = 8.8 Hz, 3H), 7.59 – 7.51 (m, 5H), 7.11 (d, J = 1.9 Hz, 1H), 6.93 (d, J = 3.4 Hz, 1H), 6.73 (dd, J = 4.1, 2.7 Hz, 1H), 4.49 (q, J = 7.1, 7.1, 7.1 Hz, 2H), 1.44 (t, J = 7.1, 7.1 Hz, 3H); ^{13}C {^{1}H} NMR (125 MHz, $CDCl_3$) δ 180.2, 163.6, 149.9, 145.9, 142.5, 142.1, 131.2, 130.7, 129.8, 129.7, 129.5, 129.1, 126.4, 125.5, 124.2, 123.9, 123.5, 116.6, 115.6, 109.1, 108.9, 61.5, 14.7; HRMS (ESI-TOF) m/z: $[M+H]^+$ calculated for $C_{26}H_{19}N_2O_6$: 455.1243; found $[M+H]^+$: 455.1244.

Ethyl 2-(3-nitrobenzoyl)-1-phenylfuro[3,2-*e*]indolizine-4-carboxylate (6g)

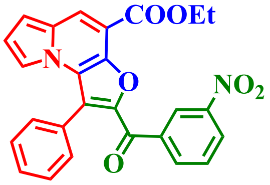

Orange red solid, 50%, ^{1}H NMR (500 MHz, $CDCl_3$) δ 9.01 (s, 1H), 8.57 (d, J = 7.8 Hz, 1H), 8.41 (d, J = 8.0 Hz, 1H), 8.34 (s, 1H), 7.69 (t, J = 8.0 Hz, 1H), 7.57 – 7.51 (m, 5H), 7.11 (s, 1H), 6.92 (dd, J = 4.1, 1.5 Hz, 1H), 6.73 (m, 1H), 4.48 (q, J = 7.1 Hz, 2H), 1.39 (t, J = 7.1 Hz, 3H); ^{13}C {^{1}H} NMR (125 MHz, $CDCl_3$) δ 179.7, 163.7, 148.2, 145.9, 142.5, 138.5, 135.7, 135.6, 130.7, 129.8, 129.7, 129.1, 126.3, 125.5, 125.4, 125.3, 125.2, 124.2, 116.6, 116.4, 115.5, 109.3, 108.9, 108.7, 61.5, 14.5; HRMS (ESI-TOF) m/z: $[M+H]^+$ calculated for $C_{26}H_{19}N_2O_6$: 455.1243; found $[M+H]^+$: 455.1271.

Ethyl 2-(3-bromobenzoyl)-1-phenylfuro[3,2-*e*]indolizine-4-carboxylate (6h)

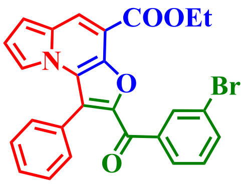

Orange solid, 70%, ^{1}H NMR (500 MHz, $CDCl_3$) δ 8.33 (s, 2H), 8.17 (d, J = 7.7 Hz, 1H), 7.69 (d, J = 7.8 Hz, 1H), 7.54 (d, J = 4.5 Hz, 5H), 7.37 (t, J = 7.9, 7.9 Hz, 1H), 7.09 (s, 1H), 6.91 (d, J = 3.9 Hz, 1H), 6.75 – 6.70 (m, 1H), 4.53 (q, J = 7.1, 7.1, 7.1 Hz, 2H), 1.42 (t, J = 7.1, 7.1 Hz, 3H); ^{13}C {^{1}H} NMR (125 MHz, $CDCl_3$) δ 180.8, 169.6, 163.9, 146.3, 141.9, 138.9, 135.6, 133.1, 130.8, 130.2, 129.9, 129.7, 129.2, 128.9, 128.7, 125.7, 125.1, 124.2, 122.5, 116.4, 115.3, 109.4, 108.4, 61.5, 14.7; HRMS (ESI-TOF) m/z: $[M+H]^+$ calculated for $C_{26}H_{19}BrNO_4$: 488.0497; found $[M+H]^+$: 488.0497.

Ethyl 2-(3,4-dichlorobenzoyl)-1-phenylfuro[3,2-*e*]indolizine-4-carboxylate (6i)

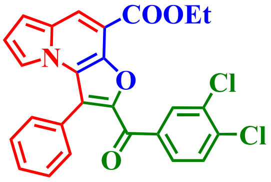

Yellow solid, 69%, ^{1}H NMR (500 MHz, $CDCl_3$) δ 8.37 (d, J = 2.1 Hz, 1H), 8.33 (s, 1H), 8.14 (dd, J = 8.4, 2.1 Hz, 1H), 7.59 (s, 1H), 7.56 (dd, J = 6.2, 3.0 Hz, 4H), 7.52 (dd, J = 6.8, 3.1 Hz, 2H), 7.09 (d, J = 3.2 Hz, 1H), 6.91 (d, J = 4.2 Hz, 1H), 6.72 (dd, J = 4.2, 2.6 Hz, 1H), 4.54 (q, J = 7.2, 7.2, 7.2 Hz, 2H), 1.44 (t, J = 7.1, 7.1 Hz, 4H); ^{13}C {^{1}H} NMR (125 MHz, $CDCl_3$) δ 179.5, 163.8, 146.1, 142.0, 137.4, 136.7, 132.7, 132.2, 130.8, 130.5, 129.9, 129.7, 129.3, 129.0, 125.9, 125.2, 124.2, 116.4, 115.4, 109.2, 108.6, 61.5, 14.7; HRMS (ESI-TOF) m/z: $[M+H]^+$ calculated for $C_{26}H_{18}Cl_2NO_4$: 478.0613; found $[M+H]^+$: 478.0608.

Ethyl 2-(3,5-bis(trifluoromethyl)benzoyl)-1-phenylfuro[3,2-*e*]indolizine-4-carboxylate (6j)

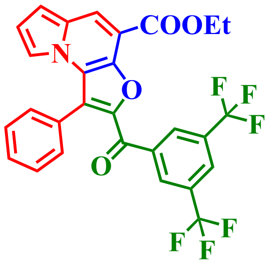

Yellowish red solid, 65%, ^{1}H NMR (500 MHz, $CDCl_3$) δ 8.67 (s, 2H), 8.32 (d, J = 6.2 Hz, 1H), 8.05 (s, 1H), 7.54 (s, 5H), 7.12 (d, J = 6.3 Hz, 1H), 6.92 (t, J = 4.8, 4.8 Hz, 1H), 6.75 – 6.68 (m, 1H), 4.48 (q, J = 7.0, 6.9, 6.9 Hz, 2H), 1.37 (q, J = 7.0, 6.8, 6.8 Hz, 3H); ^{13}C {^{1}H} NMR (125 MHz, $CDCl_3$) δ 179.1, 163.5, 145.7, 142.8, 138.8, 132.0 (q, J_{C-F} = 33.7 Hz), 130.7, 130.2, 130.2, 129.7, 129.6, 129.5, 129.1, 126.6, 125.8, 125.4, 124.2, 122.0, 116.6, 115.5, 109.3, 108.9, 61.4,

14.4; HRMS (ESI-TOF) m/z: $[M+H]^+$ calculated for $C_{28}H_{18}F_6NO_4$: 546.1140; found $[M+H]^+$: 546.1179.

Ethyl 2-(2-fluorobenzoyl)-1-phenylfuro[3,2-*e*]indolizine-4-carboxylate (6k)

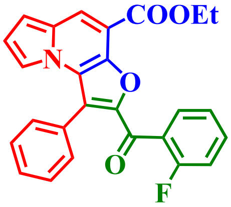

Yellow solid, 78%, ^{1}H NMR (500 MHz, $CDCl_3$) δ 8.28 (s, 1H), 7.58 (t, J = 7.2, 7.2 Hz, 1H), 7.51 – 7.43 (m, 5H), 7.43 – 7.38 (m, 1H), 7.17 (t, J = 7.5, 7.5 Hz, 1H), 7.12 – 7.08 (m, 1H), 6.99 (t, J = 9.1, 9.1 Hz, 1H), 6.87 (d, J = 4.0 Hz, 1H), 6.71 – 6.67 (m, 1H), 4.38 (q, J = 7.1, 7.1, 7.1 Hz, 2H), 1.28 (t, J = 7.1, 7.1 Hz, 3H); ^{13}C {^{1}H} NMR (125 MHz, $CDCl_3$) δ 180.9, 164.1, 160.3 (d, J_{C-F} = 253.3 Hz), 146.5, 145.8, 142.3, 130.7 (d, J_{C-F} = 18.7 Hz), 130.6, 130.0, 129.4, 128.8, 128.6, 127.1, 127.0, 125.1 (d, J_{C-F} = 26.3 Hz). 124.9, 124.6, 124.1, 116.4, 115.8, 115.5, 109.5, 108.1, 104.2, 61.4, 14.3; HRMS (ESI-TOF) m/z: $[M+H]^+$ calculated for $C_{26}H_{19}FNO_2$: 428.1298; found $[M+H]^+$: 428.1299.

Ethyl 2-(2-methoxybenzoyl)-1-phenylfuro[3,2-*e*]indolizine-4-carboxylate (6l)

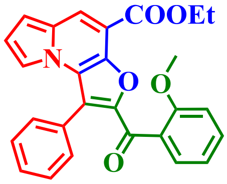

Yellow solid, 81%, ^{1}H NMR (500 MHz, $CDCl_3$) δ 8.26 (s, 1H), 7.38 (s, 6H), 7.33 – 7.29 (m, 1H), 7.07 (s, 1H), 6.92 (t, J = 7.4 Hz, 1H), 6.87 (d, J = 3.4 Hz, 1H), 6.74 – 6.65 (m, 2H), 4.40 (q, J = 6.7 Hz, 2H), 3.68 (s, 3H), 1.31 (t, J = 7.0 Hz, 3H); ^{13}C {^{1}H} NMR (125 MHz, $CDCl_3$) δ 184.1, 164.2, 157.6, 147.4, 141.7, 132.4, 130.8, 130.1, 129.8, 129.7, 128.8, 128.4, 124.4, 124.1, 123.7, 120.4, 115.9, 115.0, 111.0, 109.6, 107.9, 61.4, 55.5, 14.3; HRMS (ESI-TOF) m/z: $[M+H]^+$ calculated for $C_{27}H_{22}NO_5$: 440.1498; found $[M+H]^+$: 440.1499.

Ethyl 2-benzoyl-1-(4-methoxyphenyl)furo[3,2-*e*]indolizine-4-carboxylate (6m)

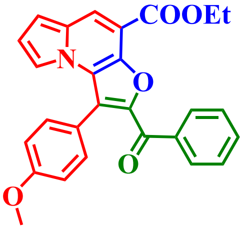

Yellow solid, 80%, ^{1}H NMR (500 MHz, $CDCl_3$) δ 8.30 (s, 1H), 8.23 (d, J = 7.3 Hz, 2H), 7.57 (t, J = 7.3, 7.3 Hz, 1H), 7.48 (dd, J = 12.6, 8.1 Hz, 4H), 7.20 (d, J = 2.9 Hz, 1H), 7.07 (d, J = 8.5

Hz, 2H), 6.90 (d, J = 3.6 Hz, 1H), 6.78 – 6.70 (m, 1H), 4.54 – 4.44 (m, 2H), 3.91 (s, 3H), 1.42 (t, J = 7.1, 7.1 Hz, 3H); ^{13}C {^{1}H} NMR (125 MHz, $CDCl_3$) δ 182.50, 164.17, 160.14, 146.87, 141.43, 137.20, 132.77, 131.19, 130.78, 130.26, 128.31, 125.05, 124.69, 124.37, 122.04, 116.28, 115.21, 114.36, 109.33, 108.19, 61.45, 55.45, 14.61; HRMS (ESI-TOF) m/z: $[M+H]^+$ calculated for $C_{27}H_{22}NO_4$: 440.1498; found $[M+H]^+$: 440.1496.

Ethyl 2-(4-chlorobenzoyl)-1-(4-methoxyphenyl)furo[3,2-*e*]indolizine-4-carboxylate (6n)

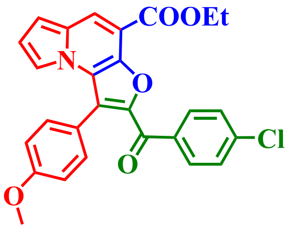

Yellow solid, 76%, ^{1}H NMR (500 MHz, $CDCl_3$) δ 8.31 (s, 1H), 8.22 (d, J = 8.6 Hz, 2H), 7.46 (dd, J = 8.4, 4.3 Hz, 4H), 7.22 – 7.18 (m, 1H), 7.08 (d, J = 8.6 Hz, 2H), 6.91 (d, J = 3.9 Hz, 1H), 6.75 – 6.72 (m, 1H), 4.49 (q, J = 7.1, 7.1, 7.0 Hz, 2H), 3.91 (s, 3H), 1.44 (t, J = 7.1, 7.1 Hz, 3H); ^{13}C {^{1}H} NMR (125 MHz, $CDCl_3$) δ 180.9, 163.9, 160.2, 146.6, 141.6, 139.2, 135.5, 131.8, 131.6, 131.2, 131.1, 130.7, 128.7, 128.6, 125.4, 125.0, 124.4, 121.8, 115.3, 114.6, 114.5, 114.3, 114.2, 109.2, 61.5, 55.5, 14.6; HRMS (ESI-TOF) m/z: $[M+H]^+$ calculated for $C_{27}H_{21}ClNO_5$: 474.1108; found $[M+H]^+$: 474.0981.

Ethyl 2-(3-methoxybenzoyl)-1-(4-methoxyphenyl)furo[3,2-*e*]indolizine-4-carboxylate (6o)

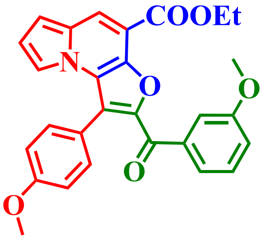

Yellow solid, 77%, ^{1}H NMR (500 MHz, $CDCl_3$) δ 8.29 (s, 1H), 7.93 (d, J = 7.9 Hz, 1H), 7.74 (s, 1H), 7.47 (d, J = 8.6 Hz, 2H), 7.40 (t, J = 8.0, 8.0 Hz, 1H), 7.19 (d, J = 2.9 Hz, 1H), 7.12 (d, J = 5.8 Hz, 1H), 7.07 (d, J = 8.6 Hz, 2H), 6.88 (d, J = 3.6 Hz, 1H), 6.74 – 6.70 (m, 1H), 4.49 (q, J = 7.9, 7.9, 7.1 Hz, 2H), 3.91 (s, 3H), 3.86 (s, 3H), 1.42 (t, J = 7.1, 7.1 Hz, 3H); ^{13}C {^{1}H} NMR ((125 MHz, $CDCl_3$) δ 181.9, 164.0, 160.1, 159.5, 146.9, 138.4, 131.2, 130.8, 129.3, 125.2, 124.6, 124.4, 123.0, 122.1, 119.5, 116.3, 115.2, 114.4, 114.4, 109.3, 108.2, 61.4, 55.5, 55.4, 14.5; HRMS (ESI-TOF) m/z: $[M+H]^+$ calculated for $C_{28}H_{24}NO_6$: 470.1604; found: 470.1609.

Ethyl 2-([1,1'-biphenyl]-4-carbonyl)-1-(4-methoxyphenyl)furo[3,2-*e*]indolizine-4-Carboxylate (6p)

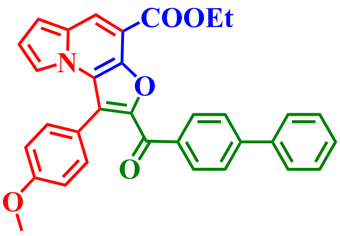

Yellow solid, 78%, ^{1}H NMR (500 MHz, $CDCl_3$) δ 8.35 (d, J = 8.4 Hz, 2H), 8.32 (s, 1H), 7.72 (d, J = 8.4 Hz, 2H), 7.66 (d, J = 7.4 Hz, 2H), 7.56 (s, 5H), 7.49 (t, J = 7.7, 7.7 Hz, 2H), 7.41 (t, J = 7.4, 7.4 Hz, 1H), 7.10 (d, J = 3.0 Hz, 1H), 6.90 (d, J = 4.3 Hz, 1H), 6.75 – 6.70 (m, 1H), 4.51 (q, J = 7.2, 7.1, 7.1 Hz, 2H), 1.47 (t, J = 7.2, 7.2 Hz, 3H); ^{13}C {^{1}H} NMR (125 MHz, $CDCl_3$) δ 181.77, 164.06, 146.98, 145.44, 141.50, 140.18, 135.86, 131.00, 130.78, 130.75, 130.42, 129.85, 129.06, 128.99, 128.26, 127.44, 127.38, 127.06, 127.01, 126.96, 125.13, 124.78, 124.52, 124.23, 116.06, 115.25, 109.37, 108.33, 108.13, 61.44, 14.58; HRMS (ESI-TOF) m/z: $[M+H]^+$ calculated for $C_{33}H_{26}NO_5$: 516.1811; found: 516.1820.

Ethyl 1-(4-methoxyphenyl)-2-(3-nitrobenzoyl)furo[3,2-*e*]indolizine-4-carboxylate (6q)

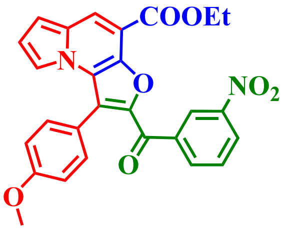

Orange solid, 68%, ^{1}H NMR (500 MHz, $CDCl_3$) δ 9.00 (s, 1H), 8.56 (d, J = 7.6 Hz, 1H), 8.40 (s, 1H), 8.33 (s, 1H), 7.69 (t, J = 7.9, 7.9 Hz, 1H), 7.46 (d, J = 7.5 Hz, 2H), 7.23 (s, 2H), 7.06 (d, J = 7.8 Hz, 2H), 6.92 (d, J = 3.6 Hz, 1H), 6.75 (s, 1H), 4.47 (q, J = 7.0, 6.5, 6.5 Hz, 2H), 3.91 (s, 3H), 1.38 (t, J = 7.0, 7.0 Hz, 3H); ^{13}C {^{1}H} NMR (125 MHz, $CDCl_3$) δ 179.8, 163.8, 160.4, 148.2, 146.0, 142.4, 138.6, 135.7, 131.2, 130.8, 129.5, 126.8, 126.2, 125.4, 125.2, 124.4, 121.4, 116.6, 115.5, 114.5, 109.2, 108.8, 61.5, 55.5, 14.5; HRMS (ESI-TOF) m/z: $[M+H]^+$ calculated for $C_{27}H_{21}N_2O_7$: 485.1349; found $[M+H]^+$: 485.1369.

Ethyl 2-(3-bromobenzoyl)-1-(4-methoxyphenyl)furo[3,2-*e*]indolizine-4-carboxylate (6r)

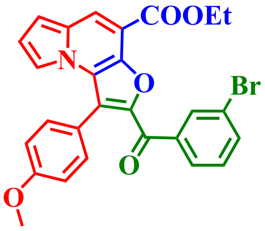

Yellow solid, 72%, ^{1}H NMR (500 MHz, $CDCl_3$) δ 8.31 (s, 2H), 8.16 (d, J = 7.3 Hz, 1H), 7.68 (d, J = 8.4 Hz, 1H), 7.45 (d, J = 7.0 Hz, 2H), 7.40 – 7.33 (m, 1H), 7.21 (s, 1H), 7.07 (d, J = 6.9 Hz, 2H), 6.90 (s, 1H), 6.73 (s, 1H), 4.53 (d, J = 6.8 Hz, 2H), 3.91 (s, 3H), 1.42 (t, J = 6.4, 6.4 Hz,

3H); ^{13}C {^{1}H} NMR (125 MHz, $CDCl_3$) δ 180.8, 164.0, 160.3, 146.4, 141.9, 139.1, 135.5, 133.1, 131.2, 130.8, 129.9, 128.7, 125.6, 125.0, 124.3, 122.4, 121.7, 116.4, 115.3, 114.4, 109.4, 108.4, 61.5, 55.5, 14.6; HRMS (ESI-TOF) m/z: $[M+H]^+$ calculated for $C_{27}H_{20}BrNO_5$: 518.0603; found $[M+H]^+$: 518.0606.

Ethyl 2-(3,4-dichlorobenzoyl)-1-(4-methoxyphenyl)furo[3,2-*e*]indolizine-4-carboxylate (6s)

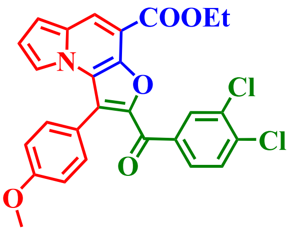

Yellow solid, 71%, ^{1}H NMR (500 MHz, $CDCl_3$) δ 8.35 (d, J = 1.9 Hz, 1H), 8.31 (s, 1H), 8.12 (dd, J = 8.4, 1.9 Hz, 1H), 7.58 (d, J = 8.4 Hz, 1H), 7.45 (d, J = 8.6 Hz, 2H), 7.22 – 7.20 (m, 1H), 7.08 (d, J = 8.6 Hz, 2H), 6.91 (s, 1H), 6.73 (dd, J = 3.9, 2.8 Hz, 1H), 4.53 (q, J = 7.2, 7.2, 7.1 Hz, 2H), 3.92 (s, 3H), 1.44 (t, J = 7.1, 7.1 Hz, 3H); ^{13}C {^{1}H} NMR (125 MHz, $CDCl_3$) δ 179.6, 163.9, 160.4, 146.2, 141.9, 137.2, 136.8, 132.8, 132.2, 131.1, 130.8, 130.5, 129.3, 125.9, 125.2, 124.4, 121.6, 116.5, 115.4, 114.5, 109.2, 108.6, 61.5, 55.5, 14.6; HRMS (ESI-TOF) m/z: $[M+H]^+$ calculated for $C_{27}H_{20}Cl_2NO_5$: 508.0718; found $[M+H]^+$: 508.0718.

Ethyl 2-(3,5-bis(trifluoromethyl)benzoyl)-1-(4-methoxyphenyl)furo[3,2-*e*]indolizine-4-carboxylate (6t)

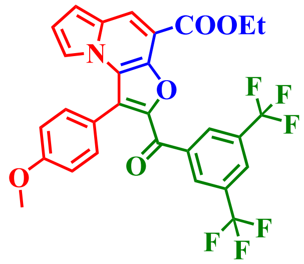

Yellow solid, 63%, ^{1}H NMR (500 MHz, $CDCl_3$) δ 8.65 (s, 2H), 8.33 (s, 1H), 8.05 (s, 1H), 7.45 (d, J = 8.7 Hz, 2H), 7.25 (d, J = 2.3 Hz, 1H), 7.07 (d, J = 8.7 Hz, 2H), 6.93 (d, J = 4.1 Hz, 1H), 6.75 (dd, J = 4.1, 2.7 Hz, 1H), 4.47 (q, J = 7.1, 7.1, 7.1 Hz, 2H), 3.91 (s, 3H), 1.37 (t, J = 7.1, 7.1 Hz, 3H); ^{13}C {^{1}H} NMR (125 MHz, $CDCl_3$) δ 179.2, 163.5, 160.5, 145.8, 142.8, 138.9, 131.9 (q, J_{C-F} = 34.0 Hz), 131.1, 130.7, 130.2, 130.2, 126.6, 125.7, 125.4, 124.4, 124.2, 122.0, 121.2, 116.6, 115.5, 114.5, 109.3, 108.8, 61.4, 55.4, 14.4; HRMS (ESI-TOF) m/z: $[M+H]^+$ calculated for $C_{29}H_{20}F_6NO_5$: 576.1245; found $[M+H]^+$: 576.1245.

Ethyl 2-(2-fluorobenzoyl)-1-(4-methoxyphenyl)furo[3,2-*e*]indolizine-4-carboxylate (6u)

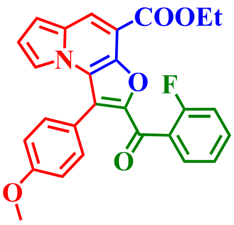

Yellow solid, 76%, ^{1}H NMR (500 MHz, $CDCl_3$) δ 8.29 (s, 1H), 7.59 (t, J = 7.2 Hz, 1H), 7.42 (d, J = 8.6 Hz, 3H), 7.23 (s, 1H), 7.19 (t, J = 7.5 Hz, 1H), 7.06 – 6.97 (m, 3H), 6.89 (d, J = 4.0 Hz, 1H), 6.75 – 6.70 (m, 1H), 4.39 (q, J = 7.1 Hz, 2H), 3.88 (s, 3H), 1.28 (t, J = 7.1 Hz, 3H); ^{13}C {^{1}H} NMR (125 MHz, $CDCl_3$) δ 181.0, 164.1, 160.3 (d, $J_{C\text{-}F}$ = 252.2 Hz), 160.2, 146.6, 142.3, 133.2, 132.4, 131.4 (d, $J_{C\text{-}F}$ = 5.9 Hz), 130.8, 130.6, 127.2 (d, $J_{C\text{-}F}$ = 14.5 Hz), 125.1 (d, $J_{C\text{-}F}$ = 7.0 Hz), 124.6, 124.3, 124.1, 121.1, 115.2, 114.2 (d, $J_{C\text{-}F}$ = 7.1 Hz), 109.4, 108.3, 61.4, 55.5, 14.3; HRMS (ESI-TOF) m/z: $[M+H]^+$ calculated for $C_{27}H_{21}FNO_5$: 458.1404; found $[M+H]^+$: 458.1405.

Ethyl 2-(2-methoxybenzoyl)-1-(4-methoxyphenyl)furo[3,2-*e*]indolizine-4-carboxylate (6v)

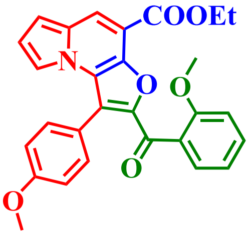

Yellow solid, 80%, ^{1}H NMR (500 MHz, $CDCl_3$) δ 8.25 (s, 1H), 7.39 (dd, J = 7.5, 1.7 Hz, 1H), 7.35 – 7.30 (m, 3H), 7.18 (d, J = 2.3 Hz, 1H), 6.94 (d, J = 7.5 Hz, 1H), 6.91 (d, J = 8.7 Hz, 2H), 6.86 (d, J = 4.1 Hz, 1H), 6.75 (d, J = 8.4 Hz, 1H), 6.70 (dd, J = 4.1, 2.7 Hz, 1H), 4.39 (q, J = 7.1 Hz, 2H), 3.86 (s, 3H), 3.68 (s, 3H), 1.30 (t, J = 7.1 Hz, 3H); ^{13}C {^{1}H} NMR (125 MHz, $CDCl_3$) δ 184.2, 164.2, 159.9, 157.6, 147.4, 141.7, 132.2, 131.5, 131.3 (d, J = 13.5Hz), 130.9, 129.7, 128.8, 124.5, 124.2, 123.6, 121.5, 120.4 (d, J = 17.4 Hz), 114.9, 113.8 (d, J = 16.7Hz), 111.1, 111.0, 109.5, 107.8 (d, J = 13.3 Hz), 61.3, 55.4, 14.3; HRMS (ESI-TOF) m/z: $[M+H]^+$ calculated for $C_{28}H_{23}NO_6$: 470.1603; found $[M+H]^+$: 470.1603.

Ethyl 2-(3-chlorobenzoyl)-1-(3-methoxyphenyl)furo[3,2-*e*]indolizine-4-carboxylate (6w)

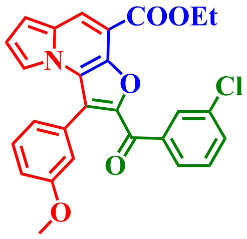

Yellow solid, 75%, ^{1}H NMR (500 MHz, $CDCl_3$) δ 8.32 (s, 1H), 8.18 (t, J = 1.7, 1.7 Hz, 1H), 8.10 (d, J = 7.8 Hz, 1H), 7.56 – 7.52 (m, 1H), 7.45 (dd, J = 16.4, 8.1 Hz, 2H), 7.14 (d, J = 2.3 Hz,

1H), 7.10 – 7.05 (m, 2H), 7.04 – 7.02 (m, 1H), 6.91 (d, *J* = 3.6 Hz, 1H), 6.73 (dd, *J* = 4.1, 2.7 Hz, 1H), 4.53 (q, *J* = 7.1, 7.1, 7.1 Hz, 2H), 3.84 (s, 3H), 1.42 (t, *J* = 7.1, 7.1 Hz, 3H); $^{13}C\ \{^1H\}$ NMR (125 MHz, $CDCl_3$) δ 180.8, 164.0, 159.9, 146.3, 141.9, 138.7, 134.4, 132.6, 131.3, 130.8, 130.2, 130.1, 129.7, 128.2, 125.5, 125.1, 124.1, 121.9, 116.5, 115.4, 115.1, 114.9, 109.3, 108.5, 61.5, 55.4, 14.6; HRMS (ESI-TOF) m/z: $[M+H]^+$ calculated for $C_{27}H_{21}ClNO_5$: 474.1108; found $[M+H]^+$: 474.1118.

Ethyl 2-(4-bromobenzoyl)-1-(4-(trifluoromethoxy)phenyl)furo[3,2-*e*]indolizine-4-carboxylate (6x)

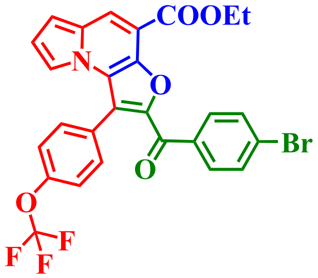

Orange solid, 70%, ^1H NMR (500 MHz, $CDCl_3$) δ 8.32 (s, 1H), 8.16 (d, *J* = 8.6 Hz, 2H), 7.66 (d, *J* = 8.6 Hz, 2H), 7.59 (d, *J* = 8.6 Hz, 2H), 7.41 (d, *J* = 8.2 Hz, 2H), 7.06 (d, *J* = 2.2 Hz, 1H), 6.92 (d, *J* = 4.0 Hz, 1H), 6.76 (dd, *J* = 4.0, 2.7 Hz, 1H), 4.49 (q, *J* = 7.1, 7.1, 7.1 Hz, 2H), 1.45 (t, *J* = 7.1, 7.1 Hz, 3H); $^{13}C\ \{^1H\}$ NMR (125 MHz, $CDCl_3$) δ 180.9, 163.7, 149.9, 146.5, 141.8, 135.6, 131.8, 131.6, 130.8, 128.8, 128.4, 125.1, 124.0, 121.6, 121.2, 119.6, 116.1, 115.6, 109.3, 108.7, 61.5, 14.6; HRMS (ESI-TOF) m/z: $[M+H]^+$ calculated for $C_{27}H_{19}BrF_3NO_5$: 572.0320; found $[M+H]^+$: 572.0332.

Ethyl 2-(3-bromobenzoyl)-1-(naphthalen-1-yl)furo[3,2-*e*]indolizine-4-carboxylate (6y)

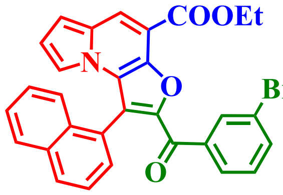

Yellow solid, 70%, ^1H NMR (500 MHz, $CDCl_3$) δ 8.34 (dd, *J* = 2.3, 4.0 Hz, 2H), 8.19 (dt, *J* = 1.2, 7.8 Hz, 1H), 8.04 (d, *J* = 9.6 Hz, 2H), 7.97 (d, *J* = 7.8 Hz, 1H), 7.88 (d, *J* = 7.9 Hz, 1H), 7.68 – 7.65 (m, 1H), 7.63 – 7.54 (m, 3H), 7.35 (t, *J* = 7.9 Hz, 1H), 7.10 (d, *J* = 2.5 Hz, 1H), 6.92 – 6.89 (m, 1H), 6.66 (dd, *J* = 2.7, 4.1 Hz, 1H), 4.55 (q, *J* = 7.1 Hz, 2H), 1.44 (t, *J* = 7.1 Hz, 3H); $^{13}C\ \{^1H\}$ NMR (125 MHz, $CDCl_3$) δ 180.7, 164.0, 146.5, 142.0, 138.9, 135.6, 133.5, 133.3, 133.2, 133.0, 130.8, 128.8, 128.6, 128.4, 127.4, 127.1, 125.7, 125.3, 125.0, 124.3, 122.5, 116.7, 116.3, 115.4, 109.4, 108.6, 61.6, 14.6; HRMS (ESI-TOF) m/z: $[M+H]^+$ calculated for $C_{30}H_{21}BrNO_4$: 538.0654; found $[M+H]^+$: 538.0657.

Ethyl 1-(4-bromophenyl)-2-(4-methylbenzoyl)furo[3,2-*e*]indolizine-4-carboxylate (6z)

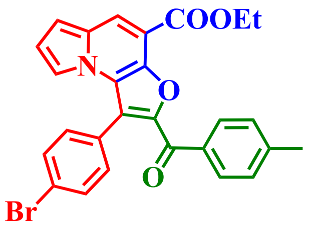

Yellow solid, 74%, ^{1}H NMR (500 MHz, $CDCl_3$) δ 8.30 (s, 1H), 8.20 (d, J = 8.2 Hz, 2H), 7.68 (d, J = 8.4 Hz, 2H), 7.43 (d, J = 8.4 Hz, 2H), 7.31 (d, J = 8.1 Hz, 2H), 7.10 (d, J = 2.4 Hz, 1H), 6.90 (d, J = 4.1 Hz, 1H), 6.74 (dd, J = 2.7, 4.1 Hz, 1H), 4.50 (q, J = 7.2 Hz, 2H), 2.45 (s, 3H), 1.45 (t, J = 7.2 Hz, 3H); ^{13}C {^{1}H} NMR (125 MHz, $CDCl_3$) δ 181.8, 163.9, 147.0, 143.9, 141.4, 134.3, 132.2, 131.5, 130.8, 130.6, 130.3, 129.5, 129.3, 129.2, 129.1, 129.1, 124.8, 124.5, 123.9, 123.7, 123.4, 109.4, 61.4, 21.9, 14.7; HRMS (ESI-TOF) m/z: $[M+H]^+$ calculated for $C_{27}H_{21}BrNO_4$: 502.0654; found $[M+H+2]^+$: 504.0651.

Ethyl 1-(4-bromophenyl)-2-(4-cyanobenzoyl)furo[3,2-*e*]indolizine-4-carboxylate (6aa)

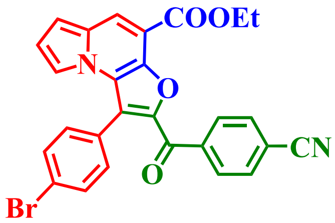

Yellow solid, 63%, ^{1}H NMR (500 MHz, $CDCl_3$) δ 8.37 (d, J = 8.3 Hz, 2H), 8.33 (s, 1H), 7.82 (d, J = 8.3 Hz, 2H), 7.72 (d, J = 8.2 Hz, 2H), 7.44 (d, J = 8.3 Hz, 2H), 7.12 (d, J = 2.2 Hz, 1H), 6.94 (d, J = 4.1 Hz, 1H), 6.77 (dd, J = 2.8, 4.0 Hz, 1H), 4.48 (q, J = 7.1 Hz, 2H), 1.43 (t, J = 7.1 Hz, 3H); ^{13}C {^{1}H} NMR (125 MHz, $CDCl_3$) δ 180.2, 163.5, 146.0, 142.4, 140.3, 132.3, 132.2, 131.4, 130.7, 130.6, 128.8, 125.5, 125.0, 124.0, 123.9, 118.2, 116.5, 116.1, 115.8, 109.1, 61.5, 14.7; HRMS (ESI-TOF) m/z: $[M+H]^+$ calculated for $C_{27}H_{18}BrN_2O_4$: 513.0450; found $[M+H]^+$: 513.0451.

Ethyl 1-(4-bromophenyl)-2-(2-nitrobenzoyl)furo[3,2-*e*]indolizine-4-carboxylate (6ab)

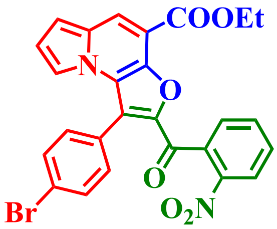

Yellow solid, f76%, ^{1}H NMR (500 MHz, $CDCl_3$) δ 8.26 (s, 1H), 8.18 (d, J = 8.2 Hz, 1H), 7.77 (td, J = 1.0, 7.5 Hz, 1H), 7.67 (t, J = 8.5 Hz, 3H), 7.59 (dd, J = 1.2, 7.5 Hz, 1H), 7.44 (d, J = 8.3 Hz, 2H), 7.12 (d, J = 2.3 Hz, 1H), 6.89 (d, J = 4.1 Hz, 1H), 6.73 (dd, J = 2.7, 4.1 Hz, 1H), 4.32 (q, J = 7.1 Hz, 2H), 1.20 (t, J = 7.1 Hz, 3H); ^{13}C {^{1}H} NMR (125 MHz, $CDCl_3$) δ 181.4, 163.7,

147.1, 145.3, 142.4, 135.0, 134.4, 132.2, 131.5, 131.1, 130.8, 129.8, 128.1, 125.4, 124.2, 123.9, 123.0, 116.1, 115.6, 109.3, 108.7, 61.3, 14.3; HRMS (ESI-TOF) m/z: $[M+H]^+$ calculated for $C_{26}H_{18}BrN_2O_6$: 533.0348; found $[M+H+2]^+$: 533.0348.

Ethyl 1-(4-bromophenyl)-2-(4-(trifluoromethyl)benzoyl)furo[3,2-*e*]indolizine-4-carboxylate (6ac)

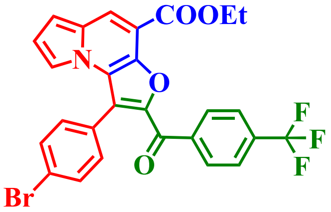

Yellow solid, 69%, ^{1}H NMR (500 MHz, $CDCl_3$) δ 8.36 (d, J = 8.1 Hz, 2H), 8.33 (s, 1H), 7.78 (d, J = 8.2 Hz, 2H), 7.71 (d, J = 8.4 Hz, 2H), 7.44 (d, J = 8.4 Hz, 2H), 7.13 (d, J = 2.6 Hz, 1H), 6.93 (dd, J = 1.0, 4.1 Hz, 1H), 6.76 (dd, J = 2.7, 4.2 Hz, 1H), 4.48 (q, J = 7.1 Hz, 2H), 1.41 (t, J = 7.1 Hz, 3H); ^{13}C {^{1}H} NMR (125 MHz, $CDCl_3$) δ 181.0, 163.7, 146.2, 142.2, 139.9, 134.2, 134.0, 132.3, 131.4, 130.8, 130.5, 129.0, 125.6 – 125.2 (m), 124.9, 124.6, 124.0, 123.8, 122.7, 116.4, 115.6, 109.2, 108.9, 61.5, 14.5; HRMS (ESI-TOF) m/z: $[M+H]^+$ calculated for $C_{27}H_{18}BrF_3NO_4$: 556.0371; found $[M+H]^+$: 556.0369.

Ethyl 2-(4-fluorobenzoyl)-1-(4-methoxyphenyl)-6,8-dimethylfuro[3,2-*e*]indolizine-4-carboxylate (8a)

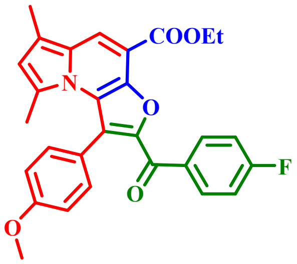

Yellow solid, 78%, ^{1}H NMR (500 MHz, $CDCl_3$) δ 8.30 (dd, J = 8.9, 5.5 Hz, 2H), 8.23 (s, 1H), 7.36 (d, J = 8.6 Hz, 2H), 7.16 (t, J = 8.7 Hz, 2H), 6.99 (d, J = 8.7 Hz, 2H), 6.36 (s, 1H), 4.47 (q, J = 7.1 Hz, 2H), 3.88 (s, 3H), 2.43 (s, 3H), 1.75 (s, 3H), 1.40 (t, J = 7.1 Hz, 3H); ^{13}C {^{1}H} NMR (125 MHz, $CDCl_3$) δ 175.8, 157.4 (d, $J_{C\text{-}F}$ = 552 Hz), 141.3, 139.0, 128.2 (d, $J_{C\text{-}F}$ = 9.0 Hz), 127.1, 126.9, 126.8, 126.7, 125.6, 120.7, 120.6, 120.5, 118.1, 115.6, 113.6, 110.5 (d, $J_{C\text{-}F}$ = 21 Hz), 109.1, 100.2; $C_{26}H_{20}FNO_4$; Calculated [M+H]+: 486.1717; Observed [M+H]+: 486.1717.

Ethyl 2-(4-bromobenzoyl)-6,8-dimethyl-1-(p-tolyl)furo[3,2-e]indolizine-4-carboxylate (8b)

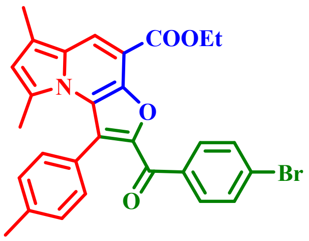

Yellow solid, 76%, ^{1}H NMR (500 MHz, $CDCl_3$) δ 8.23 (s, 1H), 8.12 (d, J = 8.6 Hz, 2H), 7.62 (d, J = 6.8 Hz, 2H), 7.33 (d, J = 8.1 Hz, 2H), 7.27 (s, 2H), 6.36 (s, 1H), 4.47 (q, J = 7.1, 7.1, 7.1 Hz, 2H), 2.44 (s, 3H), 2.43 (s, 3H), 1.71 (s, 3H), 1.41 (t, J = 7.1, 7.1 Hz, 3H); ^{13}C {^{1}H} NMR (125 MHz, $CDCl_3$) δ 80.9, 164.3, 145.9, 144.0, 138.7, 136.4, 131.8, 131.5, 130.5, 130.5, 130.3, 130.2, 129.1, 127.6, 125.9, 125.3, 123.8, 120.5, 118.4, 104.9, 61.1, 21.6, 16.1, 14.7, 11.3; HRMS (ESI-TOF) m/z: $[M+H]^+$ calculated for $C_{29}H_{25}BrNO_4$: 530.0967; found $[M+H]^+$: 530.0980.

Ethyl 2-cyano-1-phenylfuro[3,2-*e*]indolizine-4-carboxylate (10a)

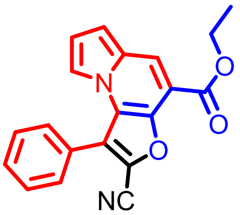

Yellow solid, 50%, ^{1}H NMR (500 MHz, $CDCl_3$) δ 8.29 (s, 1H), 7.60 (s, 5H), 7.41 (d, J = 2.6 Hz, 1H), 6.92 (dd, J = 0.9, 4.1 Hz, 1H), 6.79 (dd, J = 2.7, 4.1 Hz, 1H), 4.50 (q, J = 7.1 Hz, 2H), 1.48 (t, J = 7.1 Hz, 3H); ^{13}C {^{1}H} NMR (125 MHz, $CDCl_3$) δ 163.6, 143.5, 130.7, 130.2, 129.6, 129.5, 128.7, 127.1, 124.6, 123.7, 121.6, 116.1, 115.6, 111.8, 109.4, 108.4, 61.6, 14.5; HRMS (ESI-TOF) m/z: $[M+H]^+$ calculated for $C_{20}H_{15}N_2O_3$: 331.1082; found $[M+H]^+$: 331.1099.

Diethyl 1-phenylfuro[3,2-e]indolizine-2,4-dicarboxylate (10b)

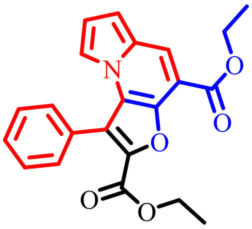

Yellow solid, 40%, ^{1}H NMR (500 MHz, $CDCl_3$) δ 8.23 (s, 1H), 7.53 – 7.52 (m, 3H), 7.49 (d, J = 3.7 Hz, 2H), 7.02 (d, J = 2.6 Hz, 1H), 6.85 – 6.84 (m, 1H), 6.69 – 6.68 (m, 1H), 4.49 (t, J = 7.1 Hz, 2H), 4.27 (t, J = 7.1 Hz, 2H), 1.49 (t, J = 7.1 Hz, 3H), 1.22 (t, J = 7.1 Hz, 3H); ^{13}C {^{1}H} NMR (125 MHz, $CDCl_3$) δ 178.8, 164.1, 158.9, 141.5, 130.7, 130.0, 129.6, 129.1, 128.9, 128.7, 127.8, 123.8, 115.5, 115.1, 107.7, 61.4, 61.2, 14.4, 14.1

Spectral details

1. Ethyl 2(4-nitrophenyl)-1-phenylfuro[3,2-*e*]indolizine-4-carboxylate (4a)

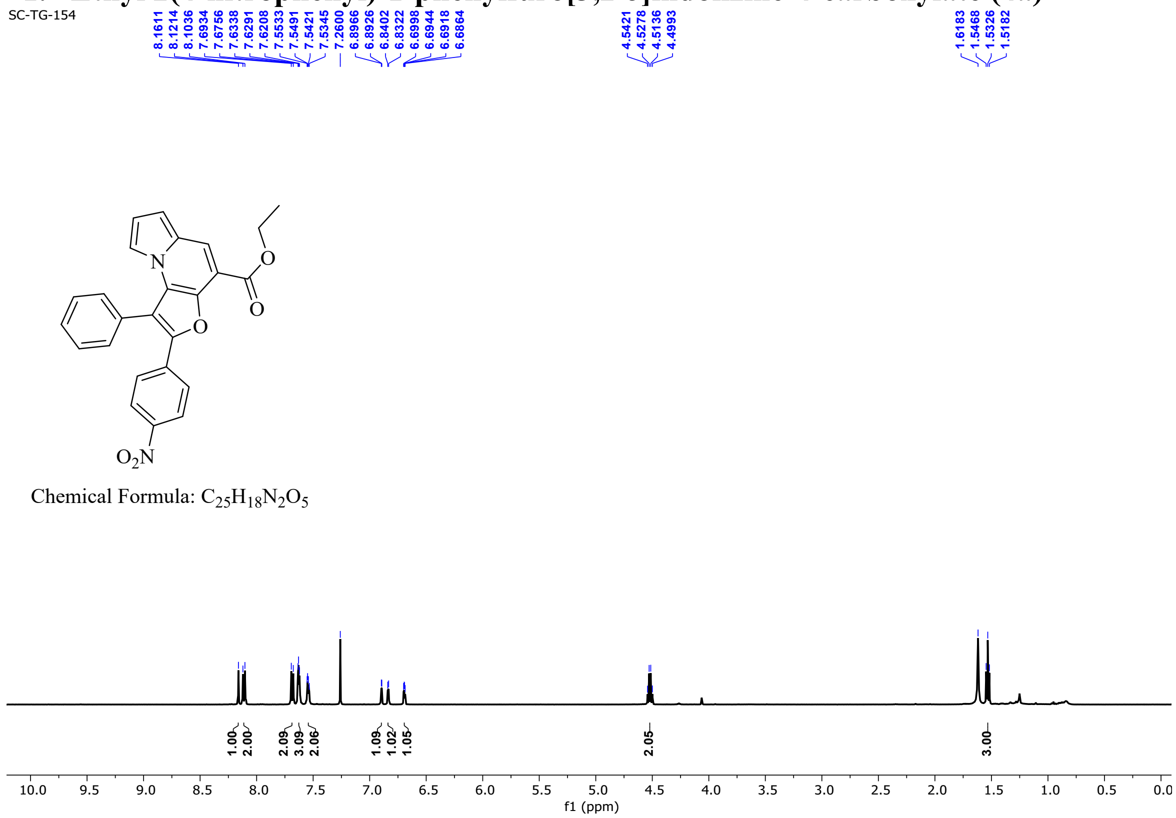

Figure S3. ^{1}H NMR Spectrum of Ethyl 2-(4-nitrophenyl)-1-phenylfuro[3,2-*e*]indolizine-4-carboxylate (4a)

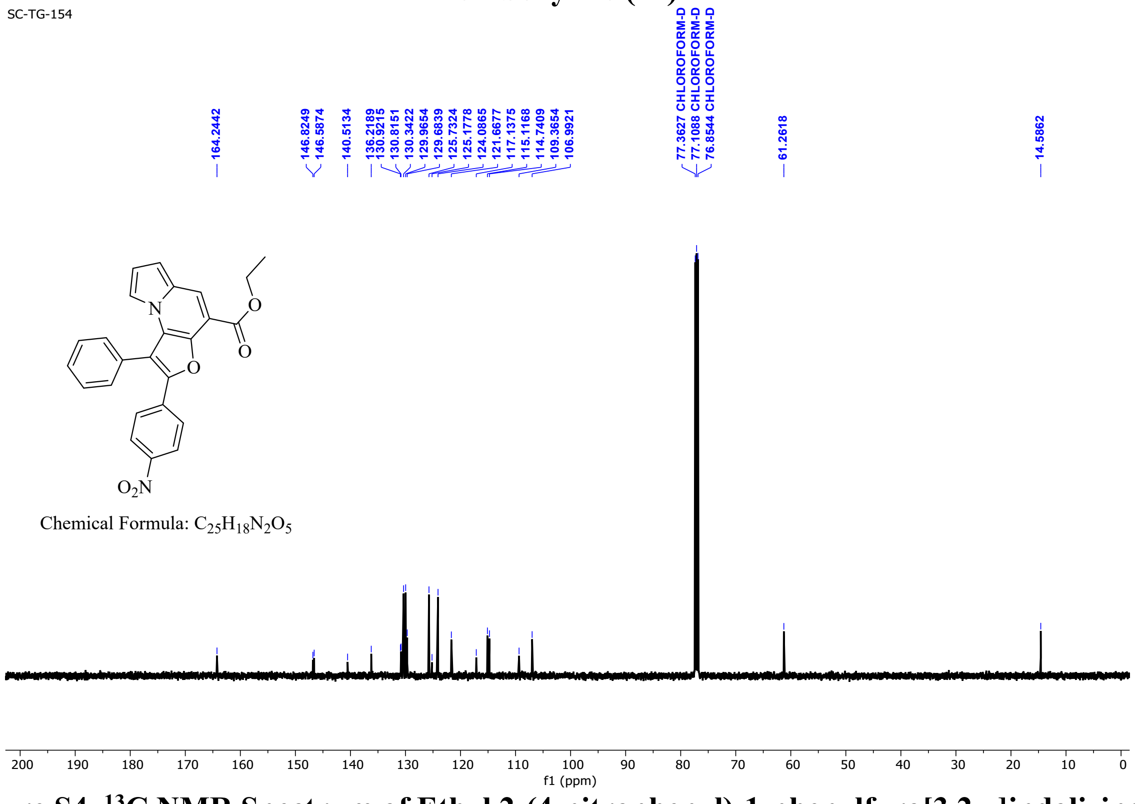

Figure S4. ^{13}C NMR Spectrum of Ethyl 2-(4-nitrophenyl)-1-phenylfuro[3,2-*e*]indolizine-4-carboxylate (4a)

2. Ethyl 1-(4-methoxyphenyl)-2-(4-nitrophenyl)furo[3,2-*e*]indolizine-4-carboxylate (4b)

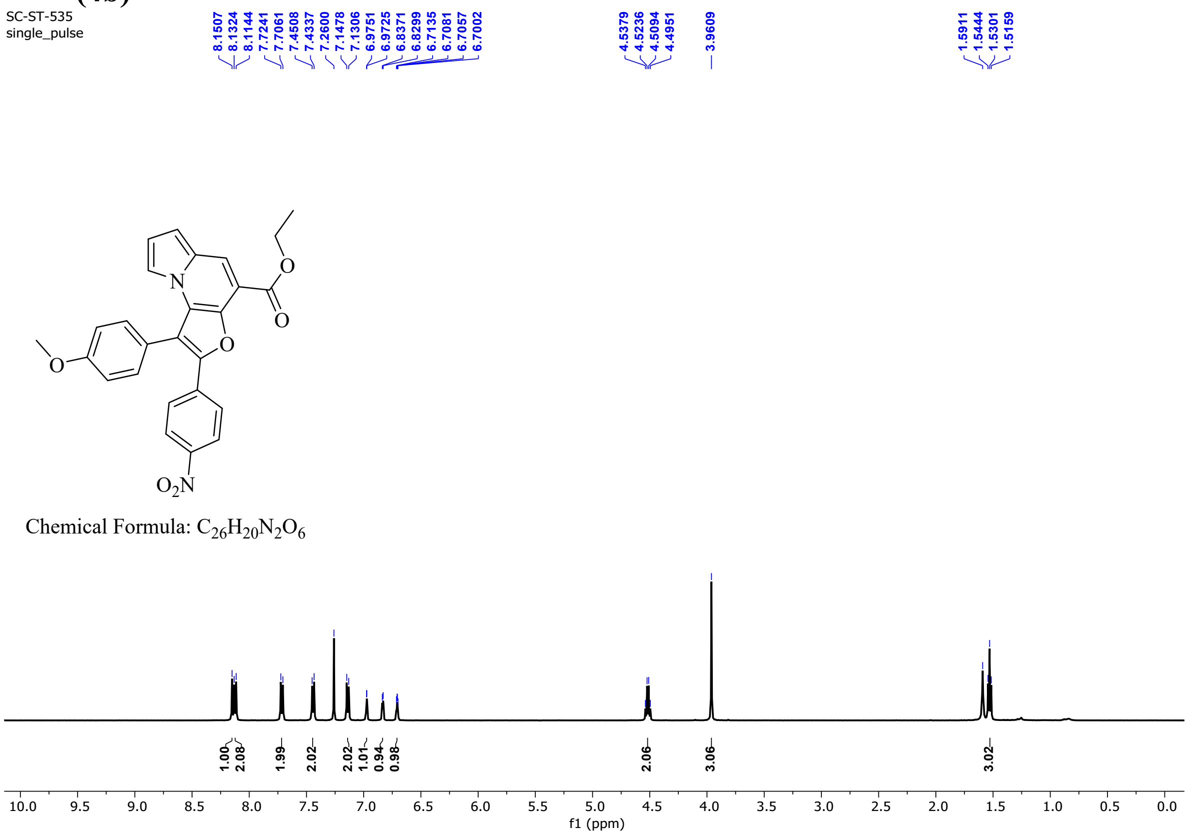

Figure S5. ^{1}H NMR Spectrum of Ethyl 1-(4-methoxyphenyl)-2-(4-nitrophenyl)furo[3,2-*e*]indolizine-4-carboxylate (4b)

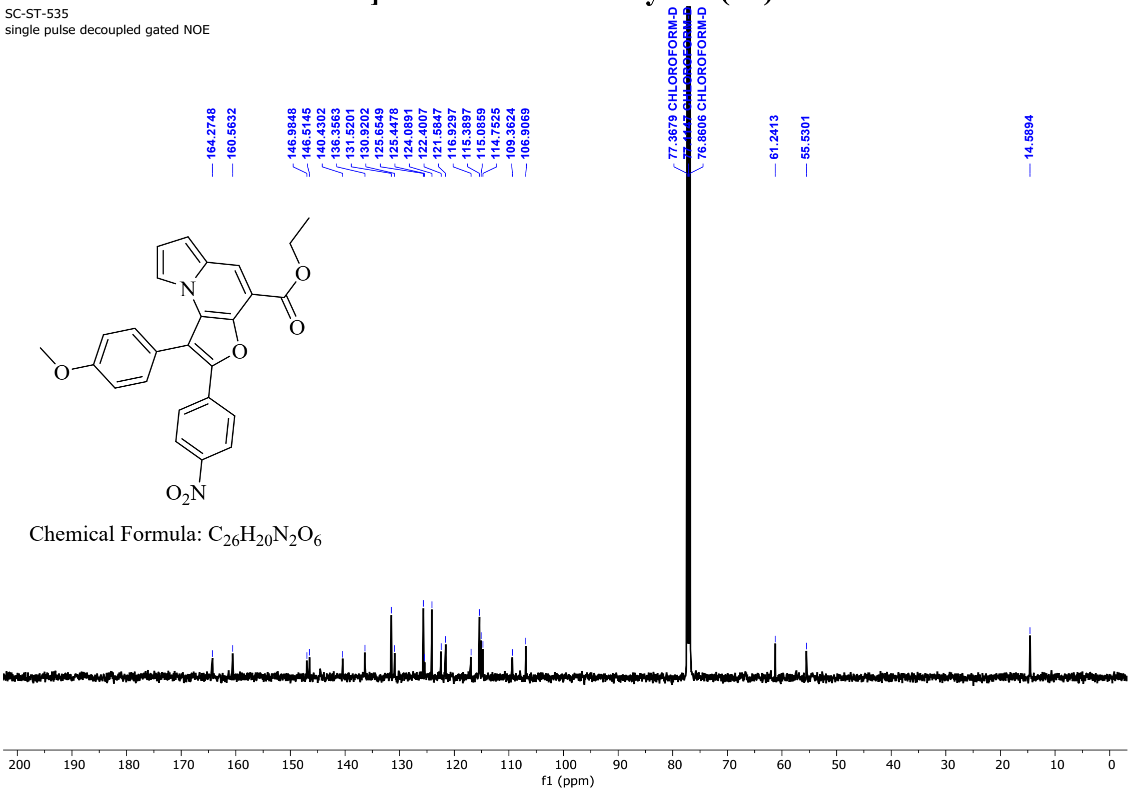

Figure S6. ^{13}C NMR Spectrum of Ethyl 1-(4-methoxyphenyl)-2-(4-nitrophenyl)furo[3,2-*e*]indolizine-4-carboxylate (4b)

3. Ethyl 2-(4-nitrophenyl)-1-(p-tolyl)furo[3,2-*e*]indolizine-4-carboxylate (4c)

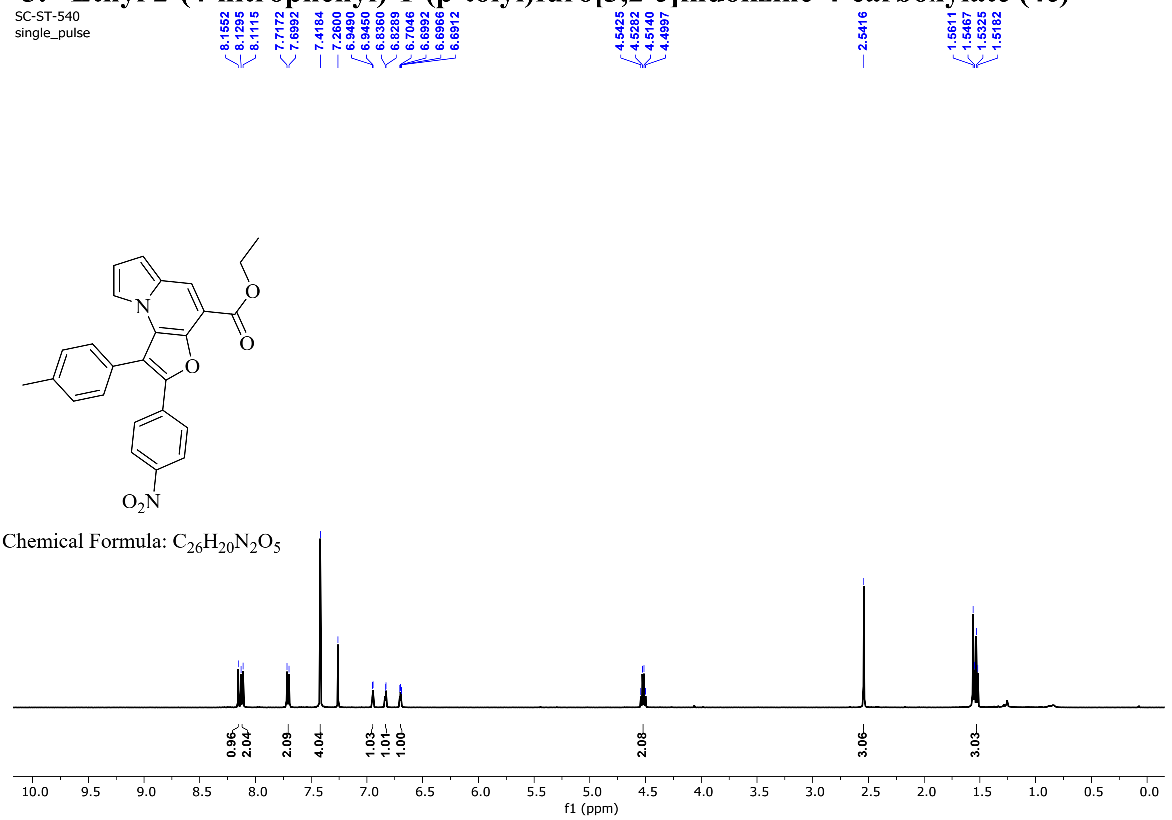

Figure S7. ^{1}H NMR Spectrum of Ethyl 2-(4-nitrophenyl)-1-(p-tolyl)furo[3,2-*e*]indolizine-4-carboxylate (4c)

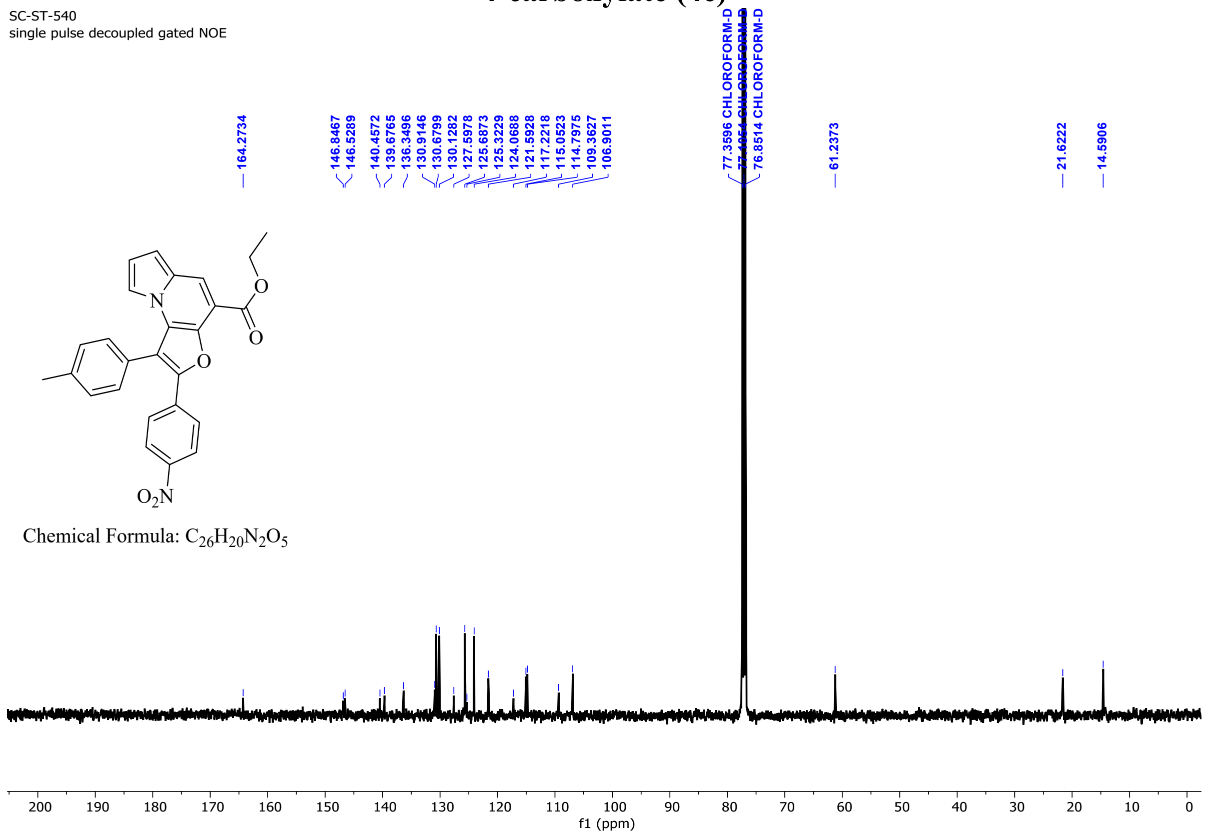

Figure S8. ^{13}C NMR Spectrum of Ethyl 2-(4-nitrophenyl)-1-(p-tolyl)furo[3,2-*e*]indolizine-4-carboxylate (4c)

4. Ethyl 1-(4-fluorophenyl)-2-(4-nitrophenyl)furo[3,2-*e*]indolizine-4-carboxylate (4d)

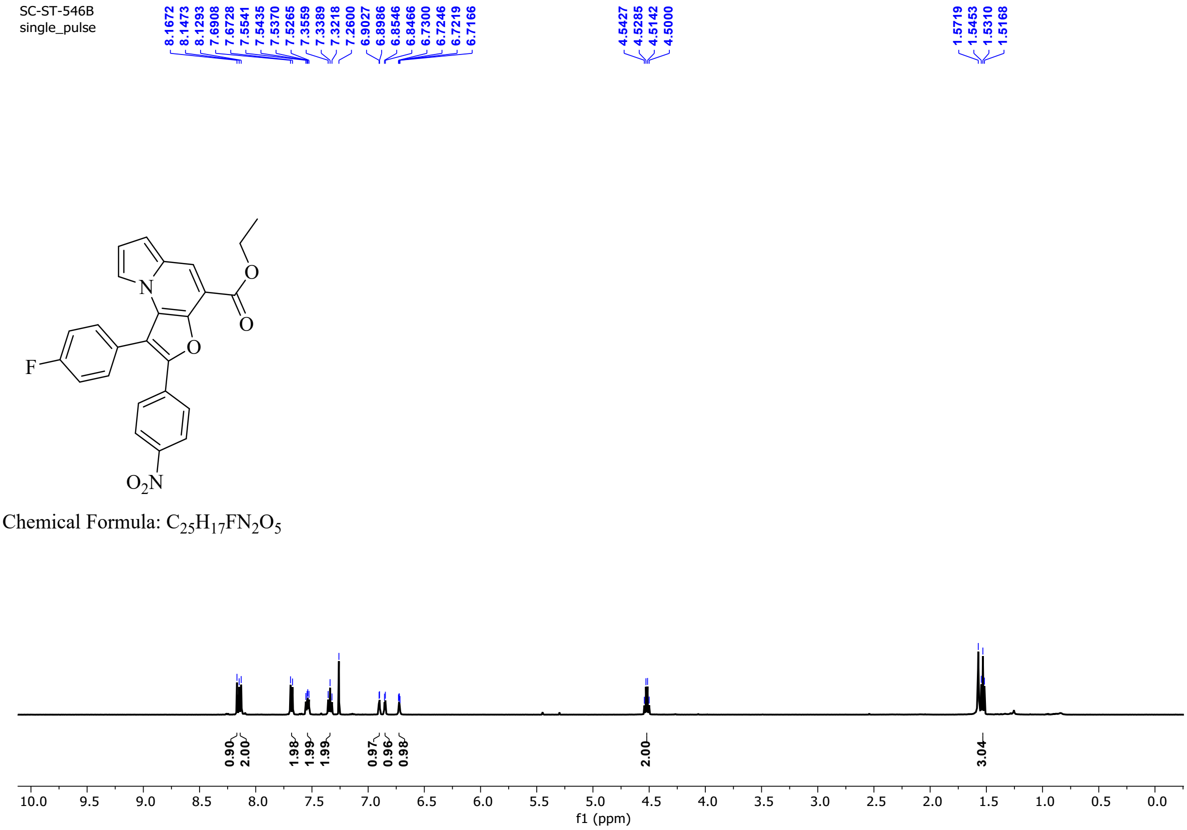

Figure S9. ¹H NMR Spectrum of Ethyl 1-(4-fluorophenyl)-2-(4-nitrophenyl)furo[3,2-*e*]indolizine-4-carboxylate (4d)

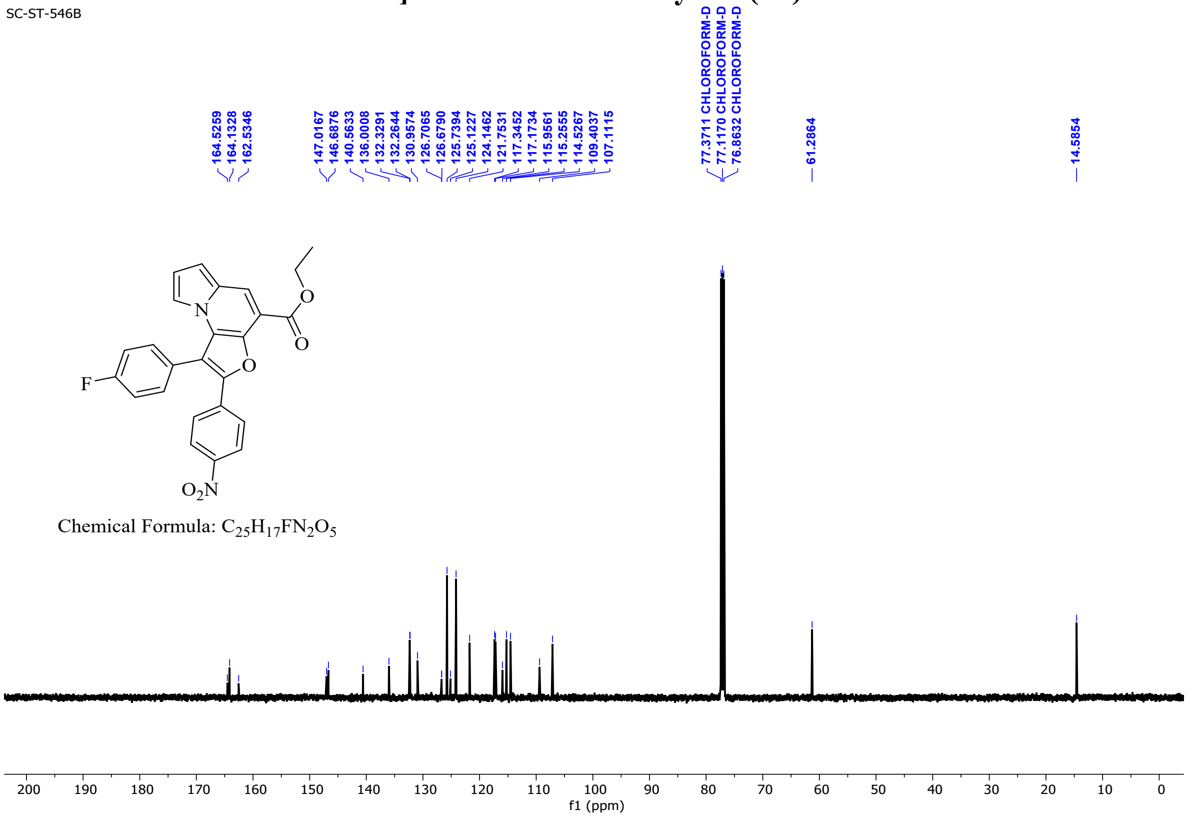

Figure S10. ¹³C NMR Spectrum of Ethyl 1-(4-fluorophenyl)-2-(4-nitrophenyl)furo[3,2-*e*]indolizine-4-carboxylate (4d)

5. Ethyl 1-(4-chlorophenyl)-2-(4-nitrophenyl)furo[3,2-*e*]indolizine-4-carboxylate (4e)

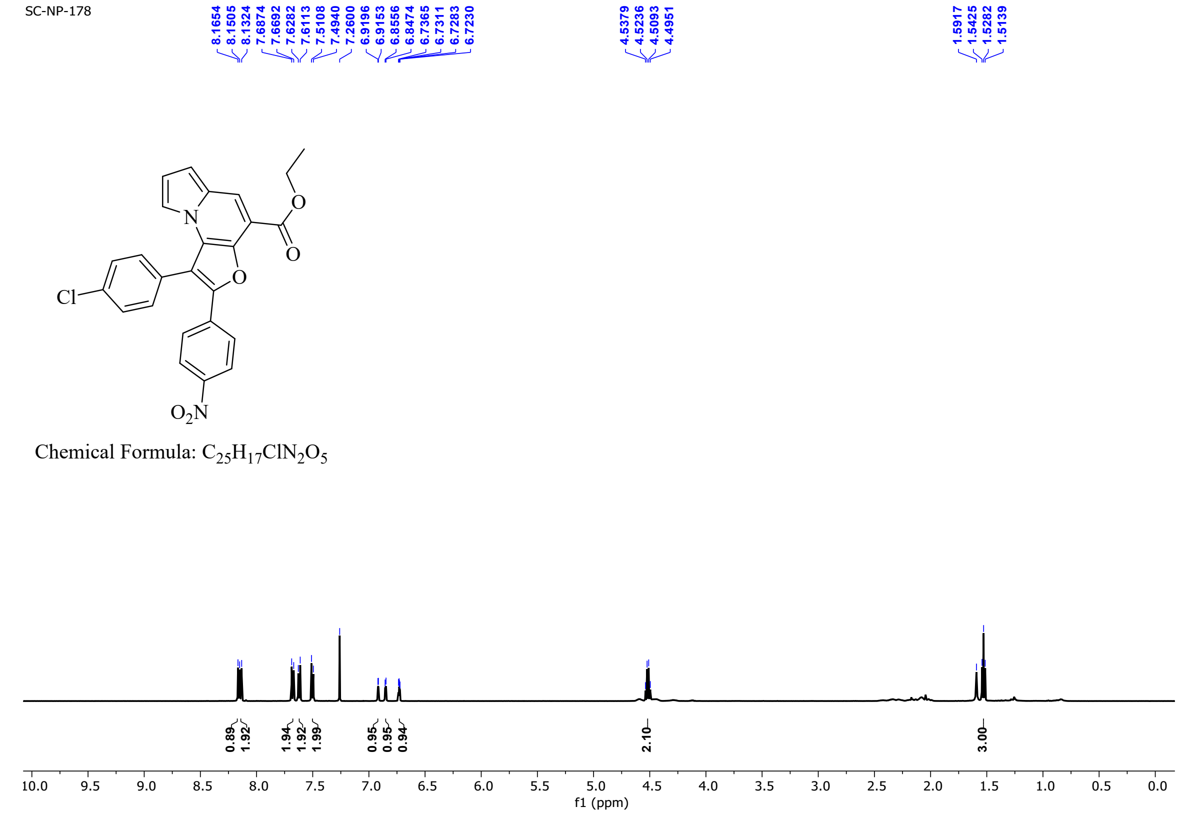

Figure S11. ¹H NMR Spectrum of Ethyl 1-(4-chlorophenyl)-2-(4-nitrophenyl)furo[3,2-*e*]indolizine-4-carboxylate (4e)

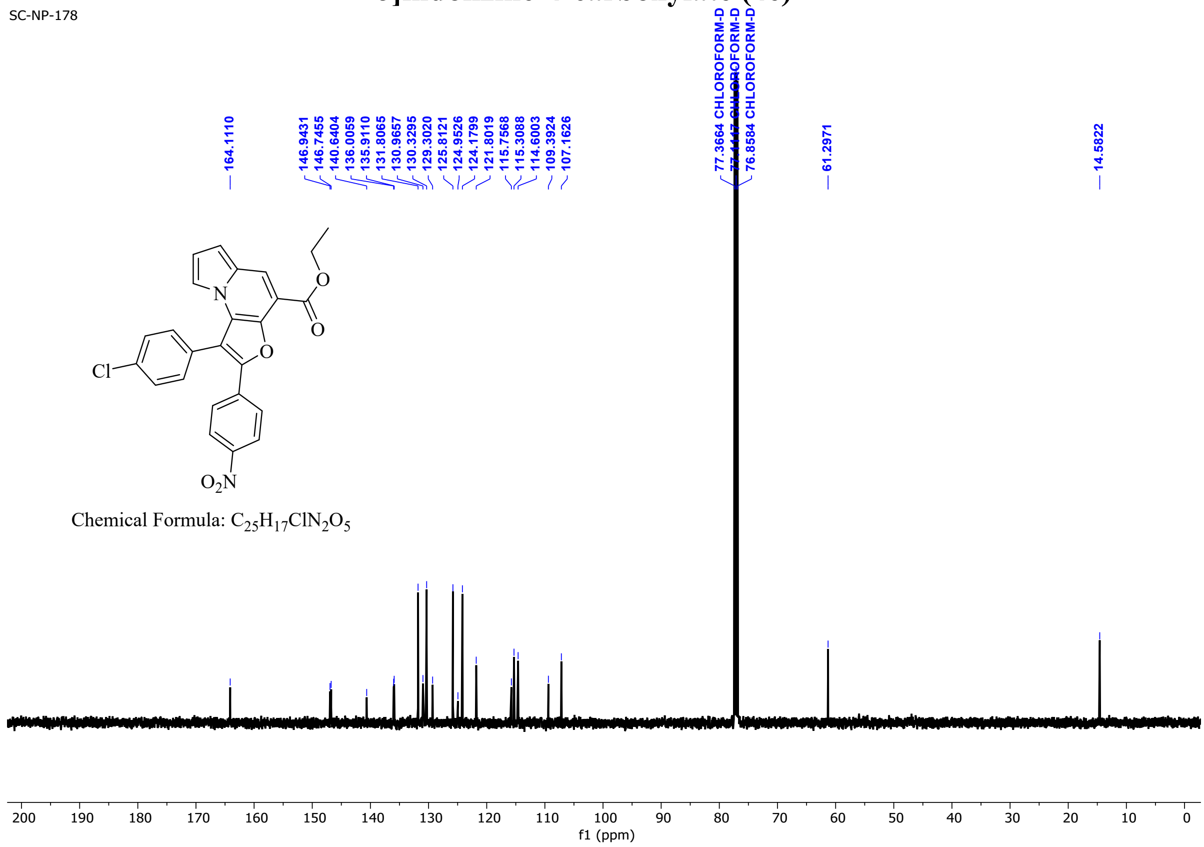

Figure S12. ¹³C NMR Spectrum of Ethyl 1-(4-chlorophenyl)-2-(4-nitrophenyl)furo[3,2-*e*]indolizine-4-carboxylate (4e)

6. Ethyl 1-(4-bromophenyl)-2-(4-nitrophenyl)furo[3,2-*e*]indolizine-4-carboxylate (4f)

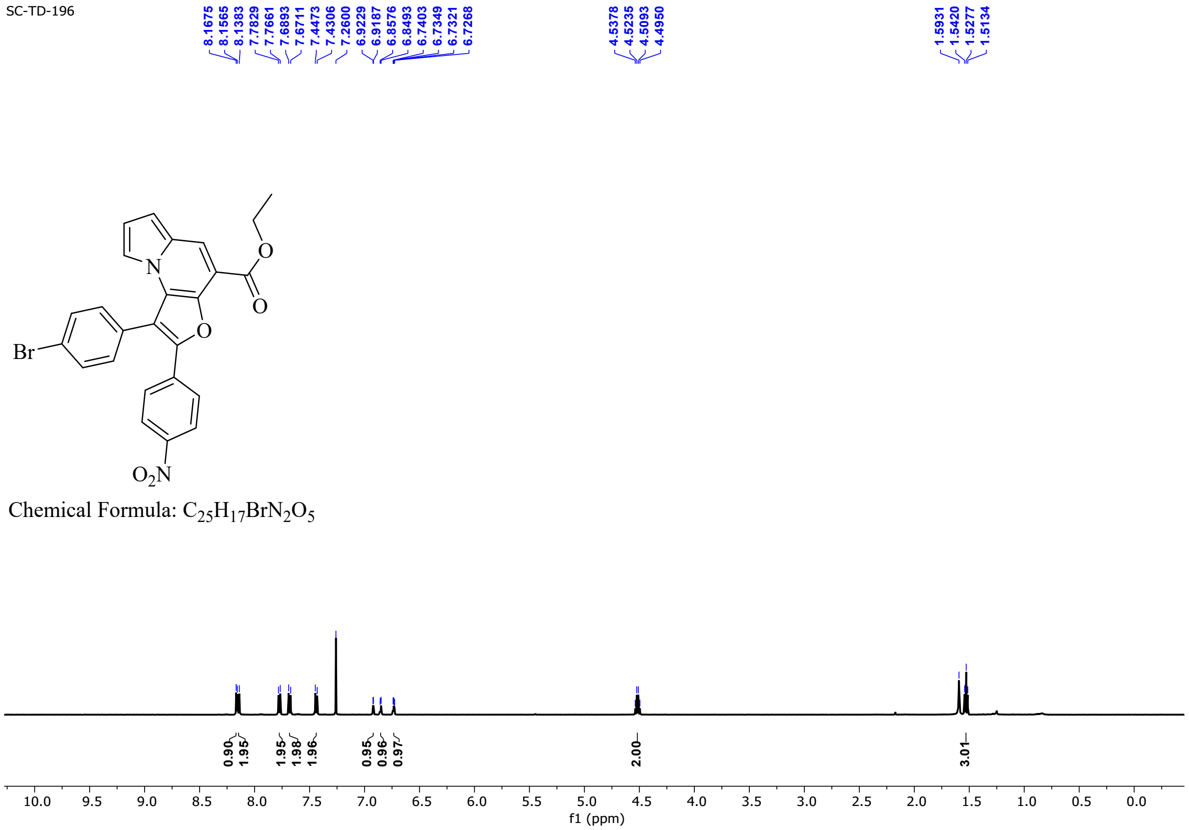

Figure S13. ^{1}**H NMR Spectrum of Ethyl 1-(4-bromophenyl)-2-(4-nitrophenyl)furo[3,2-*e*]indolizine-4-carboxylate (4f)**

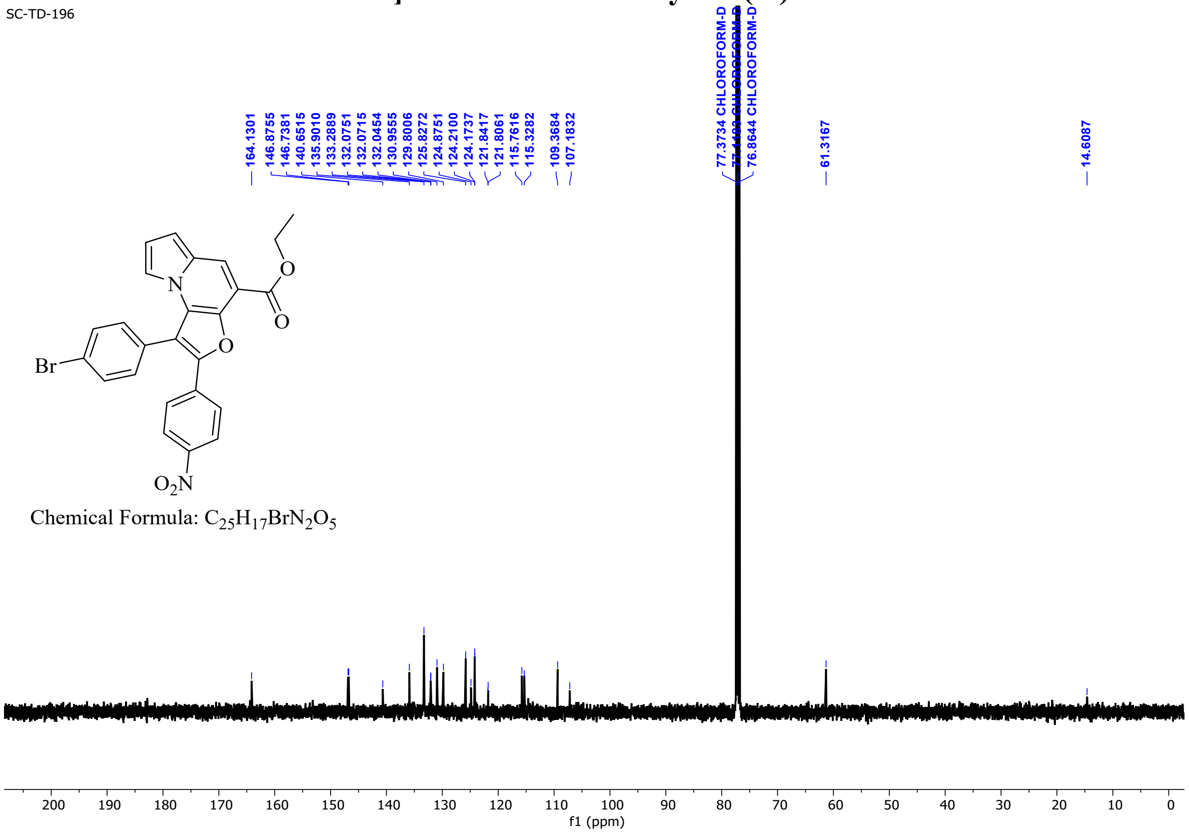

Figure S14. ^{13}**C NMR Spectrum of Ethyl 1-(4-bromophenyl)-2-(4-nitrophenyl)furo[3,2-*e*]indolizine-4-carboxylate (4f)**

7. Ethyl 1-(3-methoxyphenyl)-2-(4-nitrophenyl)furo[3,2-*e*]indolizine-4-carboxylate (4g)

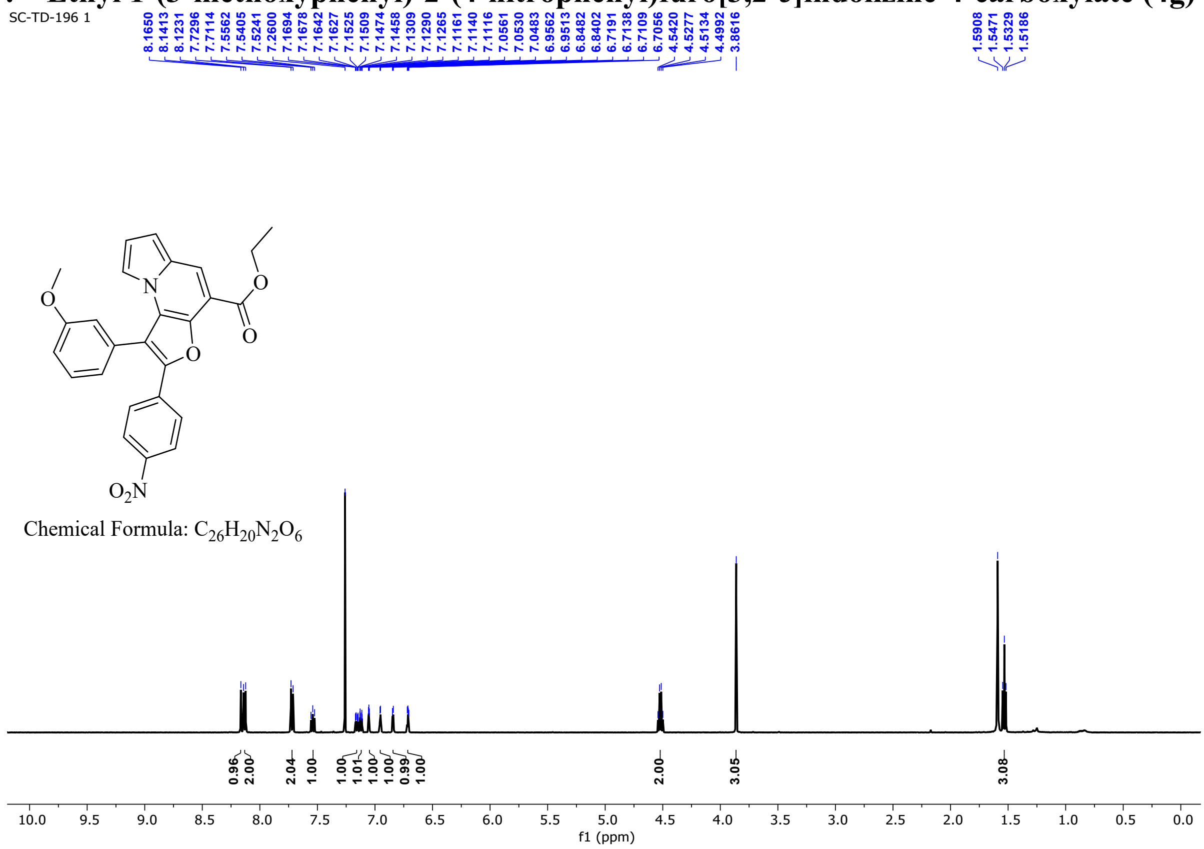

Figure S15. ^{1}H NMR Spectrum of Ethyl 1-(3-methoxyphenyl)-2-(4-nitrophenyl)furo[3,2-*e*]indolizine-4-carboxylate (4g)

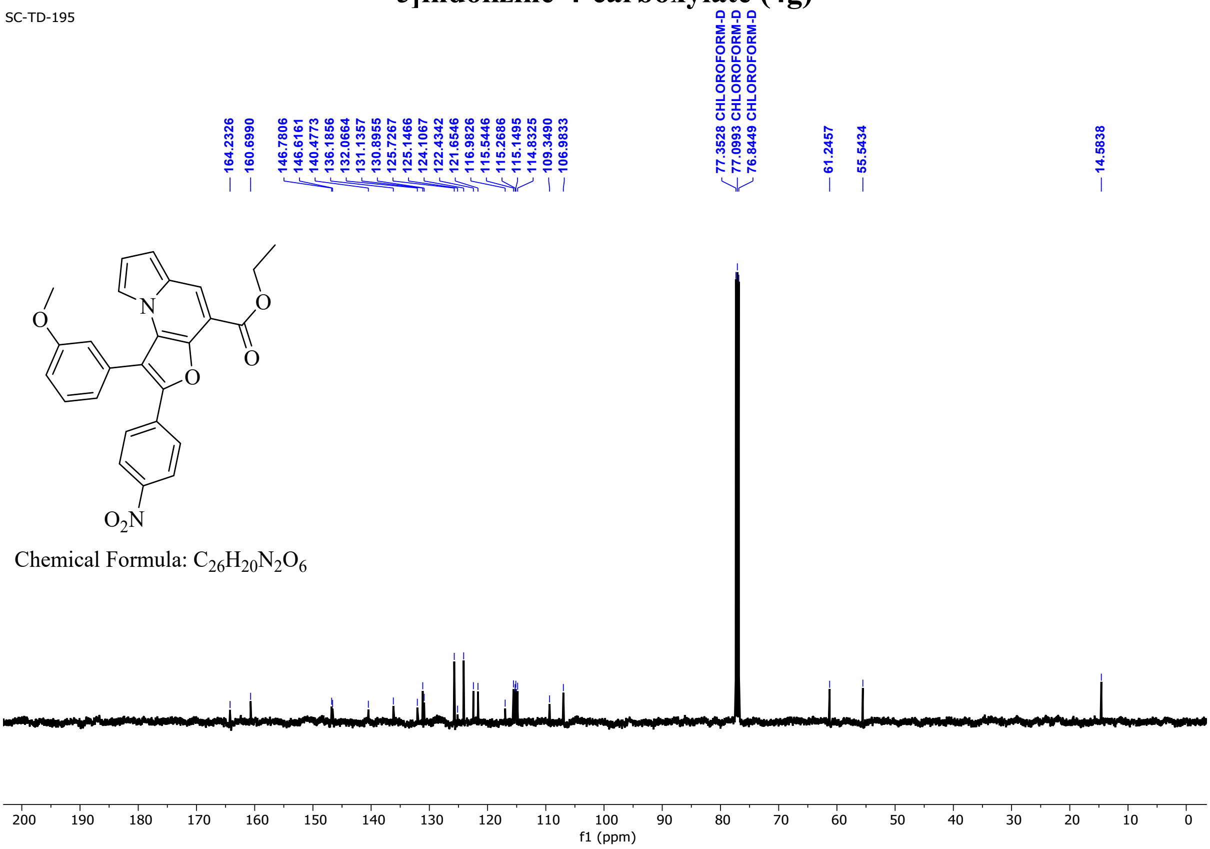

Figure S16. ^{13}C NMR Spectrum of Ethyl 1-(3-methoxyphenyl)-2-(4-nitrophenyl)furo[3,2-*e*]indolizine-4-carboxylate (4g)

8. ethyl 2-(4-nitrophenyl)-1-(4-(trifluoromethyl)phenyl)furo[3,2-e]indolizine-4-carboxylate (4h)

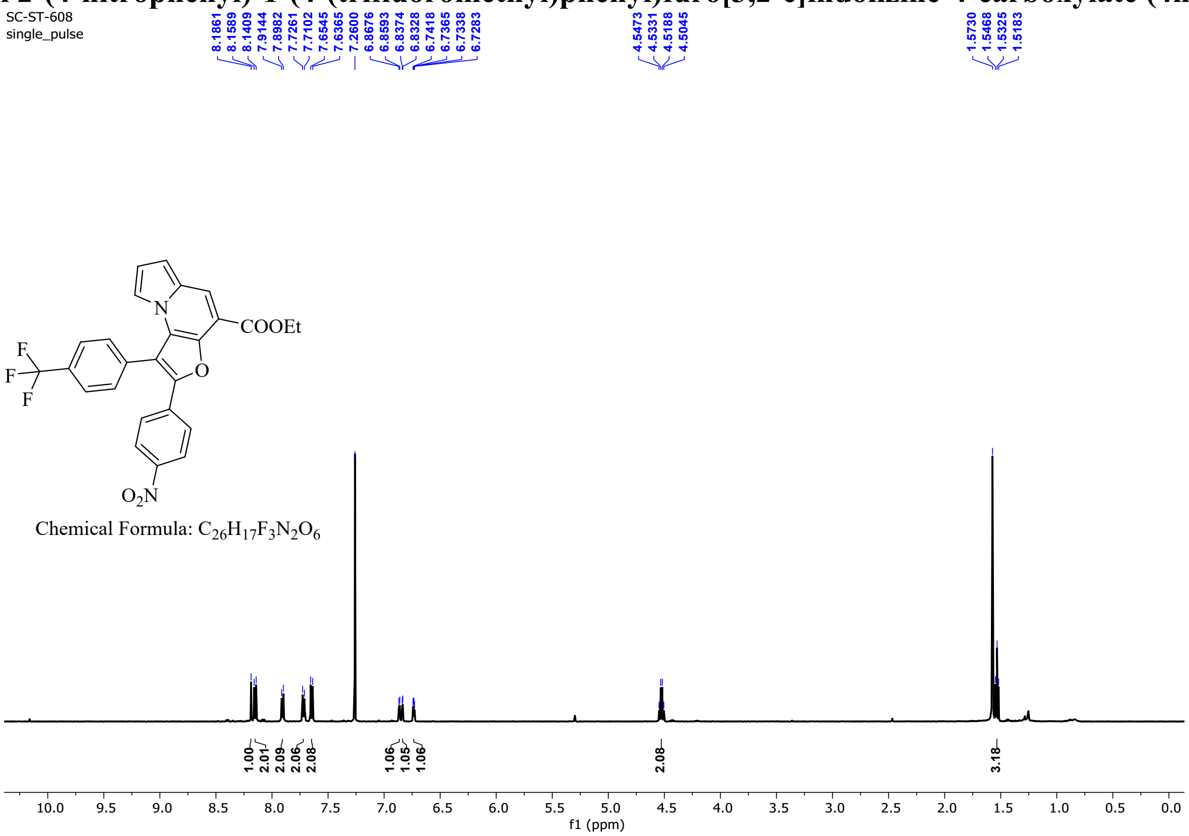

Figure S17. ^{1}H NMR Spectrum of Ethyl 2-(4-nitrophenyl)-1-(4-(trifluoromethyl)phenyl) furo[3,2-*e*]indolizine-4-carboxylate (4h)

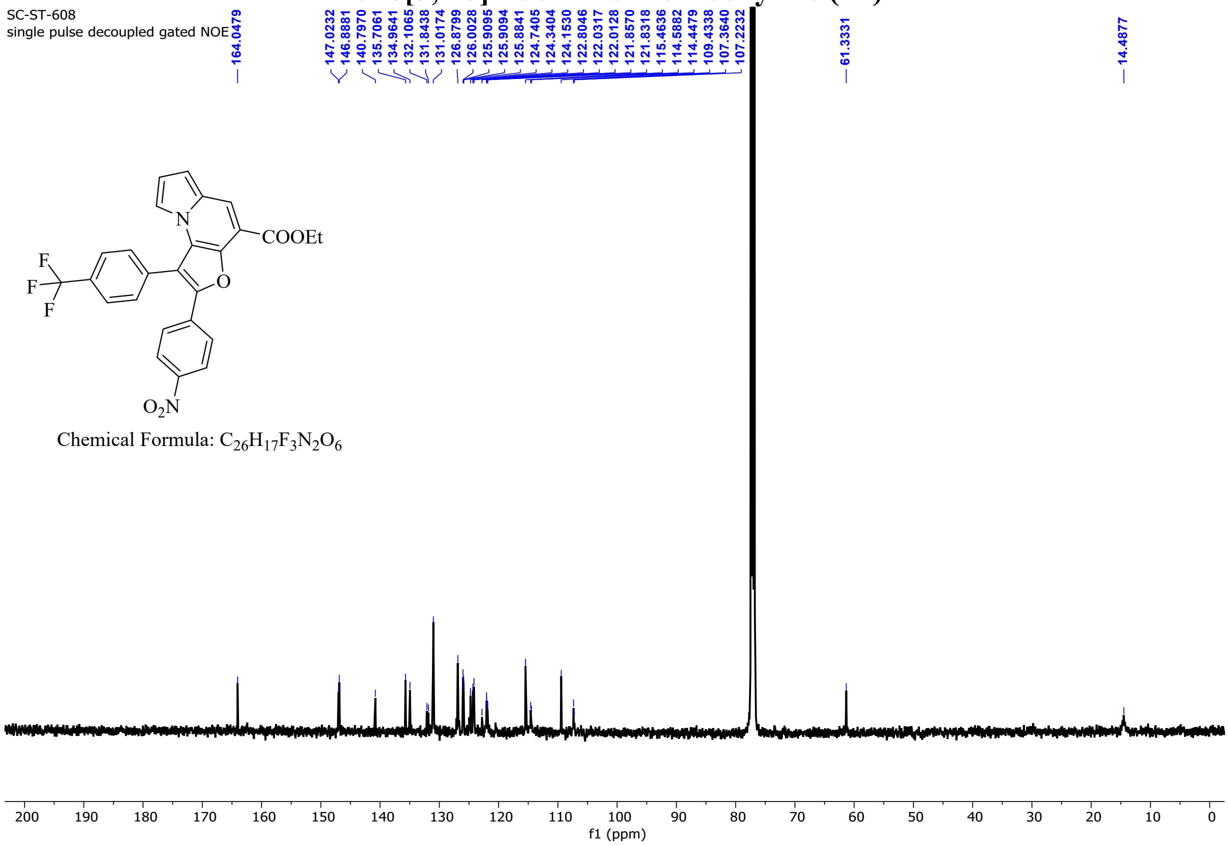

Figure S18. ^{13}C NMR Spectrum of Ethyl 2-(4-nitrophenyl)-1-(4-(trifluoromethyl)phenyl) furo[3,2-*e*]indolizine-4-carboxylate (4h)

9. Ethyl 2-(4-nitrophenyl)-1-(4-trifluoromethoxy)phenyl)uro[3,2-*e*]indolizine-4-carboxylate (4i)

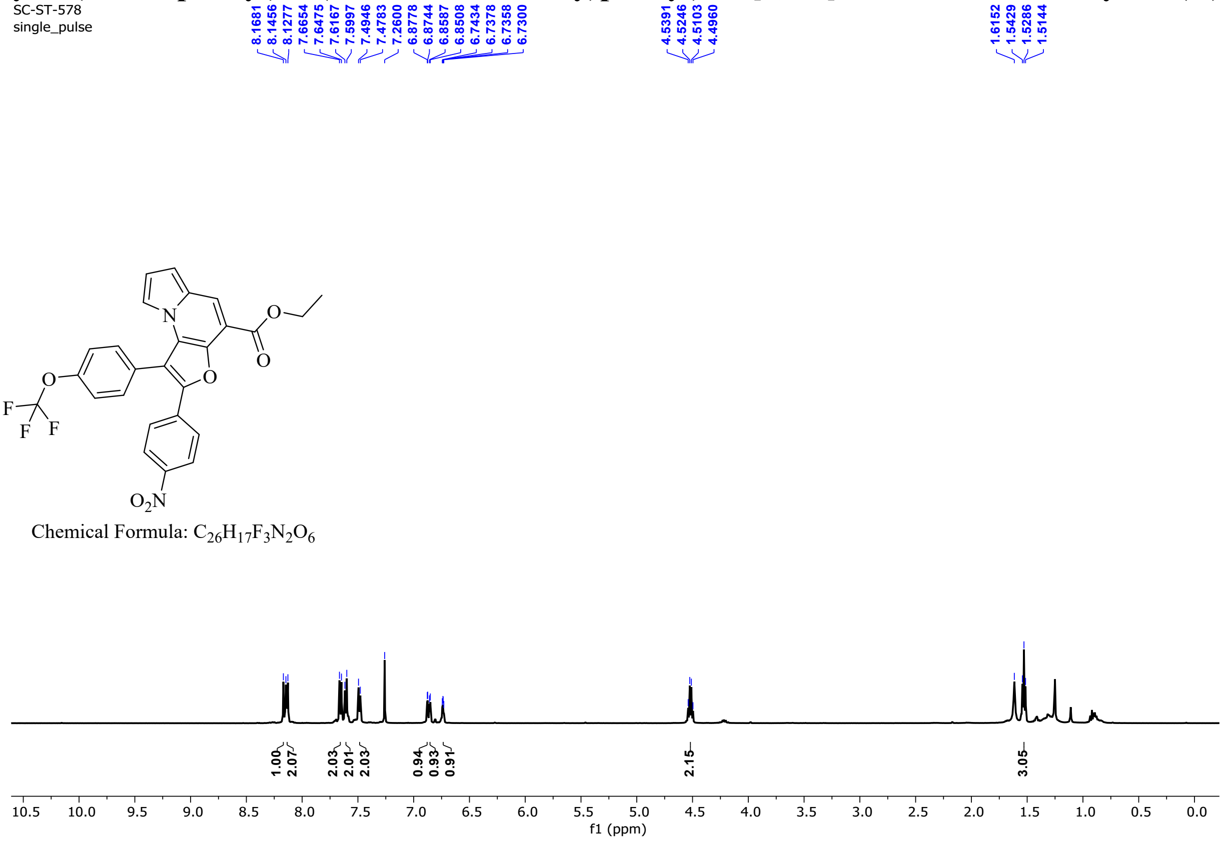

Figure S19. ^{1}H **NMR Spectrum of Ethyl 2-(4-nitrophenyl)-1-(4-trifluoromethoxy)phenyl) furo[3,2-*e*]indolizine-4-carboxylate (4i)**

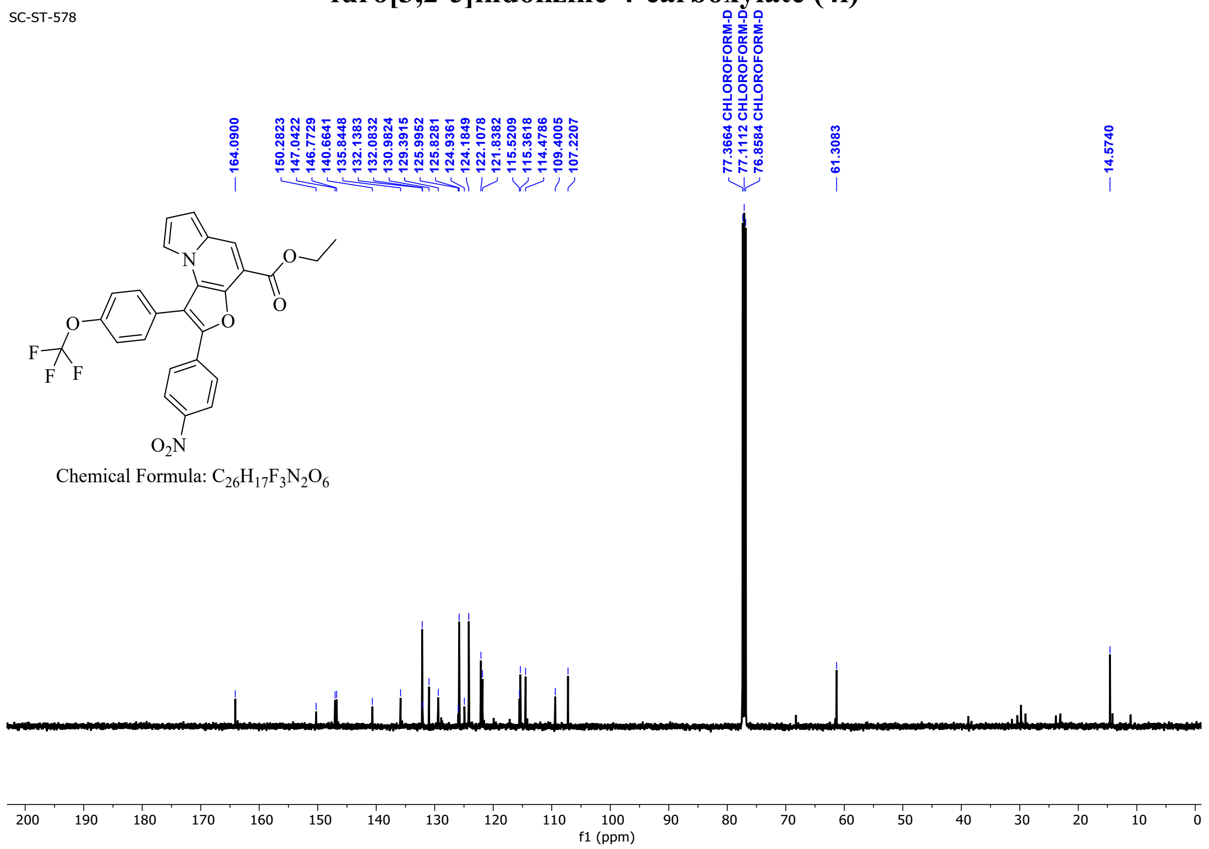

Figure S20. ^{13}C **NMR Spectrum of Ethyl 2-(4-nitrophenyl)-1-(4-trifluoromethoxy)phenyl) furo[3,2-*e*]indolizine-4-carboxylate (4i)**

10. Ethyl 1-([1,1'-biphenyl]-4-yl)-2-(4-nitrophenyl)furo[3,2-*e*]indolizine-4-carboxylate (4j)

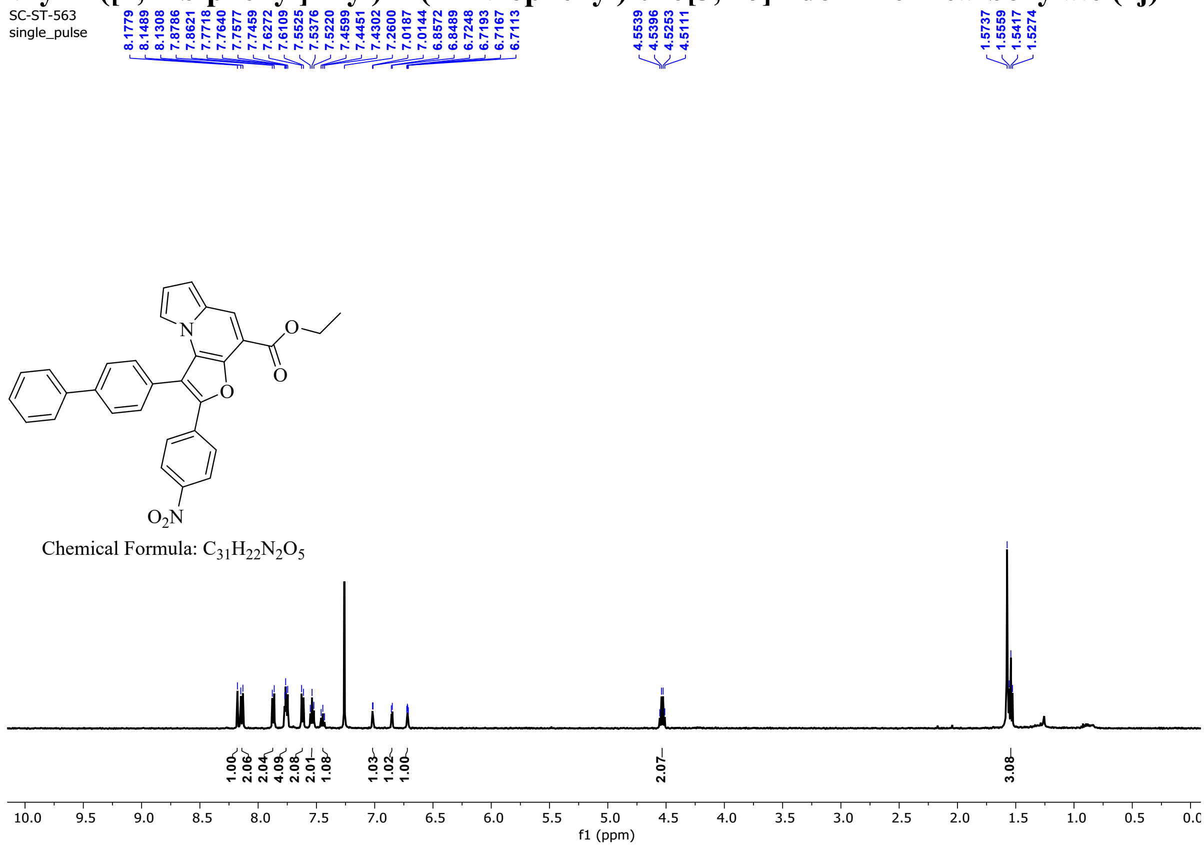

Figure S21. ^{1}H NMR Spectrum of Ethyl 1-([1,1'-biphenyl]-4-yl)-2-(4-nitrophenyl)furo[3,2-*e*]indolizine-4-carboxylate (4j)

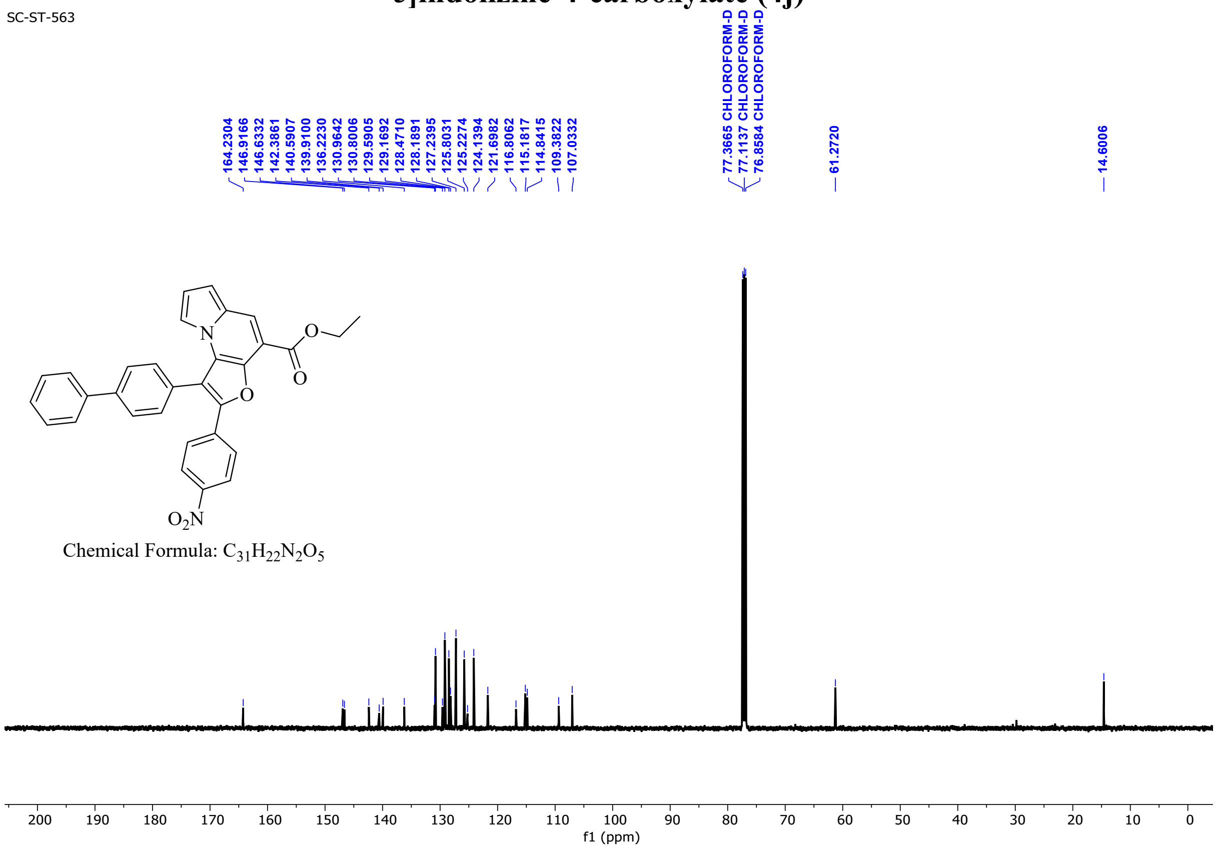

Figure S22. ^{13}C NMR Spectrum of Ethyl 1-([1,1'-biphenyl]-4-yl)-2-(4-nitrophenyl)furo[3,2-*e*]indolizine-4-carboxylate (4j)

11. Ethyl 1-(naphthalen-1-yl)-2-(4-nitrophenyl)furo[3,2-*e*]indolizine-4-carboxylate (4k)

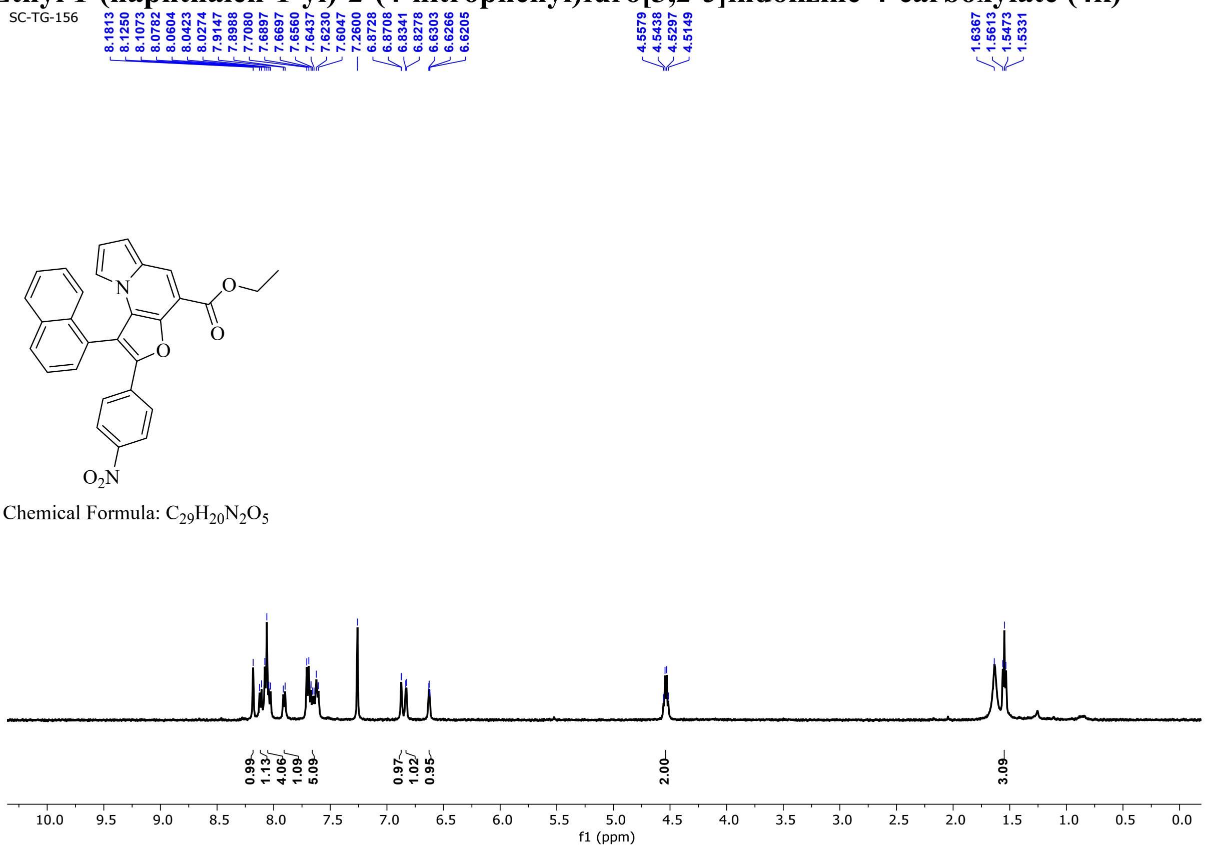

Figure S23. ^{1}H NMR Spectrum of Ethyl 1-(naphthalen-1-yl)-2-(4-nitrophenyl)furo[3,2-*e*]indolizine-4-carboxylate (4k)

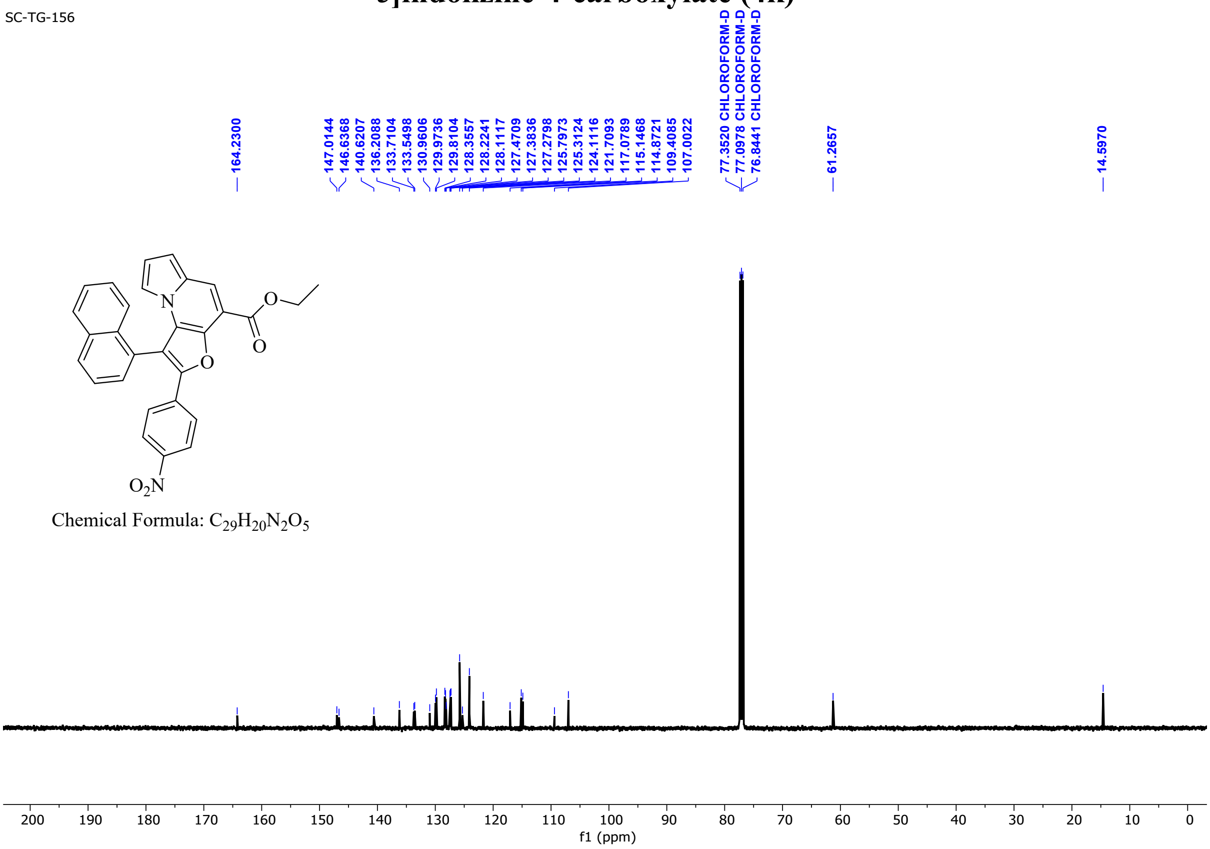

Figure S24. ^{13}C NMR Spectrum of Ethyl 1-(naphthalen-1-yl)-2-(4-nitrophenyl)furo[3,2-*e*]indolizine-4-carboxylate (4k)

12. Ethyl 2-(4-cyanophenyl)-1-phenylfuro[3,2-*e*]indolizine-4-carboxylate (4l)

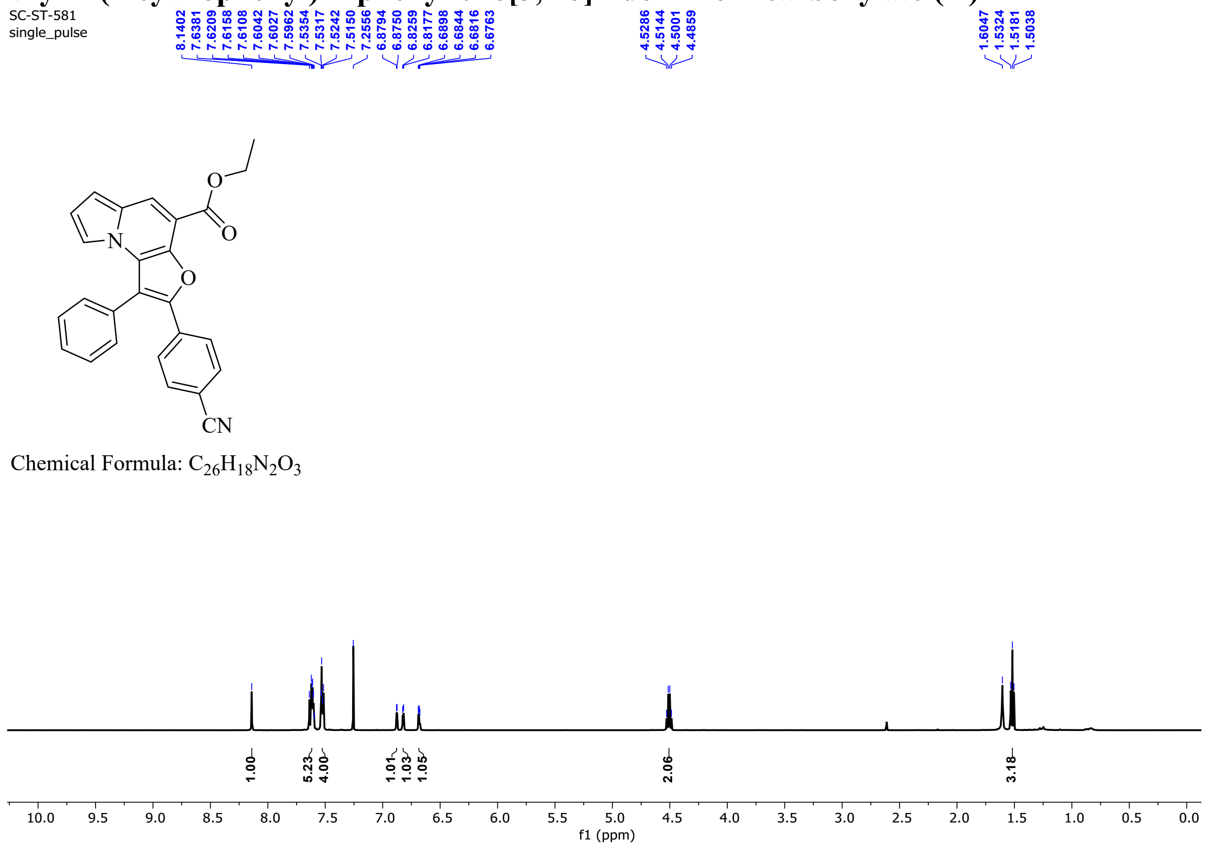

Figure S25. **^{1}H NMR Spectrum of Ethyl 2-(4-cyanophenyl)-1-phenylfuro[3,2-*e*]indolizine-4-carboxylate (4l)**

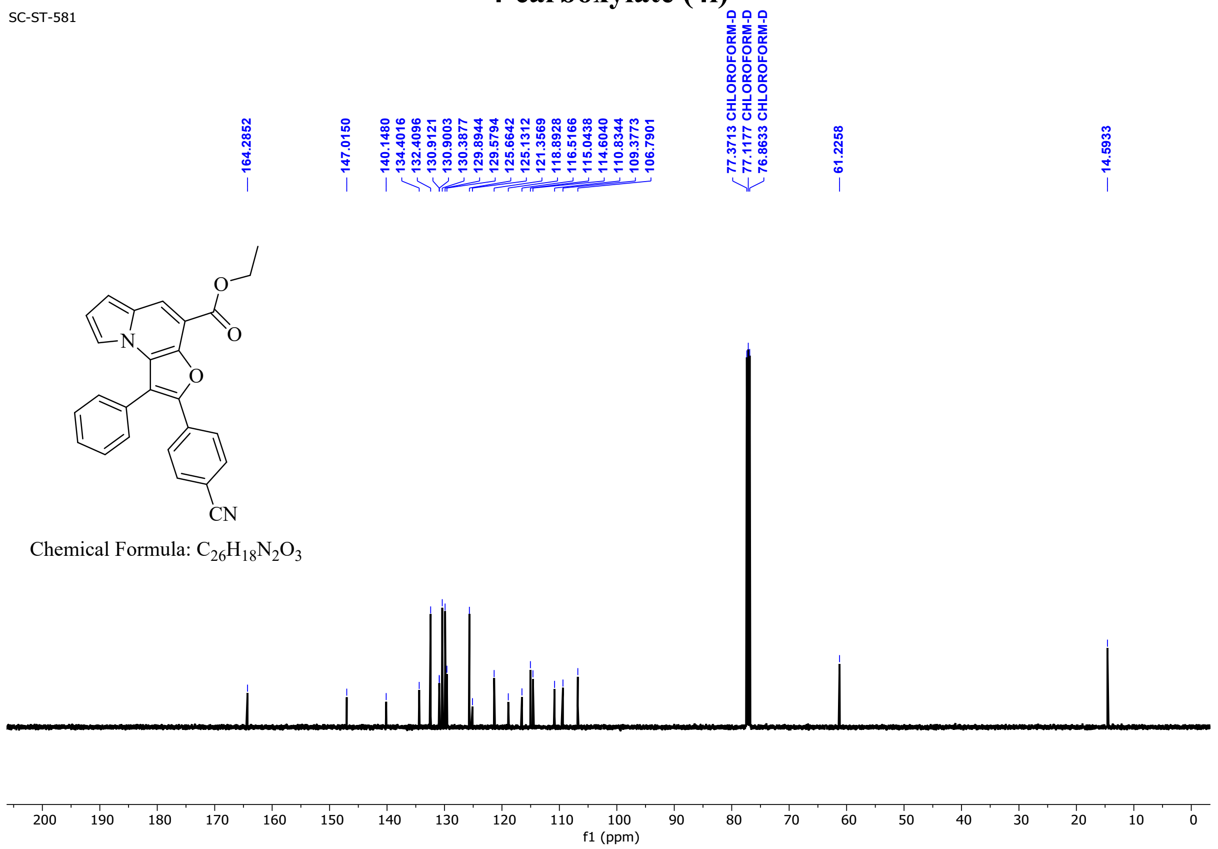

Figure S26. **^{13}C NMR Spectrum of Ethyl 2-(4-cyanophenyl)-1-phenylfuro[3,2-*e*]indolizine-4-carboxylate (4l)**

13. Ethyl 2-(4-cyanophenyl)-1-(4-methoxyphenyl)furo[3,2-*e*]indolizine-4-carboxylate (4m)

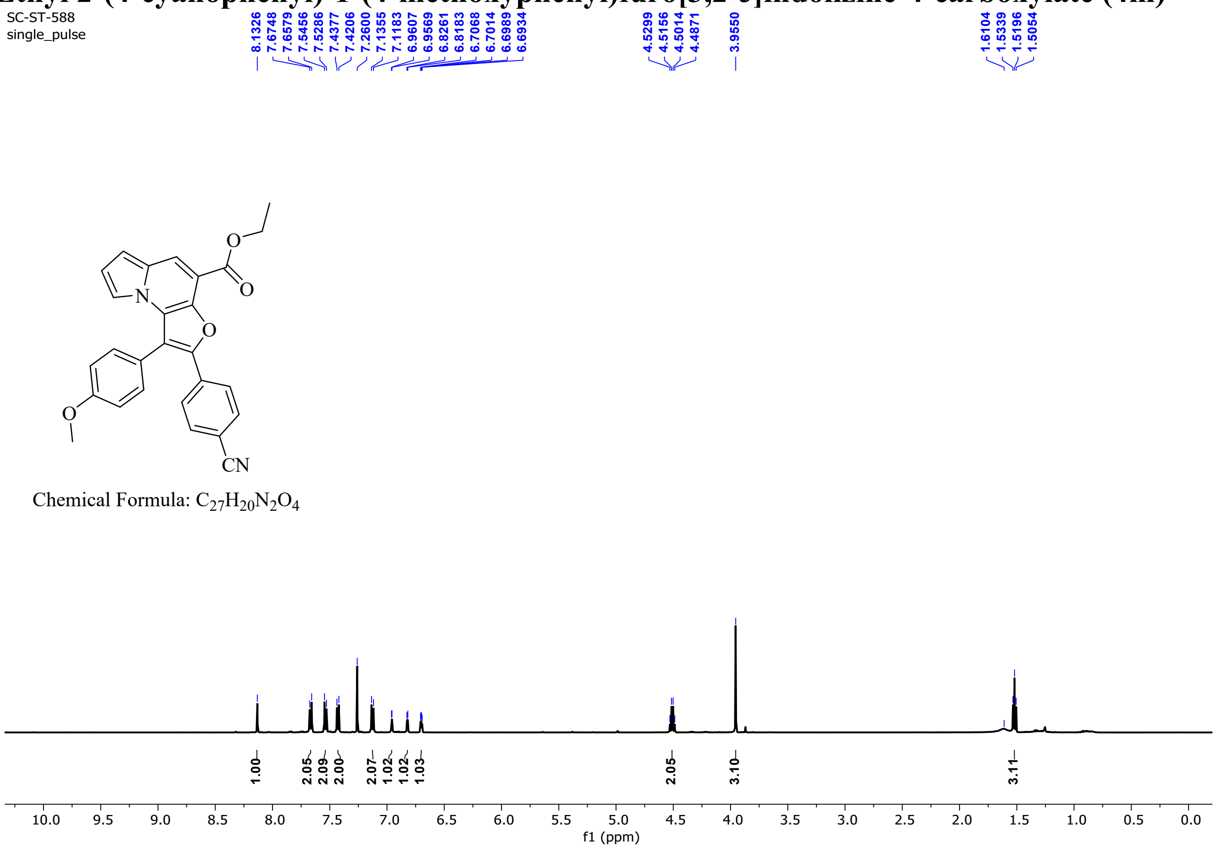

Figure S27. ^{1}H NMR Spectrum of Ethyl 2-(4-cyanophenyl)-1-(4-methoxyphenyl)furo[3,2-*e*]indolizine-4-carboxylate (4m)

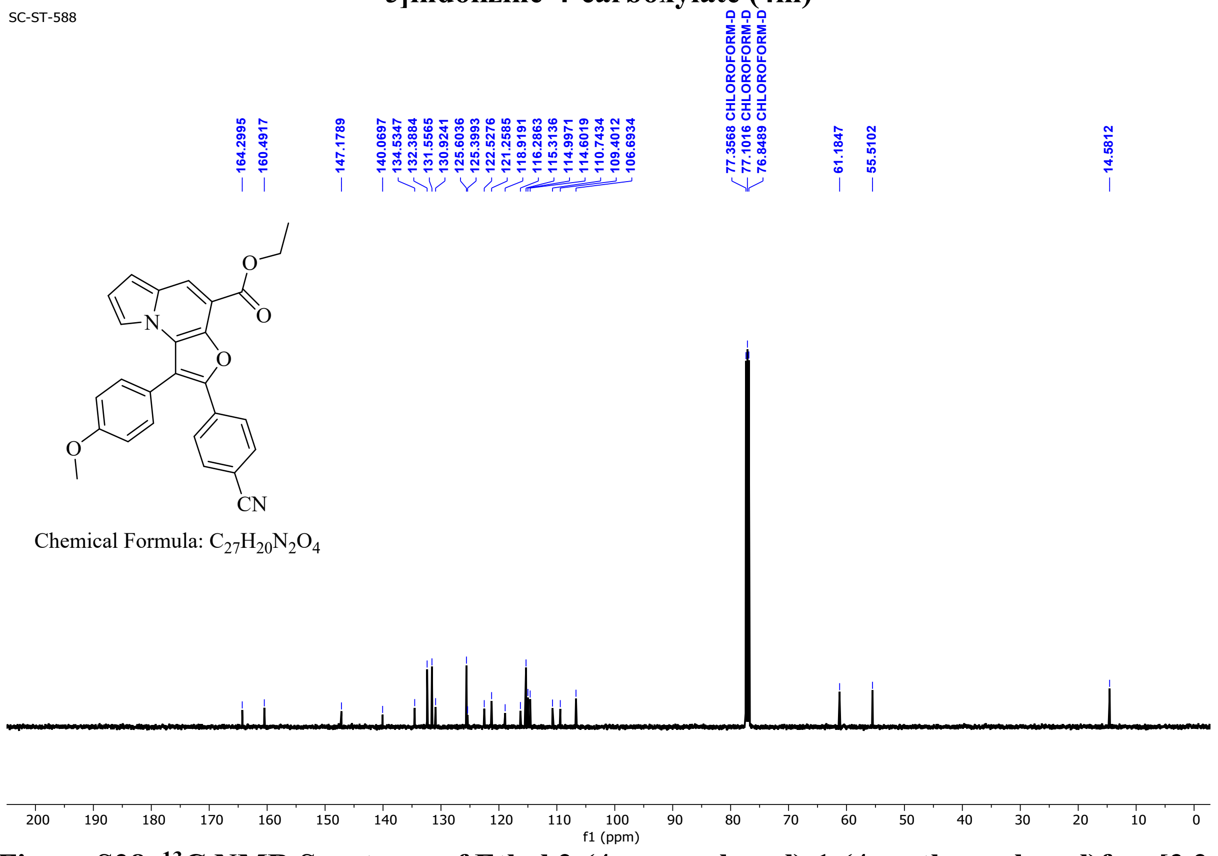

Figure S28. ^{13}C NMR Spectrum of Ethyl 2-(4-cyanophenyl)-1-(4-methoxyphenyl)furo[3,2-*e*]indolizine-4-carboxylate (4m)

14. Ethyl 2-(4-cyanophenyl)-1-(p-tolyl)furo[3,2-*e*]indolizine-4-carboxylate (4n)

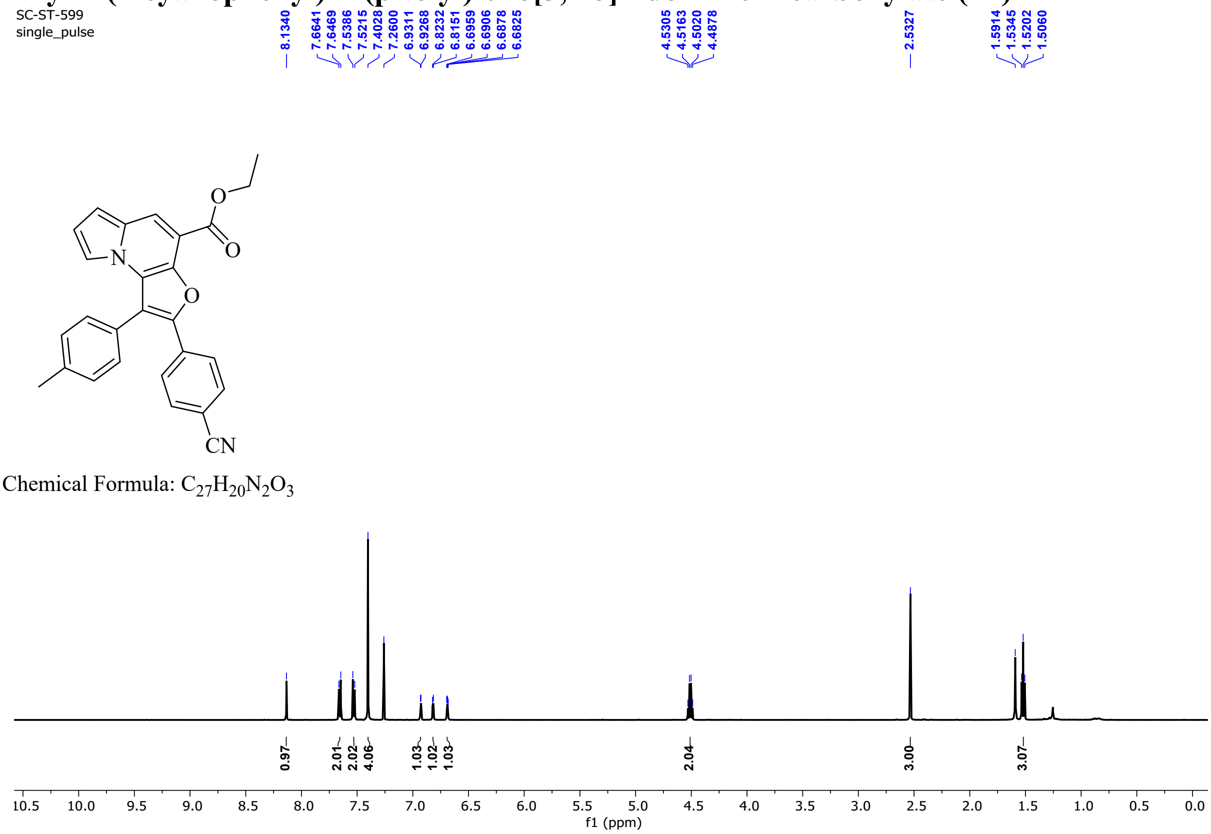

Figure S29. ^{1}H **NMR Spectrum of Ethyl 2-(4-cyanophenyl)-1-(p-tolyl)furo[3,2-*e*]indolizine-4-carboxylate (4n)**

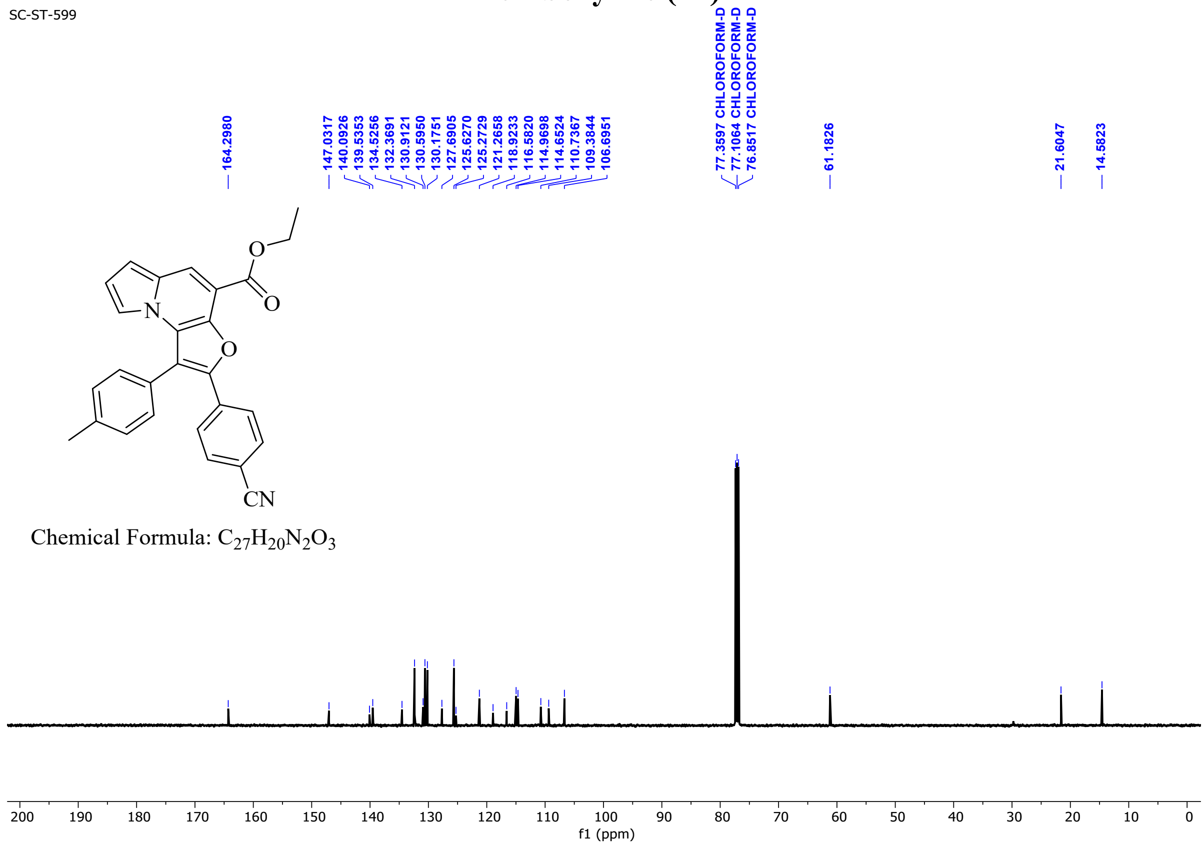

Figure S30. ^{13}C **NMR Spectrum of Ethyl 2-(4-cyanophenyl)-1-(p-tolyl)furo[3,2-*e*]indolizine-4-carboxylate (4n)**

15. Ethyl 2-(4-cyanophenyl)-1-(4-fluorophenyl)furo[3,2-*e*]indolizine-4-carboxylate (4o)

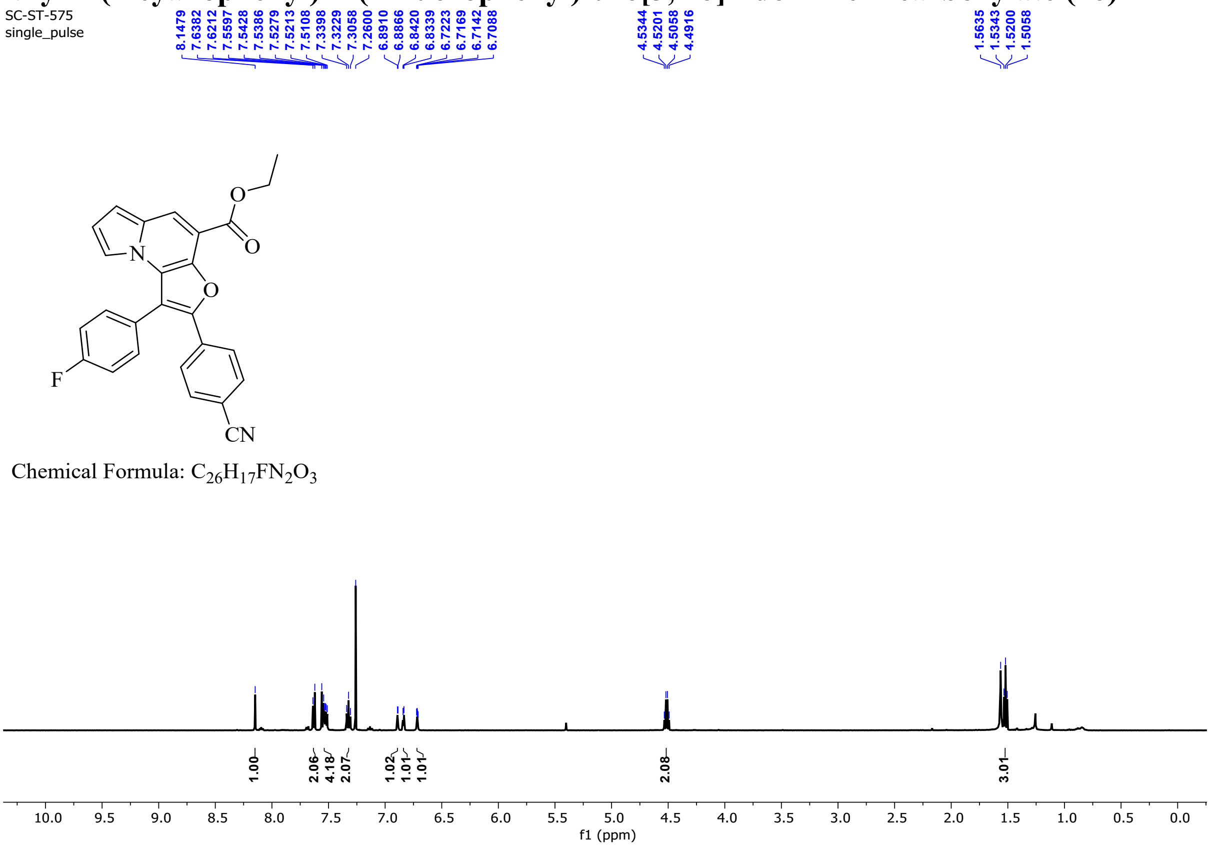

Figure S31. ^{1}**H NMR Spectrum of Ethyl 2-(4-cyanophenyl)-1-(4-fluorophenyl)furo[3,2-*e*]indolizine-4-carboxylate (4o)**

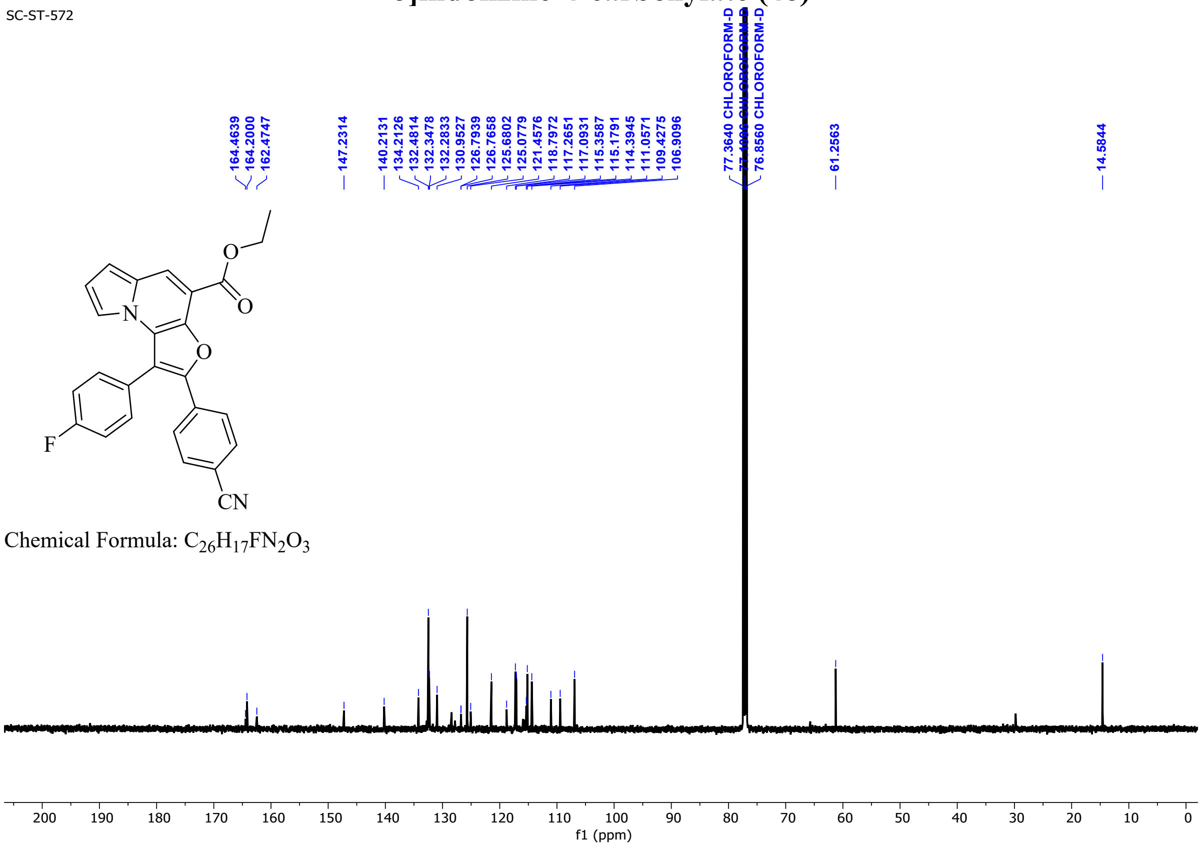

Figure S32. ^{13}**C NMR Spectrum of Ethyl 2-(4-cyanophenyl)-1-(4-fluorophenyl)furo[3,2-*e*]indolizine-4-carboxylate (4o)**

16. Ethyl 1-(4-chlorophenyl)-2-(4-cyanophenyl)furo[3,2-*e*]indolizine-4-carboxylate (4p)

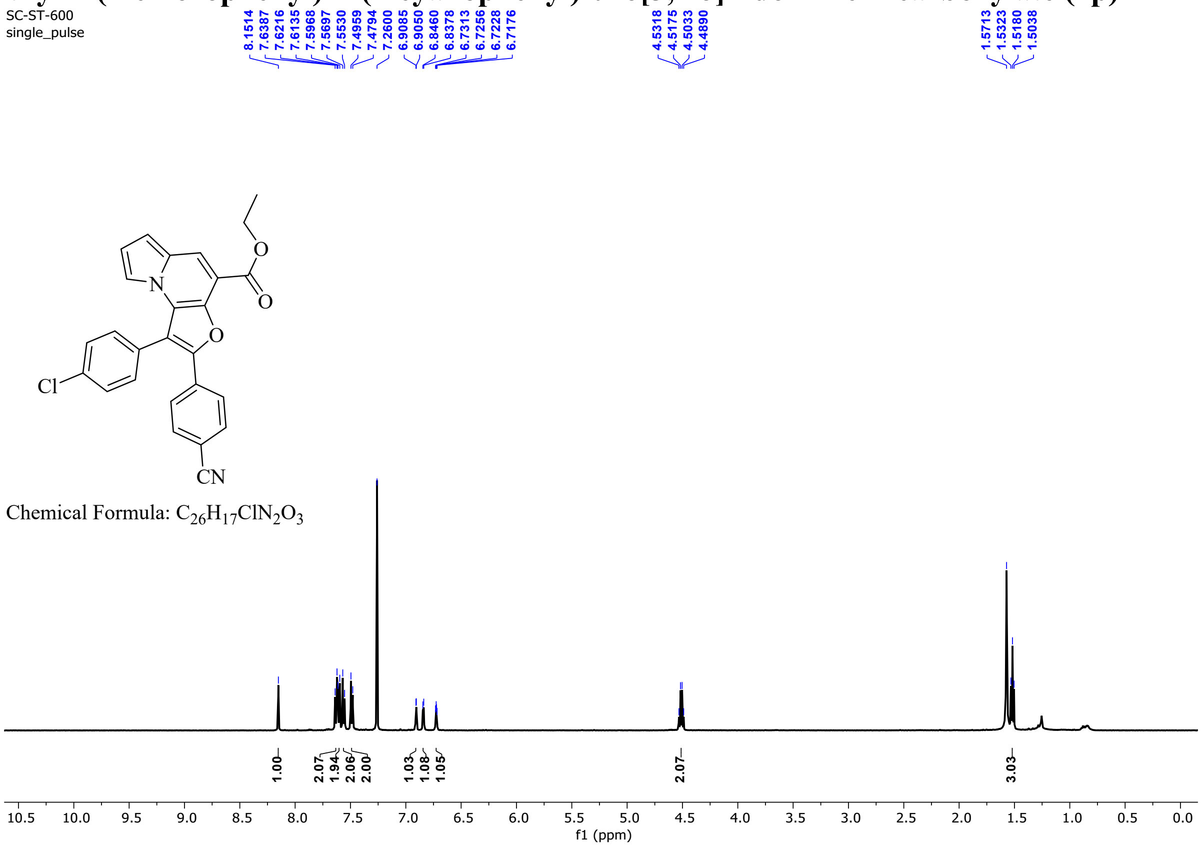

Figure S33. ^{1}H NMR Spectrum of Ethyl 1-(4-chlorophenyl)-2-(4-cyanophenyl)furo[3,2-*e*]indolizine-4-carboxylate (4p)

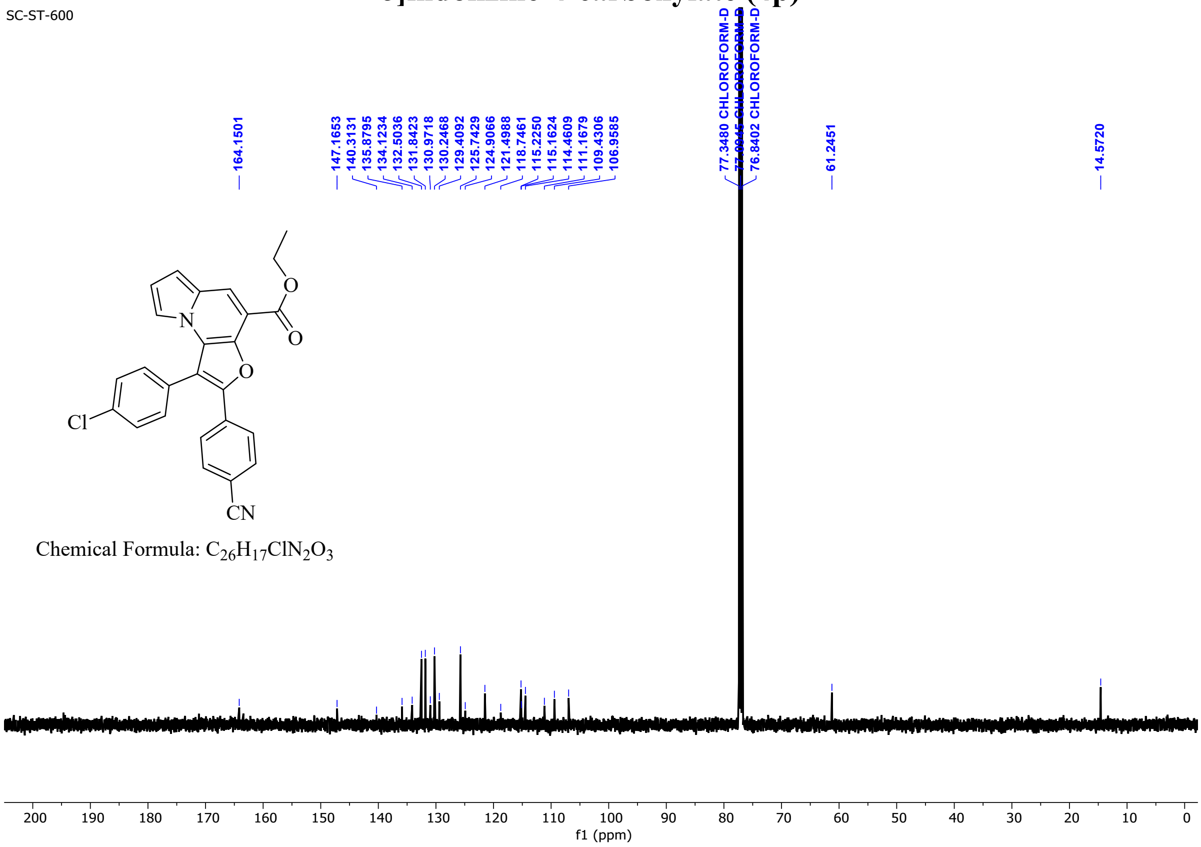

Figure S34. ^{13}C NMR Spectrum of Ethyl 1-(4-chlorophenyl)-2-(4-cyanophenyl)furo[3,2-*e*]indolizine-4-carboxylate (4p)

17. Ethyl 1-(4-bromophenyl)-2-(4-cyanophenyl)furo[3,2-*e*]indolizine-4-carboxylate (4q)

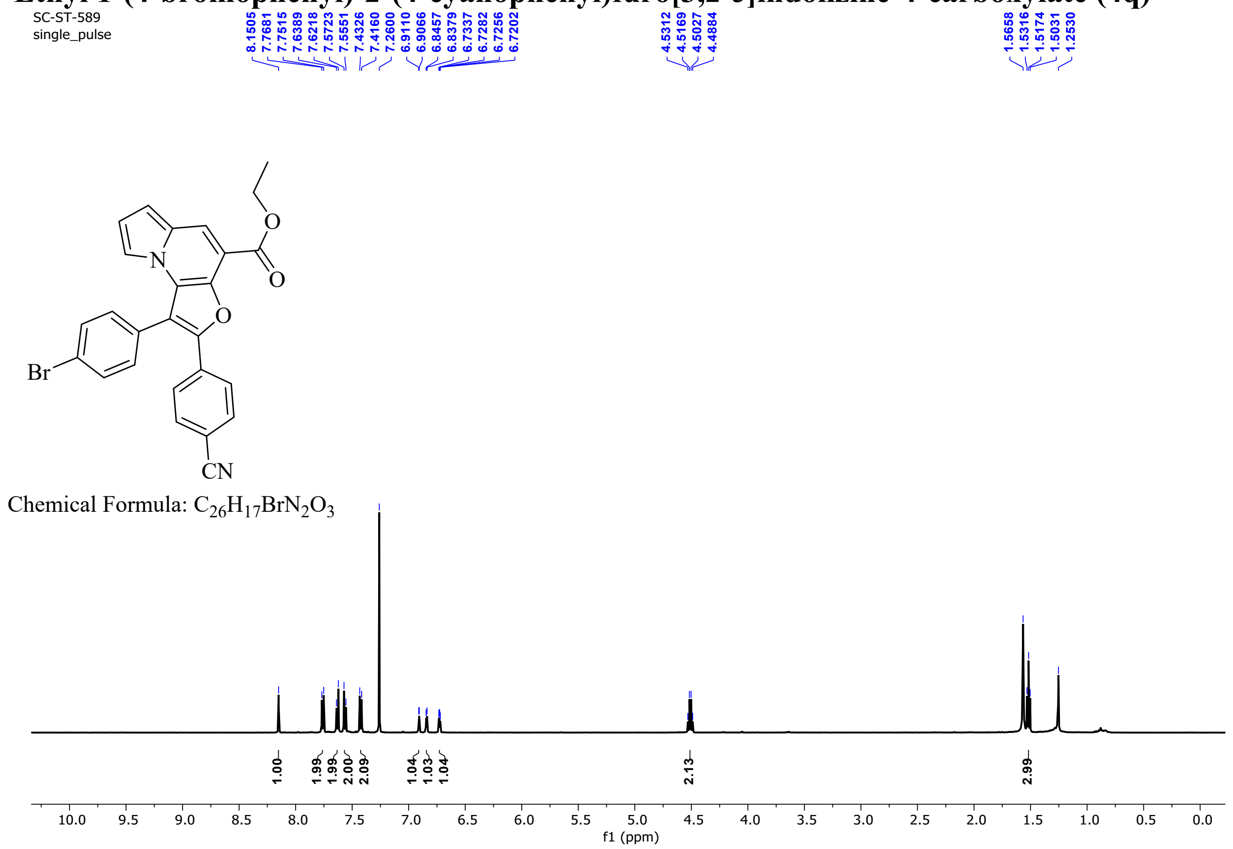

Figure S35. ^{1}H NMR Spectrum of Ethyl 1-(4-bromophenyl)-2-(4-cyanophenyl)furo[3,2-*e*]indolizine-4-carboxylate (4q)

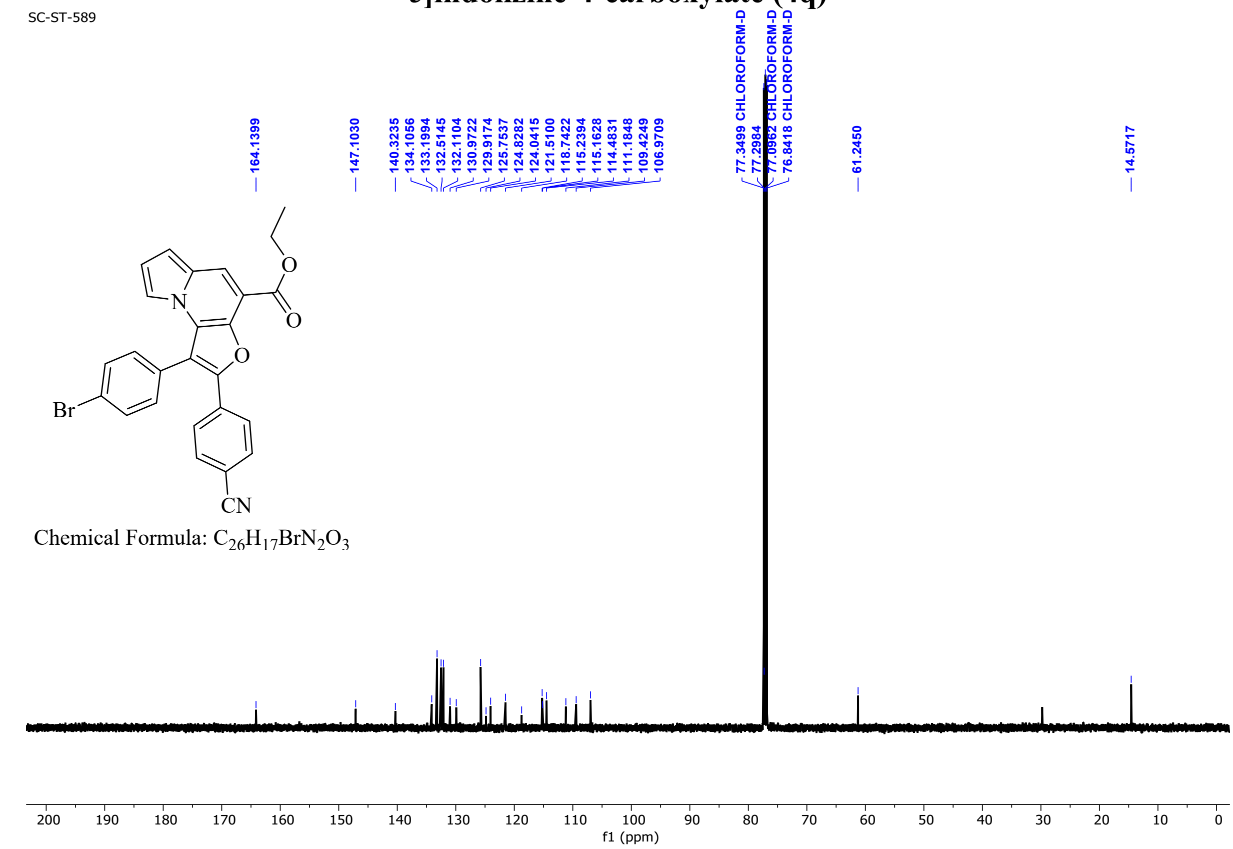

Figure S36. ^{13}C NMR Spectrum of Ethyl 1-(4-bromophenyl)-2-(4-cyanophenyl)furo[3,2-*e*]indolizine-4-carboxylate (4q)

18. Ethyl 2-(4-cyanophenyl)-1-(3-methoxyphenyl)furo[3,2-*e*]indolizine-4-carboxylate (4r)

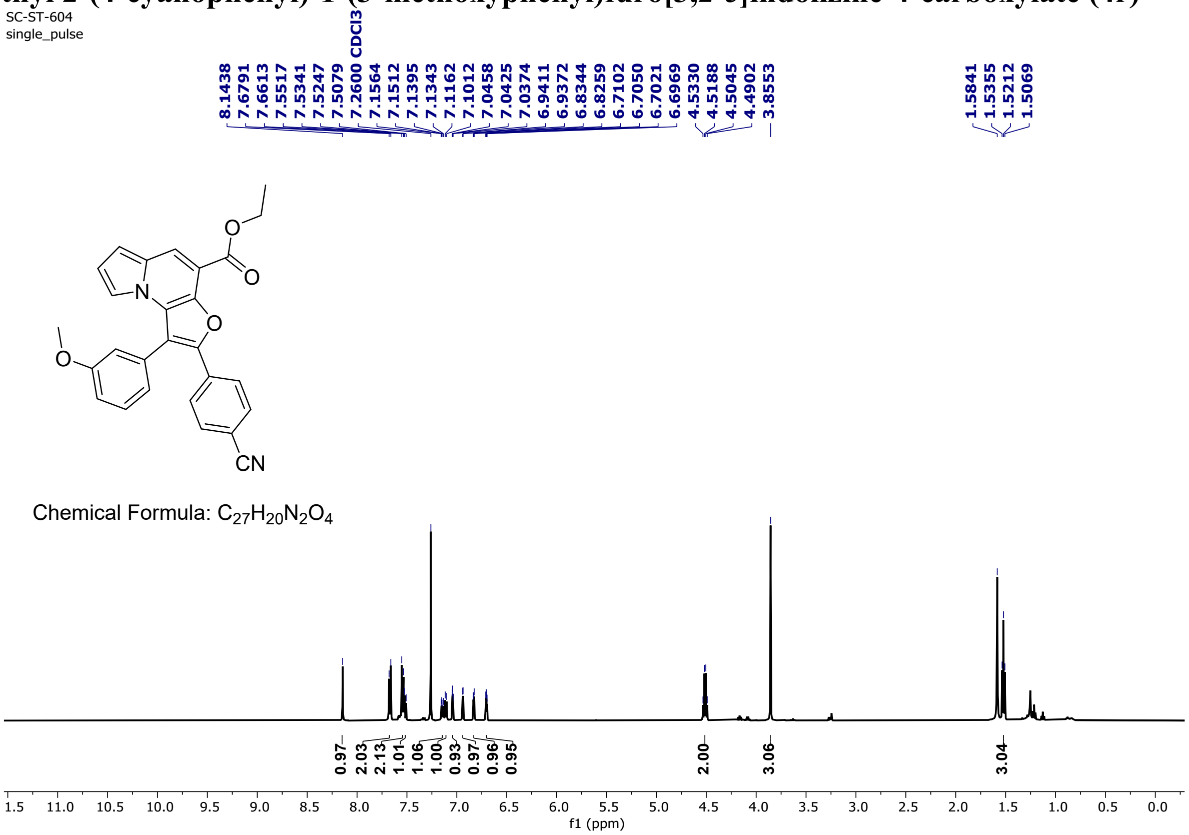

Figure S37. ¹H NMR Spectrum of Ethyl 2-(4-cyanophenyl)-1-(3-methoxyphenyl)furo[3,2-*e*]indolizine-4-carboxylate (4r)

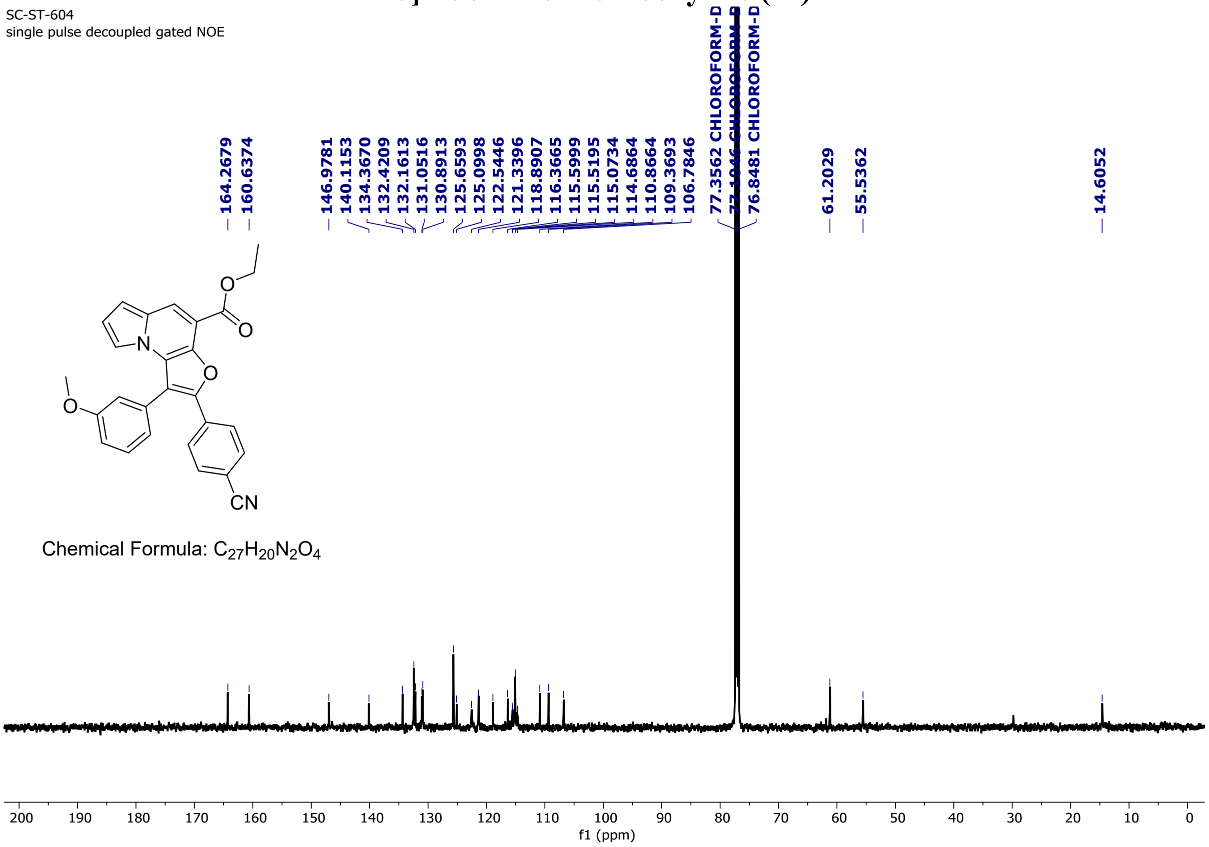

Figure S38. ¹³C NMR Spectrum of Ethyl 2-(4-cyanophenyl)-1-(3-methoxyphenyl)furo[3,2-*e*]indolizine-4-carboxylate (4r)

19. Ethyl 2-(4-cyanophenyl)-1-(4-(trifluoromethyl)phenyl)furo[3,2-*e*]indolizine-4-carboxylate (4s)

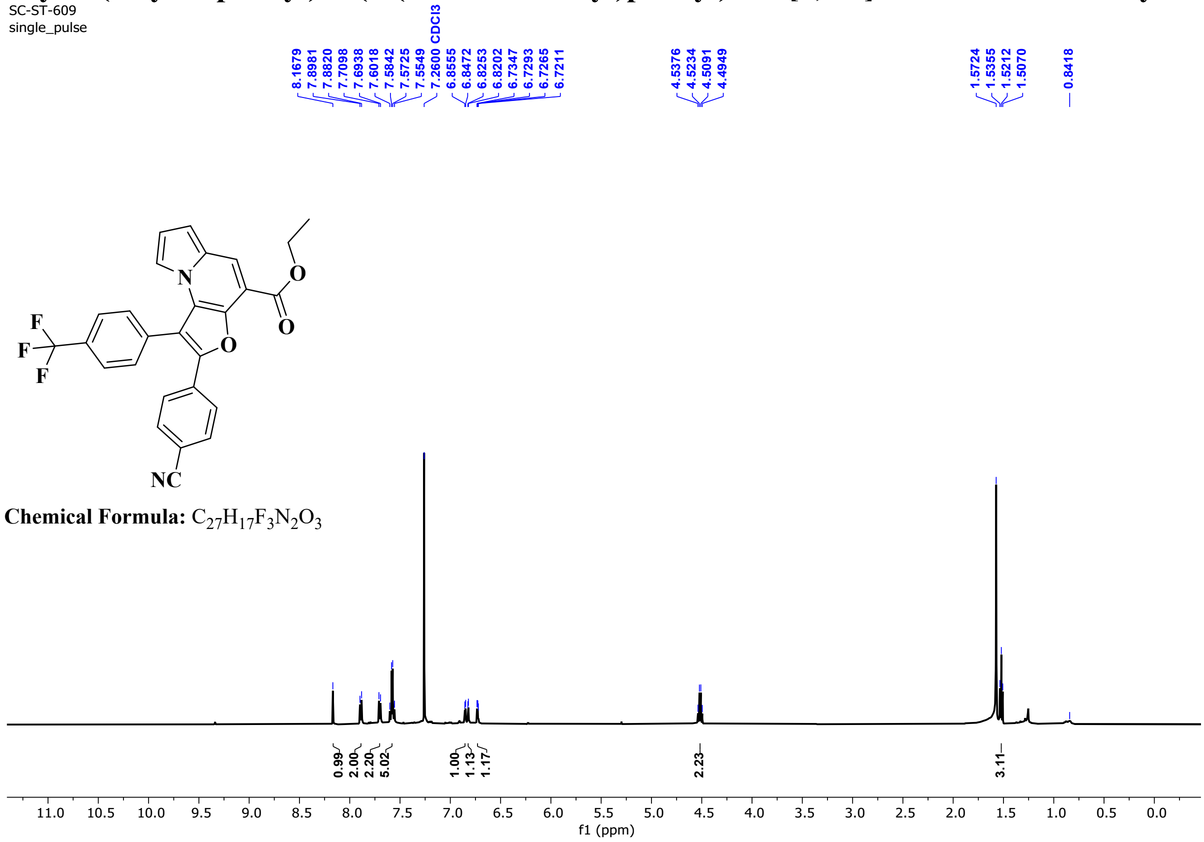

Figure S39. ^{1}H **NMR Spectrum of Ethyl 2-(4-cyanophenyl)-1-(4-(trifluoromethyl)phenyl)furo[3,2-*e*]indolizine-4-carboxylate (4s)**

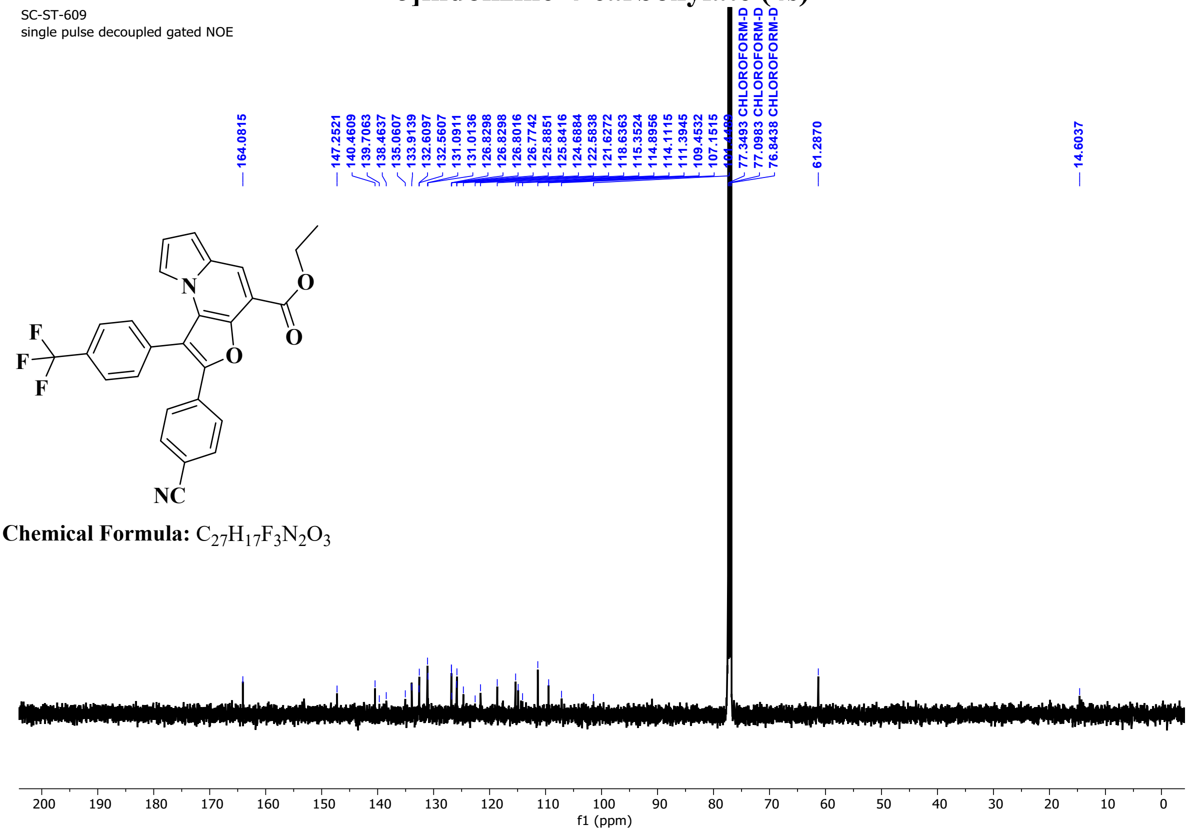

Figure S40. ^{13}C **NMR Spectrum of Ethyl 2-(4-cyanophenyl)-1-(4-(trifluoromethyl)phenyl)furo[3,2-*e*]indolizine-4-carboxylate (4s)**

20. Ethyl 2-(4-cyanophenyl)-1-(4-(trifluoromethoxy)phenyl)furo[3,2-*e*]indolizine-4-carboxylate (4t)

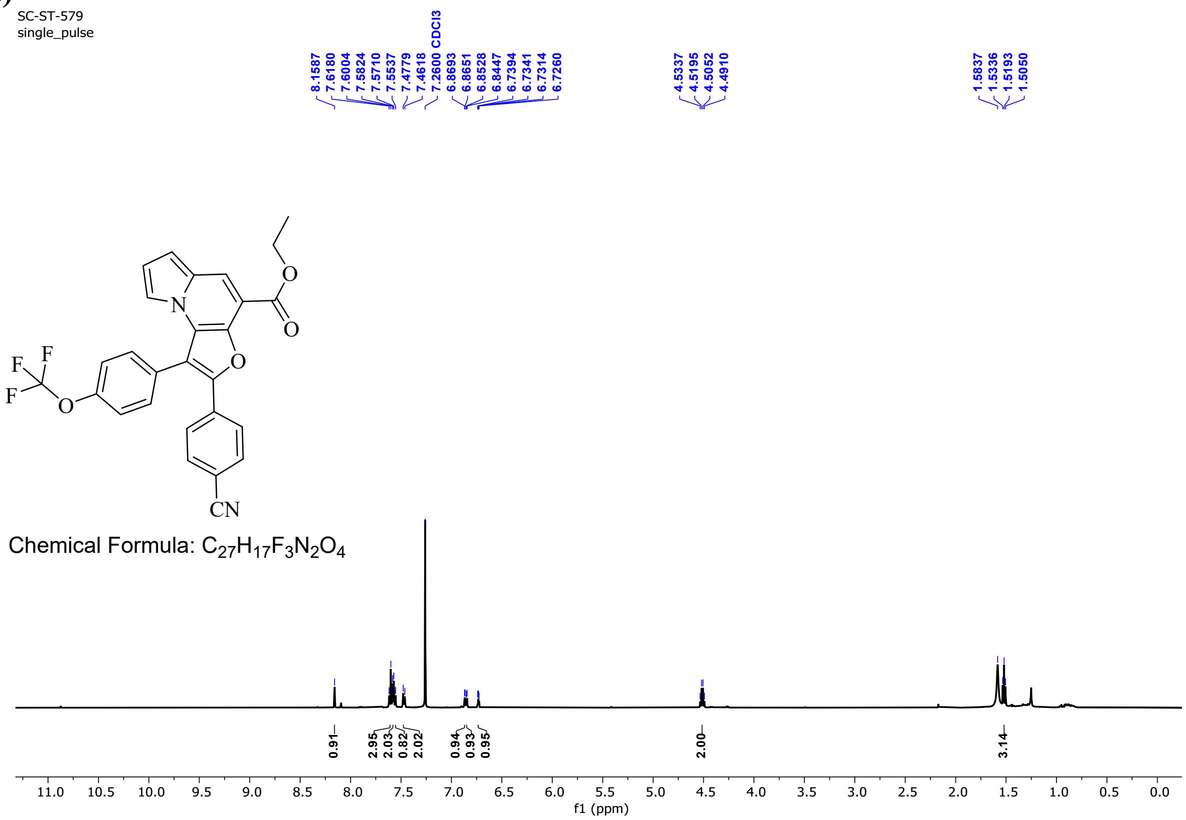

Figure S41. ^{1}H **NMR Spectrum of Ethyl 2-(4-cyanophenyl)-1-(4-(trifluoromethoxy)phenyl)furo[3,2-*e*]indolizine-4-carboxylate (4t)**

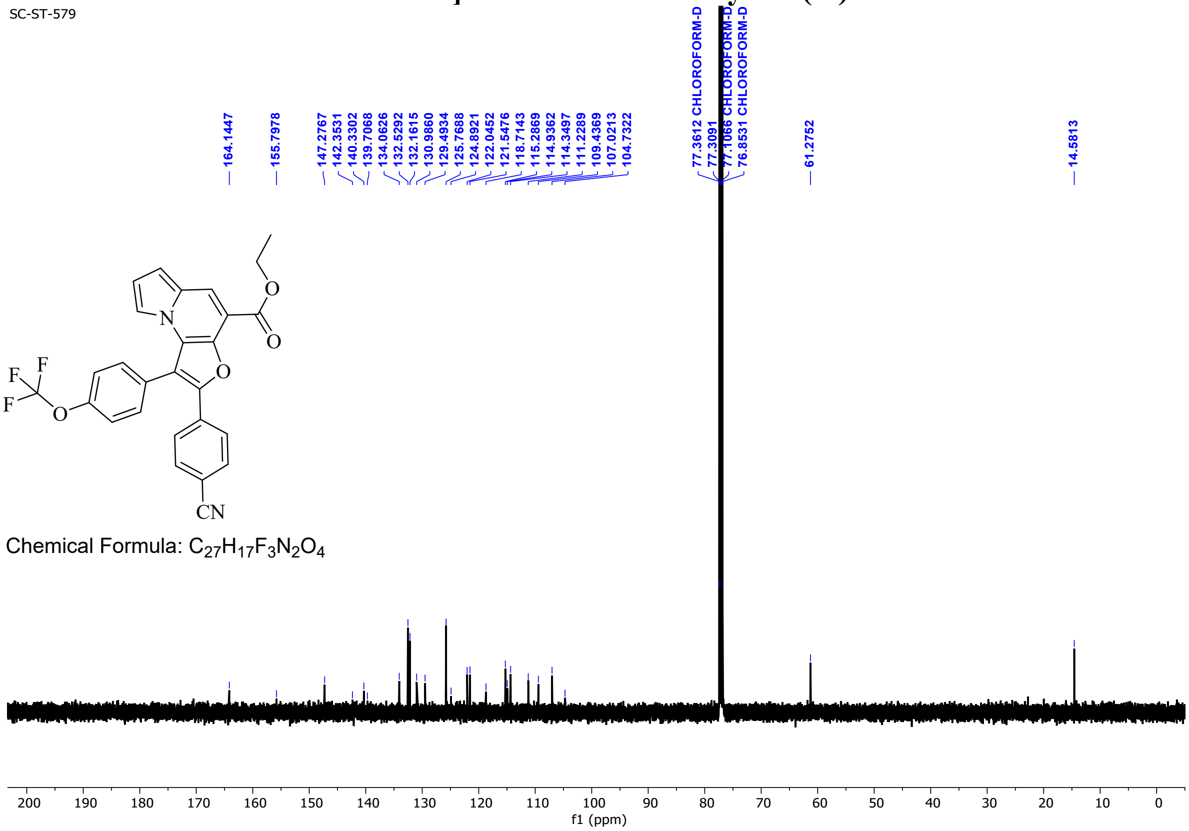

Figure S42. ^{13}C **NMR Spectrum of Ethyl 2-(4-cyanophenyl)-1-(4-(trifluoromethoxy)phenyl)furo[3,2-*e*]indolizine-4-carboxylate (4t)**

21. Ethyl 1-([1,1'-biphenyl]-4-yl)-2-(4-cyanophenyl)furo[3,2-*e*]indolizine-4-carboxylate (4u)

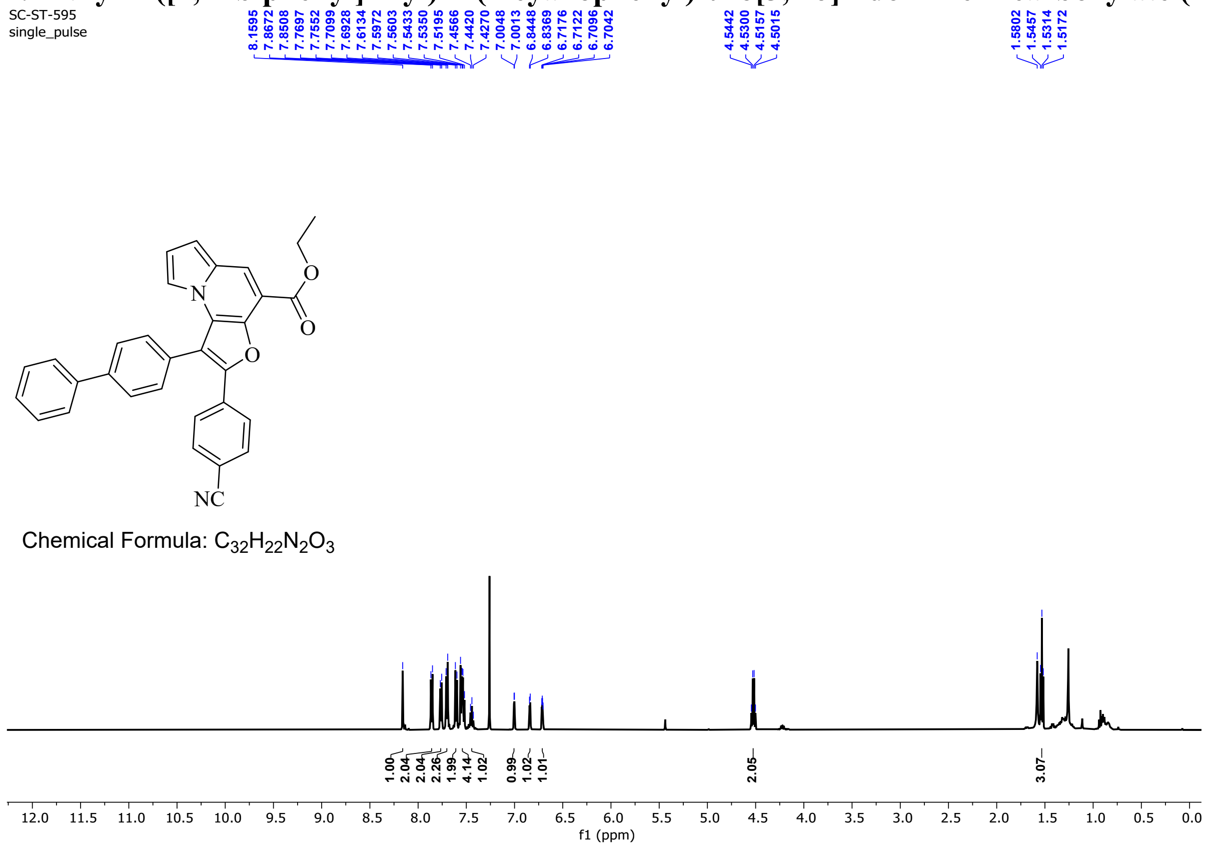

Figure S43. ^{1}H NMR Spectrum of Ethyl 1-([1,1'-biphenyl]-4-yl)-2-(4-cyanophenyl)furo[3,2-*e*]indolizine-4-carboxylate (4u)

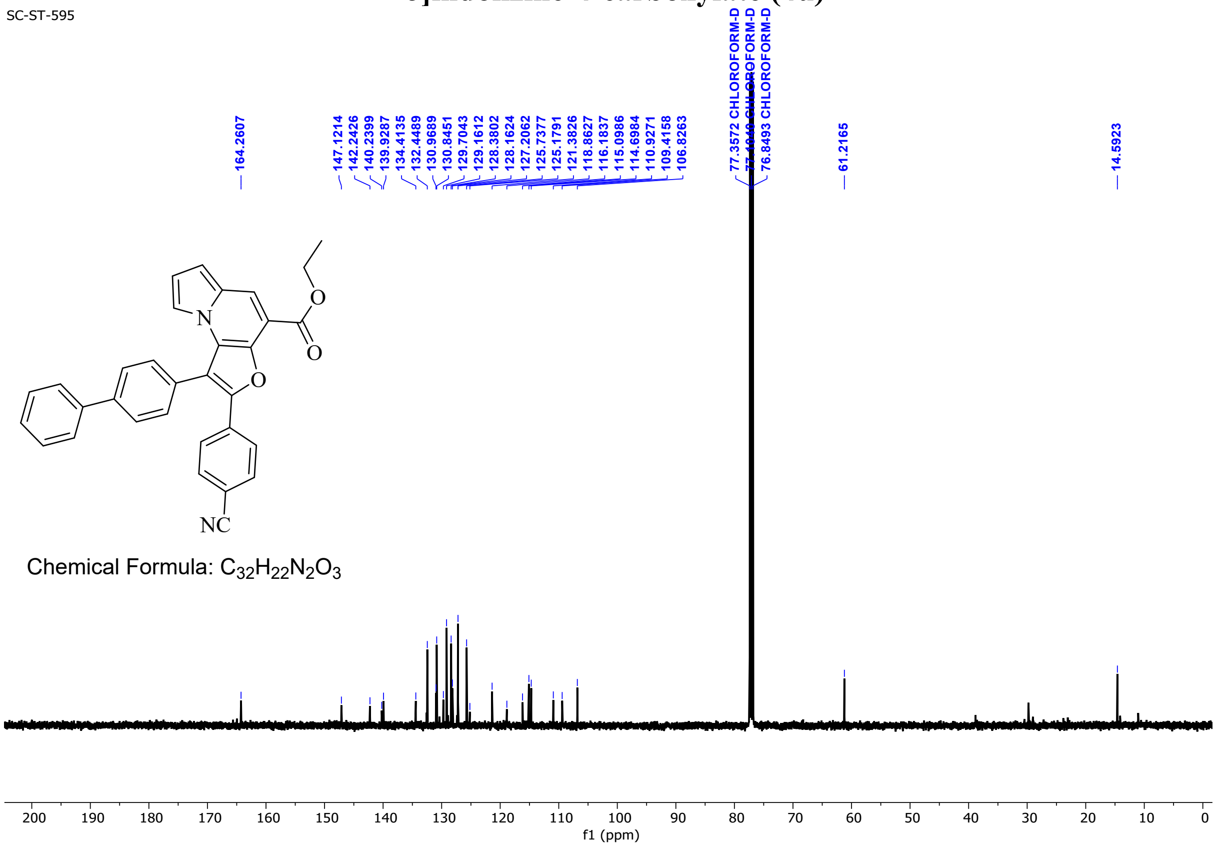

Figure S44. ^{13}C NMR Spectrum of Ethyl 1-([1,1'-biphenyl]-4-yl)-2-(4-cyanophenyl)furo[3,2-*e*]indolizine-4-carboxylate (4u)

22. Ethyl 2-(4-cyanophenyl)-1-(naphthalen-1-yl)furo[3,2-*e*]indolizine-4-carboxylate (4v)

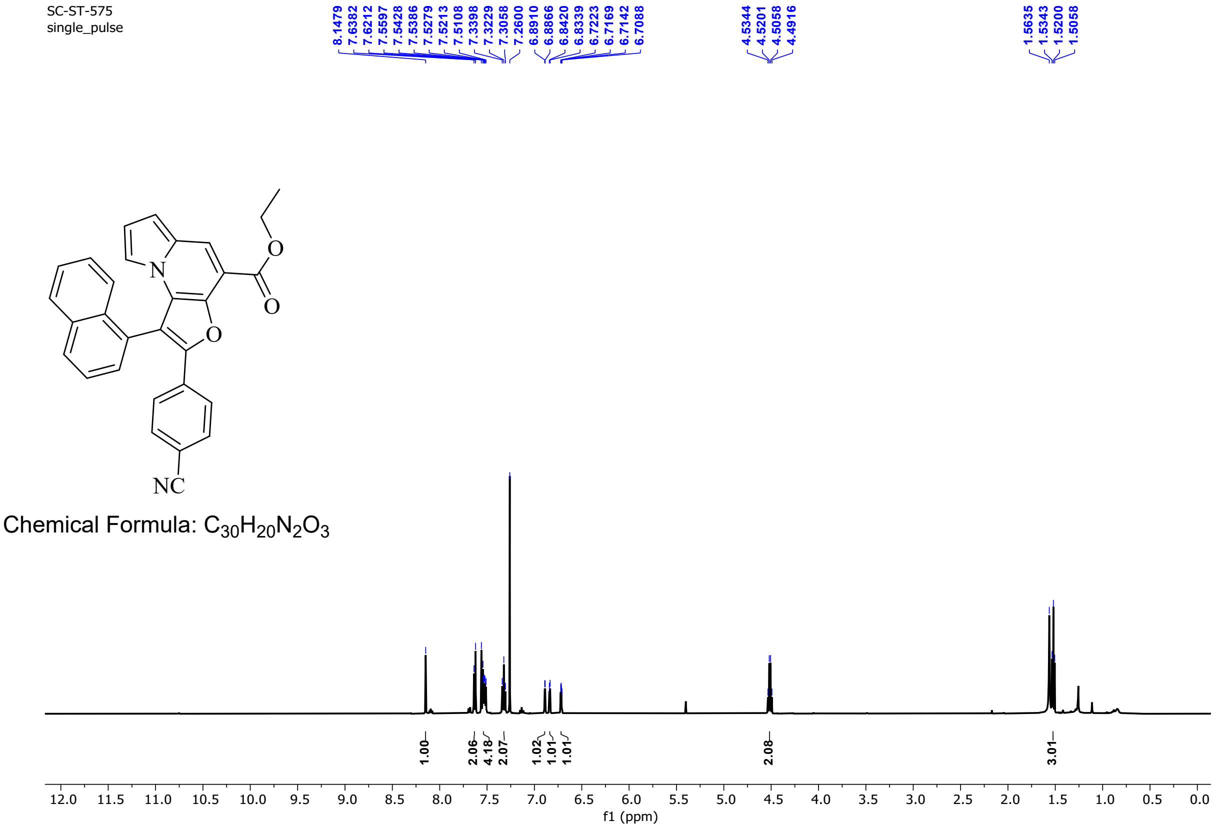

Figure S45. ^{1}**H NMR Spectrum of Ethyl 2-(4-cyanophenyl)-1-(naphthalen-1-yl)furo[3,2-*e*]indolizine-4-carboxylate (4v)**

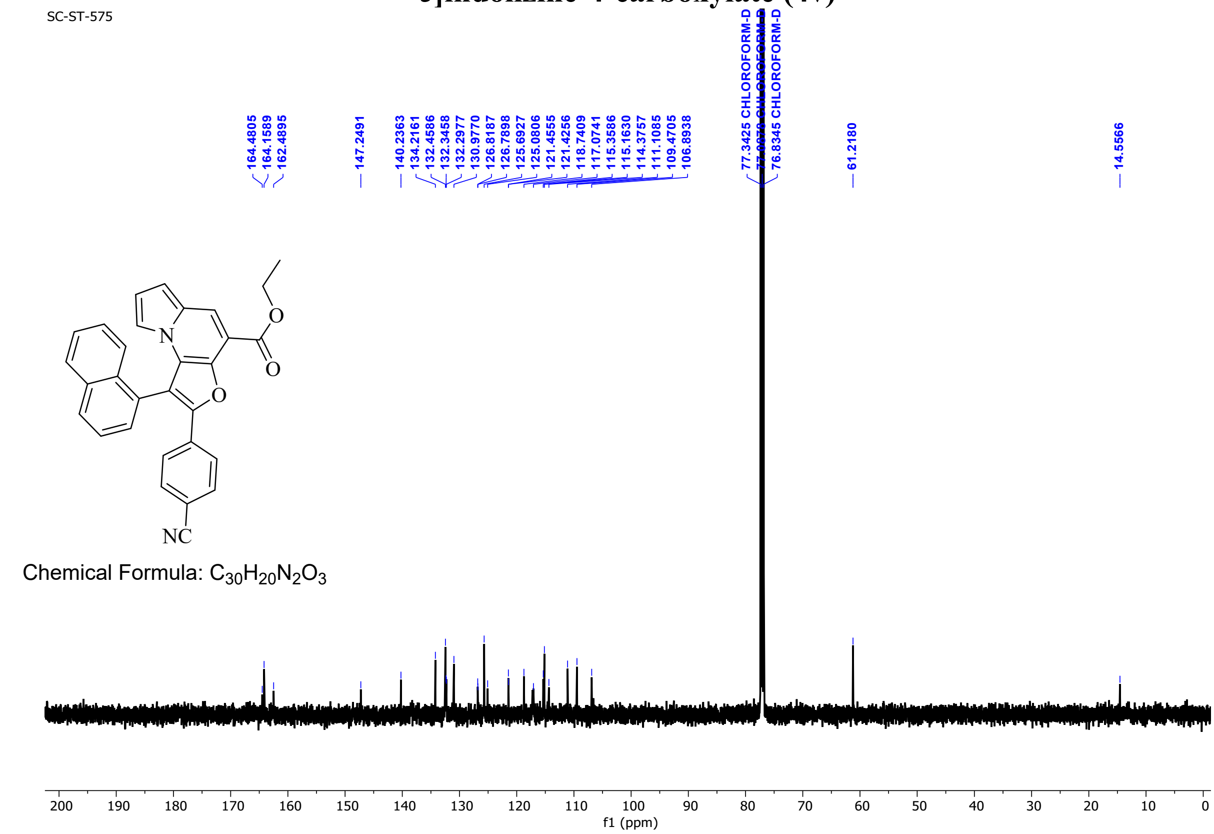

Figure S46. ^{13}**C NMR Spectrum of Ethyl 2-(4-cyanophenyl)-1-(naphthalen-1-yl)furo[3,2-*e*]indolizine-4-carboxylate (4v)**

23. Ethyl 2-(4-chlorobenzoyl)-1-phenylfuro[3,2-*e*]indolizine-4-carboxylate (6a)

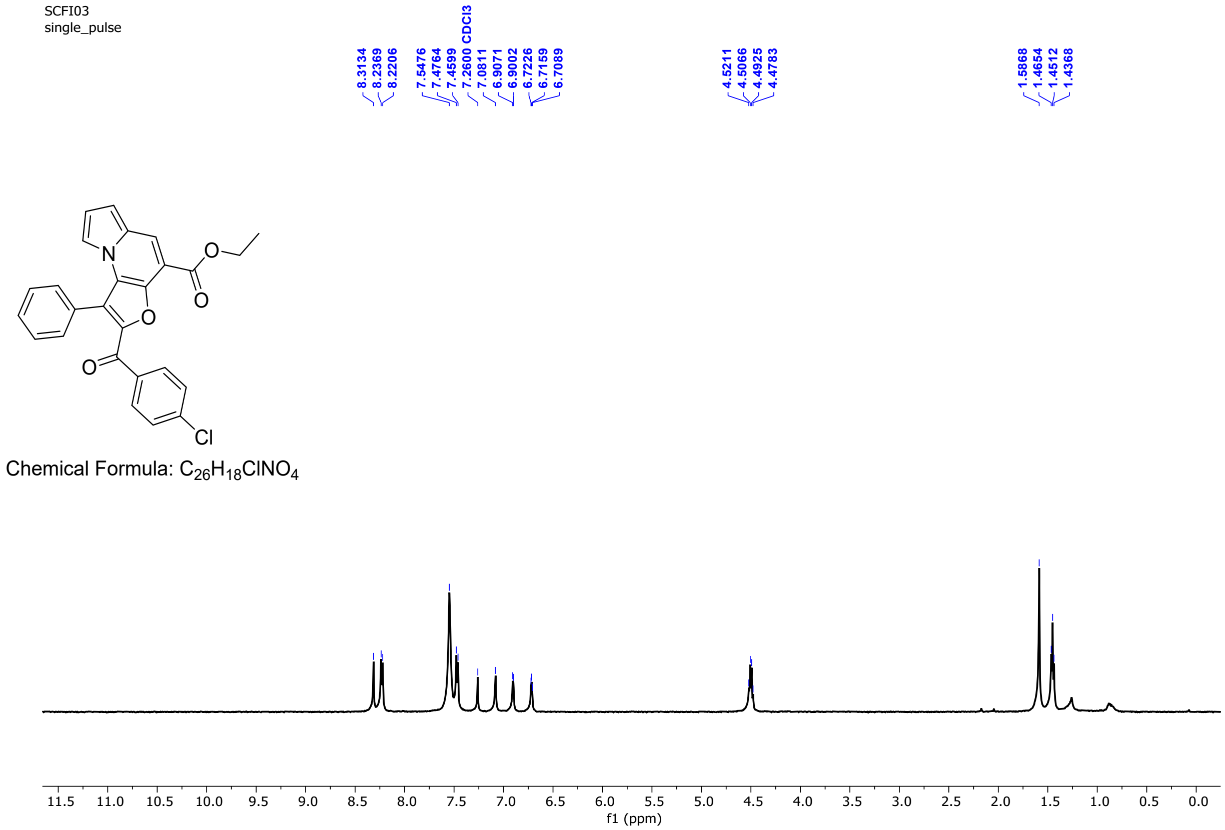

Figure S47. ^{1}H NMR Spectrum of Ethyl 2-(4-chlorobenzoyl)-1-phenylfuro[3,2-*e*]indolizine-4-carboxylate (6a)

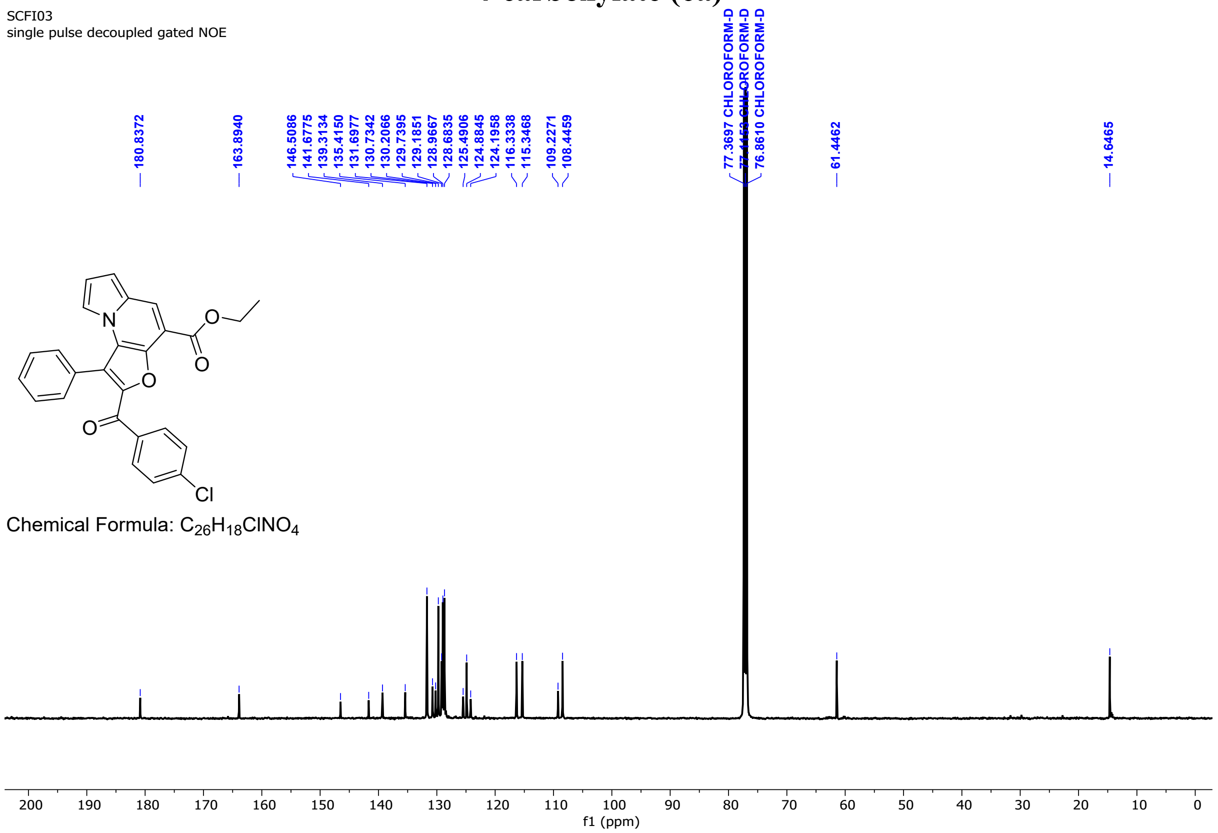

Figure S48. ^{13}C NMR Spectrum of Ethyl 2-(4-chlorobenzoyl)-1-phenylfuro[3,2-*e*]indolizine-4-carboxylate (6a)

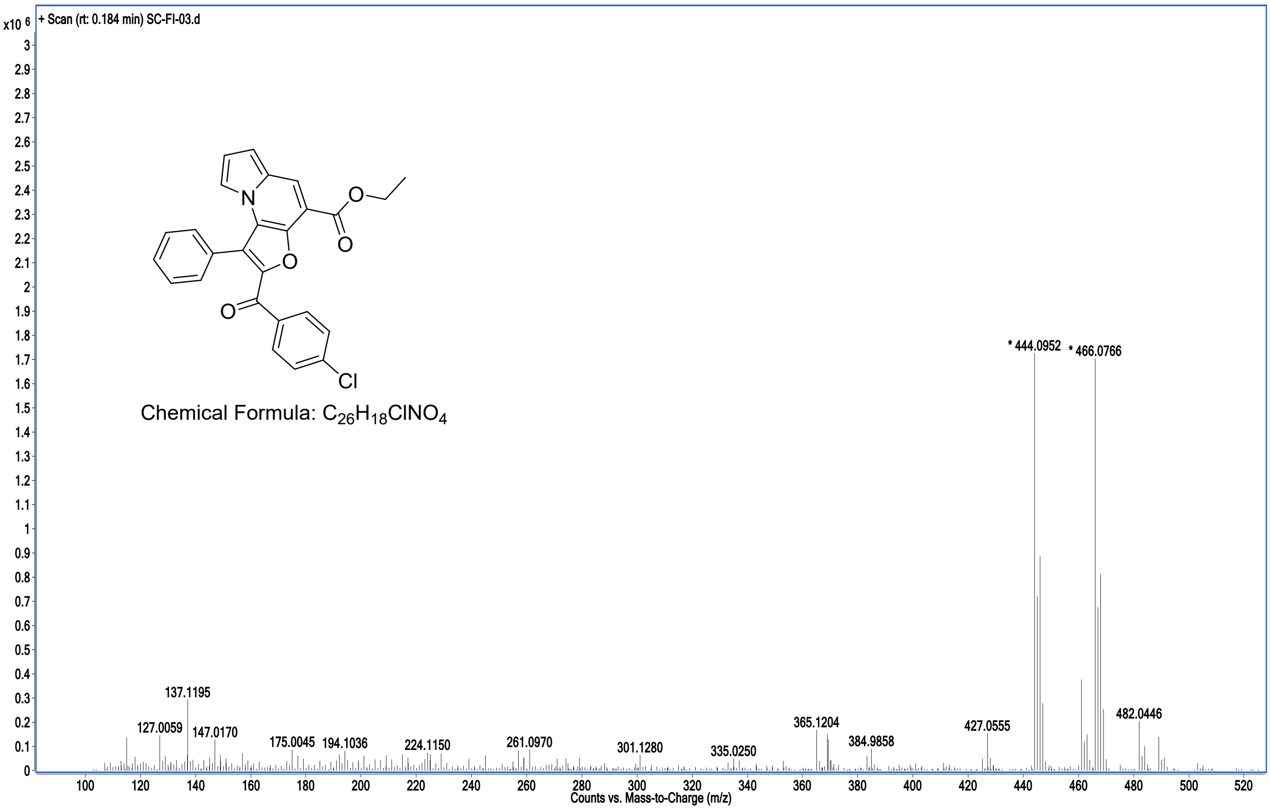

Figure S49. HRMS Spectrum of Ethyl 2-(4-chlorobenzoyl)-1-phenylfuro[3,2-*e*]indolizine-4-carboxylate (6a)

24. Ethyl 2-(4-bromobenzoyl)-1-phenylfuro[3,2-*e*]indolizine-4-carboxylate (6b)

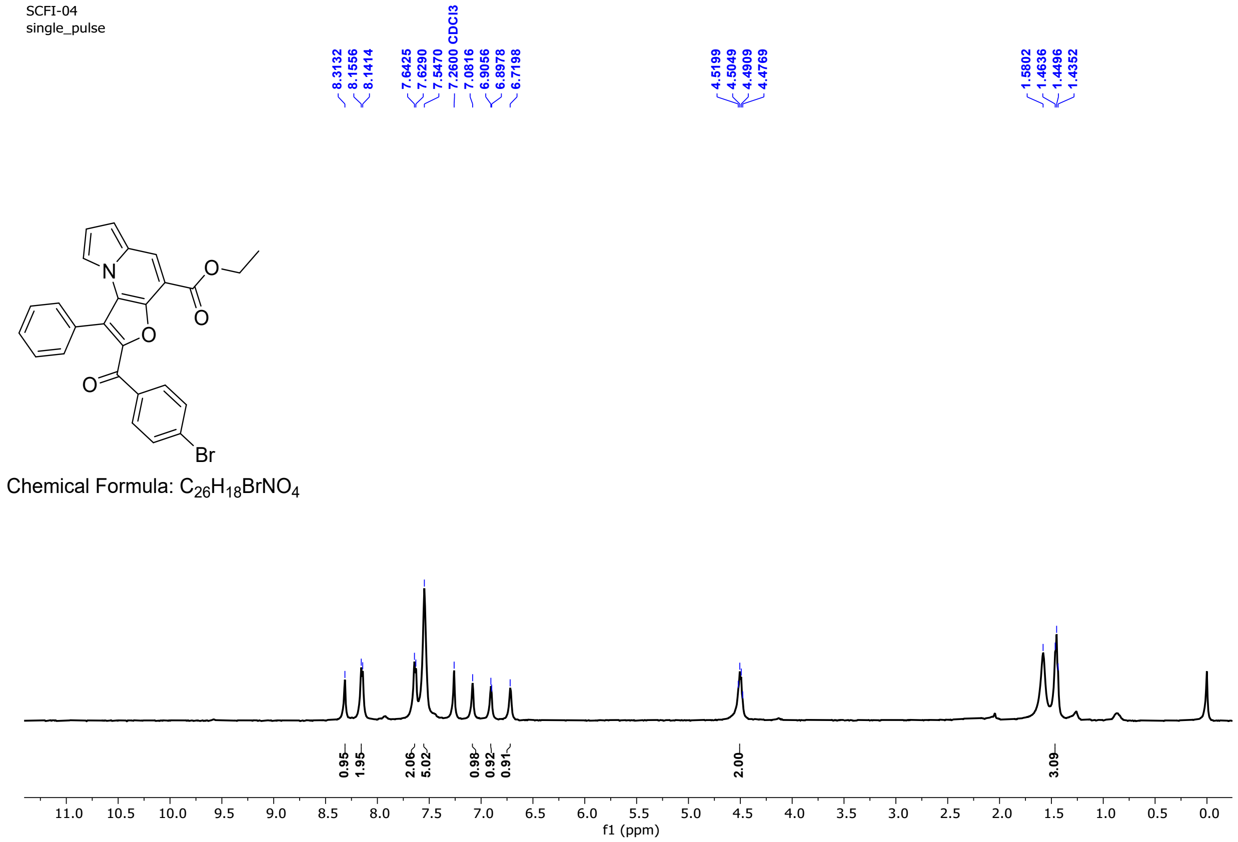

Figure S50. ^{1}H NMR Spectrum of Ethyl 2-(4-bromobenzoyl)-1-phenylfuro[3,2-*e*]indolizine-4-carboxylate (6b)

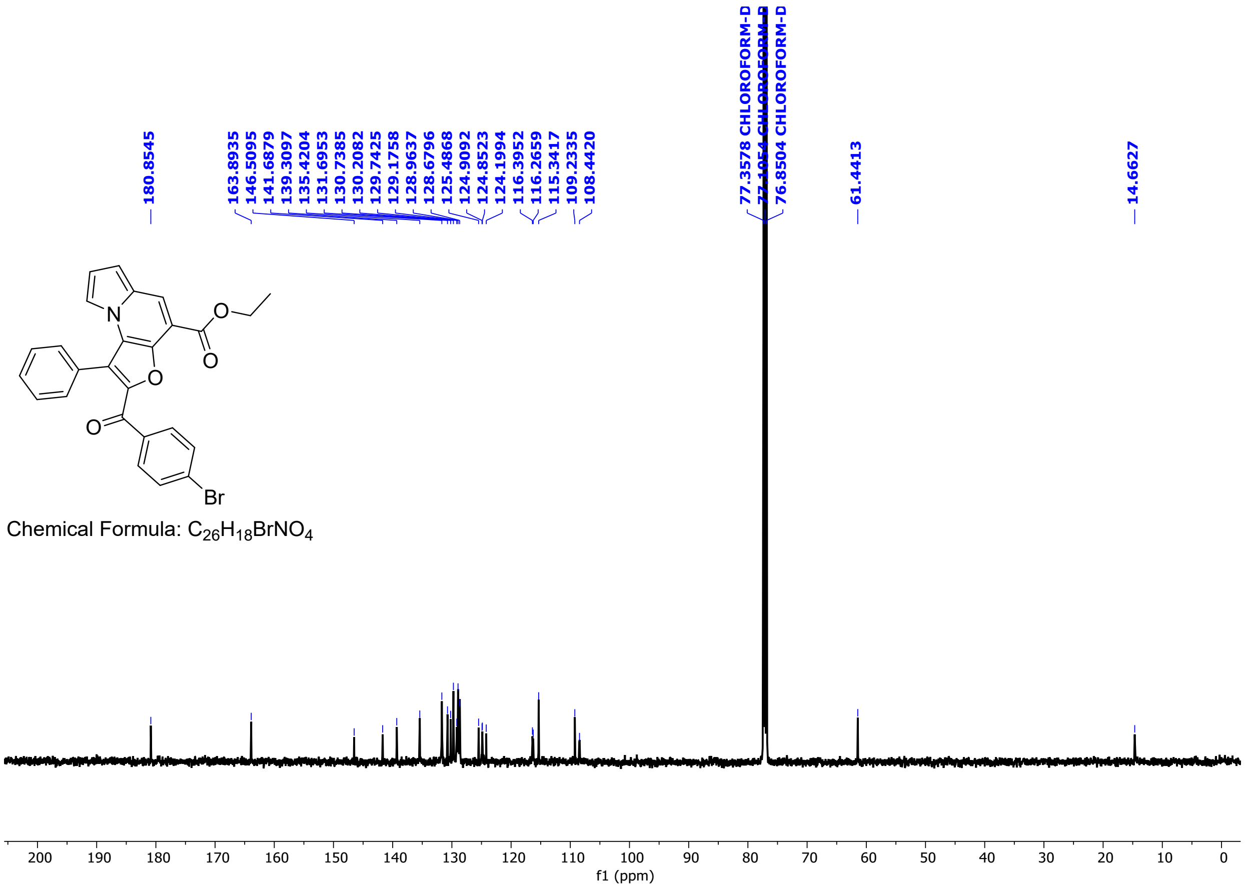

Figure S51. ^{13}**C NMR Spectrum of Ethyl 2-(4-bromobenzoyl)-1-phenylfuro[3,2-*e*]indolizine-4-carboxylate (6b)**

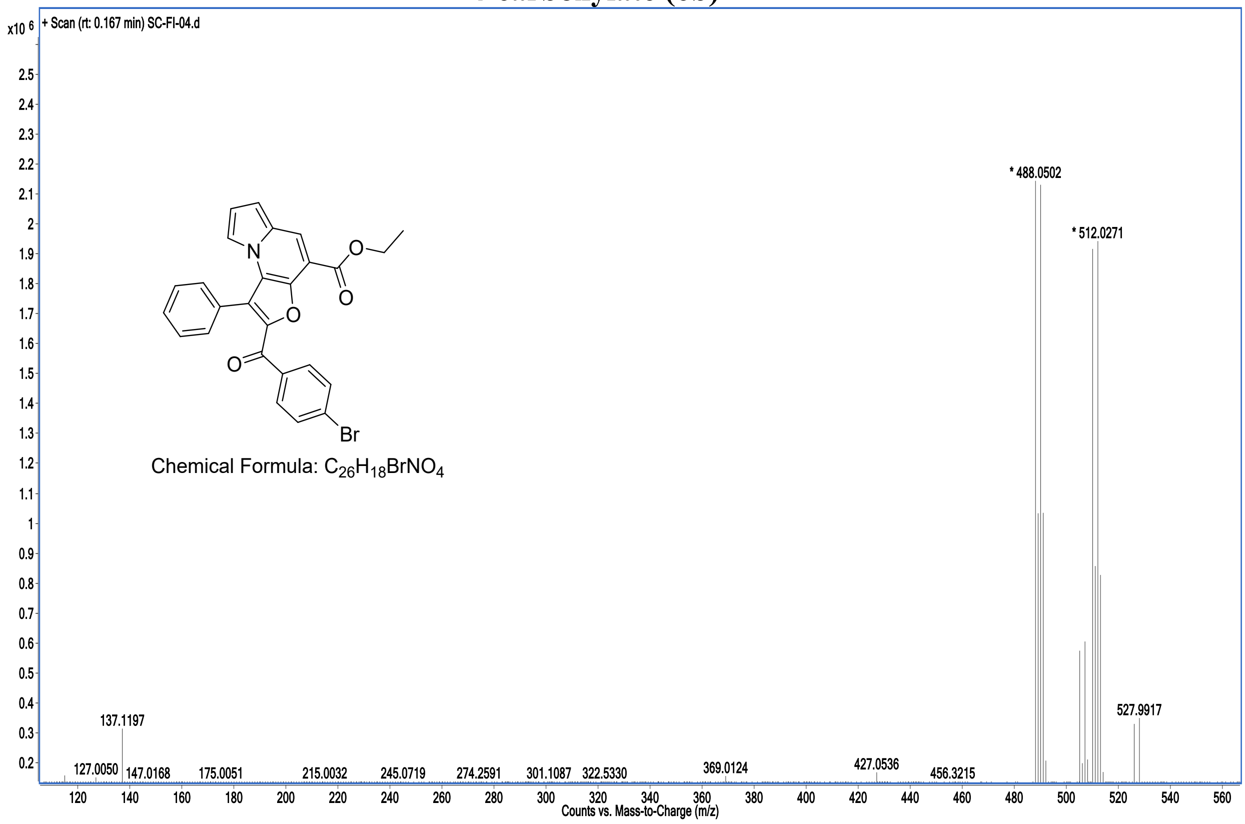

Figure S52. HRMS Spectrum of Ethyl 2-(4-bromobenzoyl)-1-phenylfuro[3,2-*e*]indolizine-4-carboxylate (6b)

25. Ethyl 2-(4-methoxybenzoyl)-1-phenylfuro[3,2-*e*]indolizine-4-carboxylate (6c)

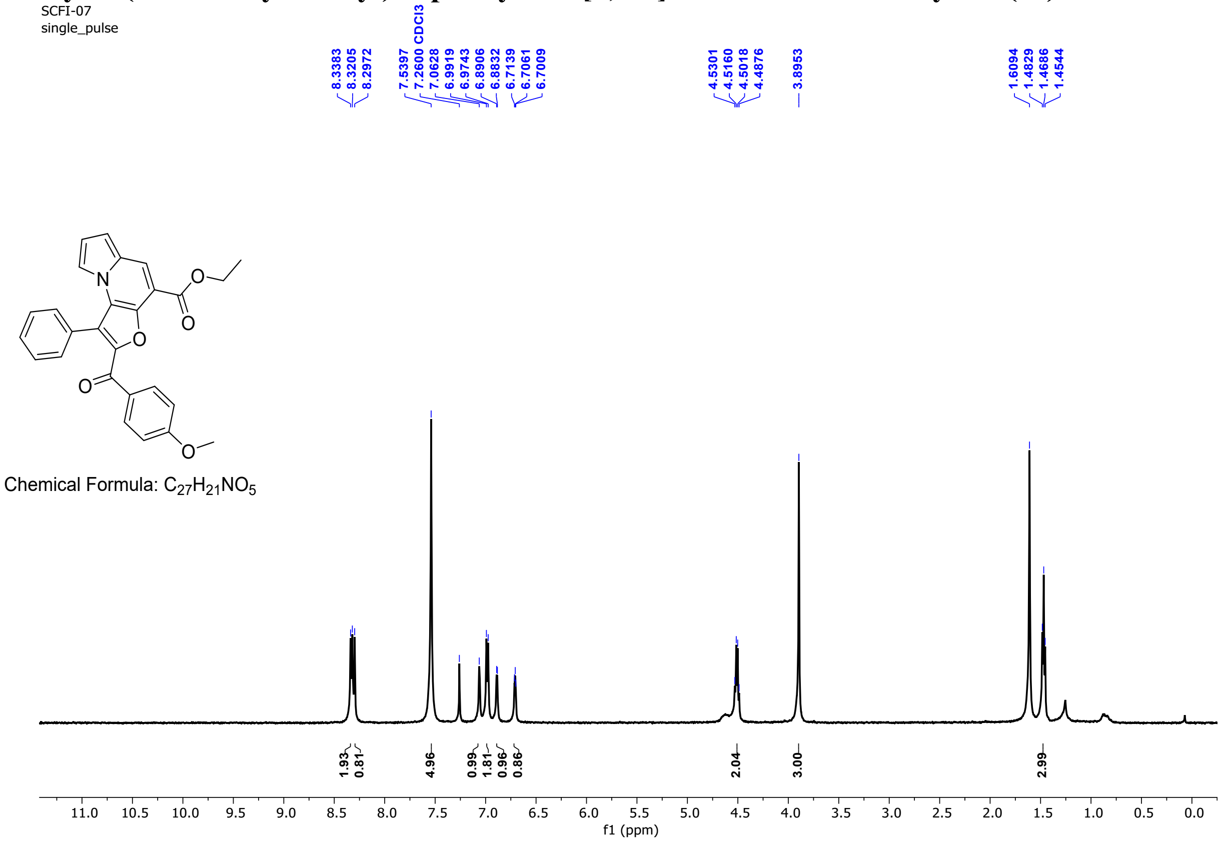

Figure S53. ^{1}H NMR Spectrum of Ethyl 2-(4-methoxybenzoyl)-1-phenylfuro[3,2-*e*]indolizine-4-carboxylate (6c)

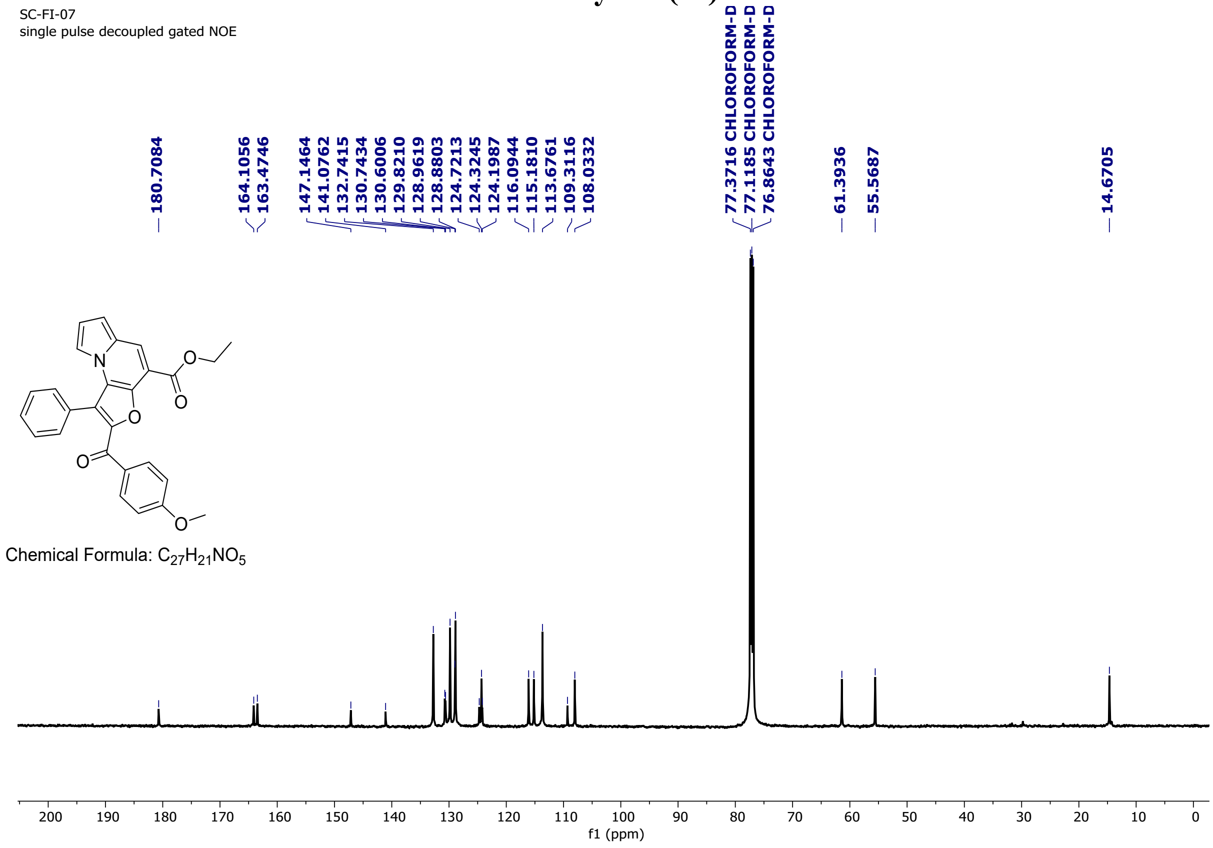

Figure S54. ^{13}C NMR Spectrum of Ethyl 2-(4-methoxybenzoyl)-1-phenylfuro[3,2-*e*]indolizine-4-carboxylate (6c)

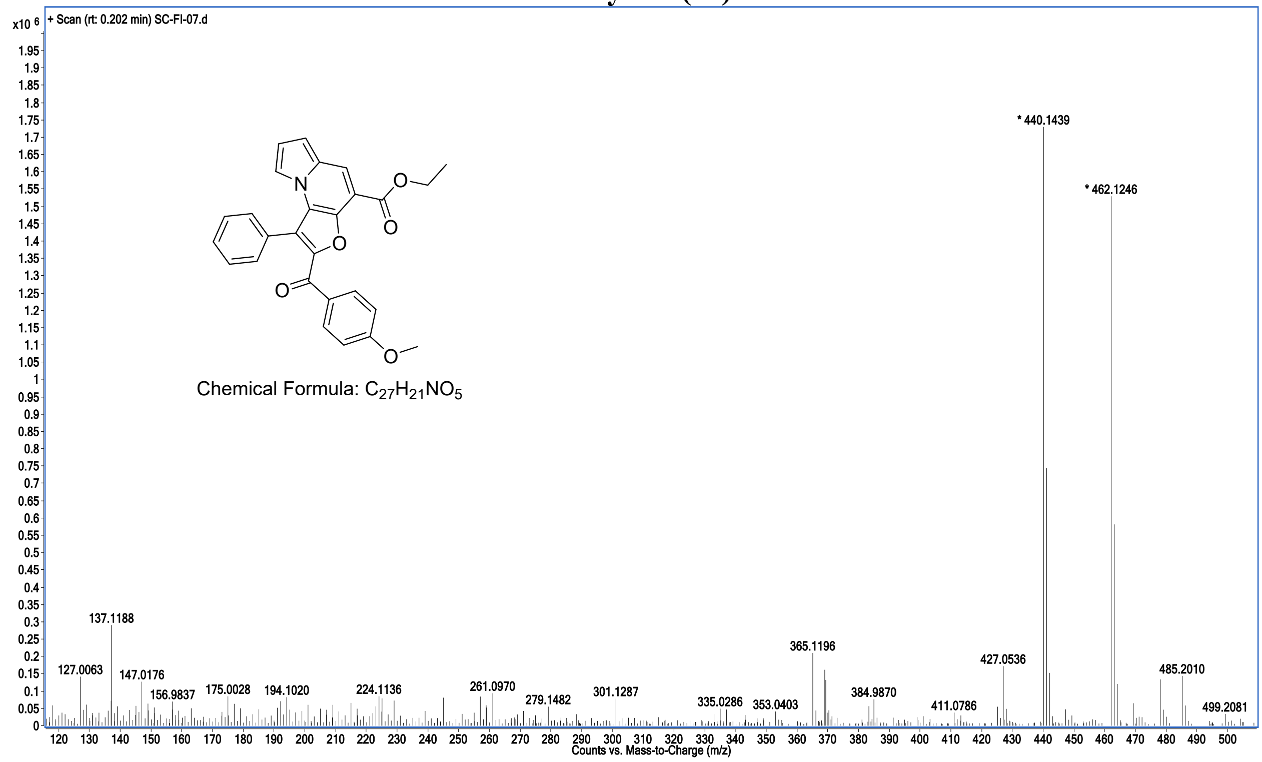

Figure S55. HRMS Spectrum of Ethyl 2-(4-methoxybenzoyl)-1-phenylfuro[3,2-*e*]indolizine-4-carboxylate (6c)

26. Ethyl 2-(3-methoxybenzoyl)-1-phenylfuro[3,2-*e*]indolizine-4-carboxylate (6d)

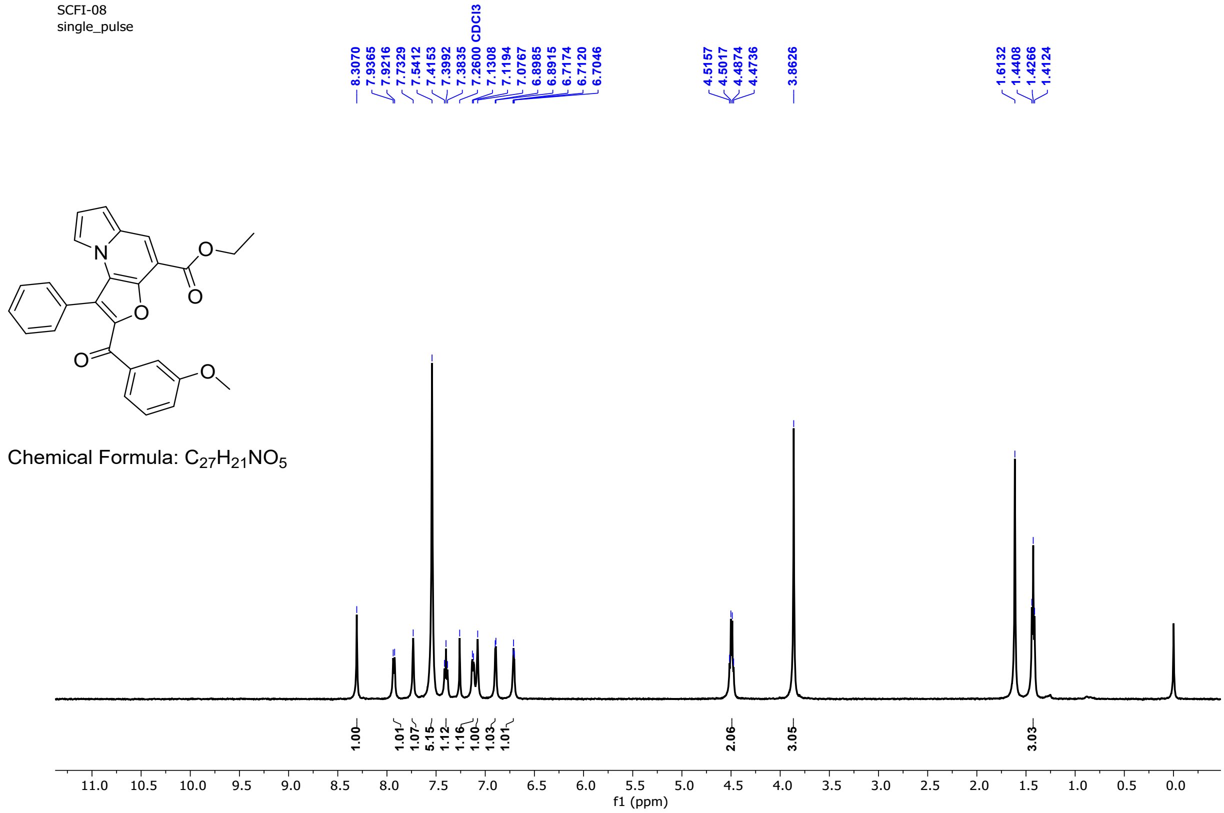

Figure S56. ^{1}H NMR Spectrum of Ethyl 2-(3-methoxybenzoyl)-1-phenylfuro[3,2-*e*]indolizine-4-carboxylate (6d)

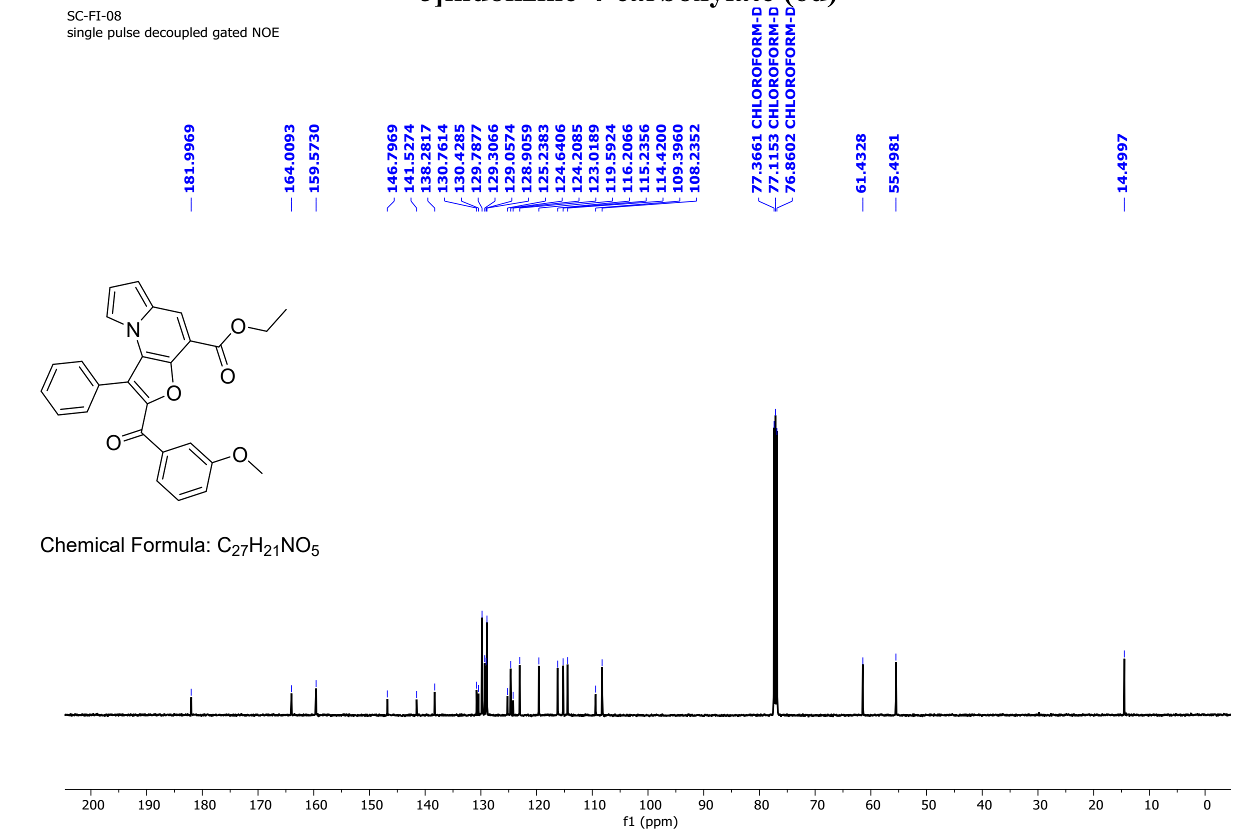

Figure S57. ^{13}C NMR Spectrum of Ethyl 2-(3-methoxybenzoyl)-1-phenylfuro[3,2-*e*]indolizine-4-carboxylate (6d)

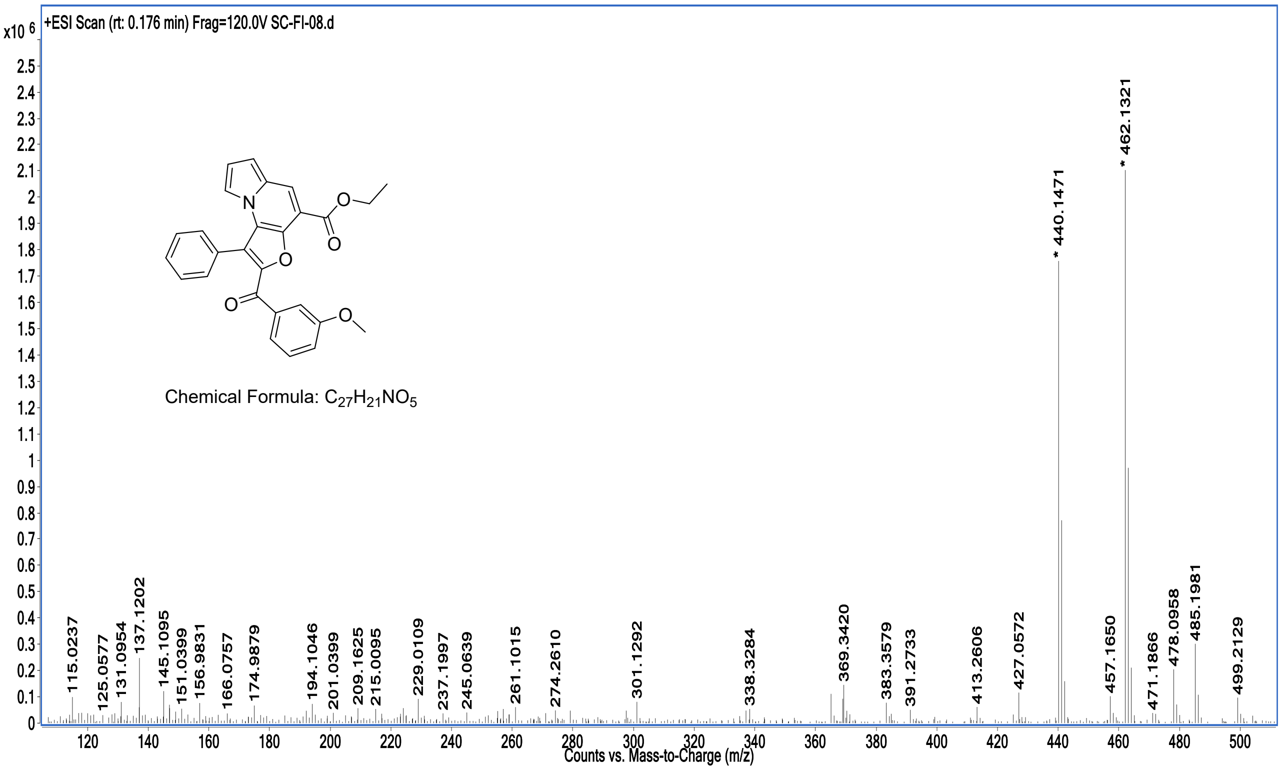

Figure S58. HRMS Spectrum of Ethyl 2-(3-methoxybenzoyl)-1-phenylfuro[3,2-*e*]indolizine-4-carboxylate (6d)

27. Ethyl 2-([1,1'-biphenyl]-4-carbonyl)-1-phenylfuro[3,2-*e*]indolizine-4-carboxylate (6e)

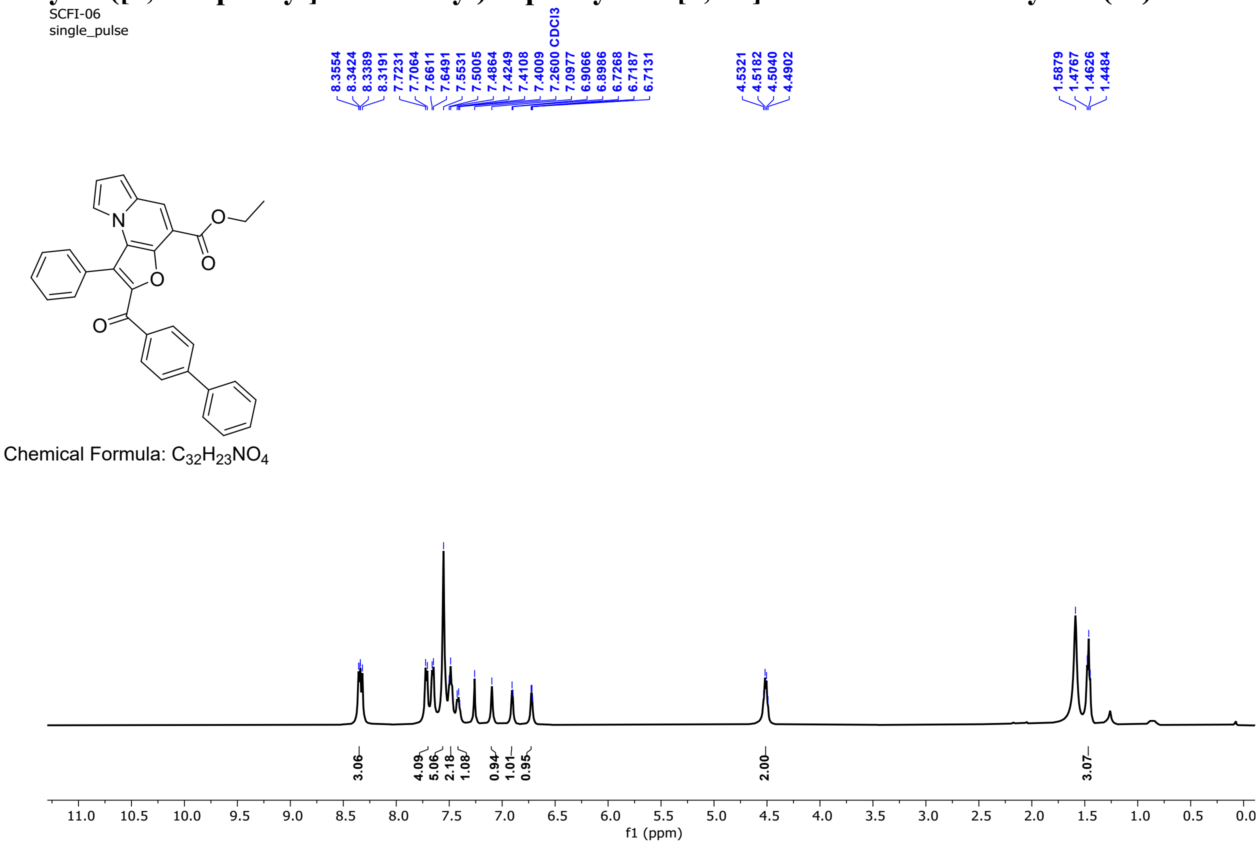

Figure S59. ^{1}H NMR Spectrum of Ethyl 2-([1,1'-biphenyl]-4-carbonyl)-1-phenylfuro[3,2-*e*]indolizine-4-carboxylate (6e)

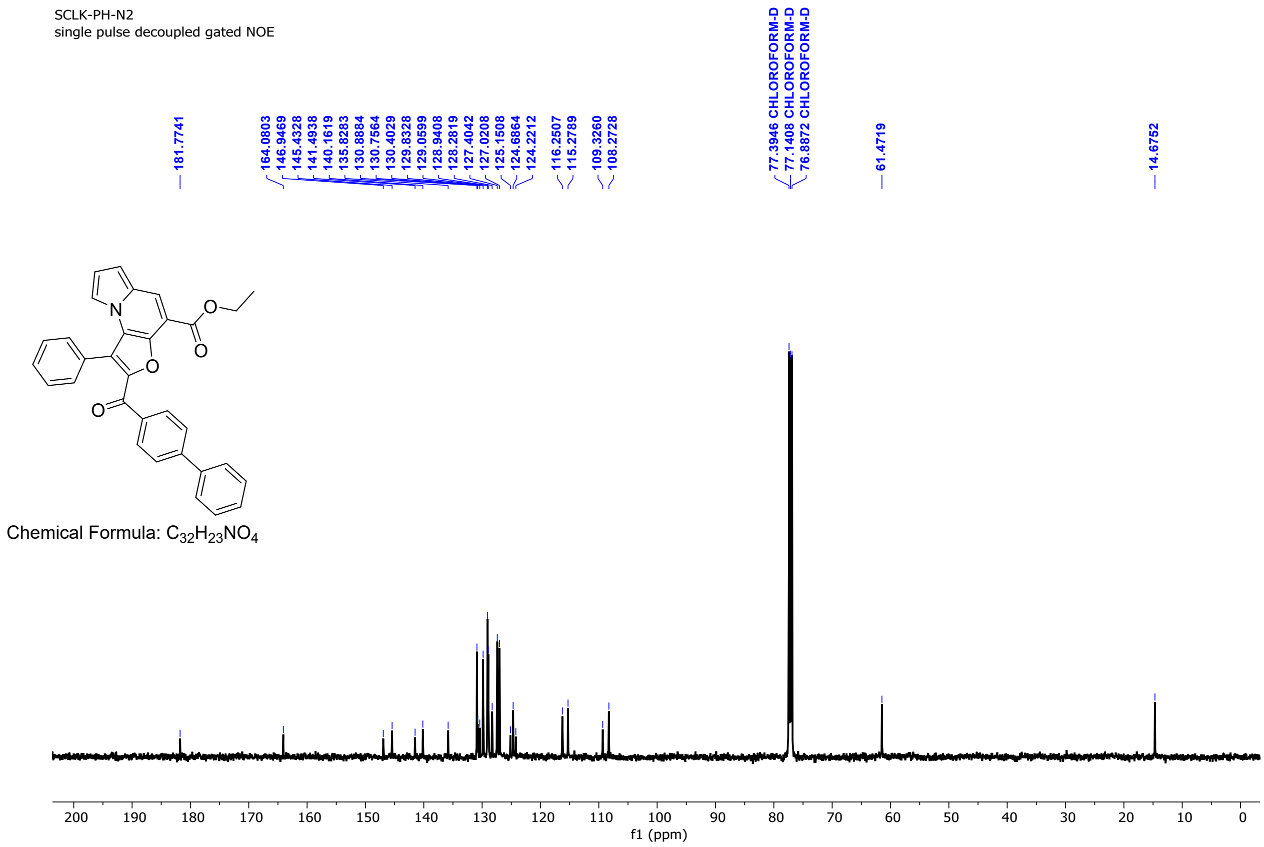

Figure S60. ^{13}**C NMR Spectrum of Ethyl 2-([1,1'-biphenyl]-4-carbonyl)-1-phenylfuro[3,2-*e*]indolizine-4-carboxylate (6e)**

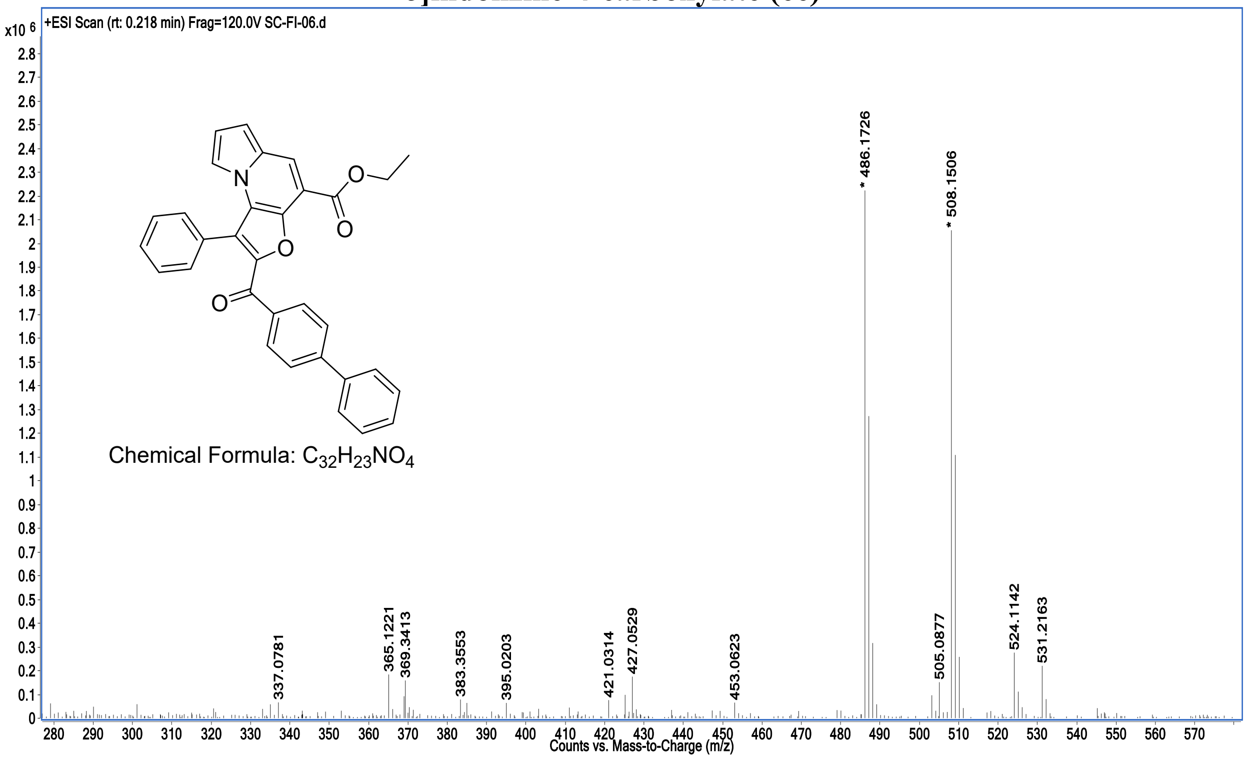

Figure S61. HRMS Spectrum of Ethyl 2-([1,1'-biphenyl]-4-carbonyl)-1-phenylfuro[3,2-*e*]indolizine-4-carboxylate (6e)

28. Ethyl 2-(4-nitrobenzoyl)-1-phenylfuro[3,2-*e*]indolizine-4-carboxylate (6f)

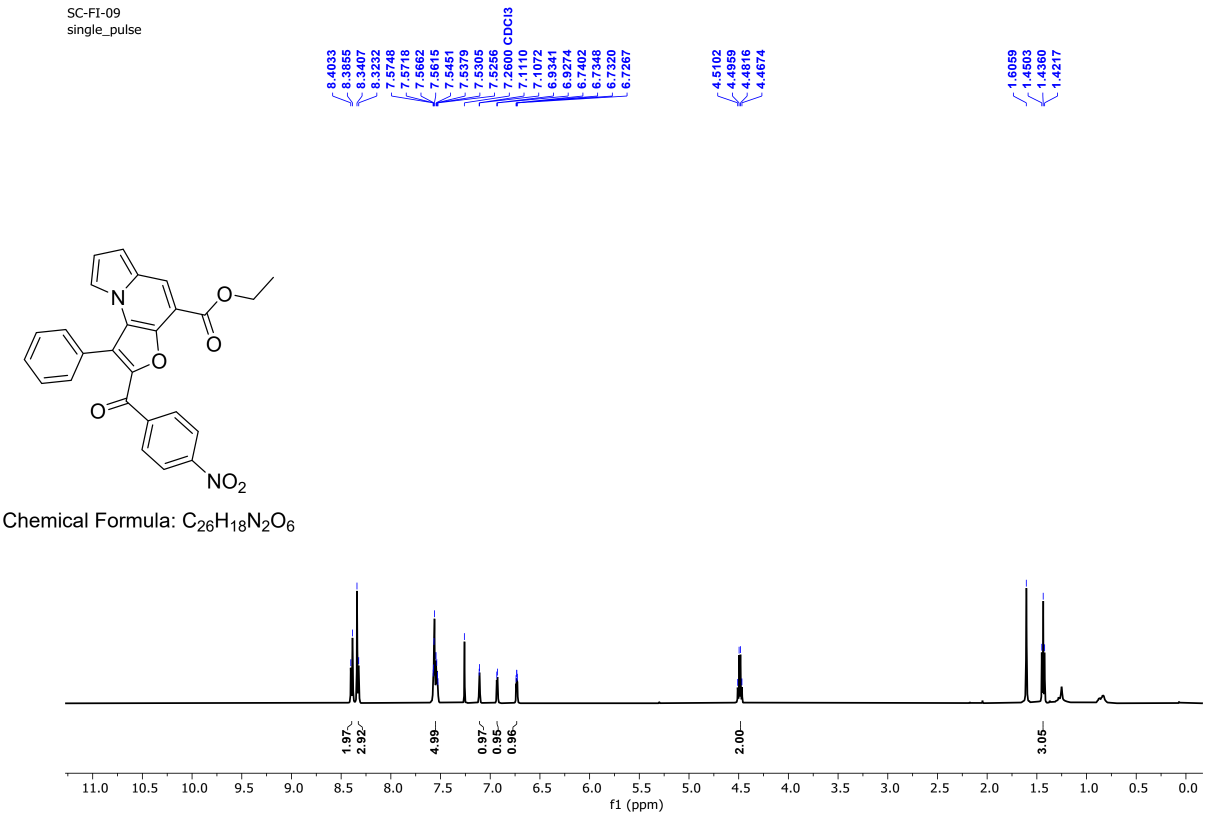

Figure S62. ^{1}H NMR Spectrum of Ethyl 2-(4-nitrobenzoyl)-1-phenylfuro[3,2-*e*]indolizine-4-carboxylate (6f)

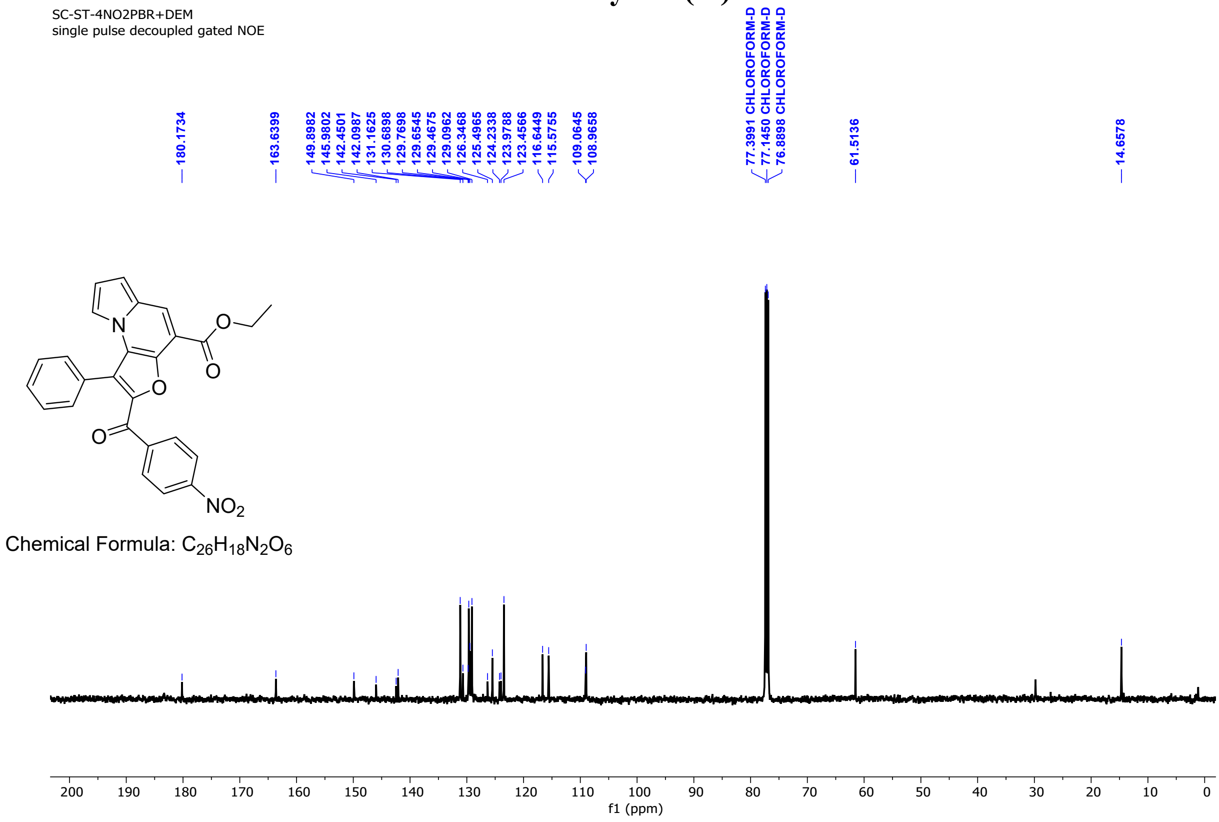

Figure S63. ^{13}C NMR Spectrum of Ethyl 2-(4-nitrobenzoyl)-1-phenylfuro[3,2-*e*]indolizine-4-carboxylate (6f)

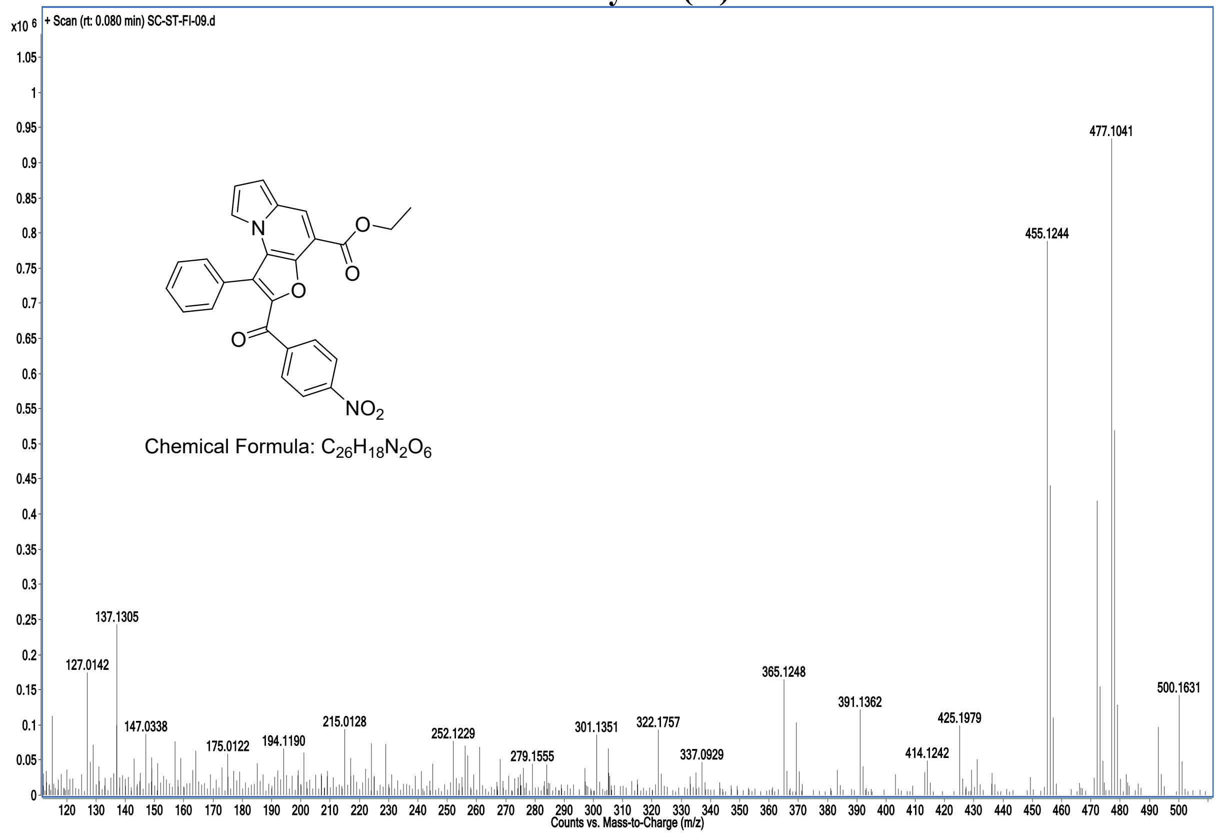

Figure S64. HRMS Spectrum of Ethyl 2-(4-nitrobenzoyl)-1-phenylfuro[3,2-*e*]indolizine-4-carboxylate (6f)

29. Ethyl 2-(3-nitrobenzoyl)-1-phenylfuro[3,2-*e*]indolizine-4-carboxylate (6g)

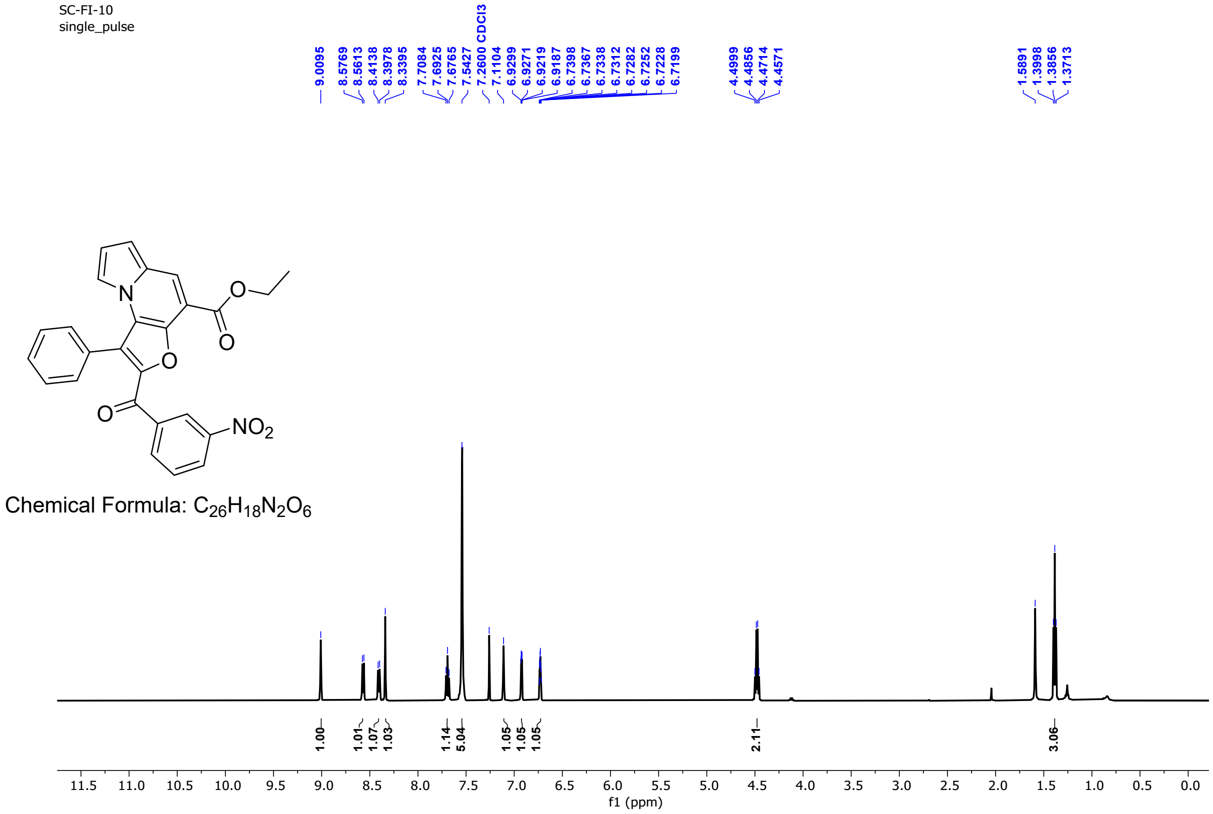

Figure S65. ^{1}**H NMR Spectrum of Ethyl 2-(3-nitrobenzoyl)-1-phenylfuro[3,2-*e*]indolizine-4-carboxylate (6g)**

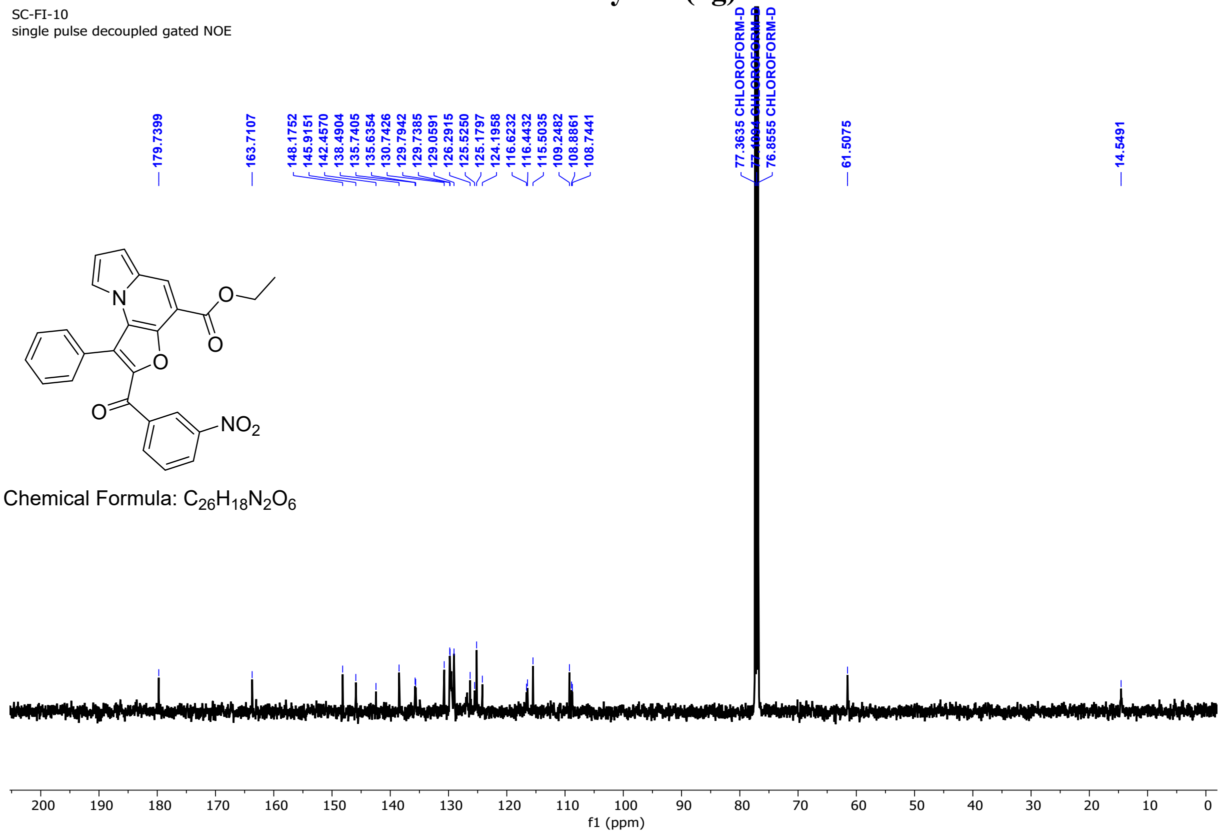

Figure S66. ^{13}**C NMR Spectrum of Ethyl 2-(3-nitrobenzoyl)-1-phenylfuro[3,2-*e*]indolizine-4-carboxylate (6g)**

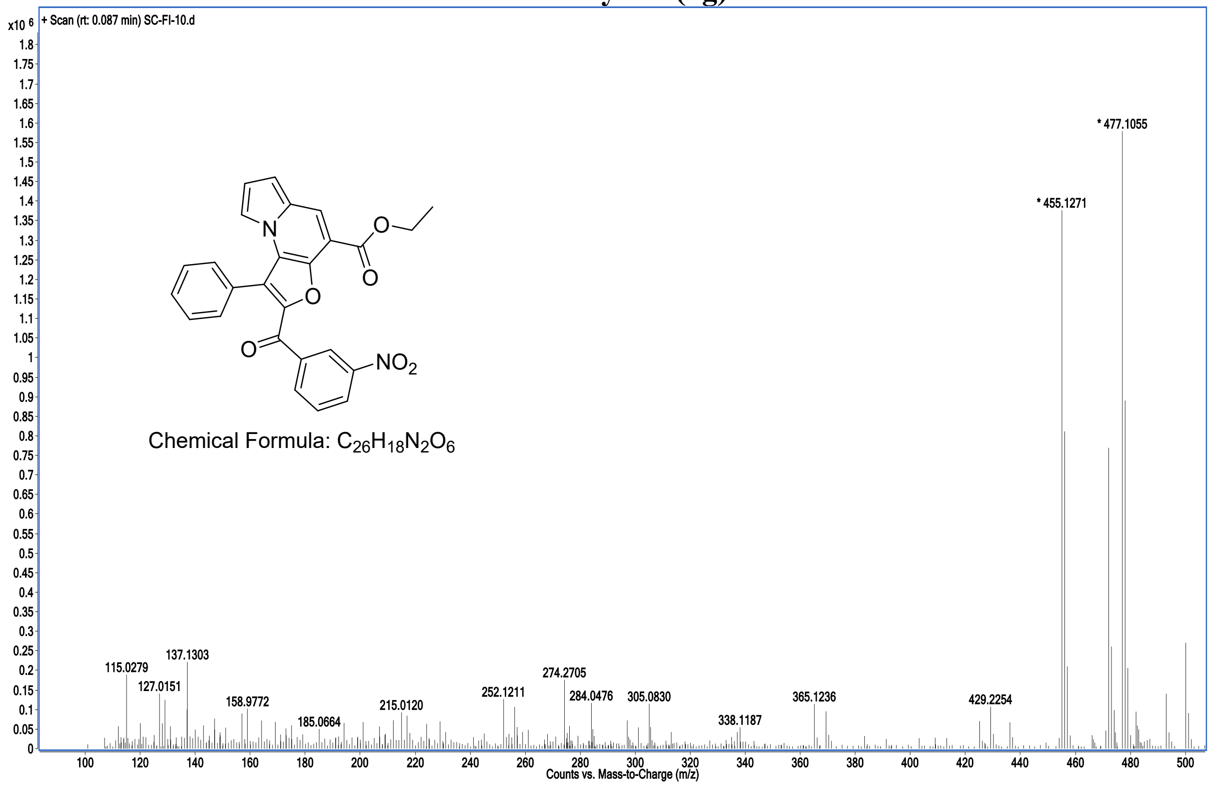

Figure S67. HRMS Spectrum of Ethyl 2-(3-nitrobenzoyl)-1-phenylfuro[3,2-*e*]indolizine-4-carboxylate (6g)

30. Ethyl 2-(3-bromobenzoyl)-1-phenylfuro[3,2-*e*]indolizine-4-carboxylate (6h)

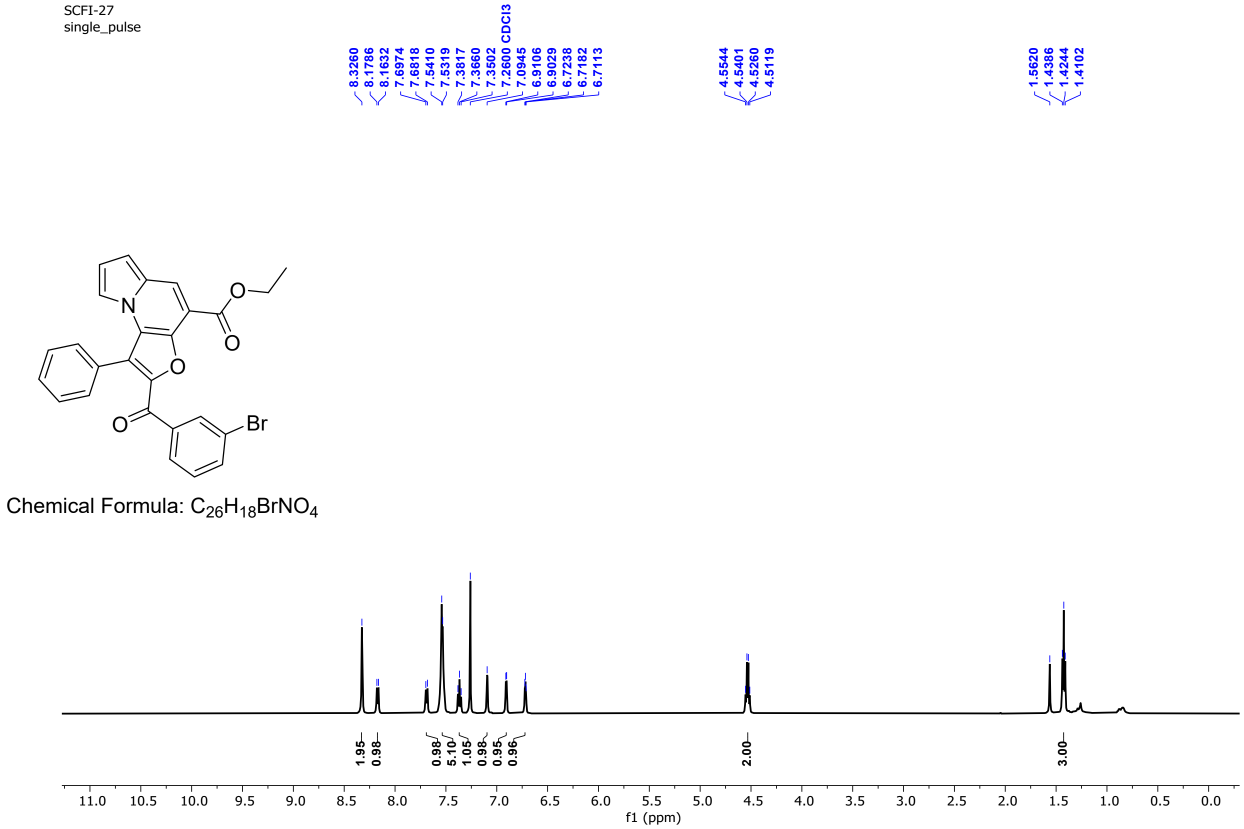

Figure S68. ^{1}H NMR Spectrum of Ethyl 2-(3-bromobenzoyl)-1-phenylfuro[3,2-*e*]indolizine-4-carboxylate (6h)

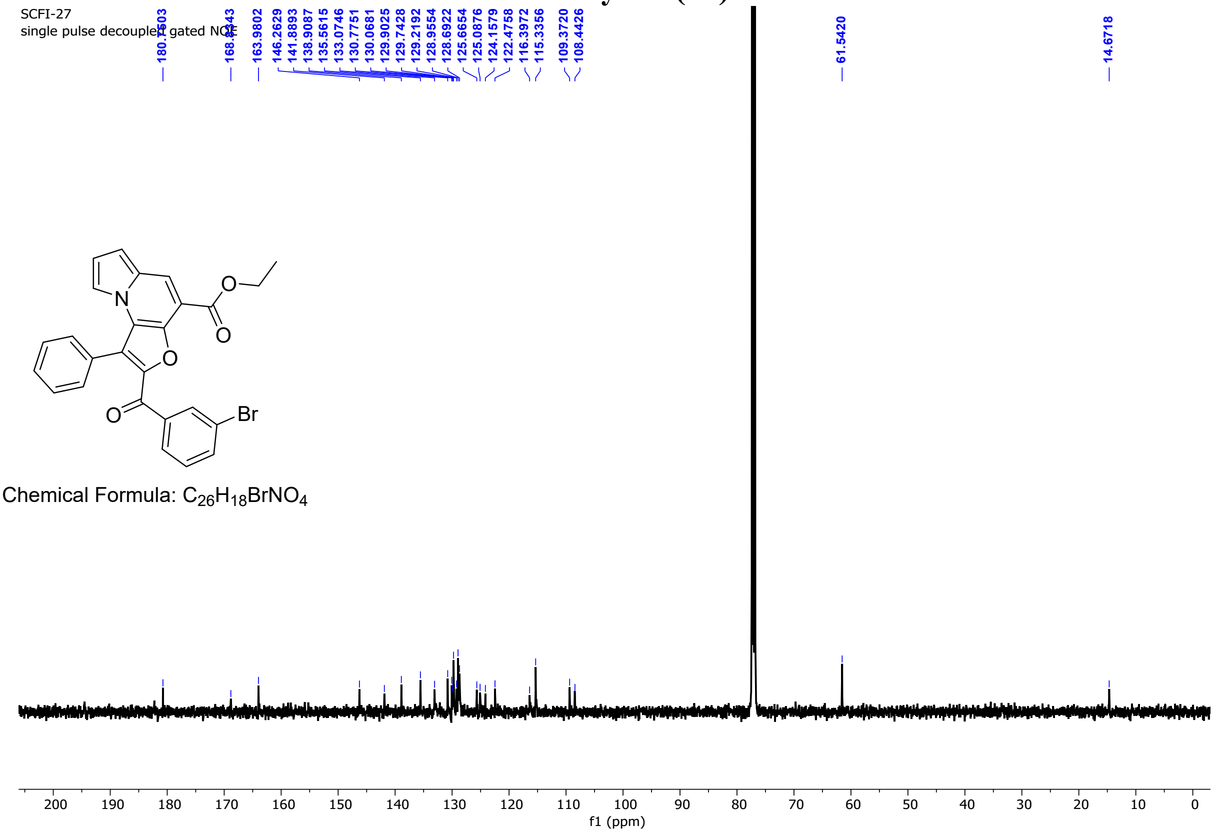

Figure S69. ^{13}C NMR Spectrum of Ethyl 2-(3-bromobenzoyl)-1-phenylfuro[3,2-*e*]indolizine-4-carboxylate (6h)

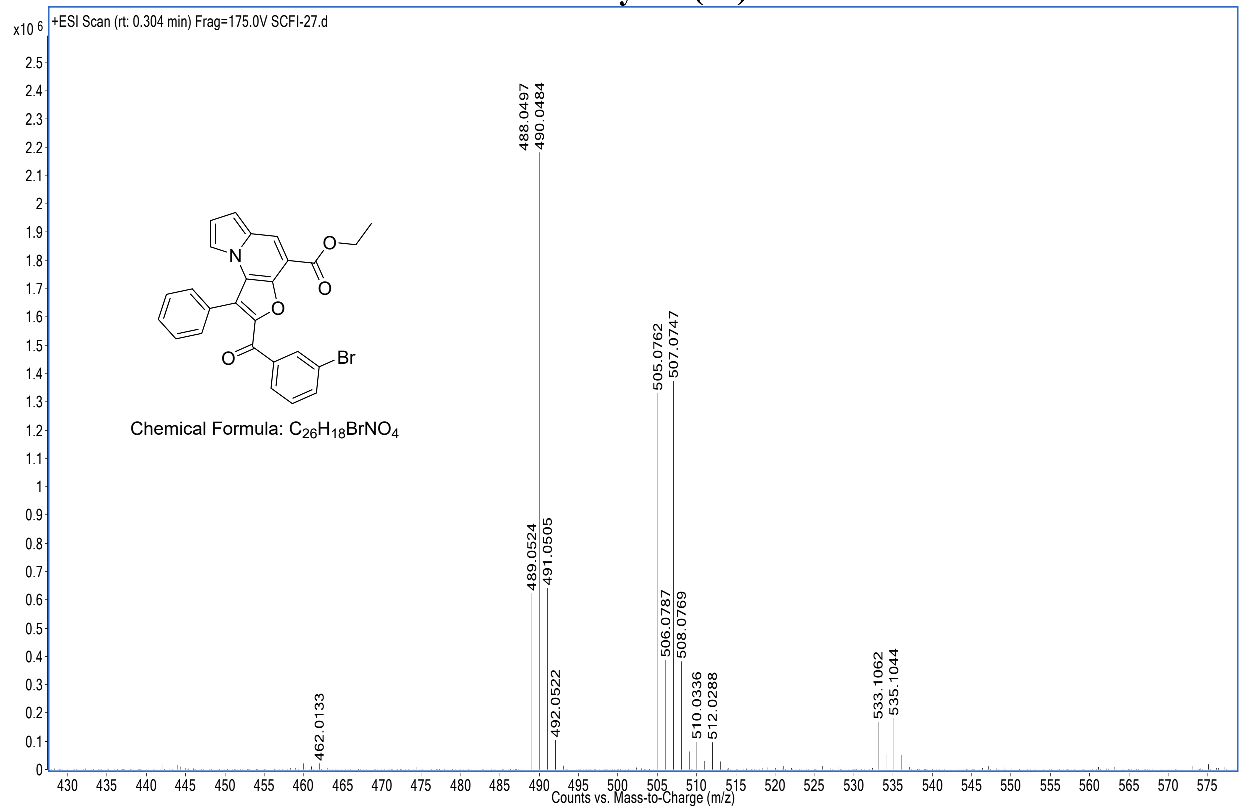

Figure S70. HRMS Spectrum of Ethyl 2-(3-bromobenzoyl)-1-phenylfuro[3,2-*e*]indolizine-4-carboxylate (6h)

31. Ethyl 2-(3,4-dichlorobenzoyl)-1-phenylfuro[3,2-*e*]indolizine-4-carboxylate (6i)

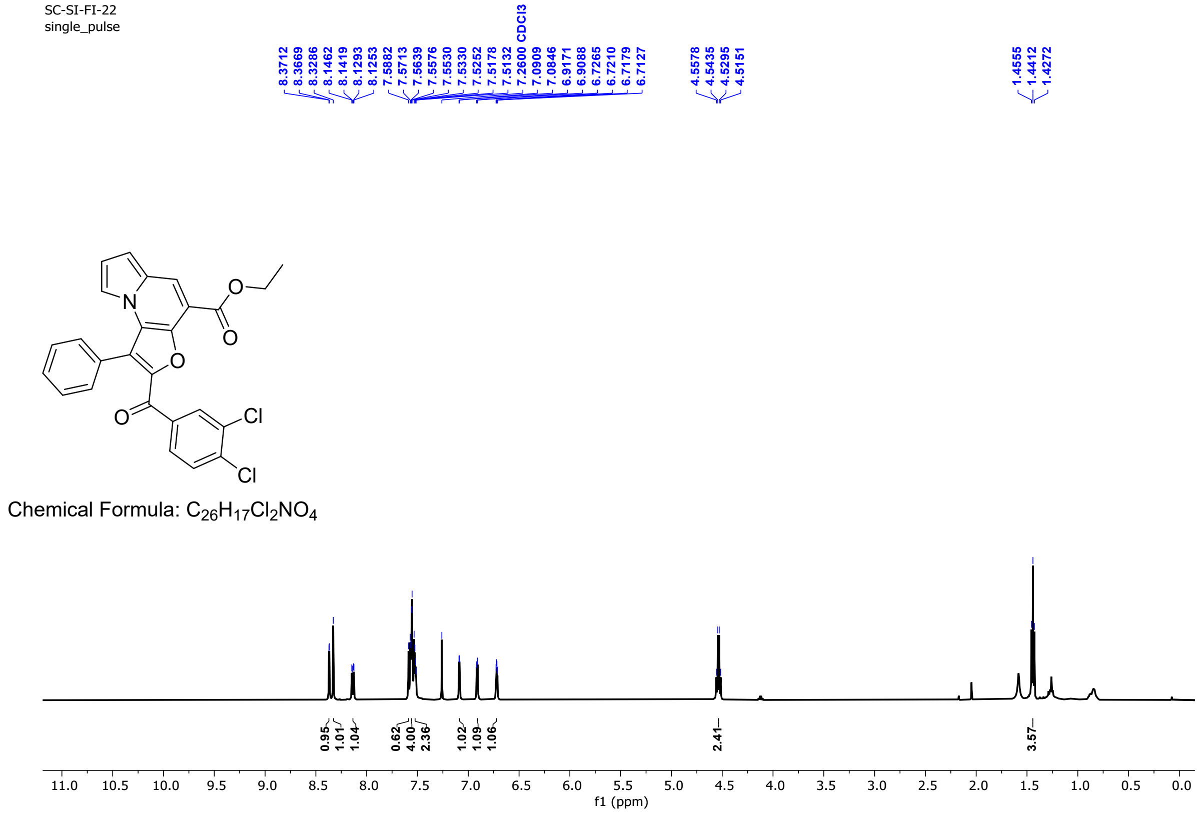

Figure S71. ^{1}H NMR Spectrum of Ethyl 2-(3,4-dichlorobenzoyl)-1-phenylfuro[3,2-*e*]indolizine-4-carboxylate (6i)

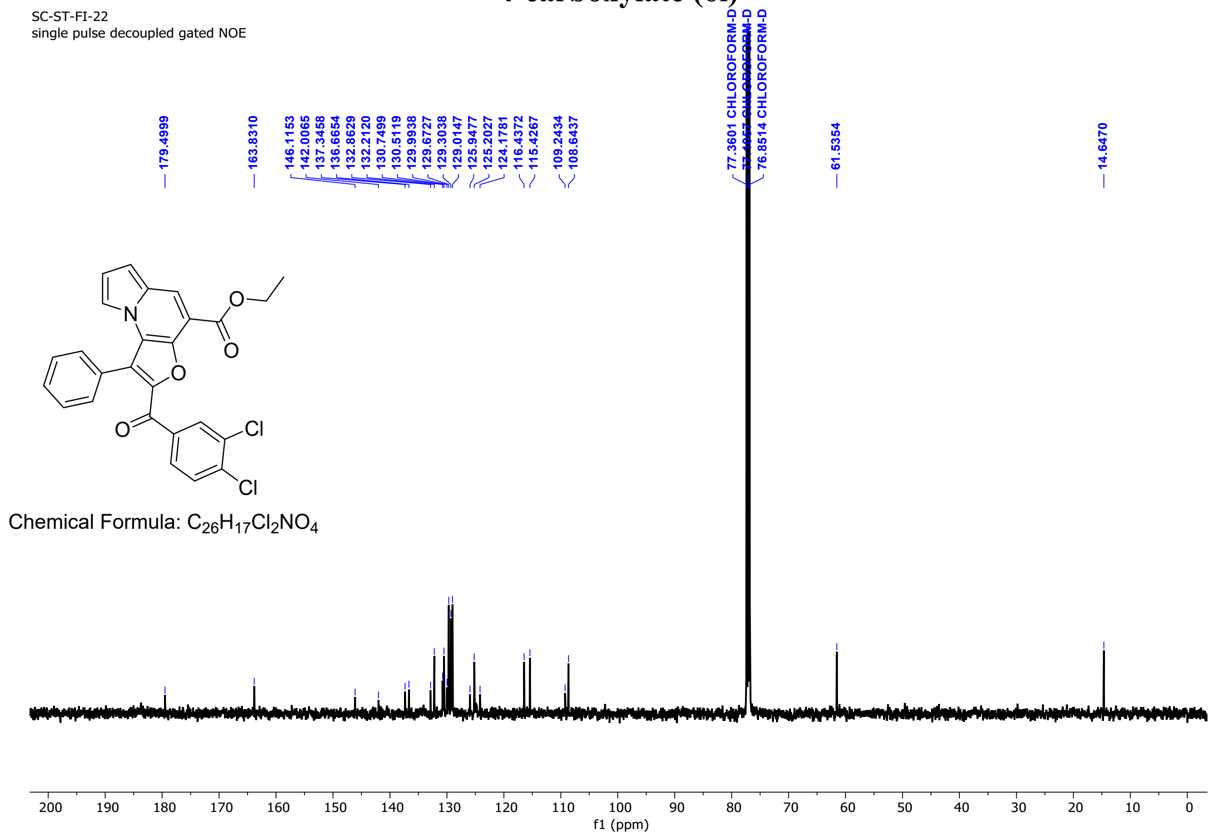

Figure S72. ^{13}C NMR Spectrum of Ethyl 2-(3,4-dichlorobenzoyl)-1-phenylfuro[3,2-*e*]indolizine-4-carboxylate (6i)

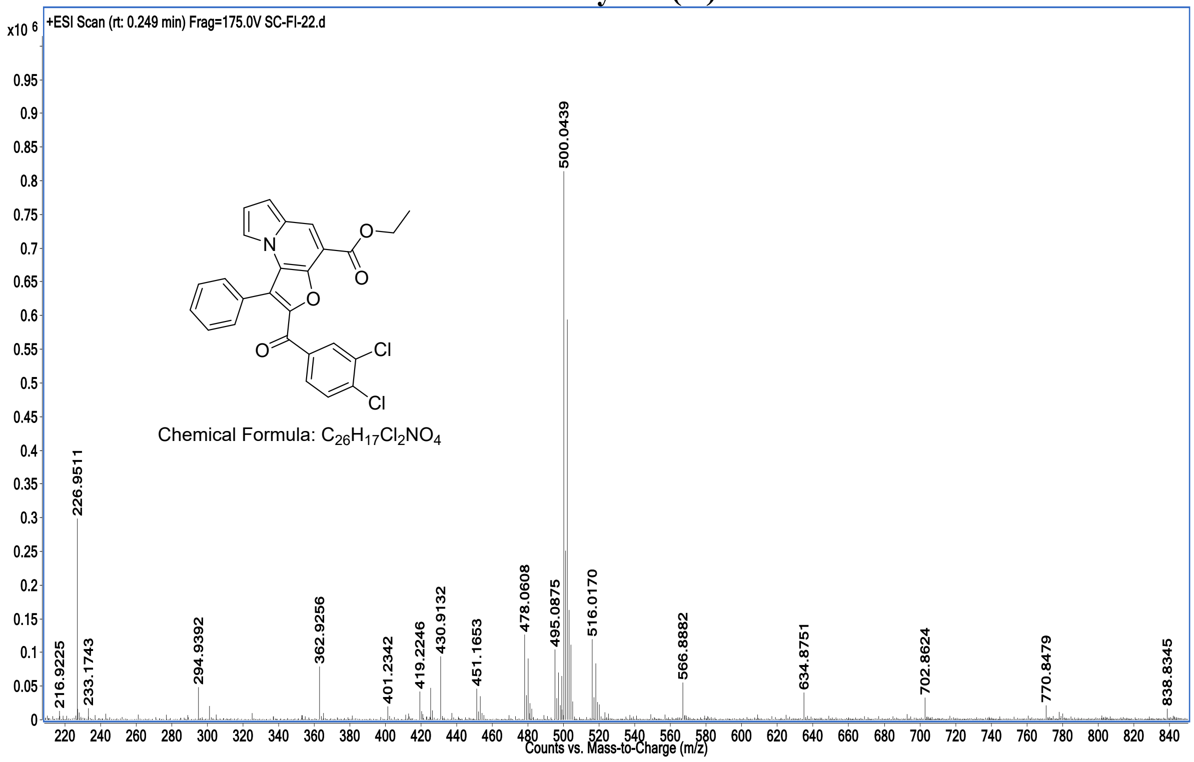

Figure S73. HRMS Spectrum of Ethyl 2-(3,4-dichlorobenzoyl)-1-phenylfuro[3,2-*e*]indolizine-4-carboxylate (6i)

32. Ethyl 2-(3,5-bis(trifluoromethyl)benzoyl)-1-phenylfuro[3,2-*e*]indolizine-4-carboxylate (6j)

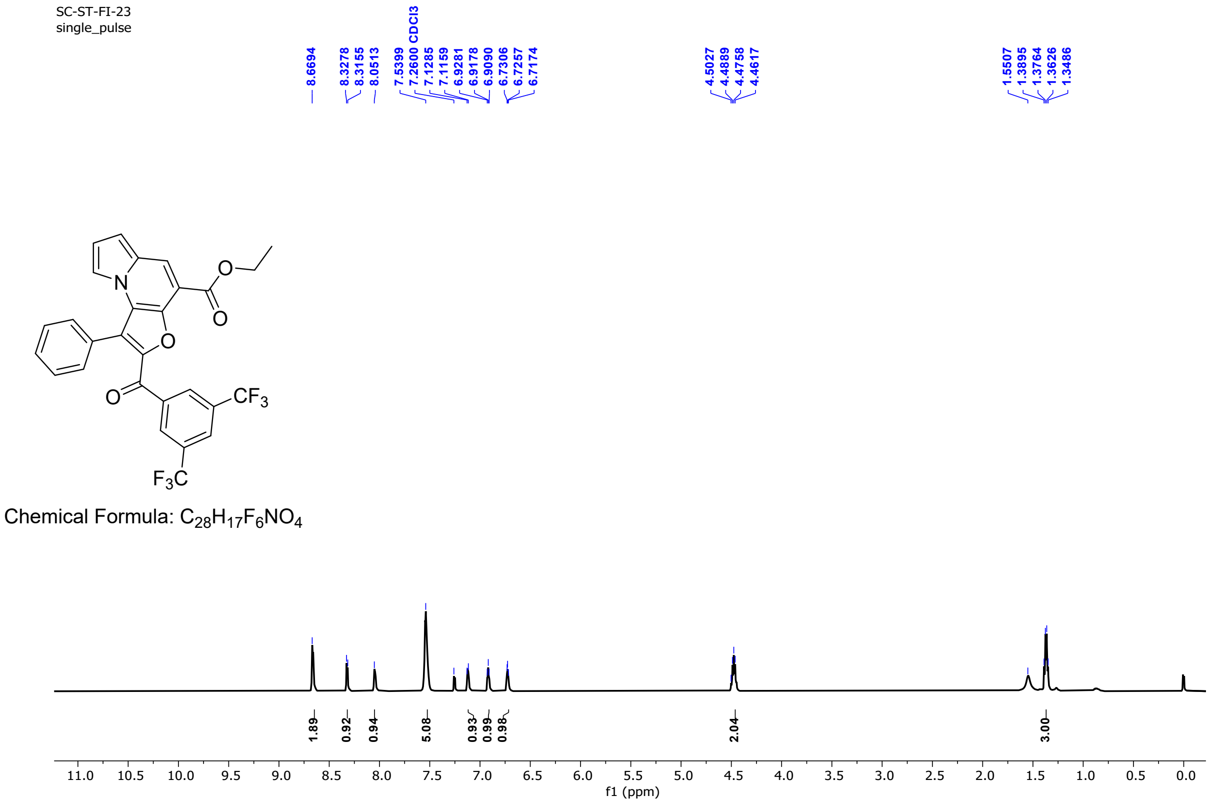

Figure S74. ¹H NMR Spectrum of Ethyl 2-(3,5-bis(trifluoromethyl)benzoyl)-1-phenylfuro[3,2-*e*]indolizine-4-carboxylate (6j)

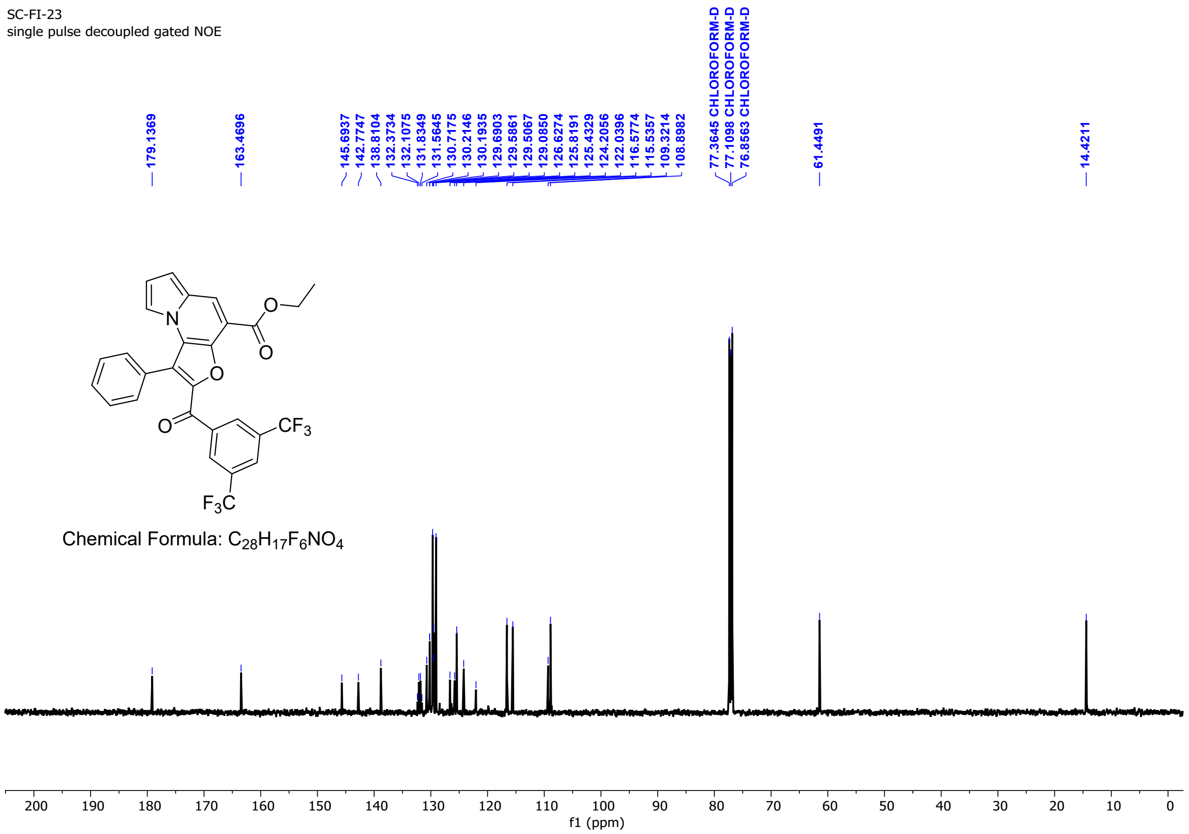

Figure S75. ^{13}C NMR Spectrum of Ethyl 2-(3,5-bis(trifluoromethyl)benzoyl)-1-phenylfuro[3,2-*e*]indolizine-4-carboxylate (6j)

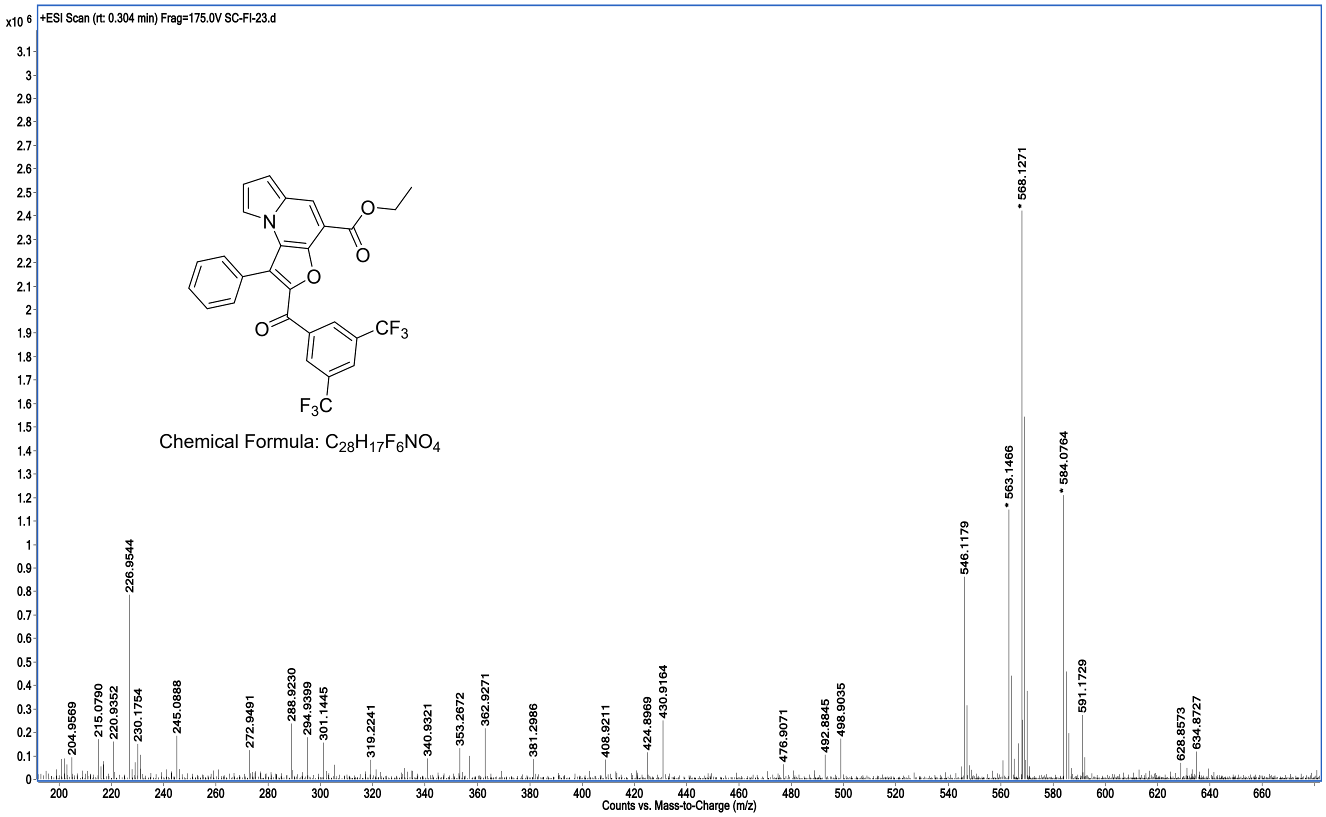

Figure S76. HRMS Spectrum of Ethyl 2-(3,5-bis(trifluoromethyl)benzoyl)-1-phenylfuro[3,2-*e*]indolizine-4-carboxylate (6j)

33. Ethyl 2-(2-fluorobenzoyl)-1-phenylfuro[3,2-*e*]indolizine-4-carboxylate (6k)

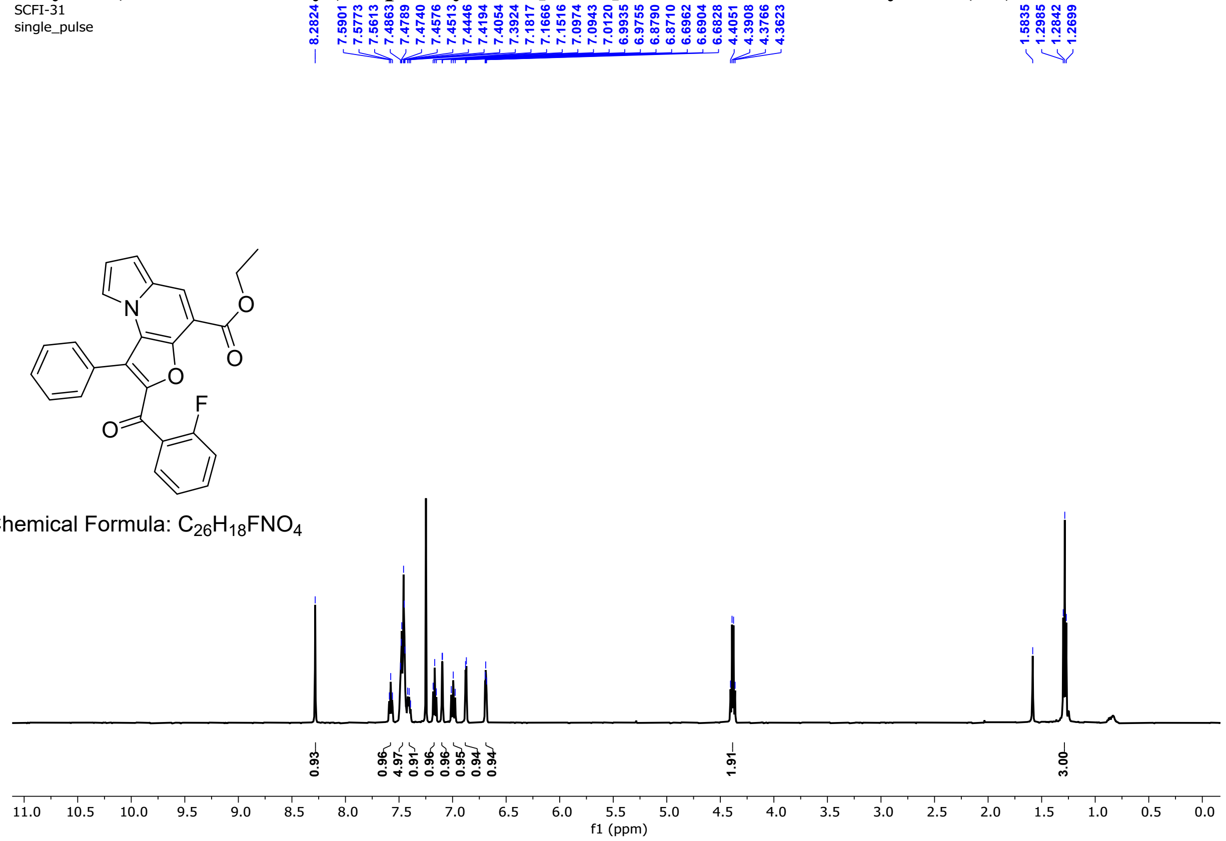

Figure S77. ^{1}H NMR Spectrum of Ethyl 2-(2-fluorobenzoyl)-1-phenylfuro[3,2-*e*]indolizine-4-carboxylate (6k)

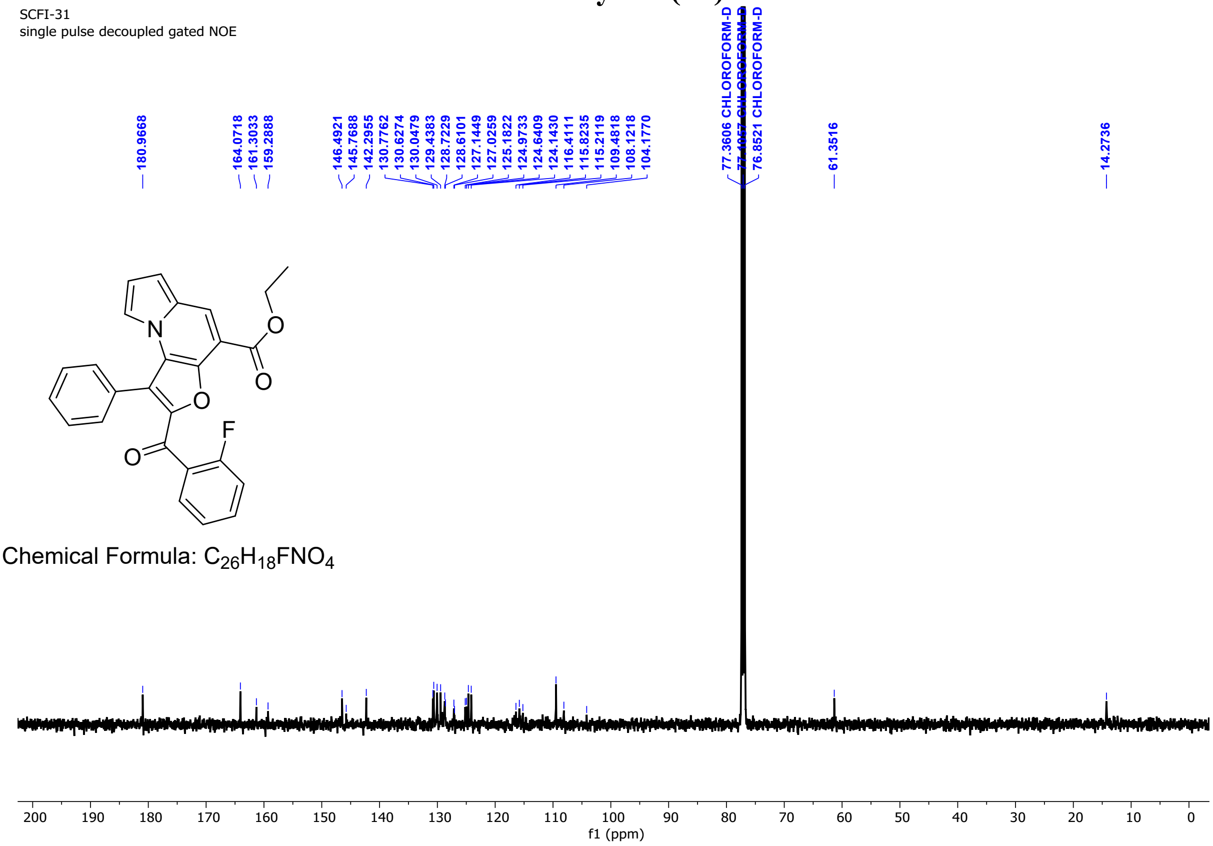

Figure S78. ^{13}C NMR Spectrum of Ethyl 2-(2-fluorobenzoyl)-1-phenylfuro[3,2-*e*]indolizine-4-carboxylate (6k)

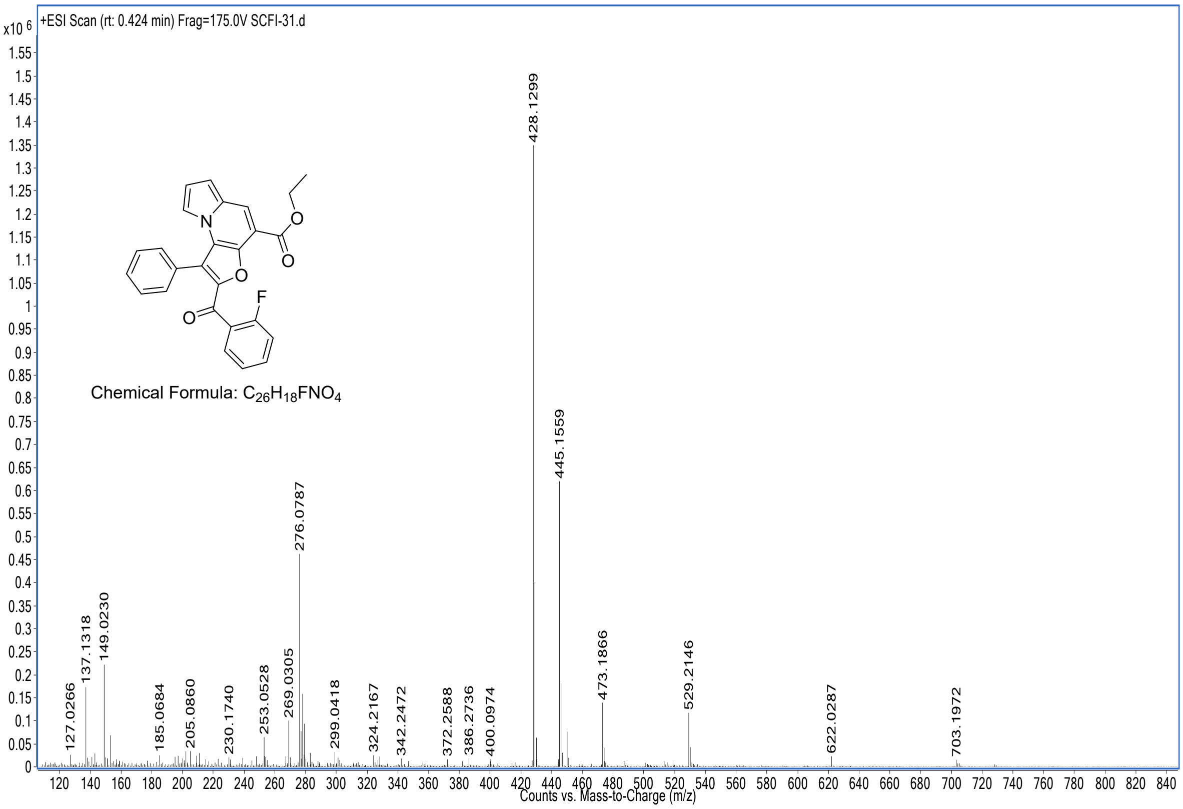

Figure S79. HRMS Spectrum of Ethyl 2-(2-fluorobenzoyl)-1-phenylfuro[3,2-*e*]indolizine-4-carboxylate (6k)

34. Ethyl 2-(2-methoxybenzoyl)-1-phenylfuro[3,2-*e*]indolizine-4-carboxylate (6l)

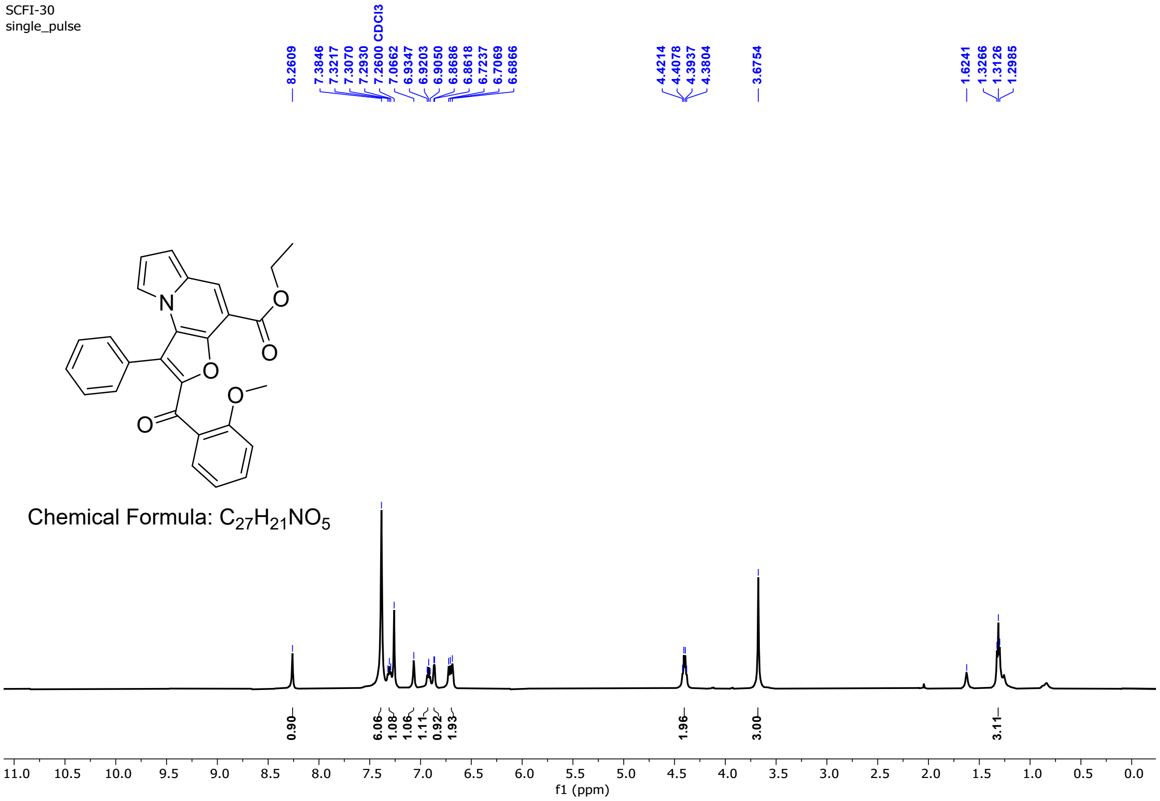

Figure S80. ^{1}H NMR Spectrum of Ethyl 2-(2-methoxybenzoyl)-1-phenylfuro[3,2-*e*]indolizine-4-carboxylate (6l)

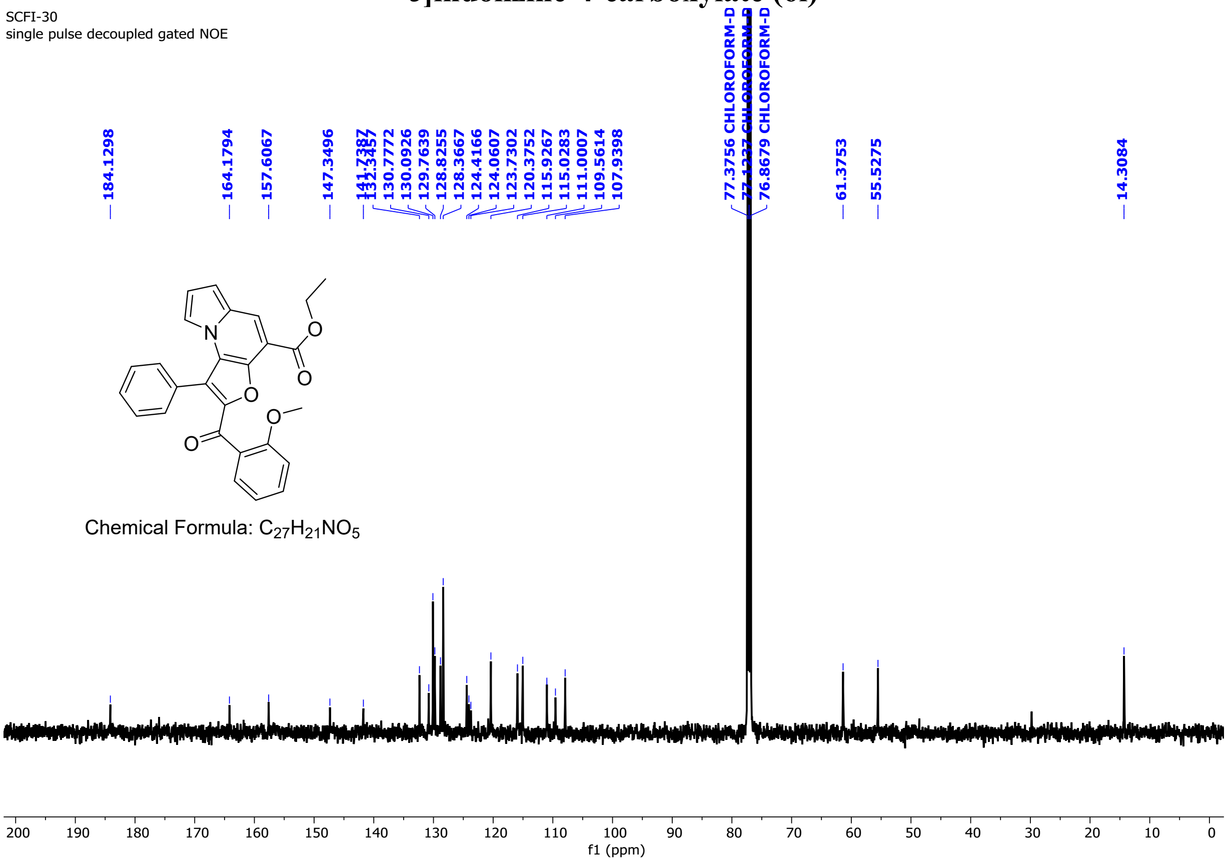

Figure S81. ^{13}C NMR Spectrum of Ethyl 2-(2-methoxybenzoyl)-1-phenylfuro[3,2-*e*]indolizine-4-carboxylate (6l)

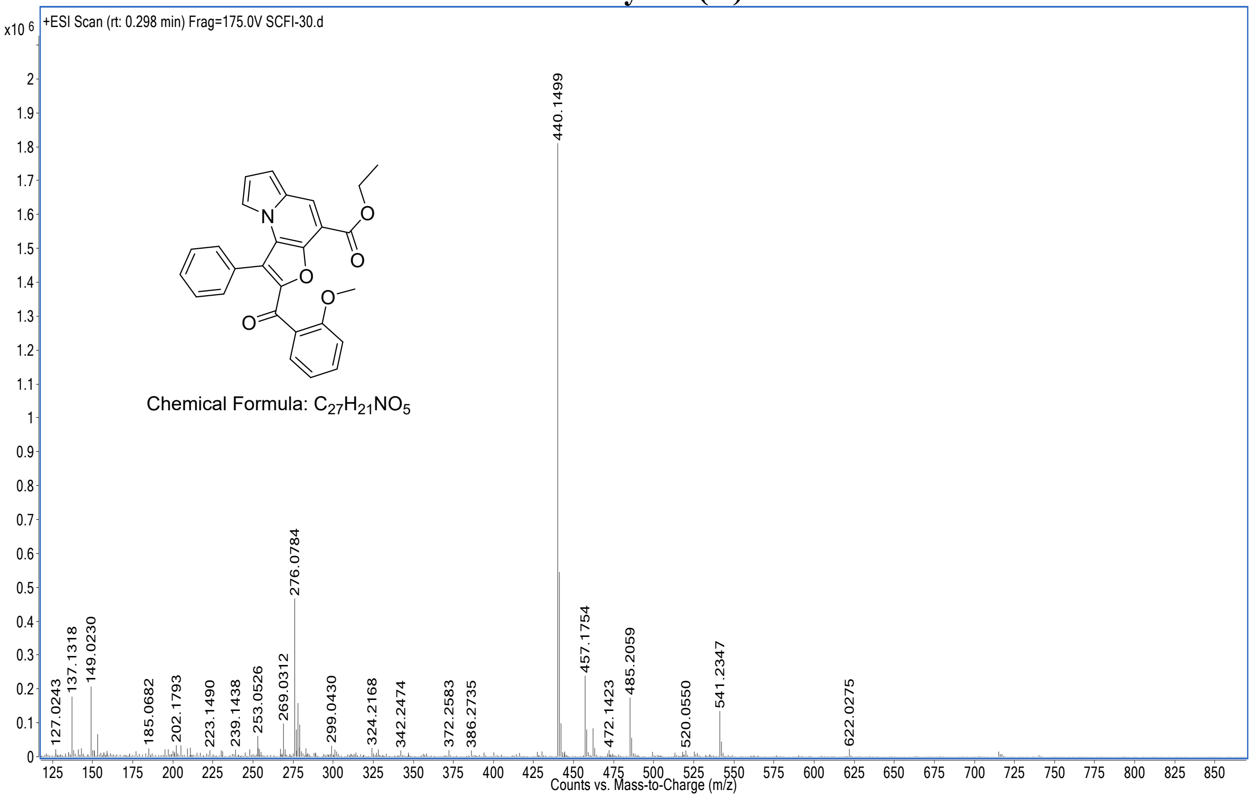

Figure S82. HRMS Spectrum of Ethyl 2-(2-methoxybenzoyl)-1-phenylfuro[3,2-*e*]indolizine-4-carboxylate (6l)

35. Ethyl 2-benzoyl-1-(4-methoxyphenyl)furo[3,2-*e*]indolizine-4-carboxylate (6m)

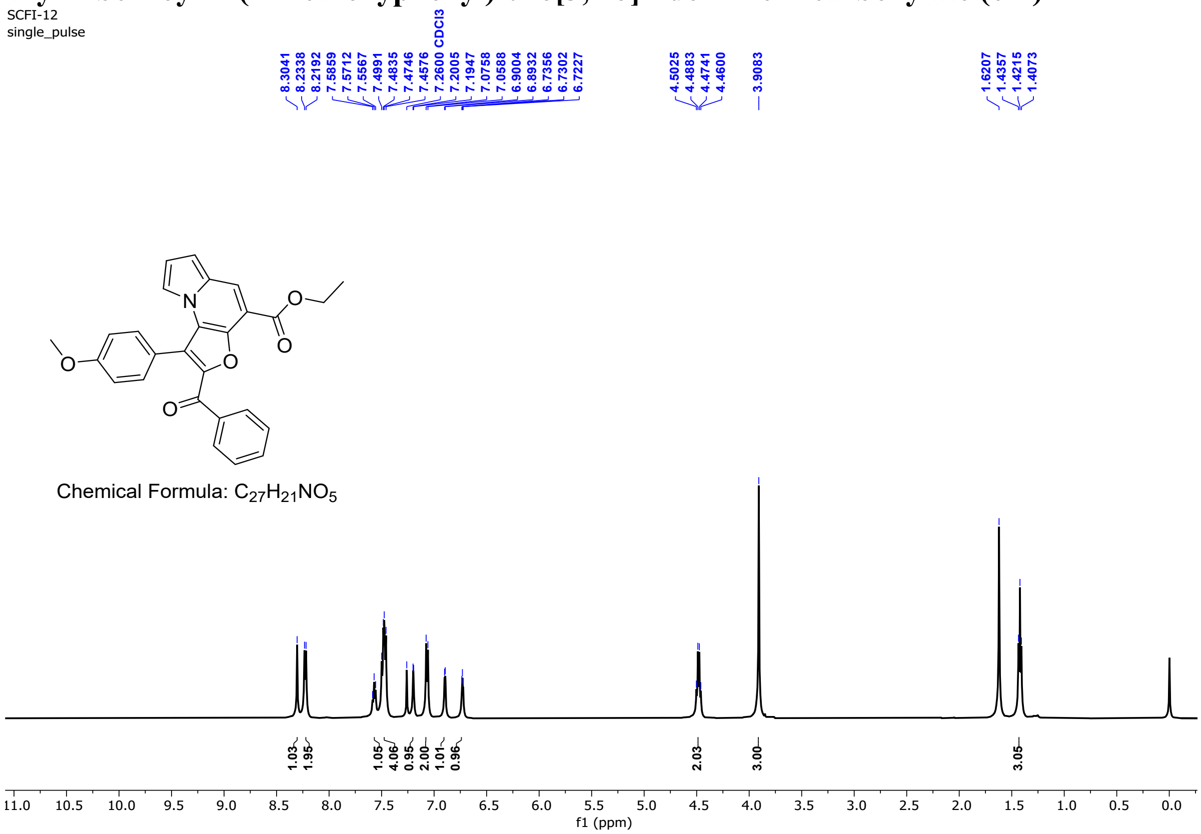

Figure S83. ^{1}H NMR Spectrum of Ethyl 2-benzoyl-1-(4-methoxyphenyl)furo[3,2-*e*]indolizine-4-carboxylate (6m)

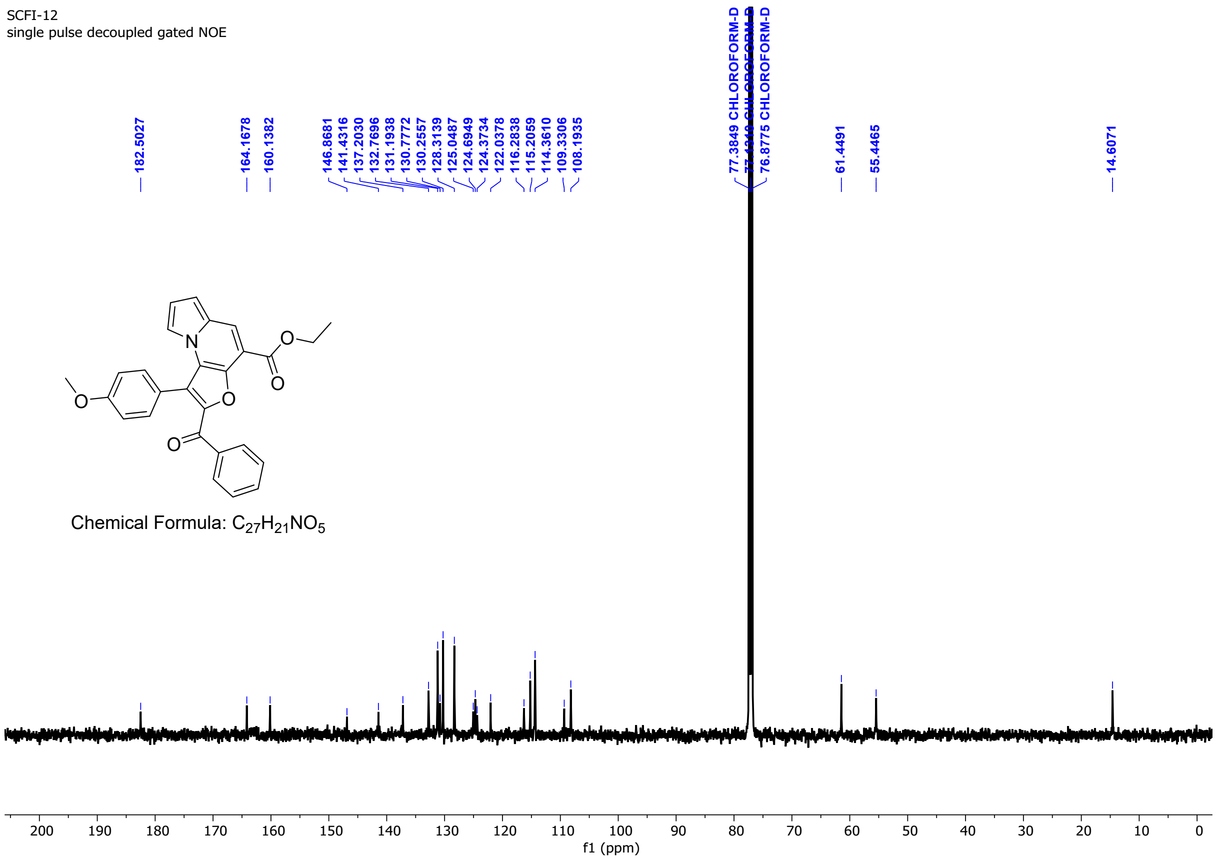

Figure S84. ^{13}C NMR Spectrum of Ethyl 2-benzoyl-1-(4-methoxyphenyl)furo[3,2-*e*]indolizine-4-carboxylate (6m)

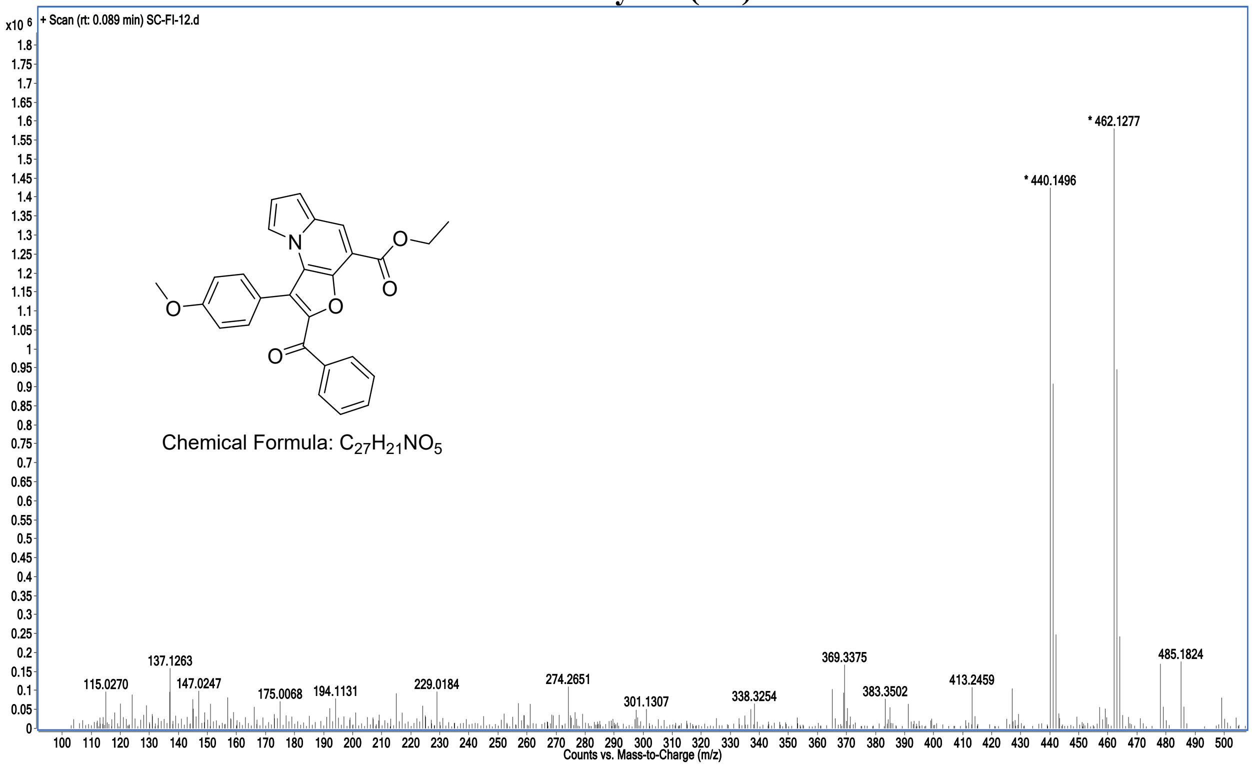

Figure S85. HRMS Spectrum of Ethyl 2-benzoyl-1-(4-methoxyphenyl)furo[3,2-*e*]indolizine-4-carboxylate (6m)

36. Ethyl 2-(4-chlorobenzoyl)-1-(4-methoxyphenyl)furo[3,2-*e*]indolizine-4-carboxylate (6n)

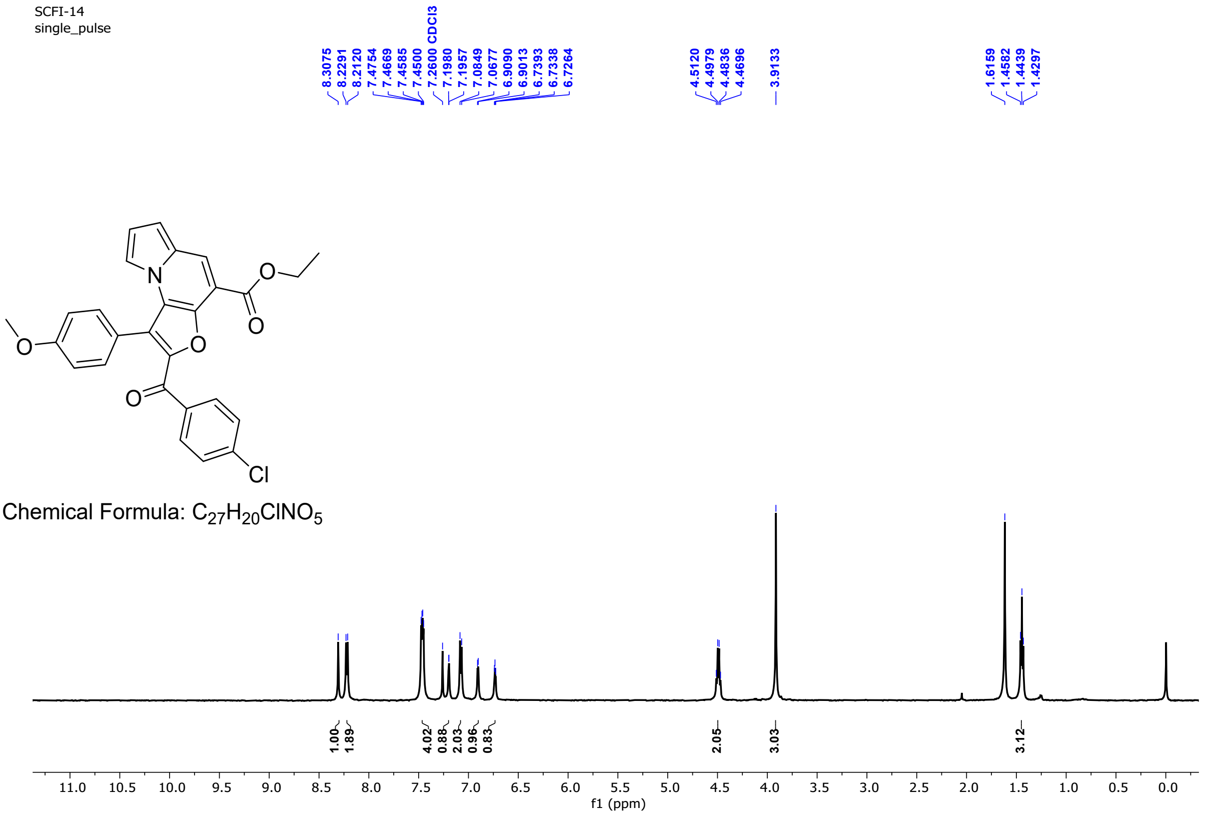

Figure S86. ^{1}H NMR Spectrum of Ethyl 2-(4-chlorobenzoyl)-1-(4-methoxyphenyl)furo[3,2-*e*]indolizine-4-carboxylate (6n)

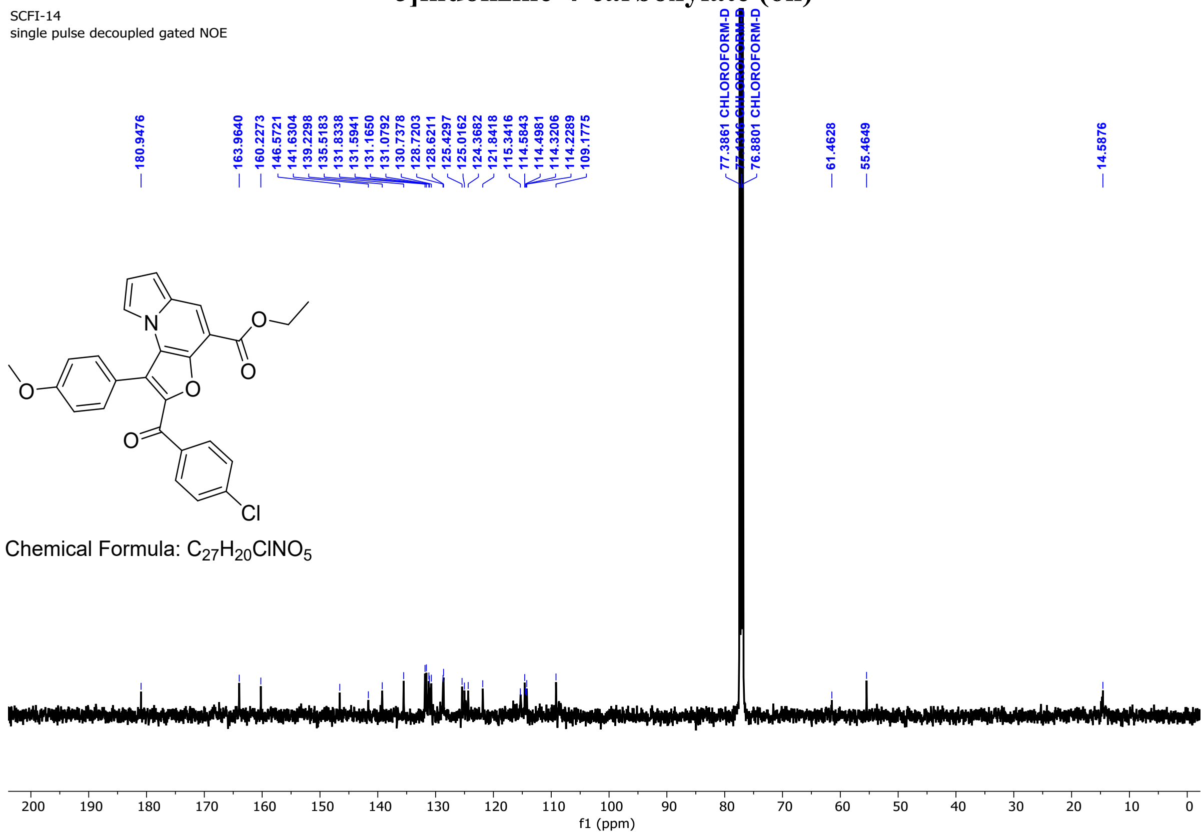

Figure S87. ^{13}C NMR Spectrum of Ethyl 2-(4-chlorobenzoyl)-1-(4-methoxyphenyl)furo[3,2-*e*]indolizine-4-carboxylate (6n)

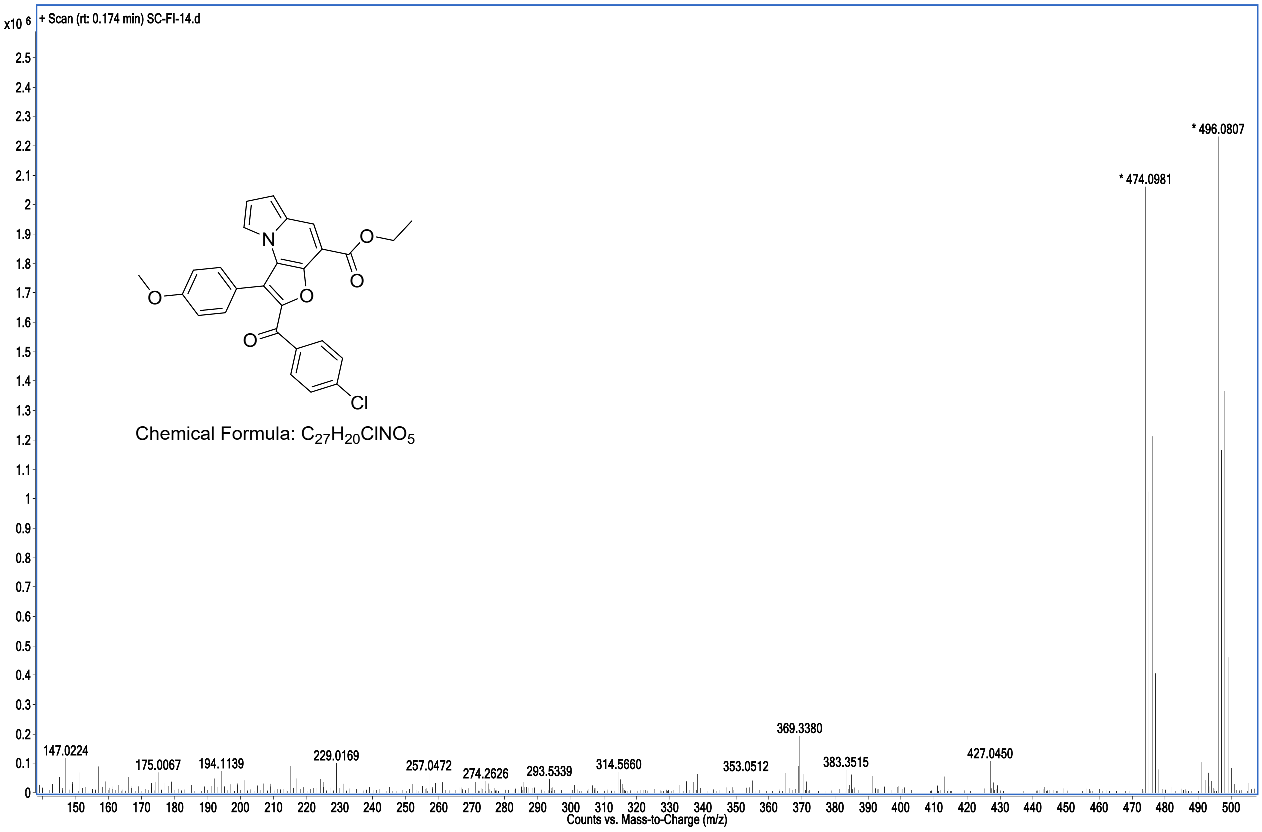

Figure S88. HRMS Spectrum of Ethyl 2-(4-chlorobenzoyl)-1-(4-methoxyphenyl)furo[3,2-*e*]indolizine-4-carboxylate (6n)

37. Ethyl 2-(3-methoxybenzoyl)-1-(4-methoxyphenyl)furo[3,2-*e*]indolizine-4-carboxylate (6o)

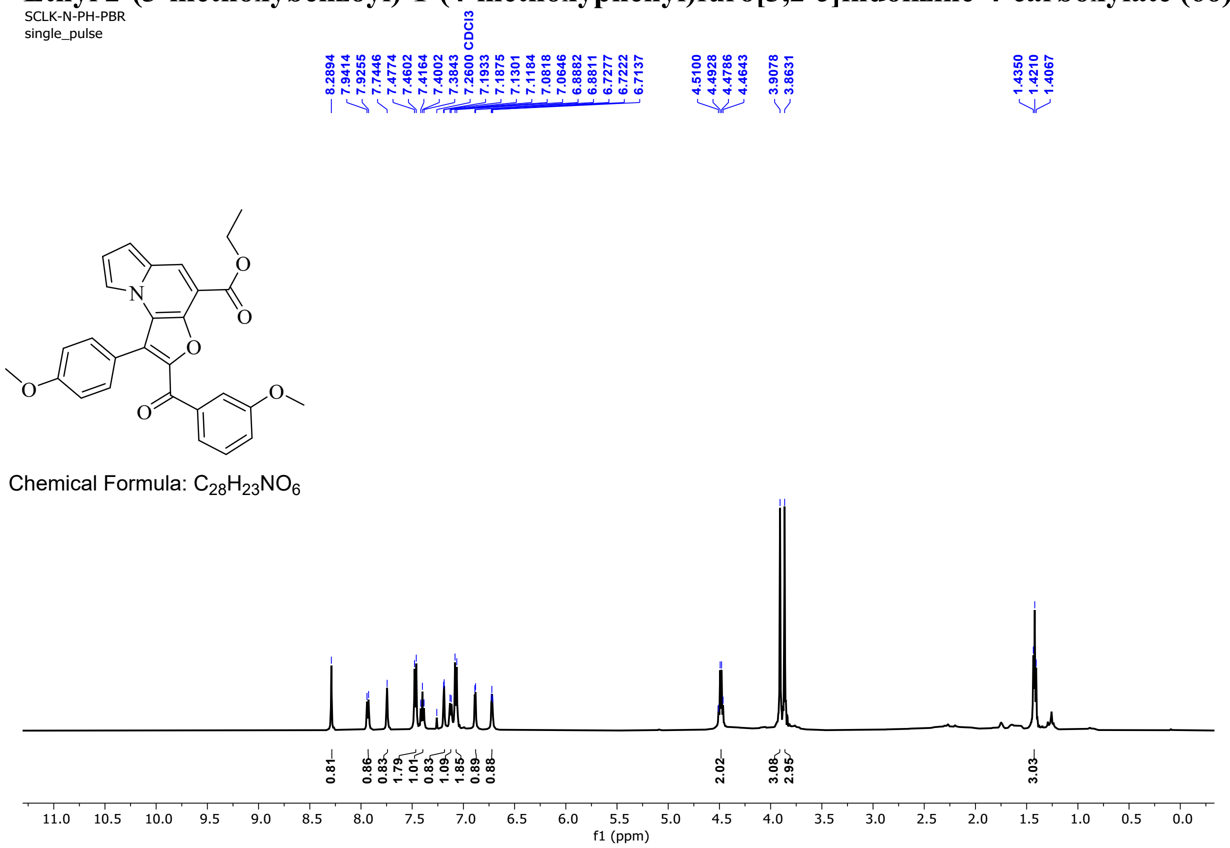

Figure S89. ^{1}H NMR Spectrum of Ethyl 2-(3-methoxybenzoyl)-1-(4-methoxyphenyl)furo[3,2-*e*]indolizine-4-carboxylate (6o)

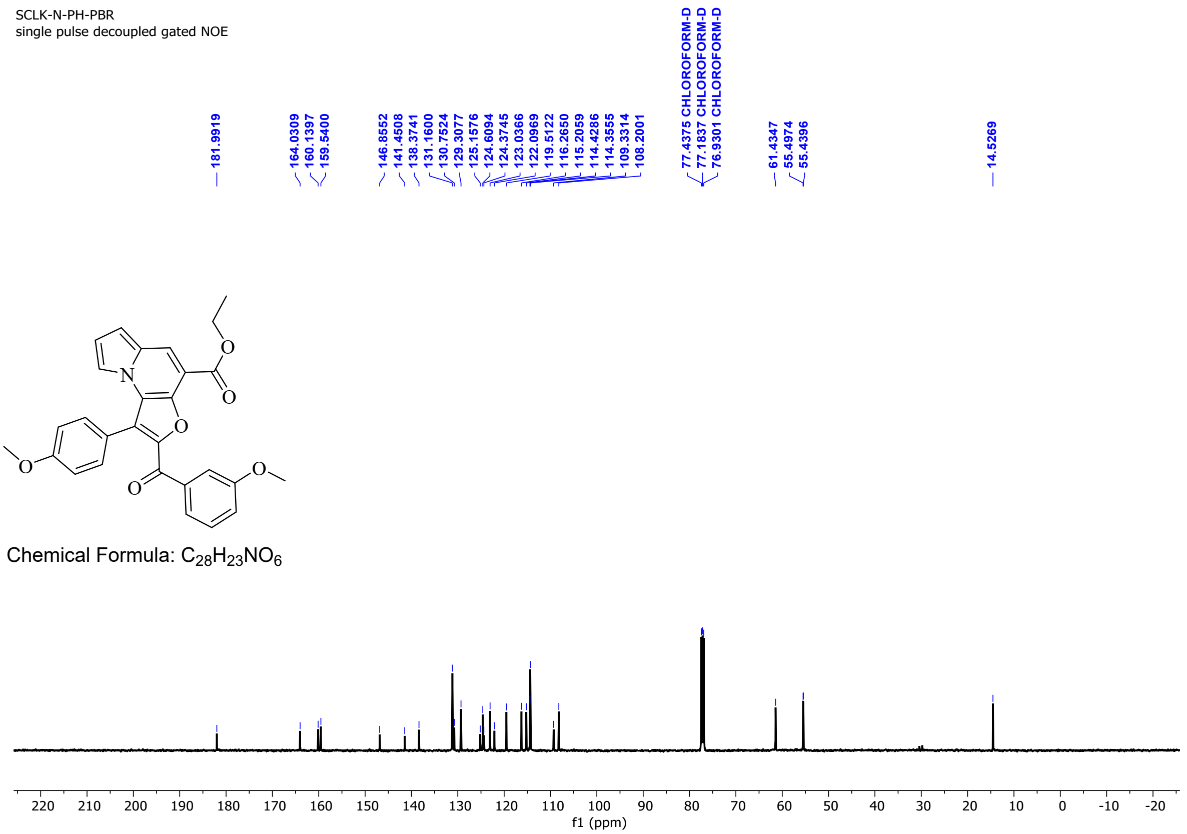

Figure S90. **^{13}C NMR Spectrum of Ethyl 2-(3-methoxybenzoyl)-1-(4methoxyphenyl)furo[3,2-*e*]indolizine-4-carboxylate (6o)**

38. Ethyl 2-([1,1'-biphenyl]-4-carbonyl)-1-(4-methoxyphenyl)furo[3,2-*e*]indolizine-4 -Carboxylate (6p)

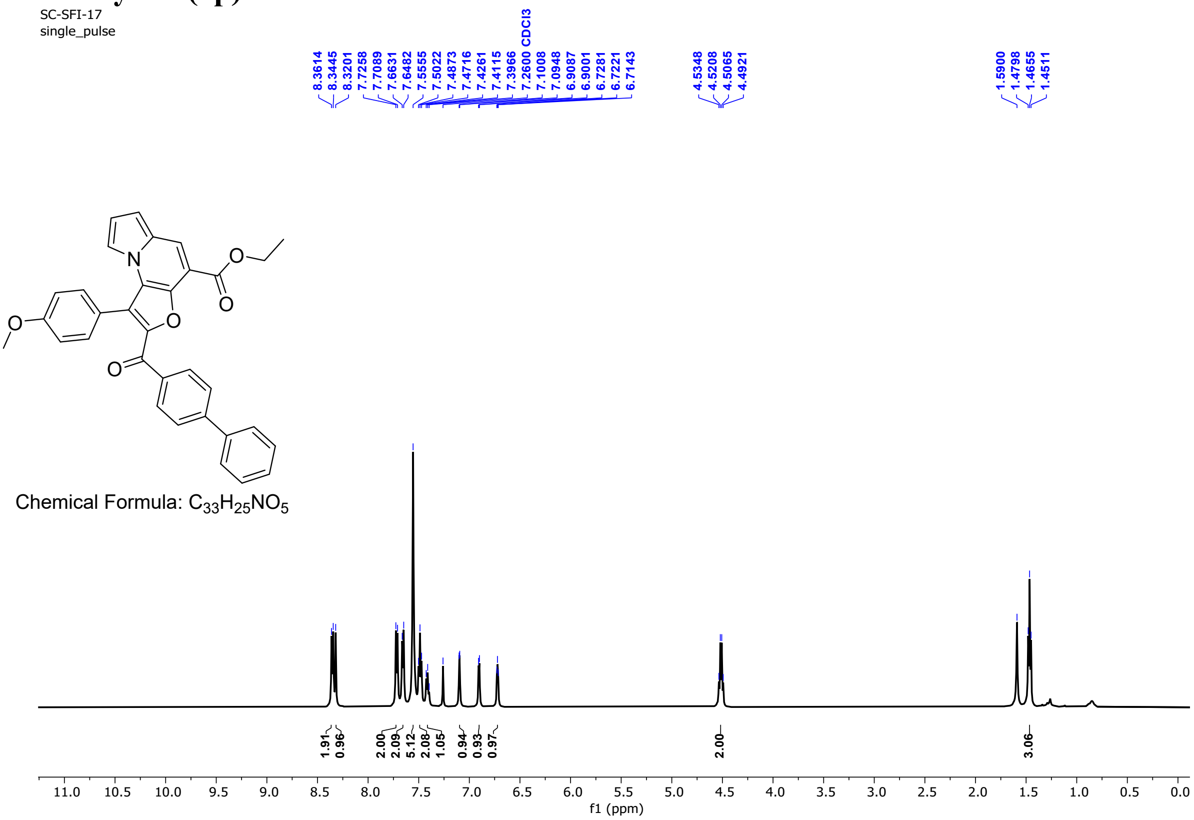

Figure S91. **^{1}H NMR Spectrum of Ethyl 2-([1,1'-biphenyl]-4-carbonyl)-1-(4-methoxyphenyl)furo [3,2-*e*]indolizine-4-Carboxylate (6p)**

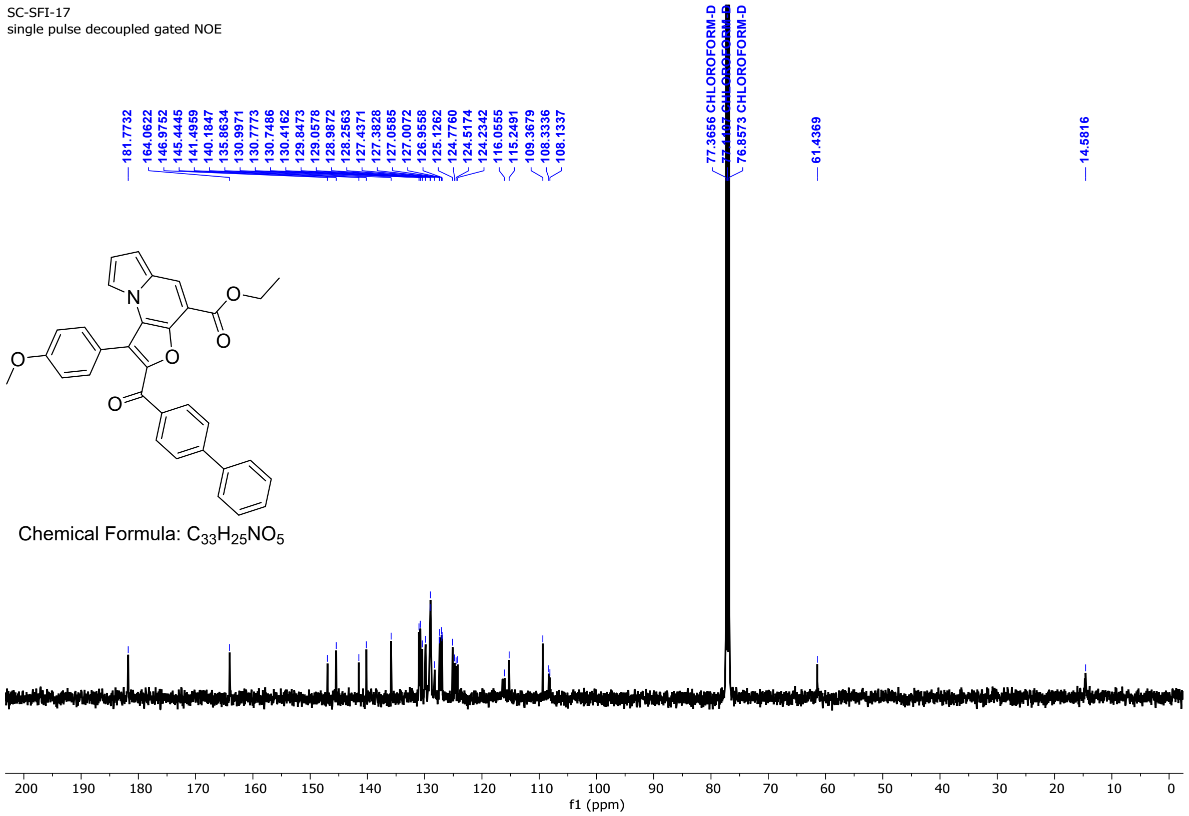

Figure S92. ^{13}C **NMR Spectrum of Ethyl 2-([1,1'-biphenyl]-4-carbonyl)-1-(4-methoxyphenyl)furo [3,2-*e*]indolizine-4-Carboxylate (6p)**

39. Ethyl 1-(4-methoxyphenyl)-2-(3-nitrobenzoyl)furo[3,2-*e*]indolizine-4-carboxylate (6q)

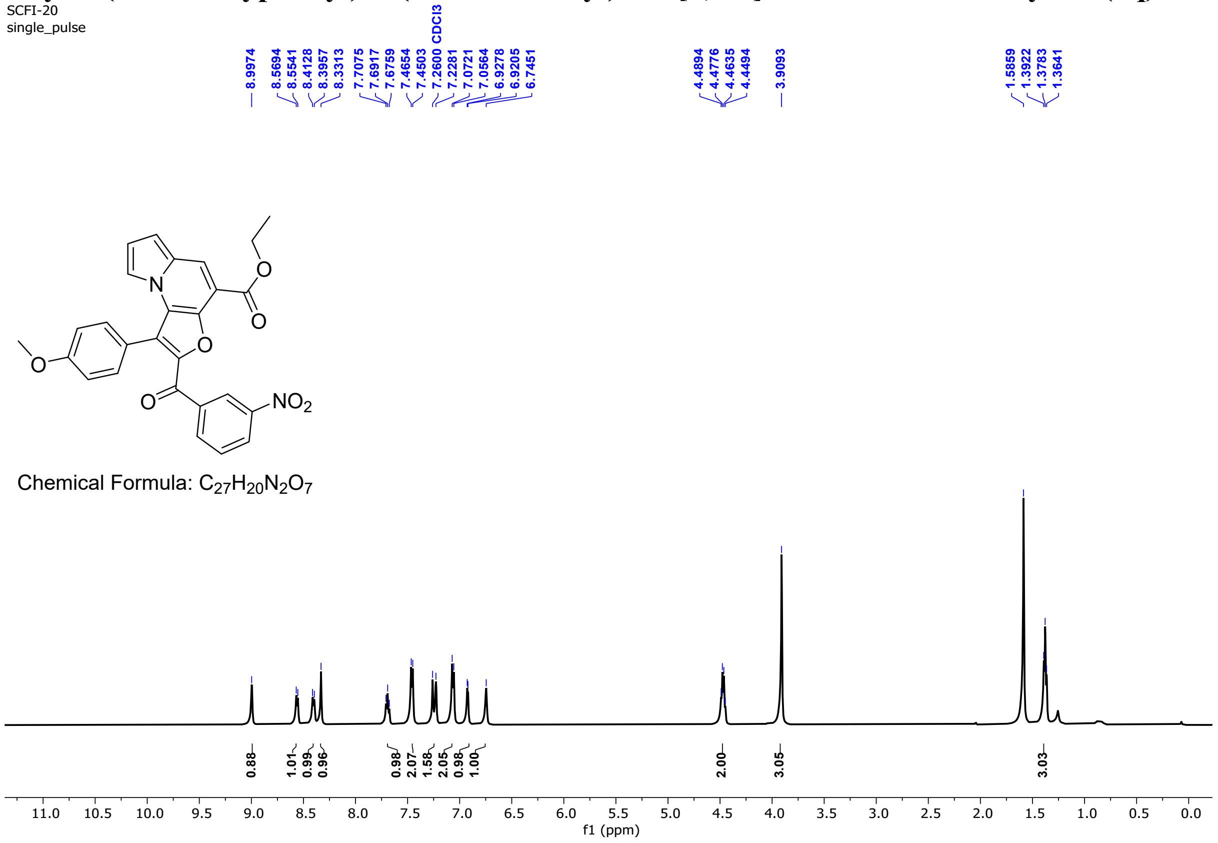

Figure S93. ^{1}H **NMR Spectrum of Ethyl 1-(4-methoxyphenyl)-2-(3-nitrobenzoyl)furo[3,2-*e*]indolizine-4-carboxylate (6q)**

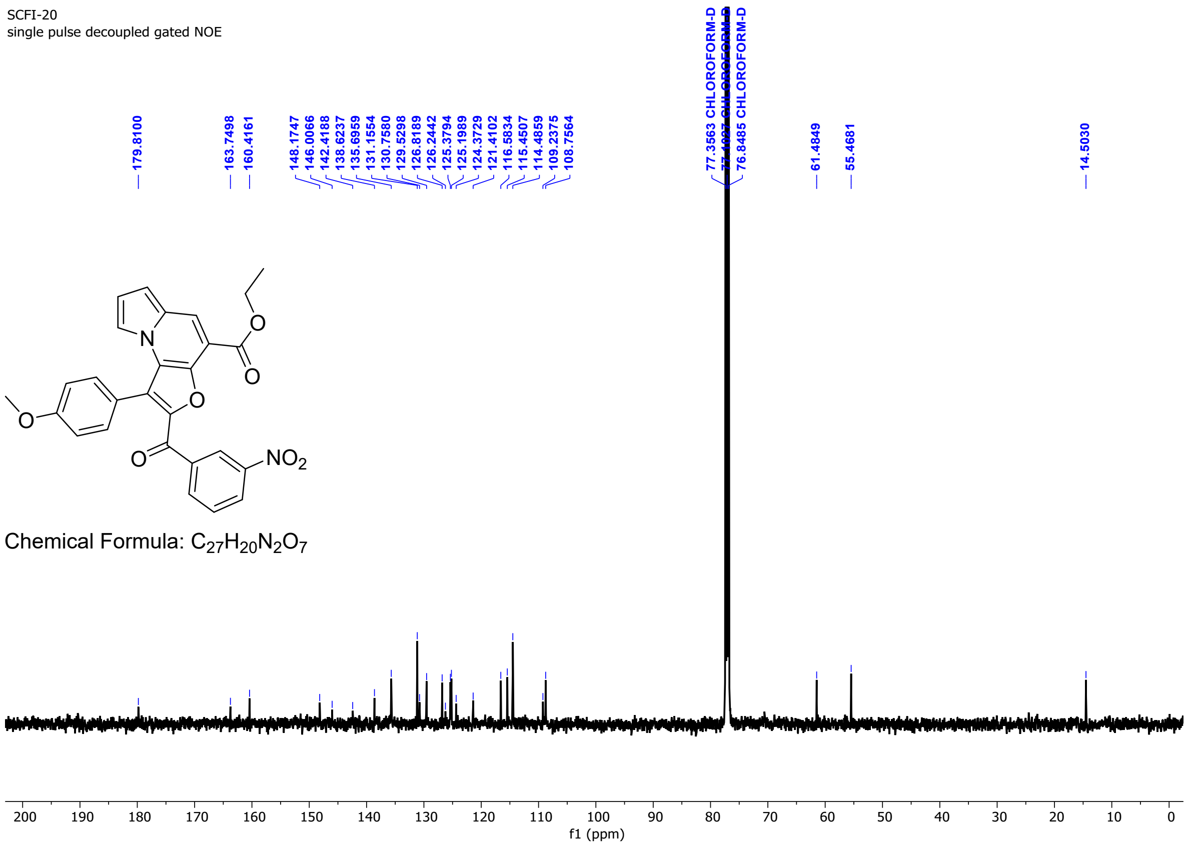

Figure S94. ^{13}C NMR Spectrum of Ethyl 1-(4-methoxyphenyl)-2-(3-nitrobenzoyl)furo[3,2-*e*]indolizine-4-carboxylate (6q)

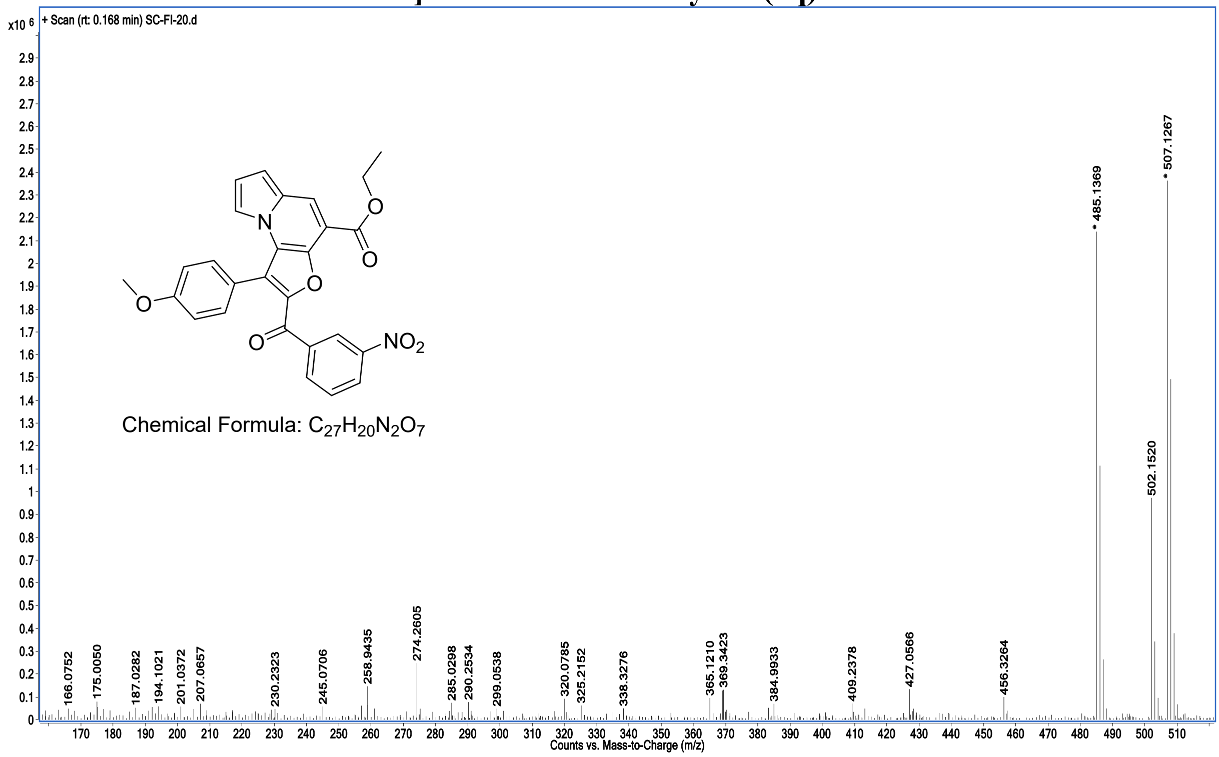

Figure S95. HRMS Spectrum of Ethyl 1-(4-methoxyphenyl)-2-(3-nitrobenzoyl)furo[3,2-*e*]indolizine-4-carboxylate (6q)

40. Ethyl 2-(3-bromobenzoyl)-1-(4-methoxyphenyl)furo[3,2-*e*]indolizine-4-carboxylate (6r)

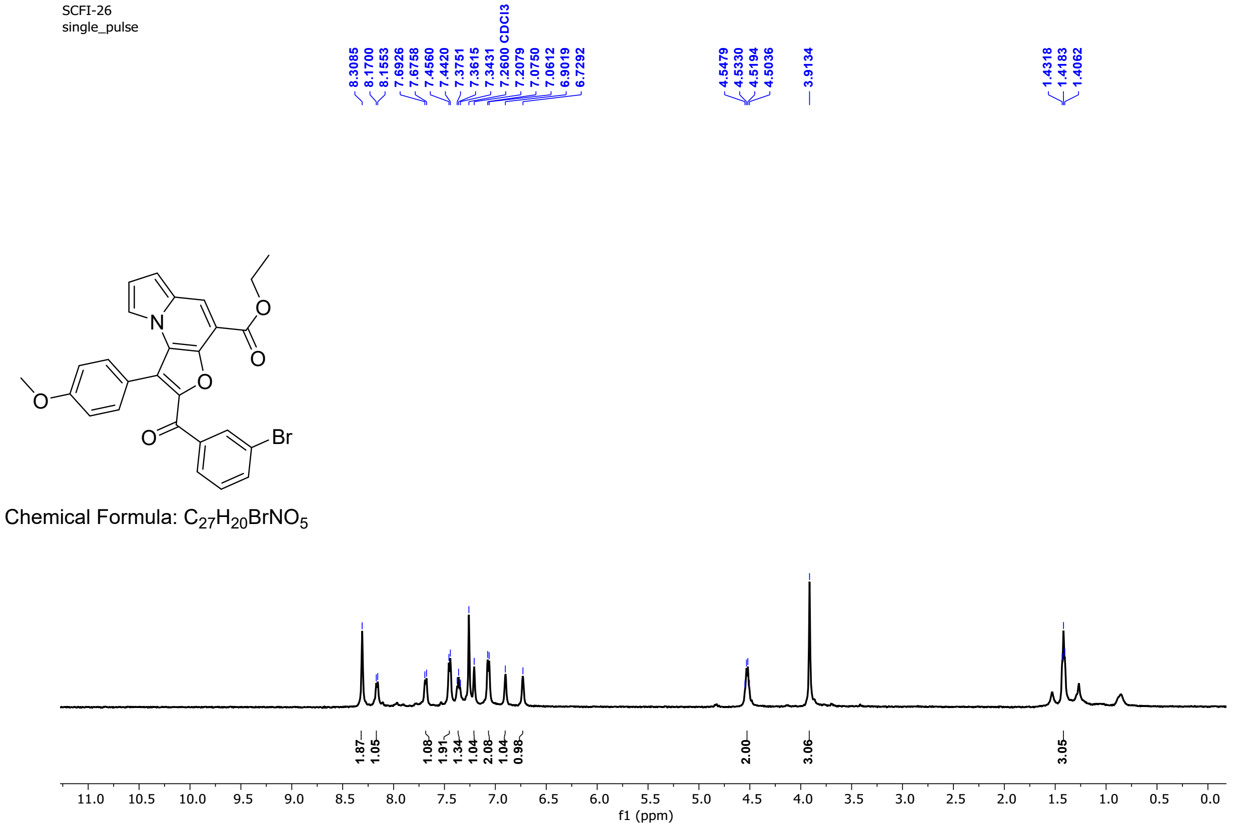

Figure S96. ^{1}H NMR Spectrum of Ethyl 2-(3-bromobenzoyl)-1-(4-methoxyphenyl)furo[3,2-*e*]indolizine-4-carboxylate (6r)

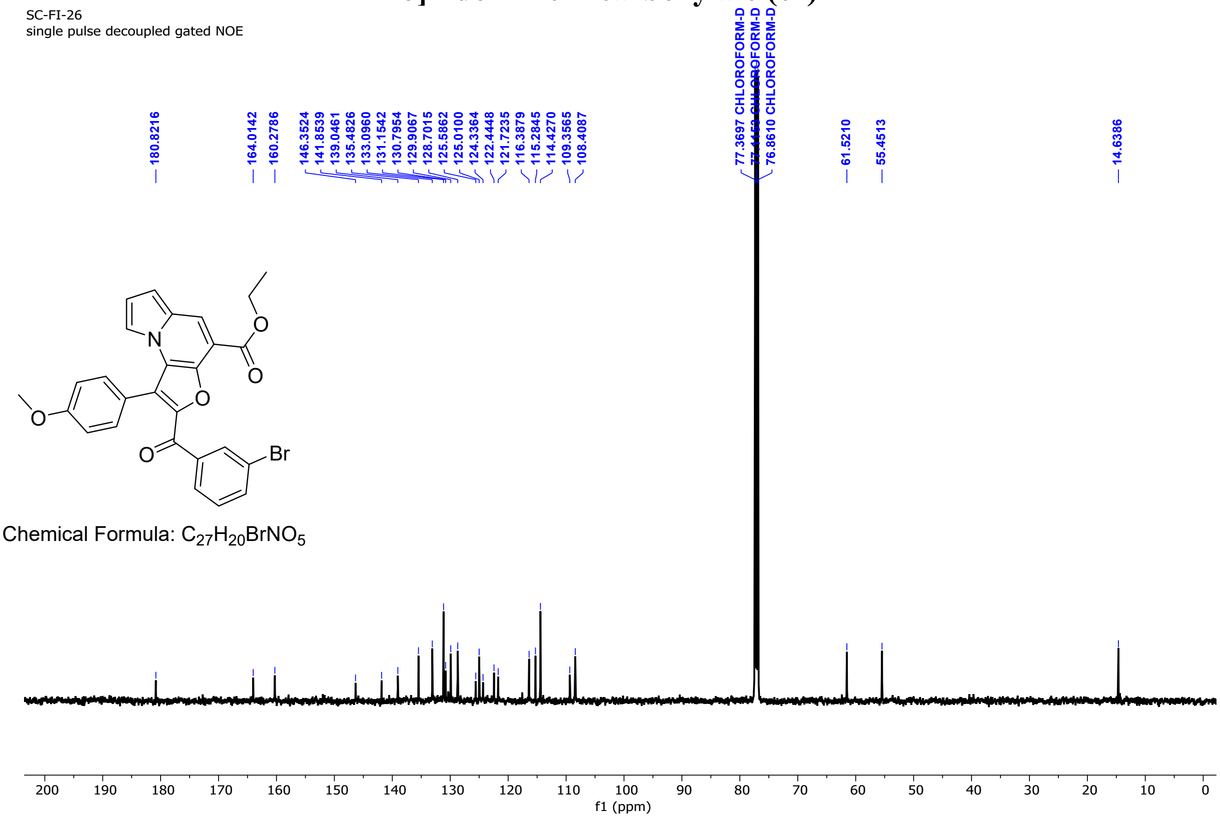

Figure S97. ^{13}C NMR Spectrum of Ethyl 2-(3-bromobenzoyl)-1-(4-methoxyphenyl)furo[3,2-*e*]indolizine-4-carboxylate (6r)

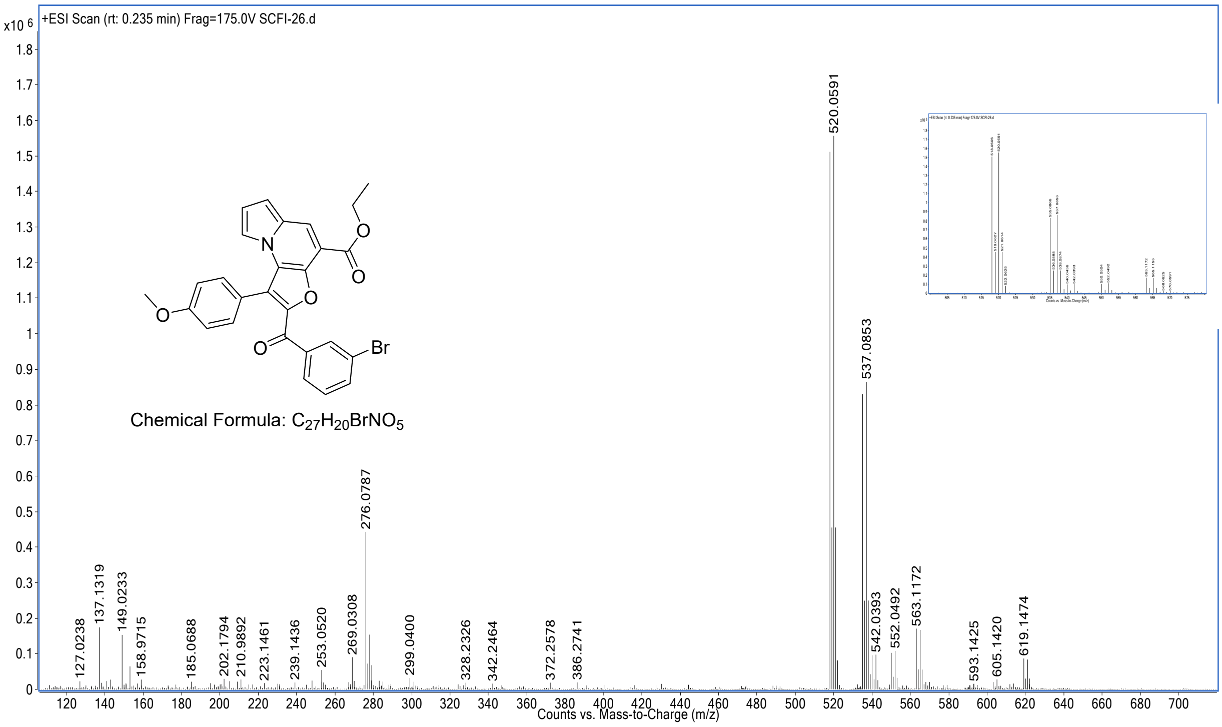

Figure S98. HRMS Spectrum of Ethyl 2-(3-bromobenzoyl)-1-(4-methoxyphenyl)furo[3,2-*e*]indolizine-4-carboxylate (6r)

41. Ethyl 2-(3,4-dichlorobenzoyl)-1-(4-methoxyphenyl)furo[3,2-*e*]indolizine-4-carboxylate (6s)

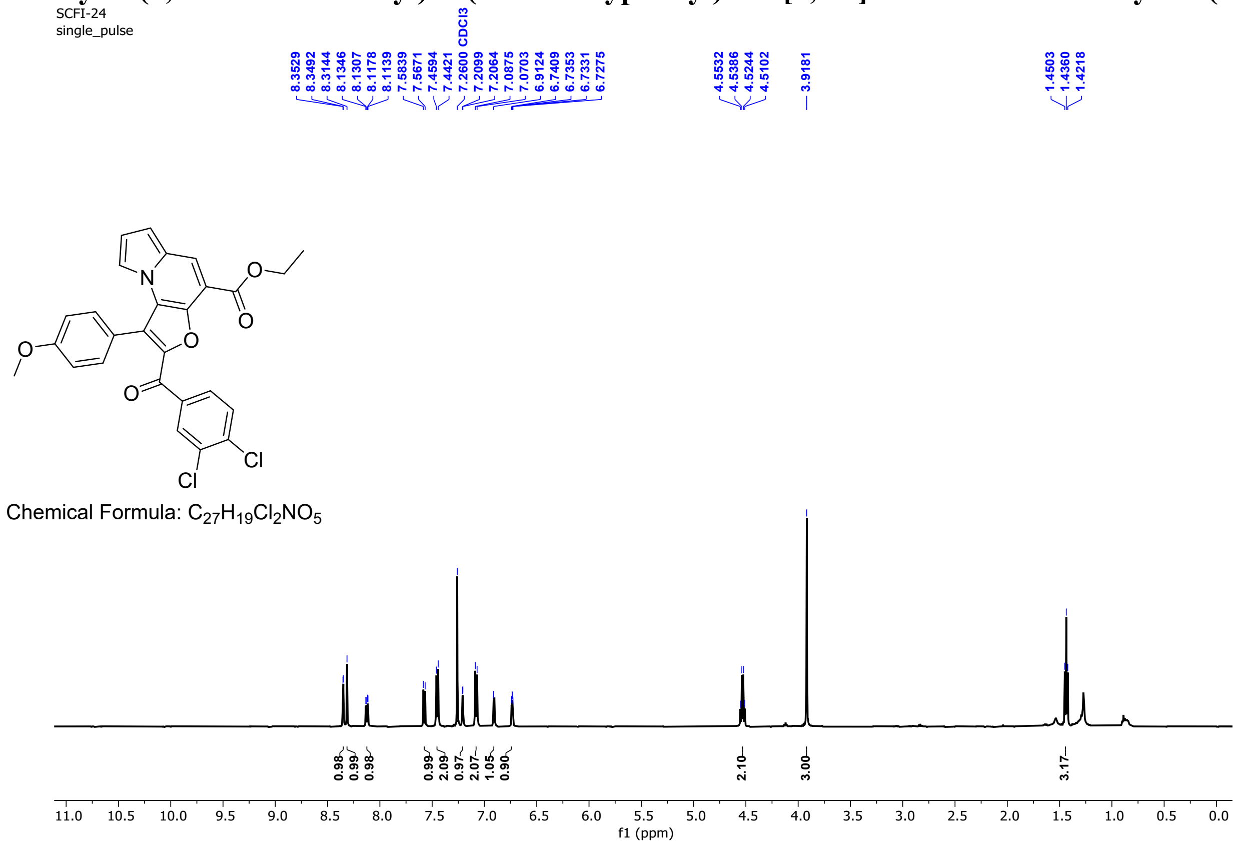

Figure S99. ^{1}H NMR Spectrum of Ethyl 2-(3,4-dichlorobenzoyl)-1-(4-methoxyphenyl)furo[3,2-*e*]indolizine-4-carboxylate (6s)

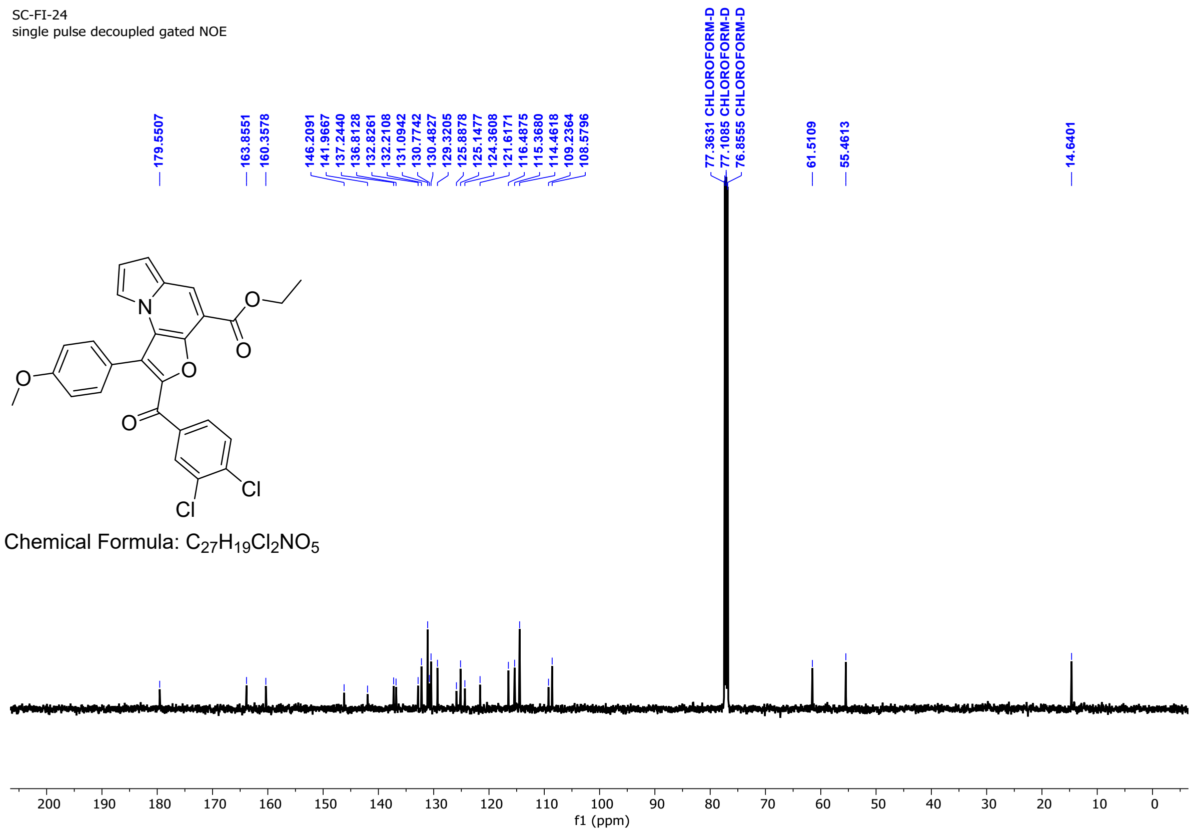

Figure S100. ^{13}C NMR Spectrum of Ethyl 2-(3,4-dichlorobenzoyl)-1-(4-methoxyphenyl)furo[3,2-*e*]indolizine-4-carboxylate (6s)

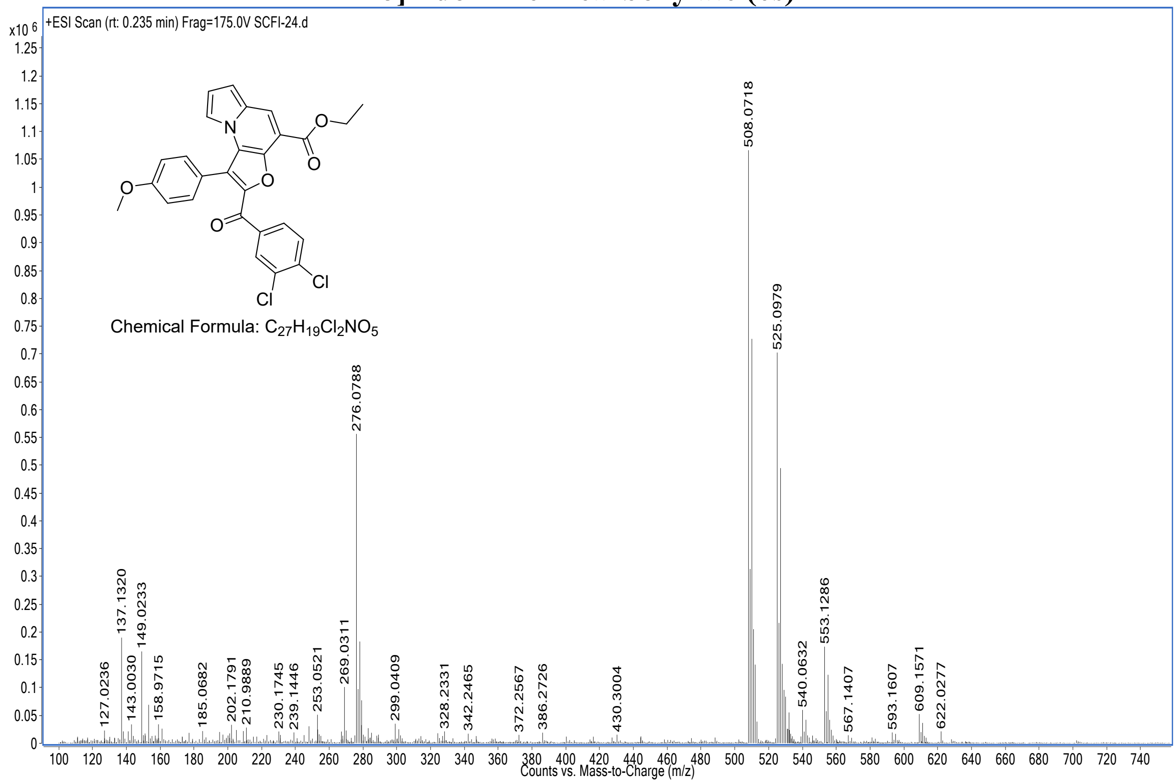

Figure S101. HRMS Spectrum of Ethyl 2-(3,4-dichlorobenzoyl)-1-(4-methoxyphenyl)furo[3,2-*e*]indolizine-4-carboxylate (6s)

42. Ethyl 2-(3,5-bis(trifluoromethyl)benzoyl)-1-(4-methoxyphenyl)furo[3,2-*e*]indolizine-4-carboxylate (6t)

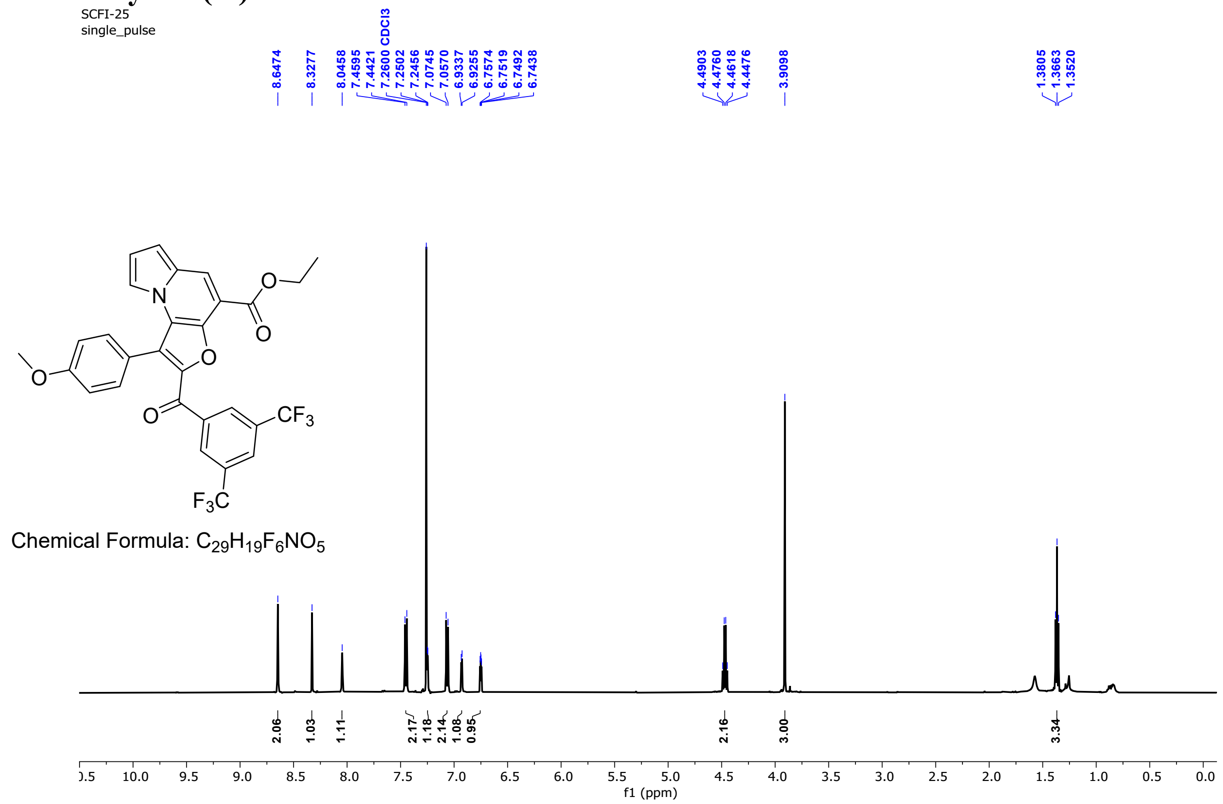

Figure S102. ^{1}H NMR Spectrum of Ethyl 2-(3,5-bis(trifluoromethyl)benzoyl)-1-(4-methoxyphenyl)furo[3,2-*e*]indolizine-4-carboxylate (6t)

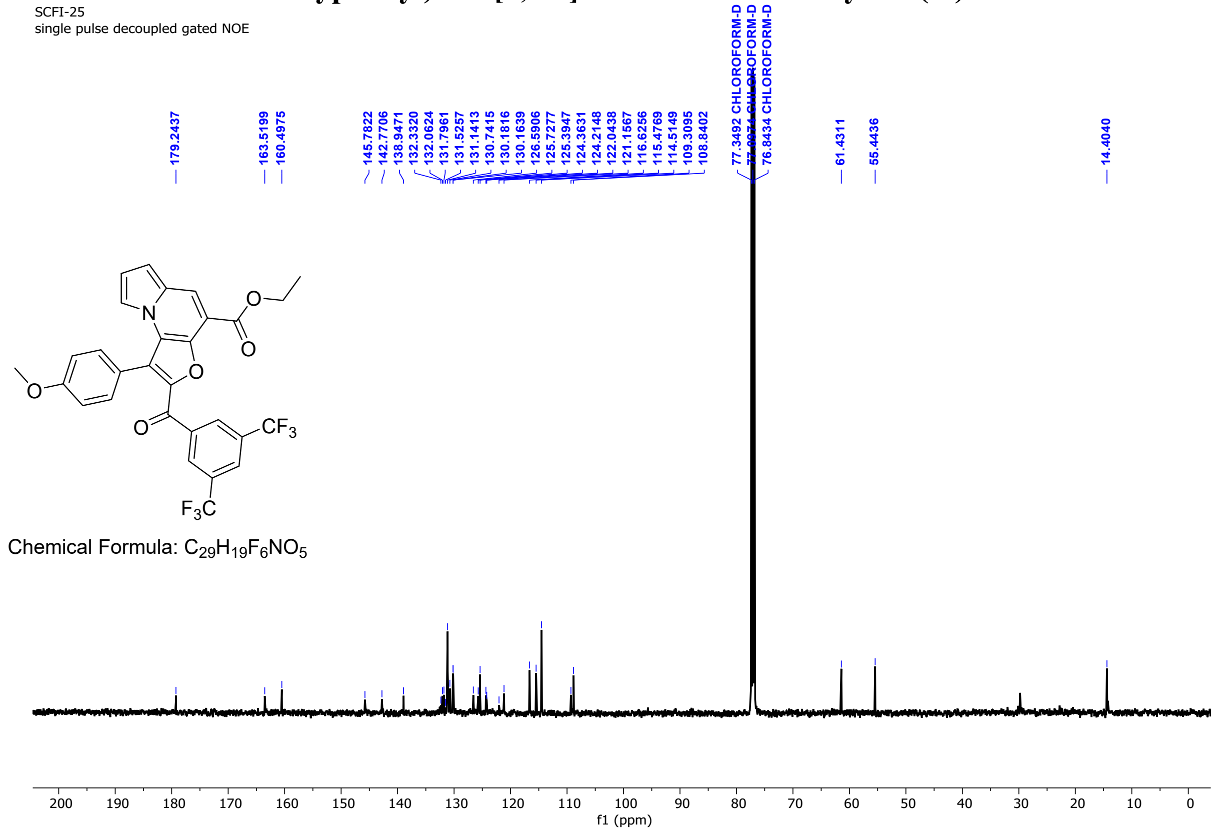

Figure S103. ^{13}C NMR Spectrum of Ethyl 2-(3,5-bis(trifluoromethyl)benzoyl)-1-(4-methoxyphenyl)furo[3,2-*e*]indolizine-4-carboxylate (6t)

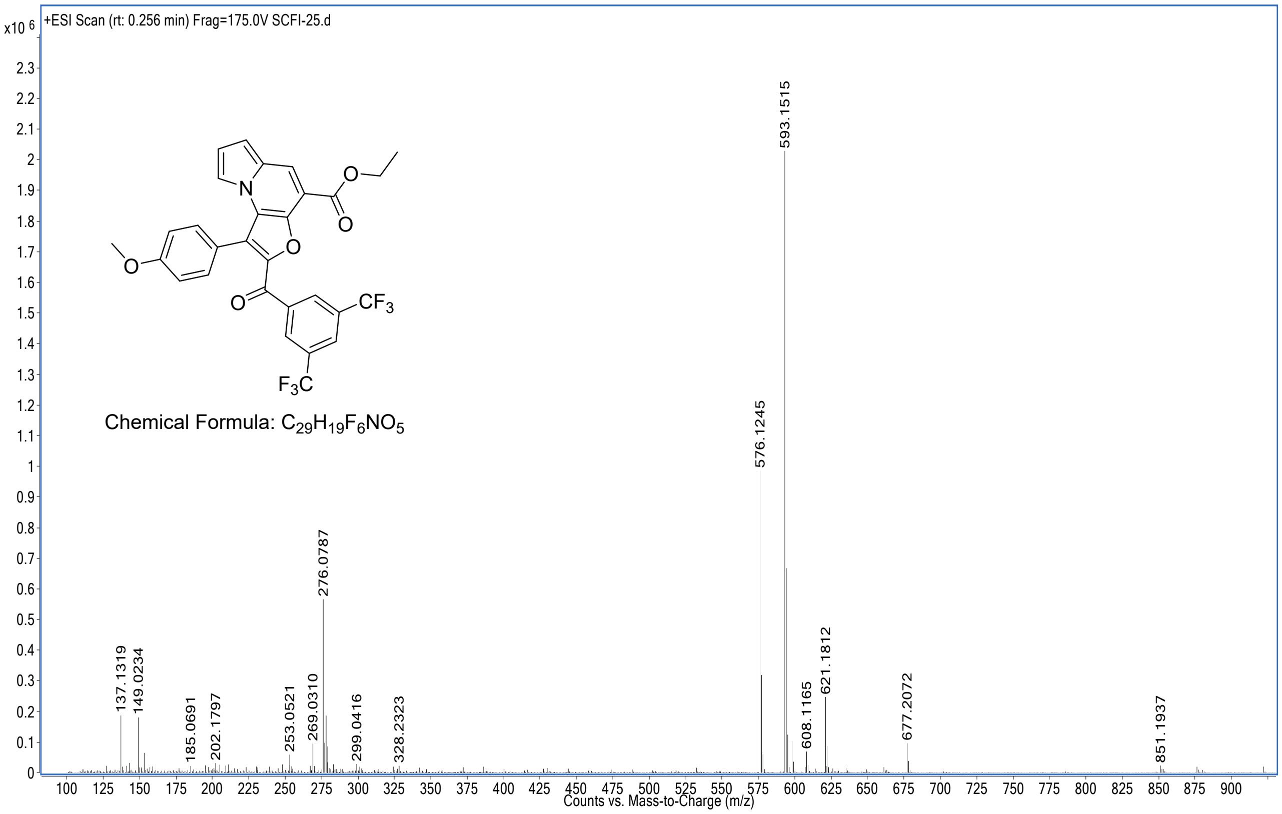

Figure S104. HRMS Spectrum of Ethyl 2-(3,5-bis(trifluoromethyl)benzoyl)-1-(4-methoxyphenyl)furo[3,2-*e*]indolizine-4-carboxylate (6t)

43. Ethyl 2-(2-fluorobenzoyl)-1-(4-methoxyphenyl)furo[3,2-*e*]indolizine-4-carboxylate (6u)

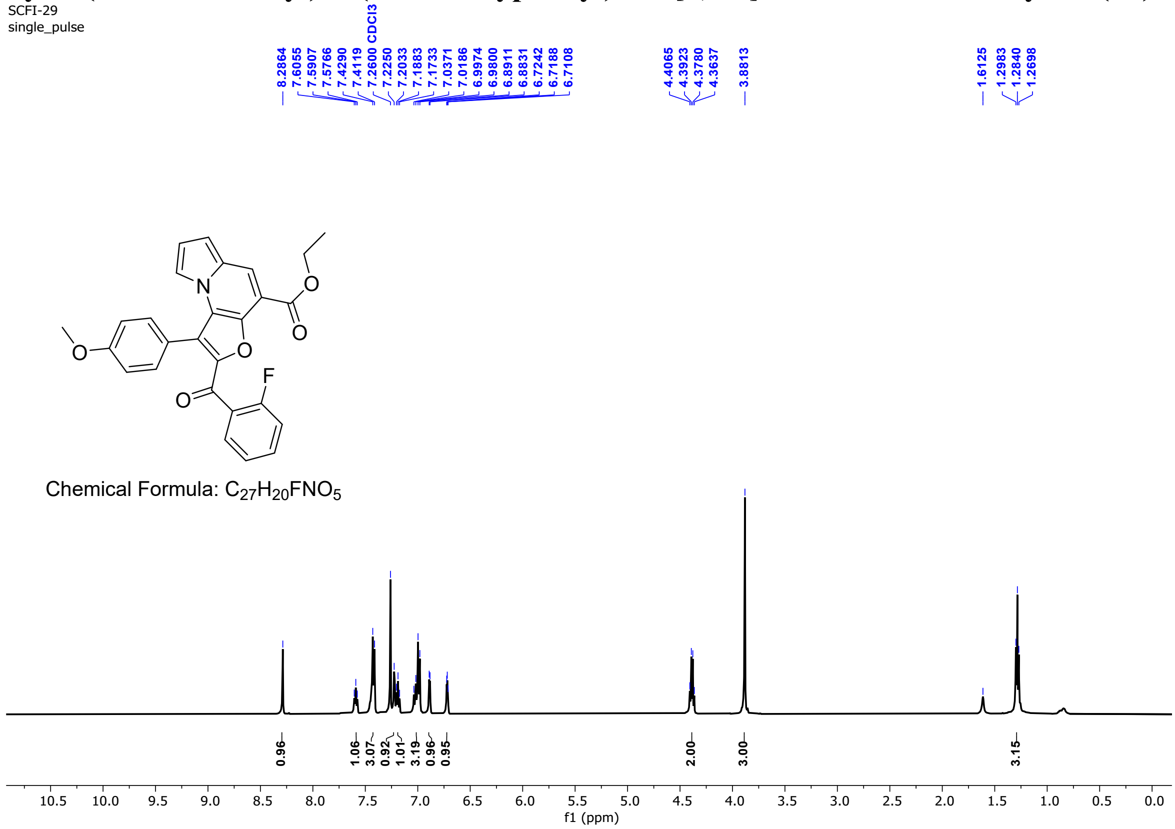

Figure S105. ^{1}H NMR Spectrum of Ethyl 2-(2-fluorobenzoyl)-1-(4-methoxyphenyl)furo[3,2-*e*]indolizine-4-carboxylate (6u)

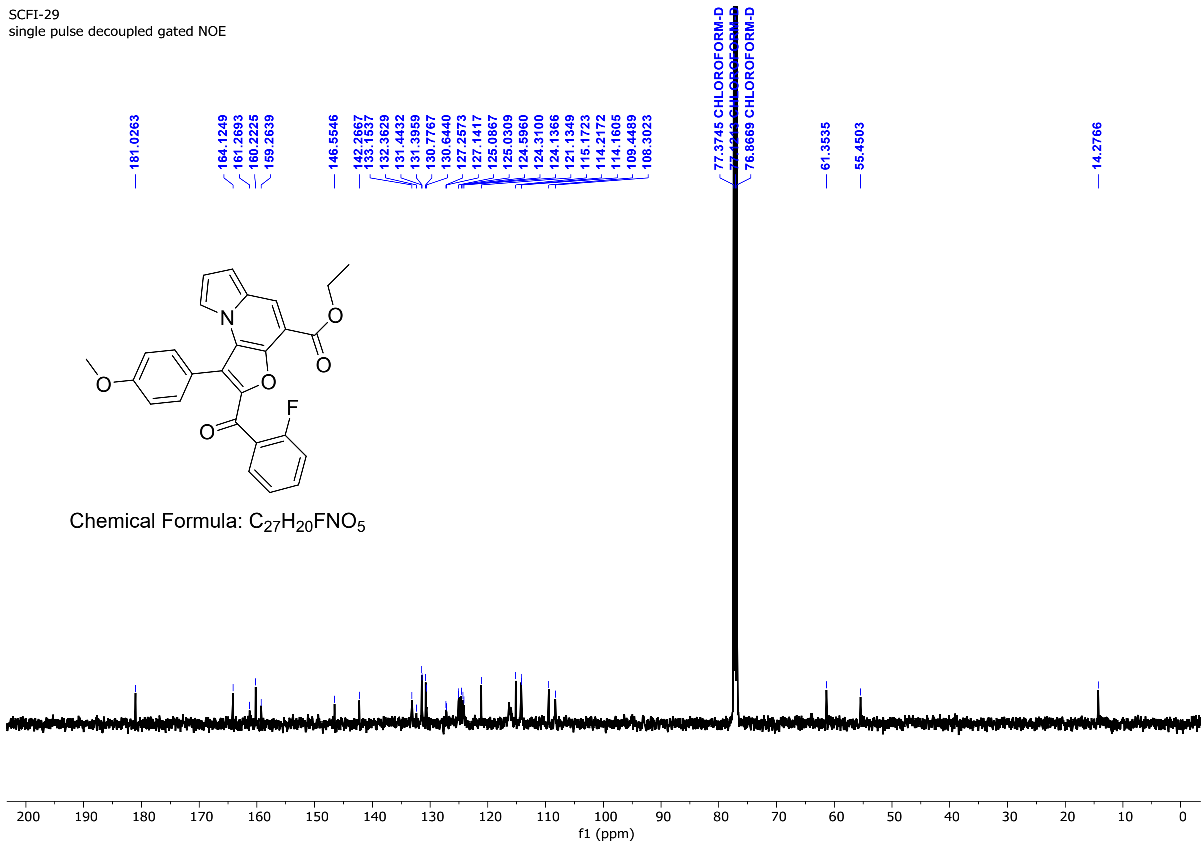

Figure S106. ^{13}**C NMR Spectrum of Ethyl 2-(2-fluorobenzoyl)-1-(4-methoxyphenyl)furo[3,2-*e*]indolizine-4-carboxylate (6u)**

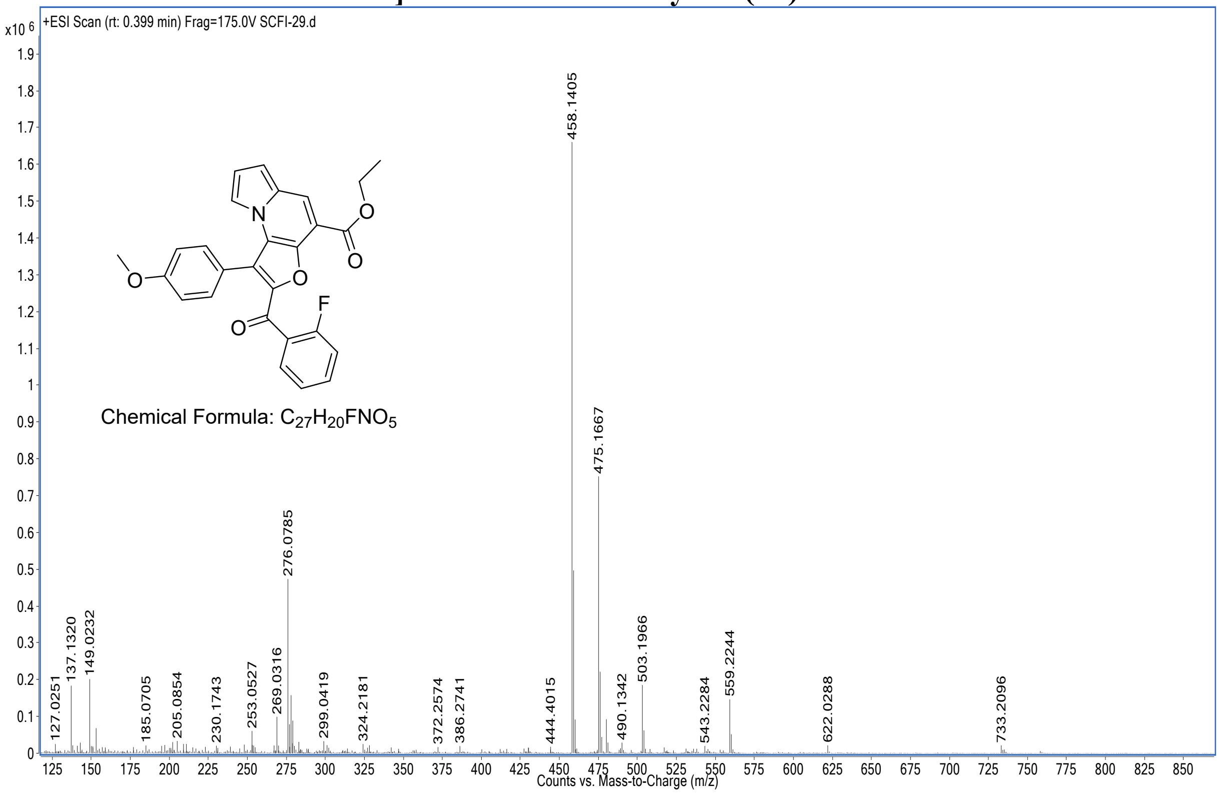

Figure S107. HRMS Spectrum of Ethyl 2-(2-fluorobenzoyl)-1-(4-methoxyphenyl)furo[3,2-*e*]indolizine-4-carboxylate (6u)

44. Ethyl 2-(2-methoxybenzoyl)-1-(4-methoxyphenyl)furo[3,2-*e*]indolizine-4-carboxylate (6v)

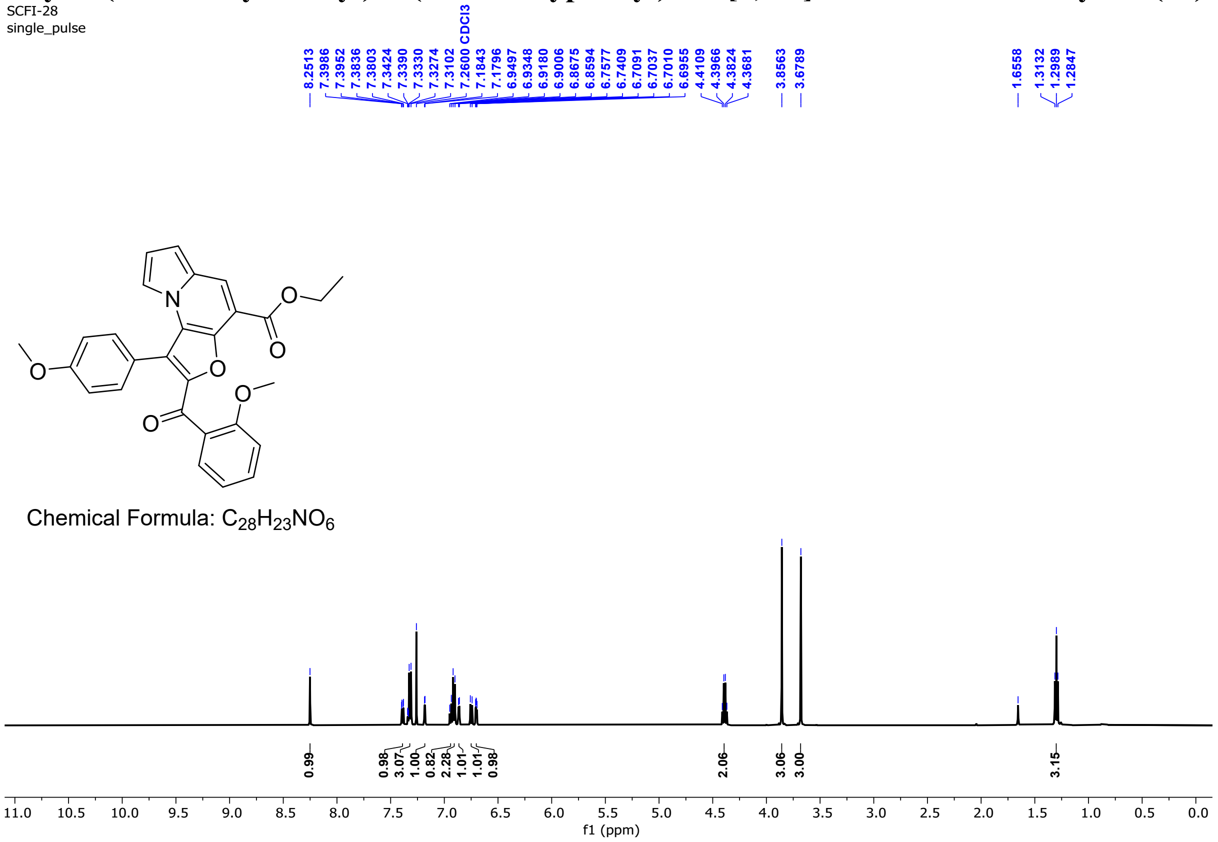

Figure S108. ¹H NMR Spectrum of Ethyl 2-(2-methoxybenzoyl)-1-(4-methoxyphenyl)furo[3,2-*e*]indolizine-4-carboxylate (6v)

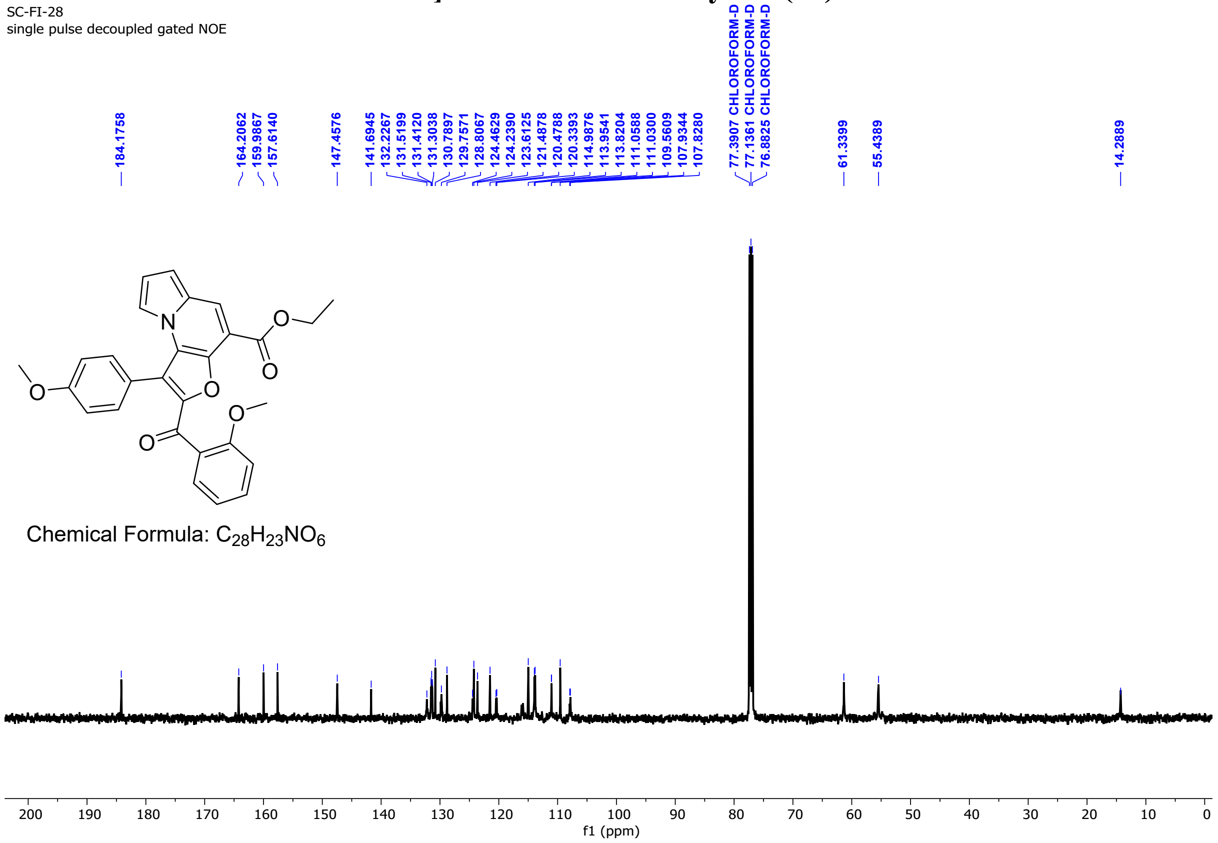

Figure S109. ¹³C NMR Spectrum of Ethyl 2-(2-methoxybenzoyl)-1-(4-methoxyphenyl)furo[3,2-*e*]indolizine-4-carboxylate (6v)

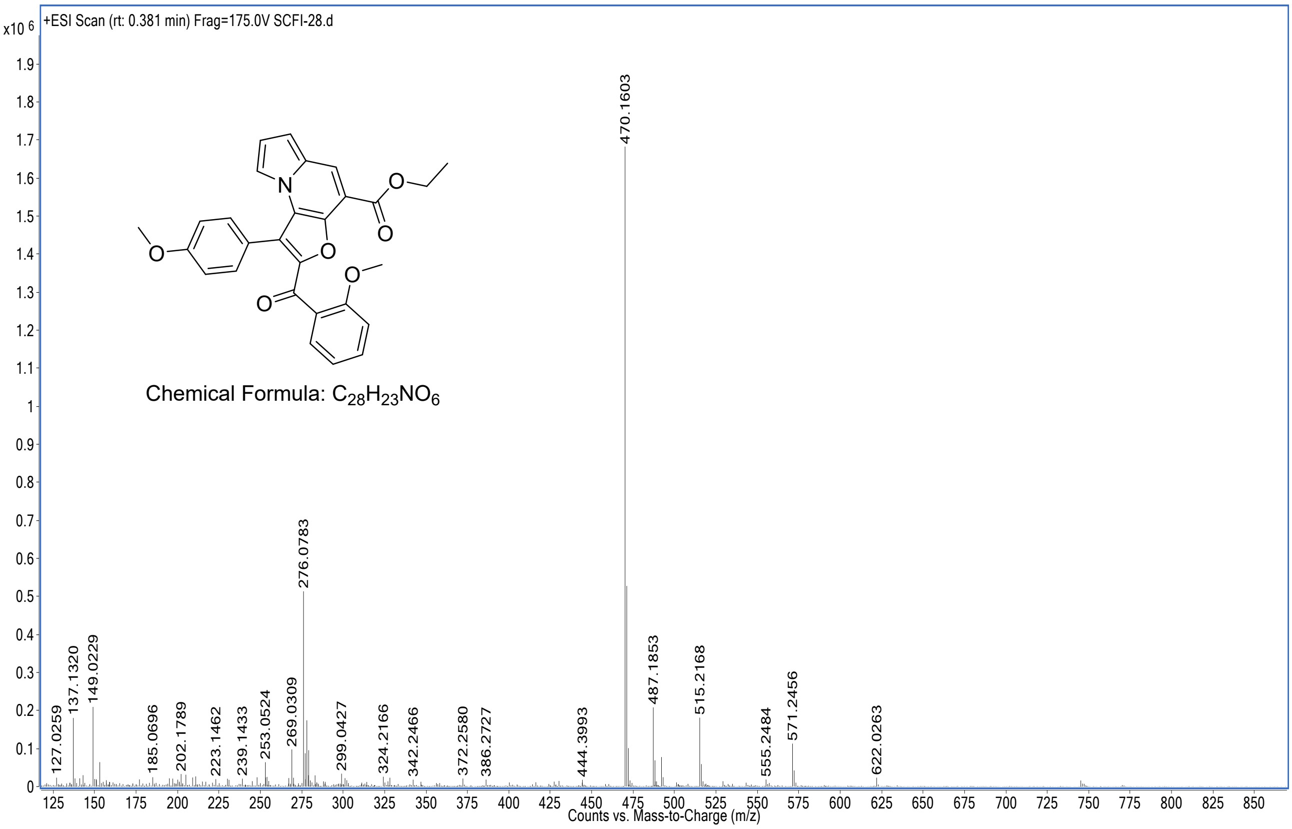

Figure S110. HRMS Spectrum of Ethyl 2-(2-methoxybenzoyl)-1-(4-methoxyphenyl)furo[3,2-*e*]indolizine-4-carboxylate (6v)

45. Ethyl 2-(3-chlorobenzoyl)-1-(3-methoxyphenyl)furo[3,2-*e*]indolizine-4-carboxylate (6w)

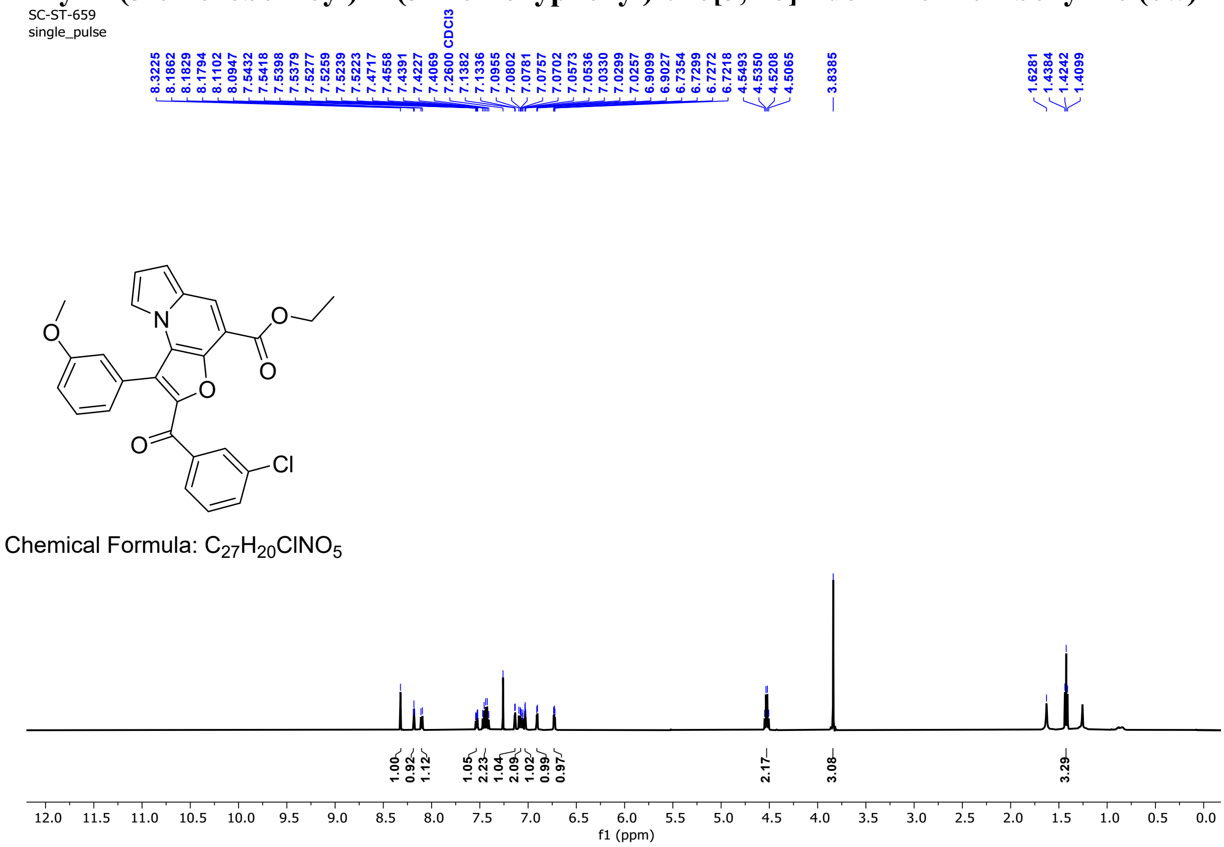

Figure S111. ^{1}H NMR Spectrum of Ethyl 2-(3-chlorobenzoyl)-1-(3-methoxyphenyl)furo[3,2-*e*]indolizine-4-carboxylate (6w)

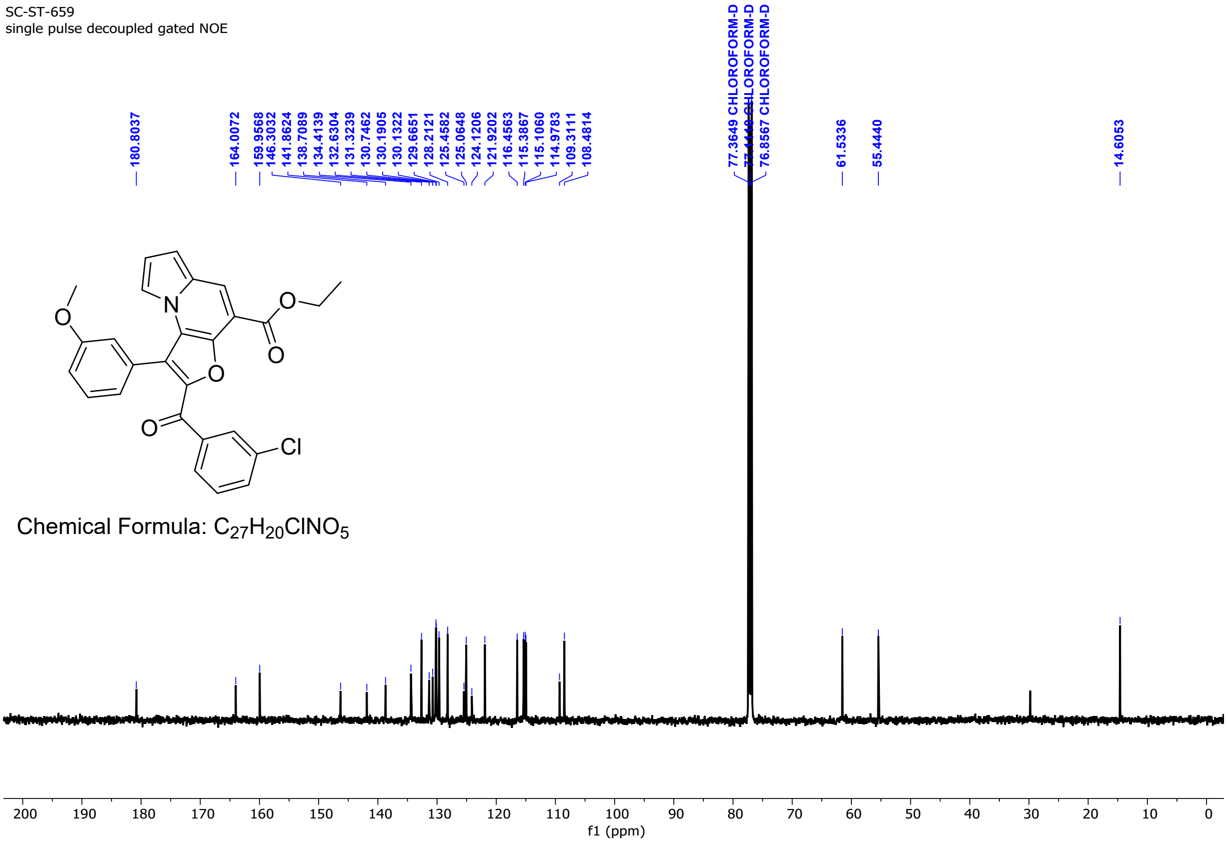

Figure S112. ^{13}C NMR Spectrum of Ethyl 2-(3-chlorobenzoyl)-1-(3-methoxyphenyl)furo[3,2-*e*]indolizine-4-carboxylate (6w)

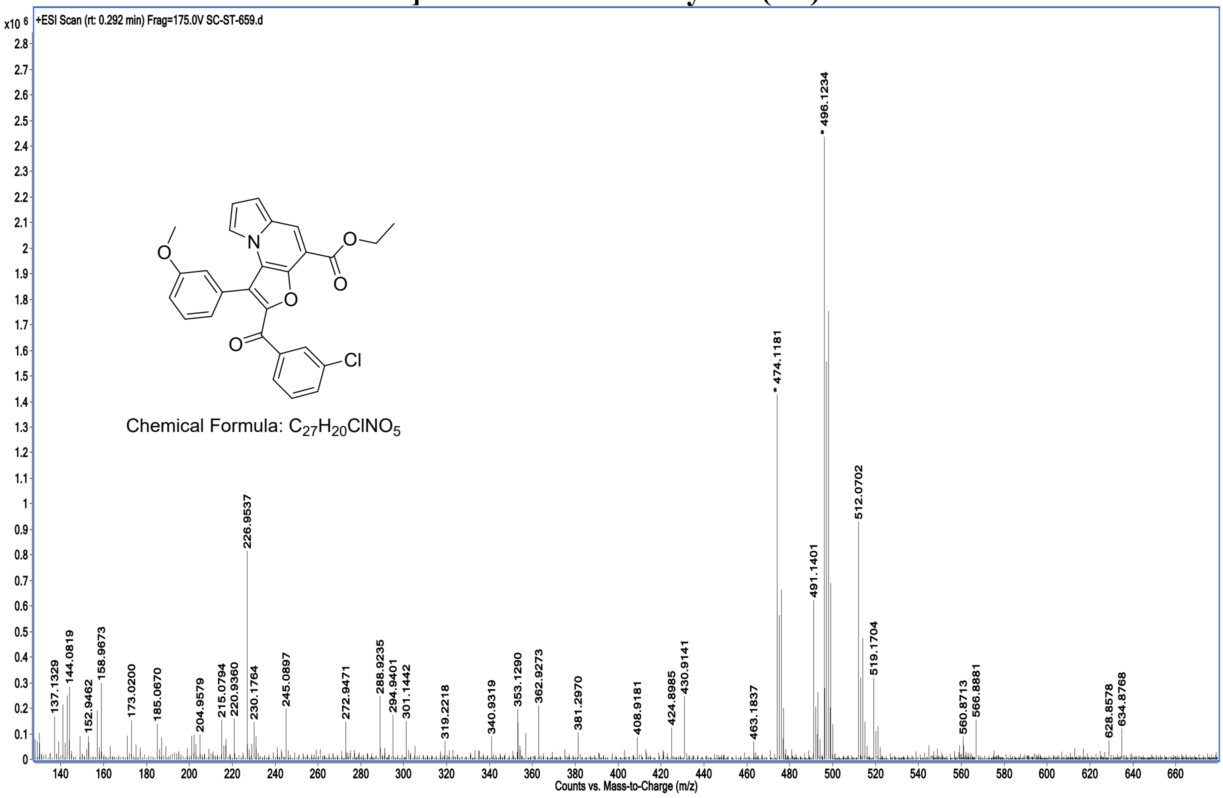

Figure S113. HRMS Spectrum of Ethyl 2-(3-chlorobenzoyl)-1-(3-methoxyphenyl)furo[3,2-*e*]indolizine-4-carboxylate (6w)

46. Ethyl 2-(4-bromobenzoyl)-1-(4-(trifluoromethoxy)phenyl)furo[3,2-*e*]indolizine-4-carboxylate (6x)

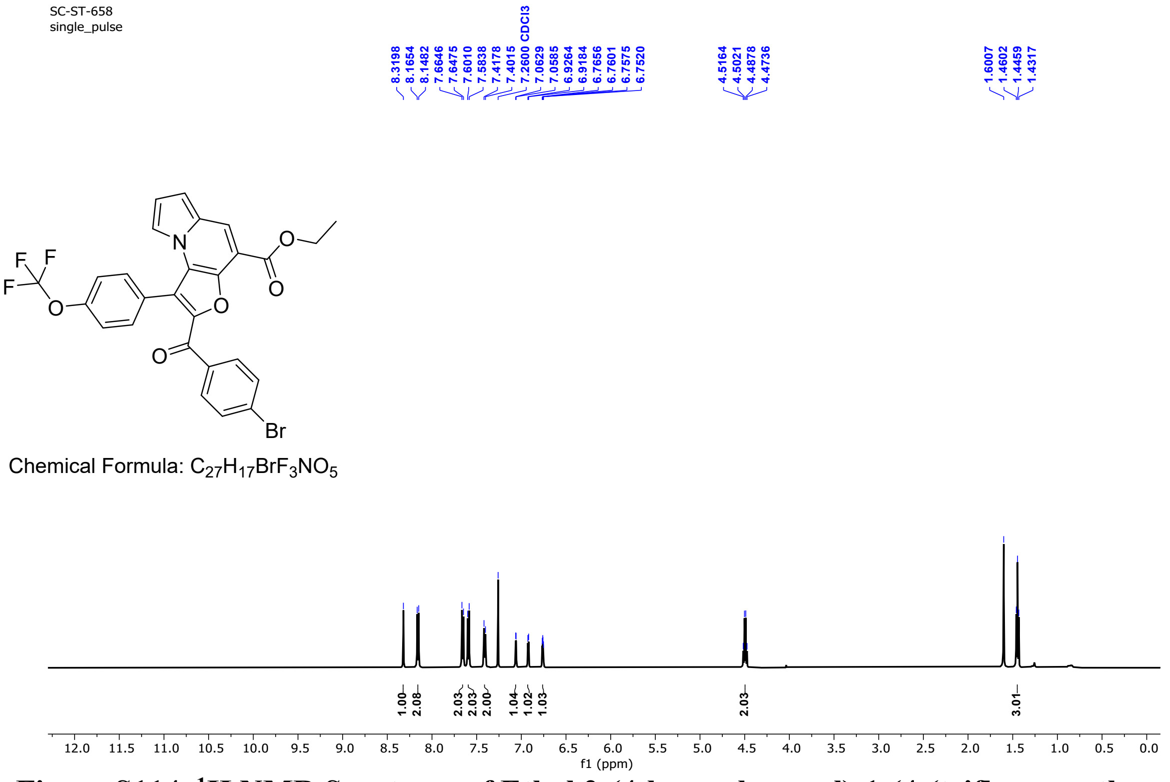

Figure S114. ^{1}H NMR Spectrum of Ethyl 2-(4-bromobenzoyl)-1-(4-(trifluoromethoxy)phenyl) furo[3,2-*e*]indolizine-4-carboxylate (6x)

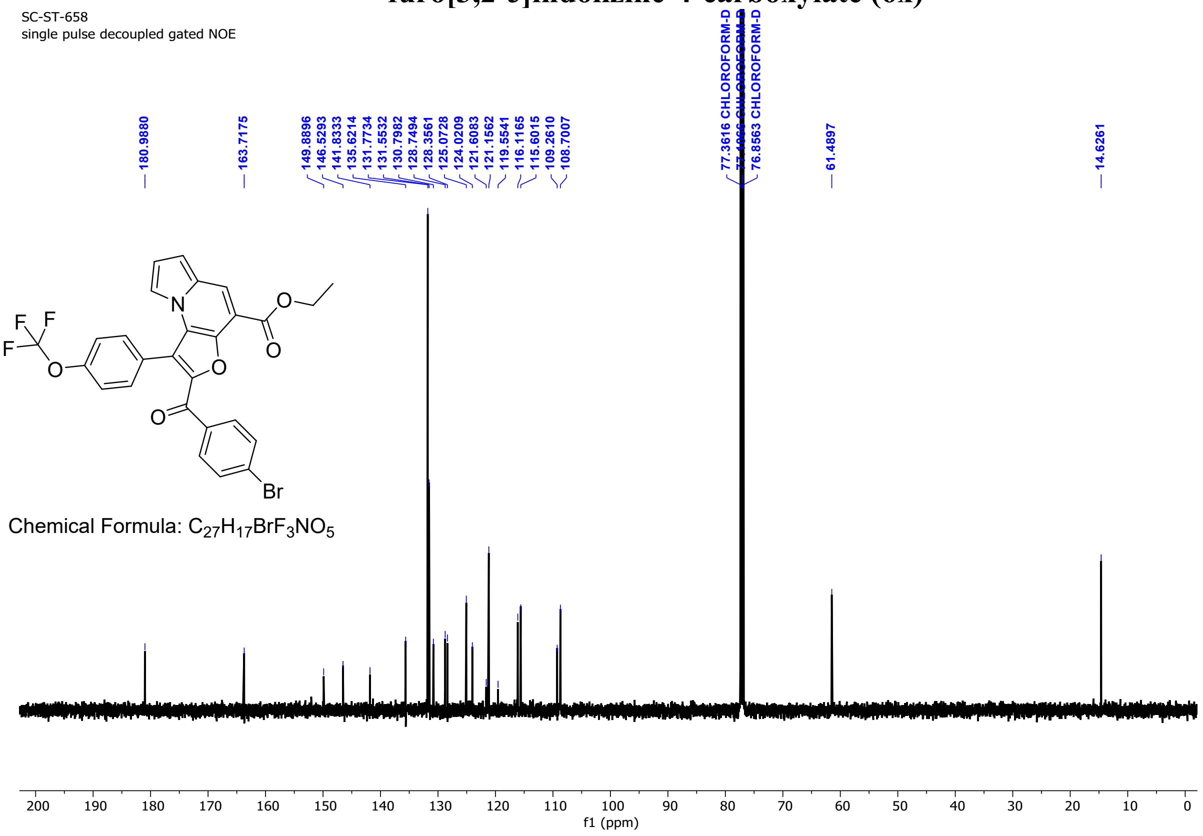

Figure S115. ^{13}C NMR Spectrum of Ethyl 2-(4-bromobenzoyl)-1-(4-(trifluoromethoxy)phenyl) furo[3,2-*e*]indolizine-4-carboxylate (6x)

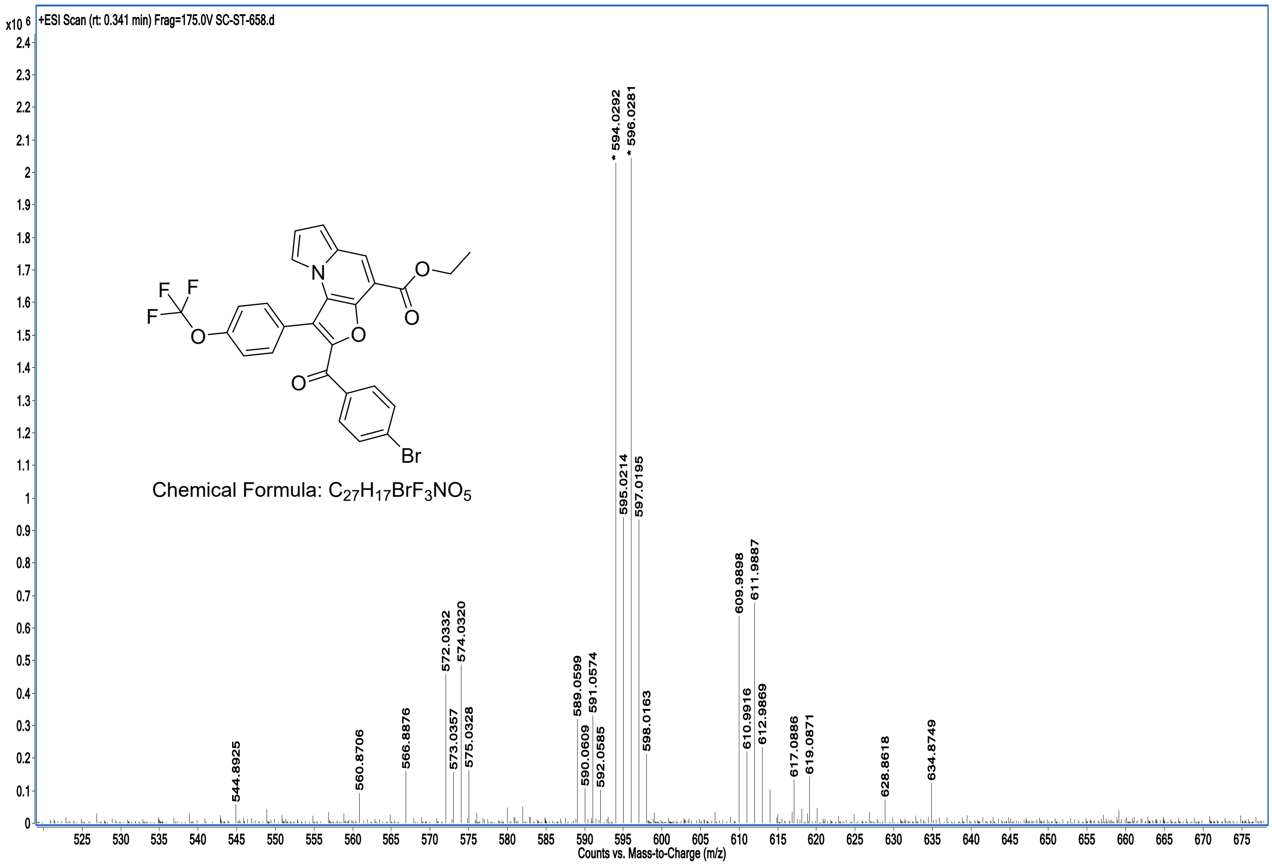

Figure S116. HRMS Spectrum of Ethyl 2-(4-bromobenzoyl)-1-(4-(trifluoromethoxy)phenyl) furo[3,2-*e*]indolizine-4-carboxylate (6x)

47. Ethyl 2-(3-bromobenzoyl)-1-(naphthalen-1-yl)furo[3,2-*e*]indolizine-4-carboxylate (6y)

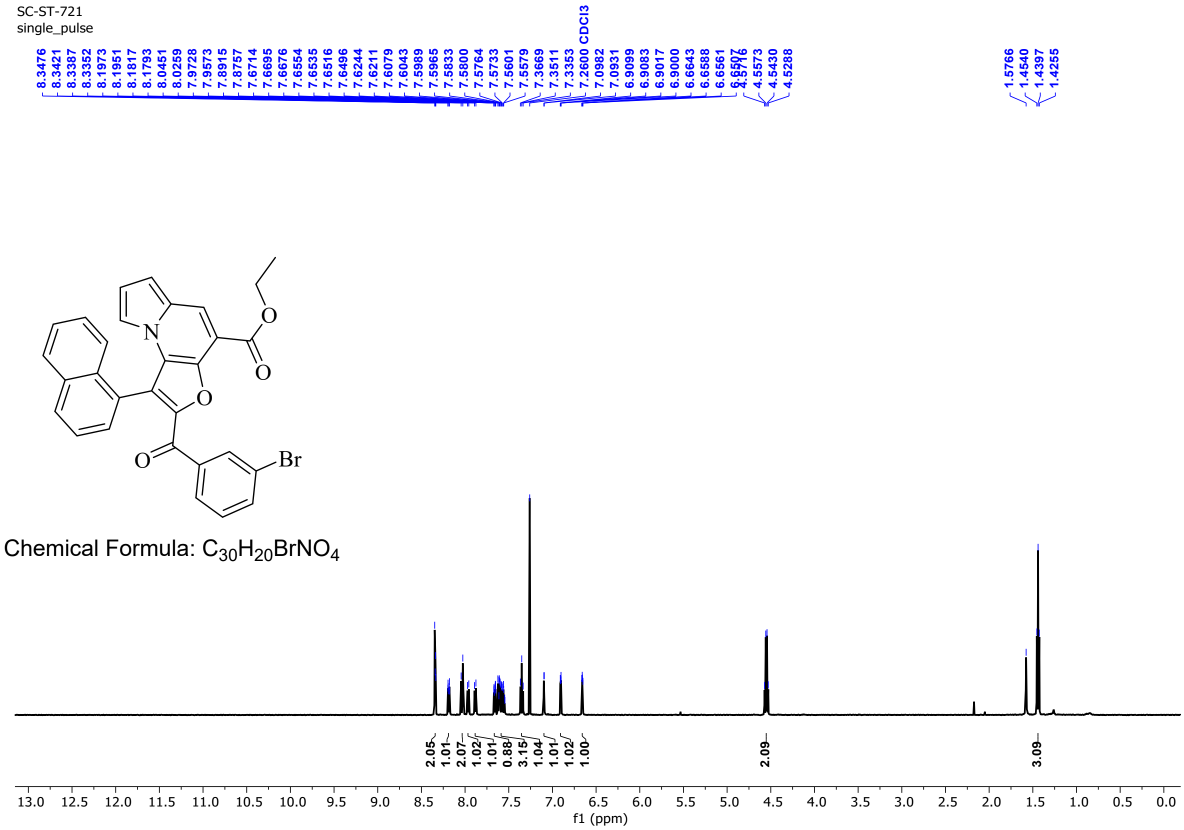

Figure S117. ^{1}H NMR Spectrum of Ethyl 2-(3-bromobenzoyl)-1-(naphthalen-1-yl)furo

[3,2 *e*]indolizine-4-carboxylate (6y)

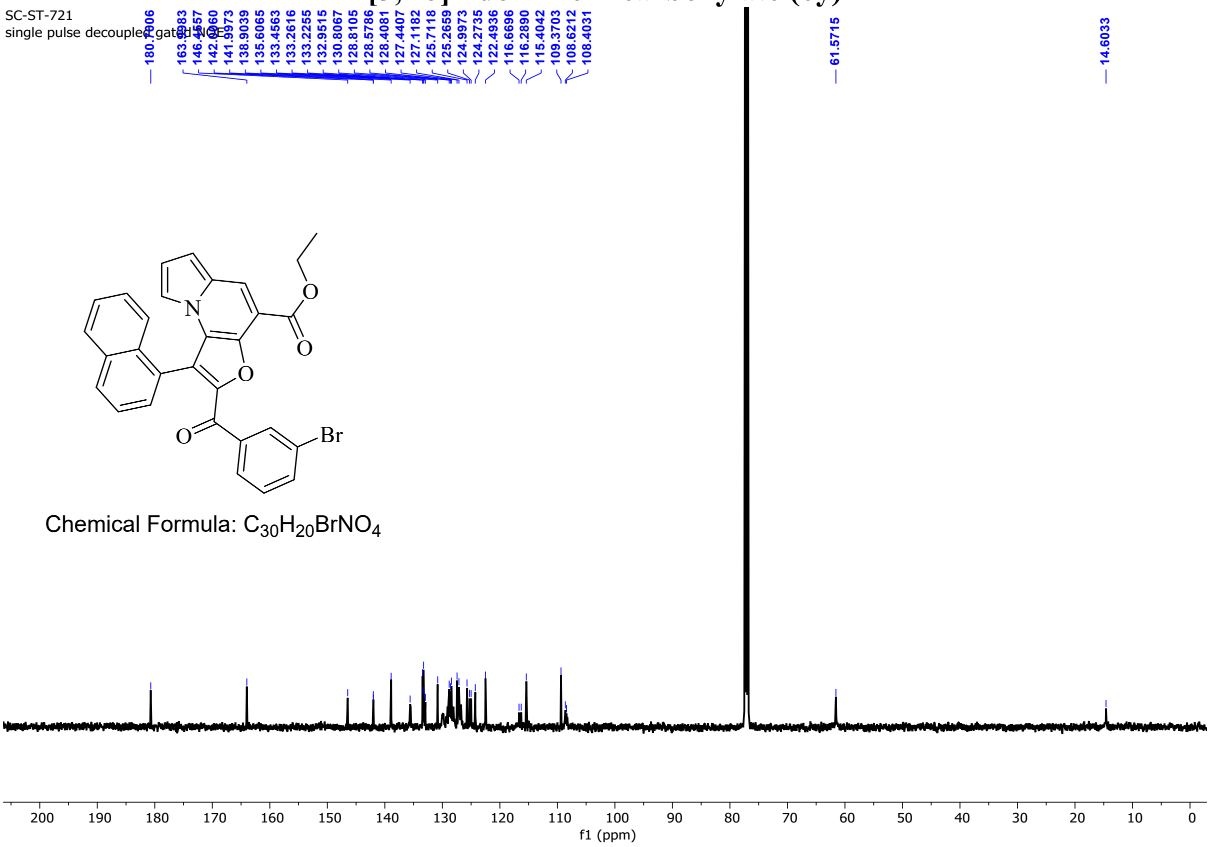

Figure S118. ^{13}**C NMR Spectrum of Ethyl 2-(3-bromobenzoyl)-1-(naphthalen-1-yl)furo [3,2 *e*]indolizine-4-carboxylate (6y)**

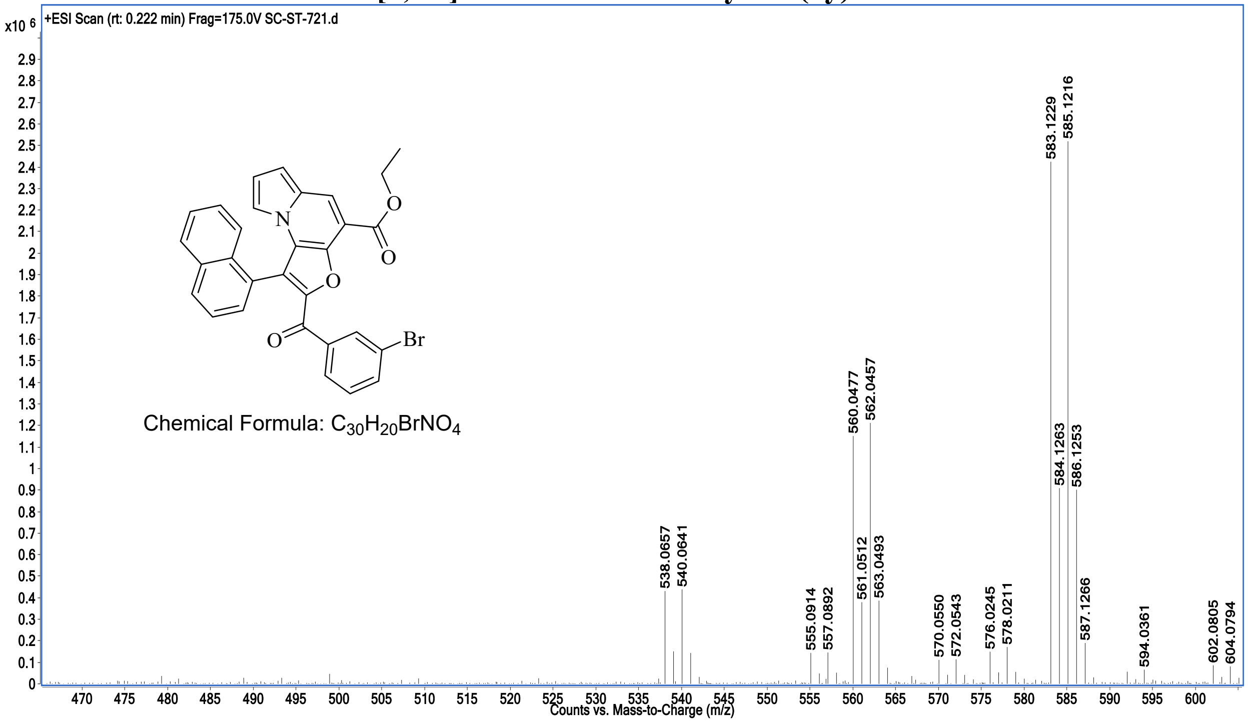

Figure S119. HRMS Spectrum of Ethyl 2-(3-bromobenzoyl)-1-(naphthalen-1-yl)furo [3,2 *e*]indolizine-4-carboxylate (6y)

48. Ethyl 1-(4-bromophenyl)-2-(4-methylbenzoyl)furo[3,2-*e*]indolizine-4-carboxylate (6z)

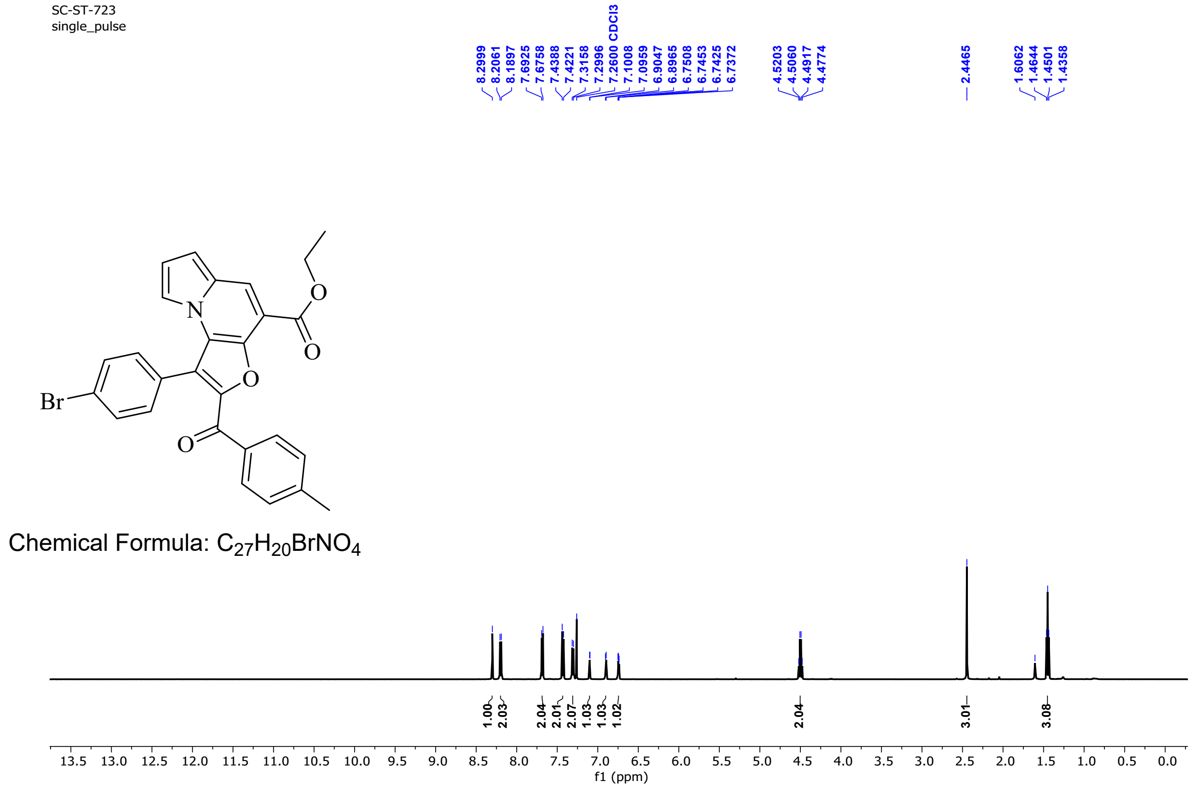

Figure S120. ^{1}H **NMR Spectrum of Ethyl 1-(4-bromophenyl)-2-(4-methylbenzoyl)furo[3,2-*e*]indolizine-4-carboxylate (6z)**

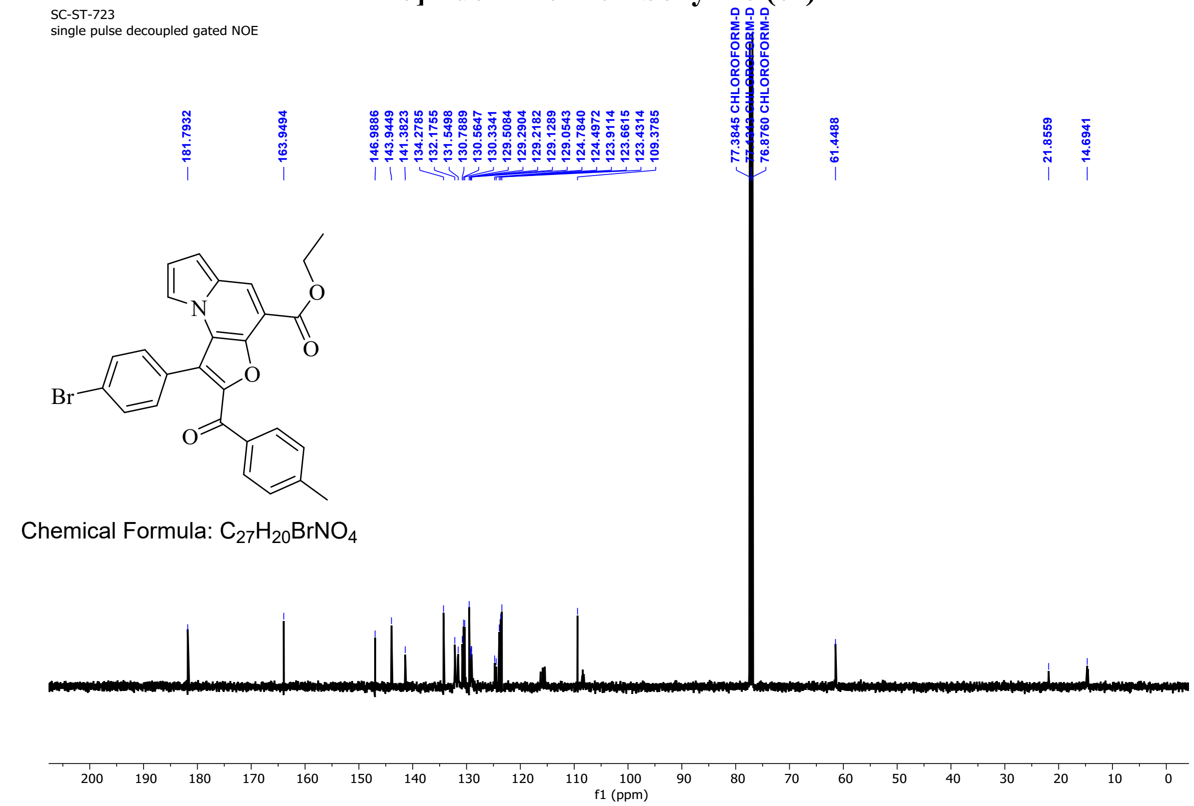

Figure S121. ^{13}C **NMR Spectrum of Ethyl 1-(4-bromophenyl)-2-(4-methylbenzoyl)furo[3,2-*e*]indolizine-4-carboxylate (6z)**

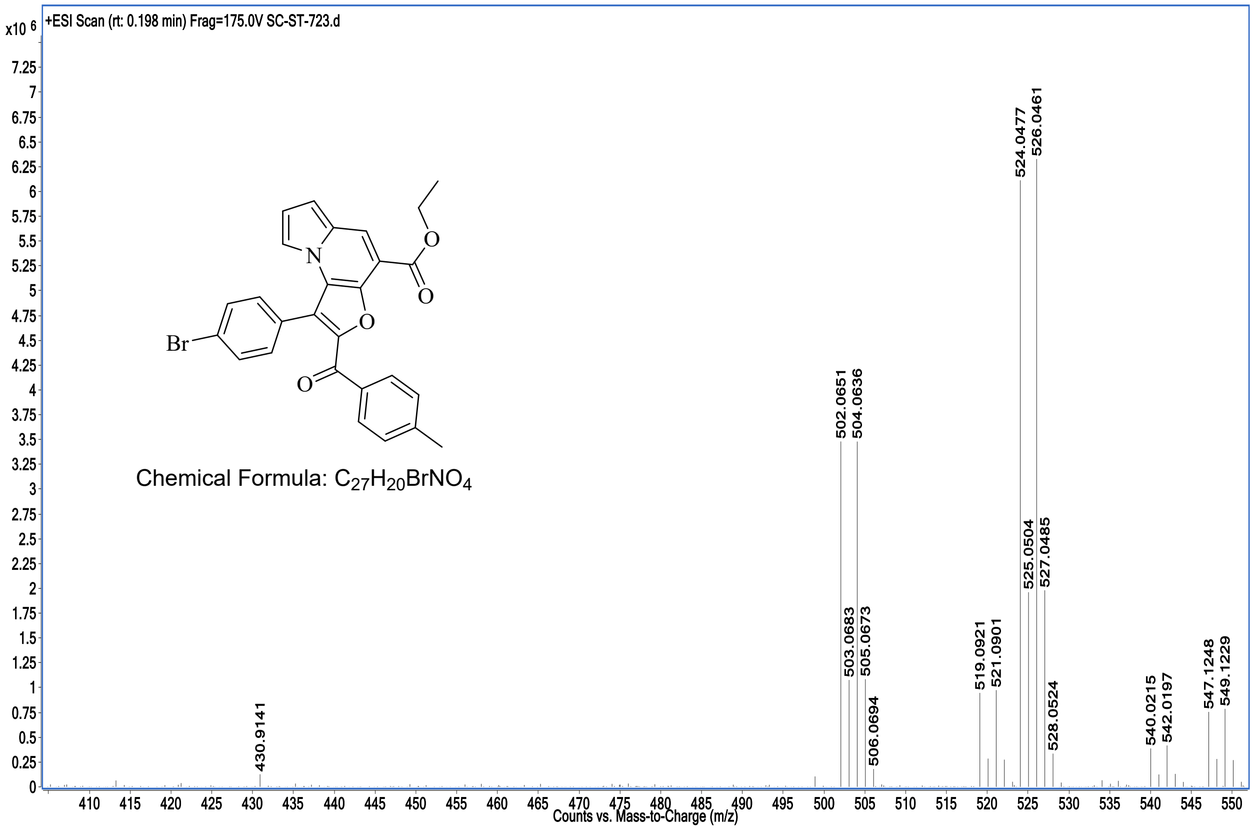

Figure S122. HRMS Spectrum of Ethyl 1-(4-bromophenyl)-2-(4-methylbenzoyl)furo[3,2-*e*]indolizine-4-carboxylate (6z)

49. Ethyl 1-(4-bromophenyl)-2-(4-cyanobenzoyl)furo[3,2-*e*]indolizine-4-carboxylate (6aa)

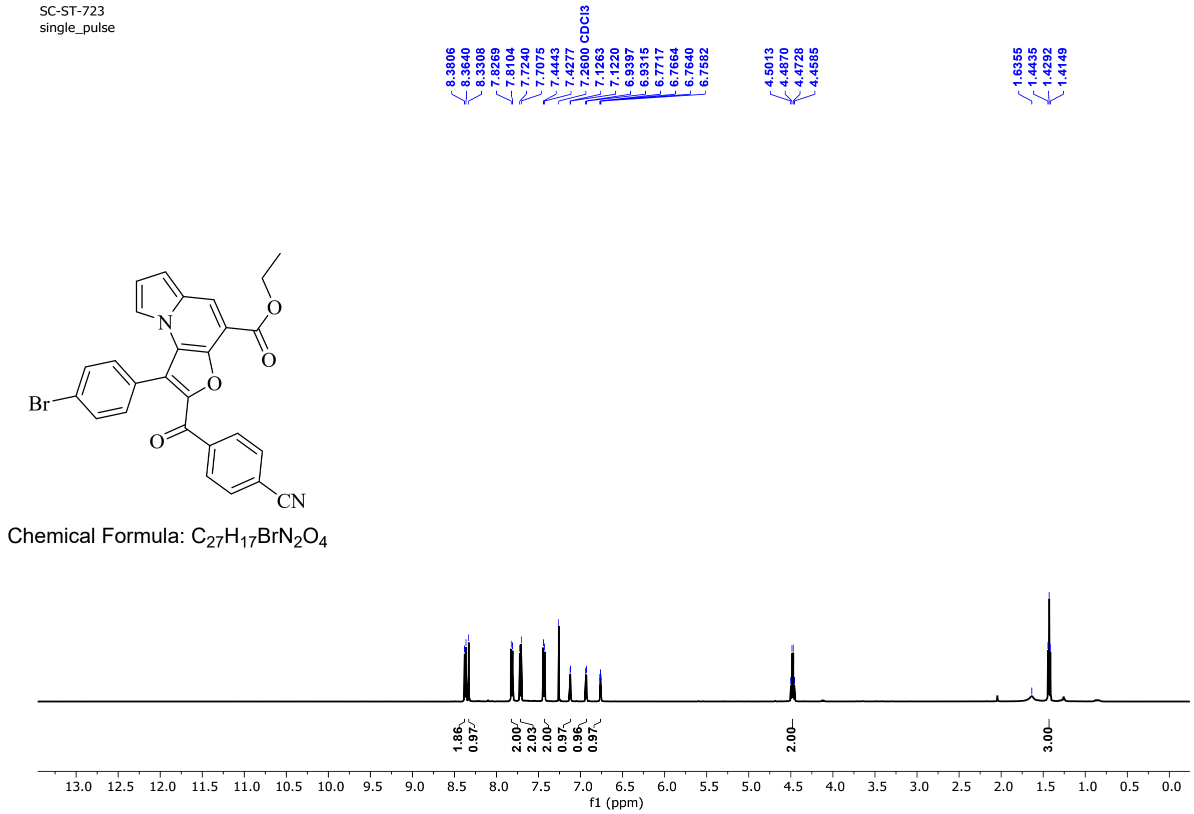

Figure S123. ^{1}H NMR Spectrum of Ethyl 1-(4-bromophenyl)-2-(4-cyanobenzoyl)furo[3,2-*e*]indolizine-4-carboxylate (6aa)

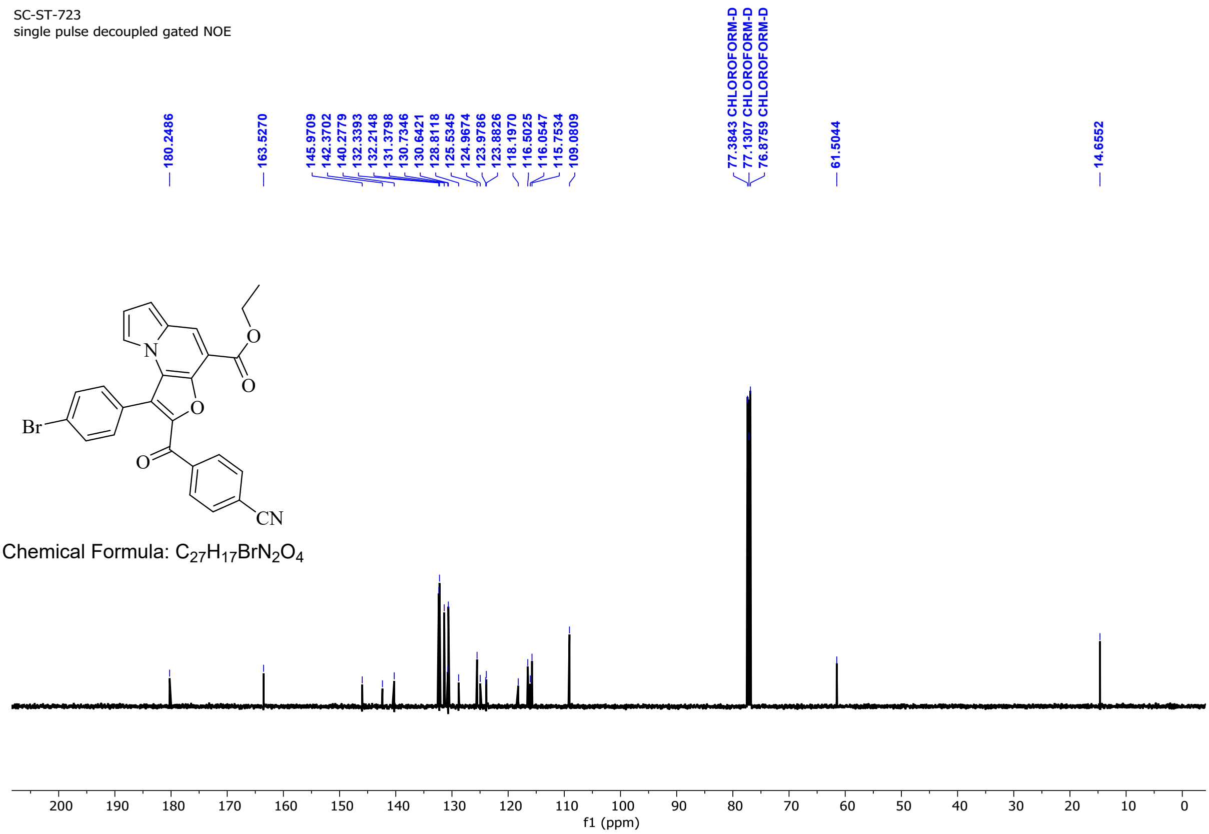

Figure S124. ^{13}C NMR Spectrum of Ethyl 1-(4-bromophenyl)-2-(4-cyanobenzoyl)furo[3,2-*e*]indolizine-4-carboxylate (6aa)

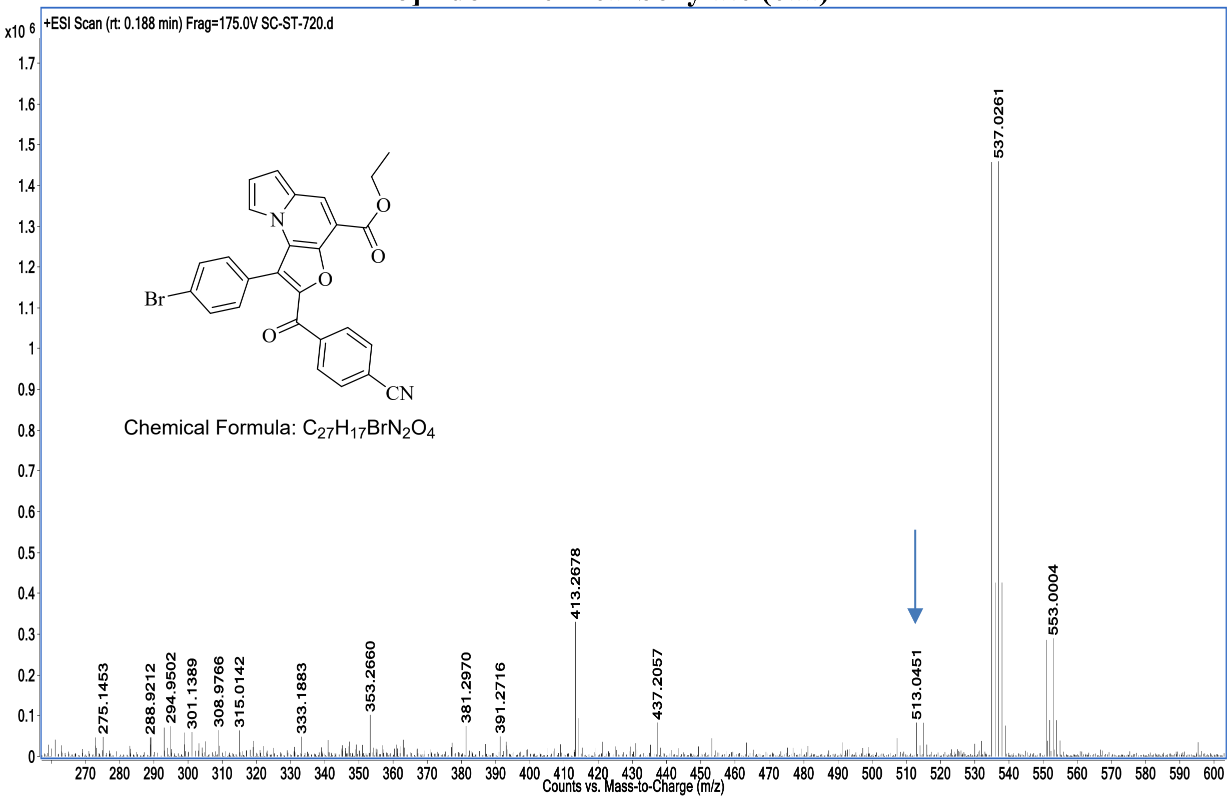

Figure S125. HRMS Spectrum of Ethyl 1-(4-bromophenyl)-2-(4-cyanobenzoyl)furo[3,2-*e*]indolizine-4-carboxylate (6aa)

50. Ethyl 1-(4-bromophenyl)-2-(2-nitrobenzoyl)furo[3,2-*e*]indolizine-4-carboxylate (6ab)

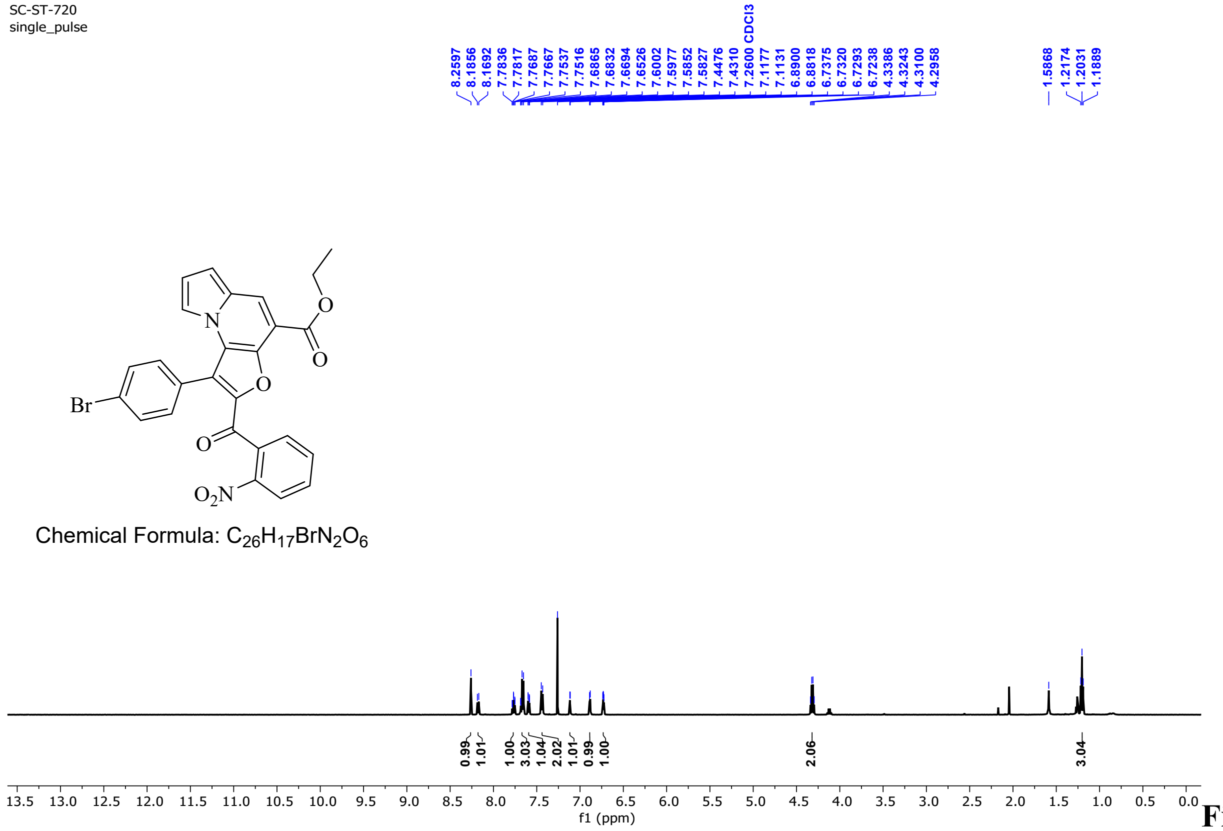

Figure S126. ^{1}H NMR Spectrum of Ethyl 1-(4-bromophenyl)-2-(2-nitrobenzoyl)furo[3,2-*e*]indolizine-4-carboxylate (6ab)

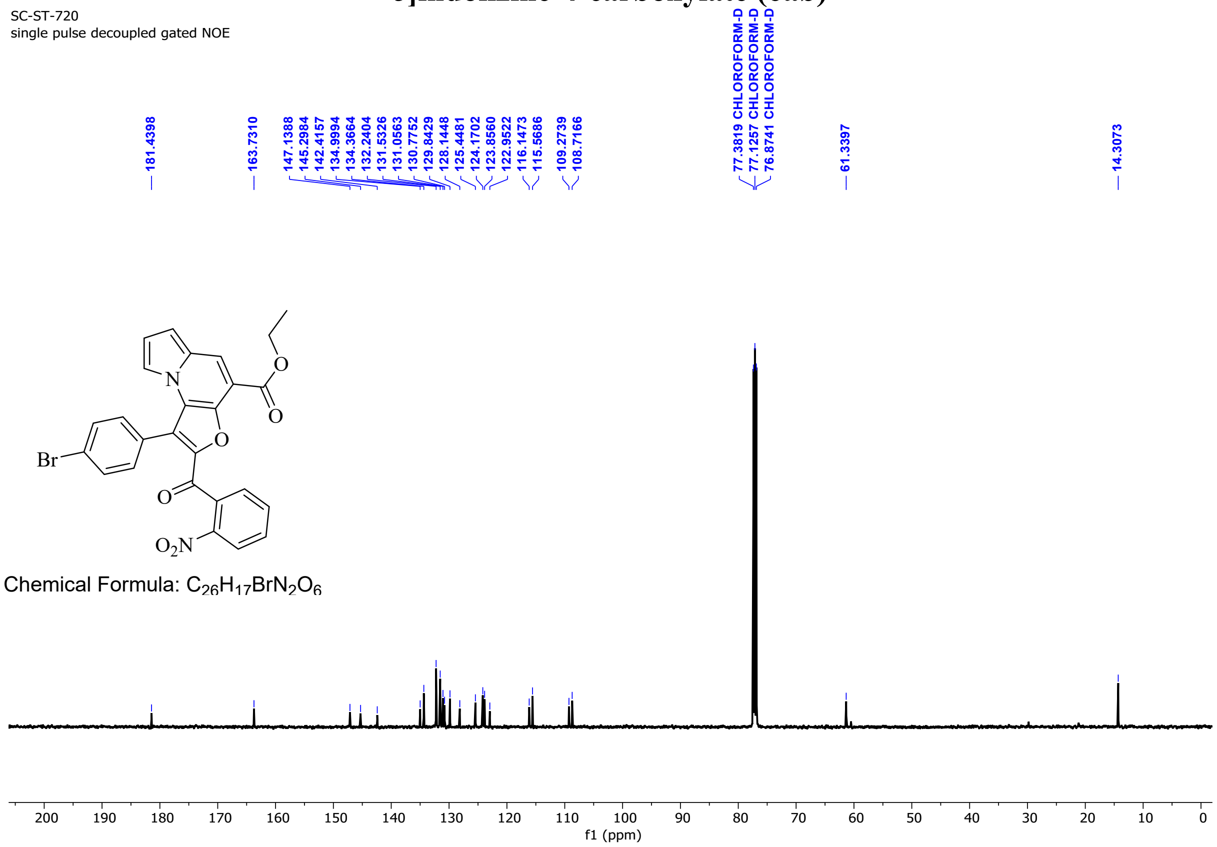

Figure S127. ^{13}C NMR Spectrum of Ethyl 1-(4-bromophenyl)-2-(2-nitrobenzoyl)furo[3,2-*e*]indolizine-4-carboxylate (6ab)

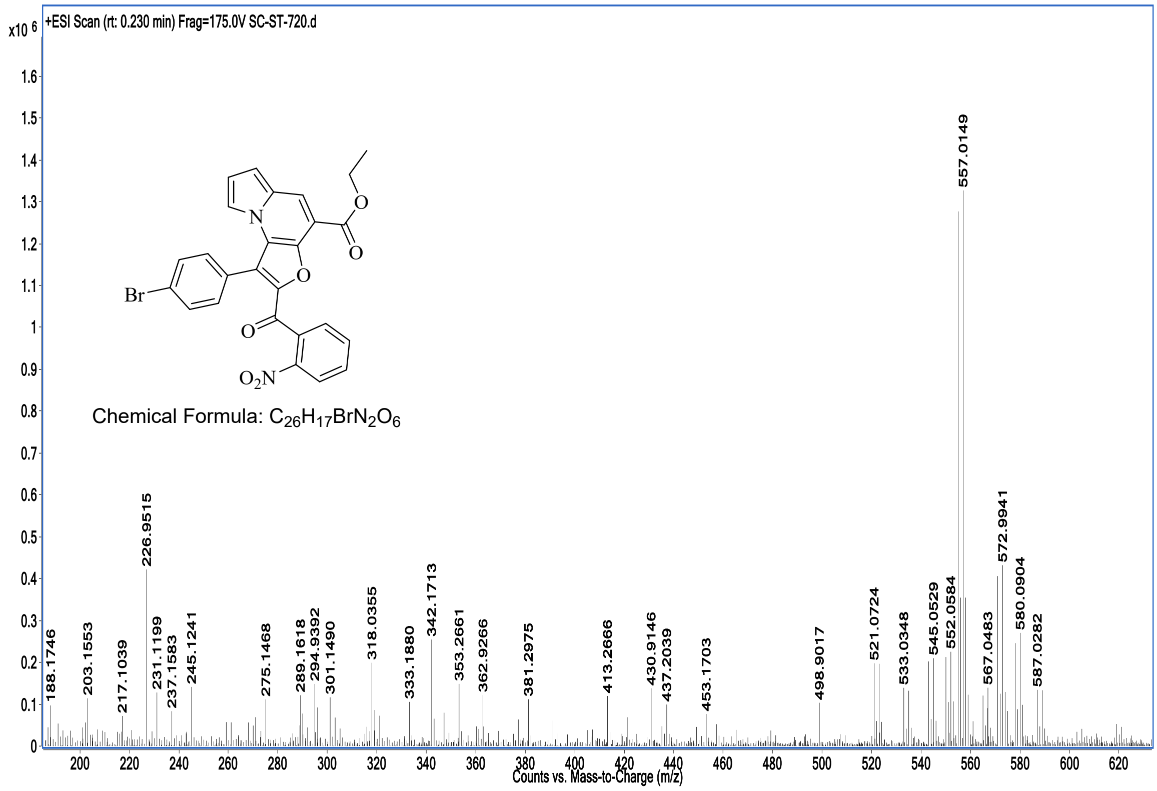

Figure S128. HRMS Spectrum of Ethyl 1-(4-bromophenyl)-2-(2-nitrobenzoyl)furo[3,2-*e*]indolizine-4-carboxylate (6ab)

51. Ethyl 1-(4-bromophenyl)-2-(4-(trifluoromethyl)benzoyl)furo[3,2-*e*]indolizine-4-carboxylate (6ac)

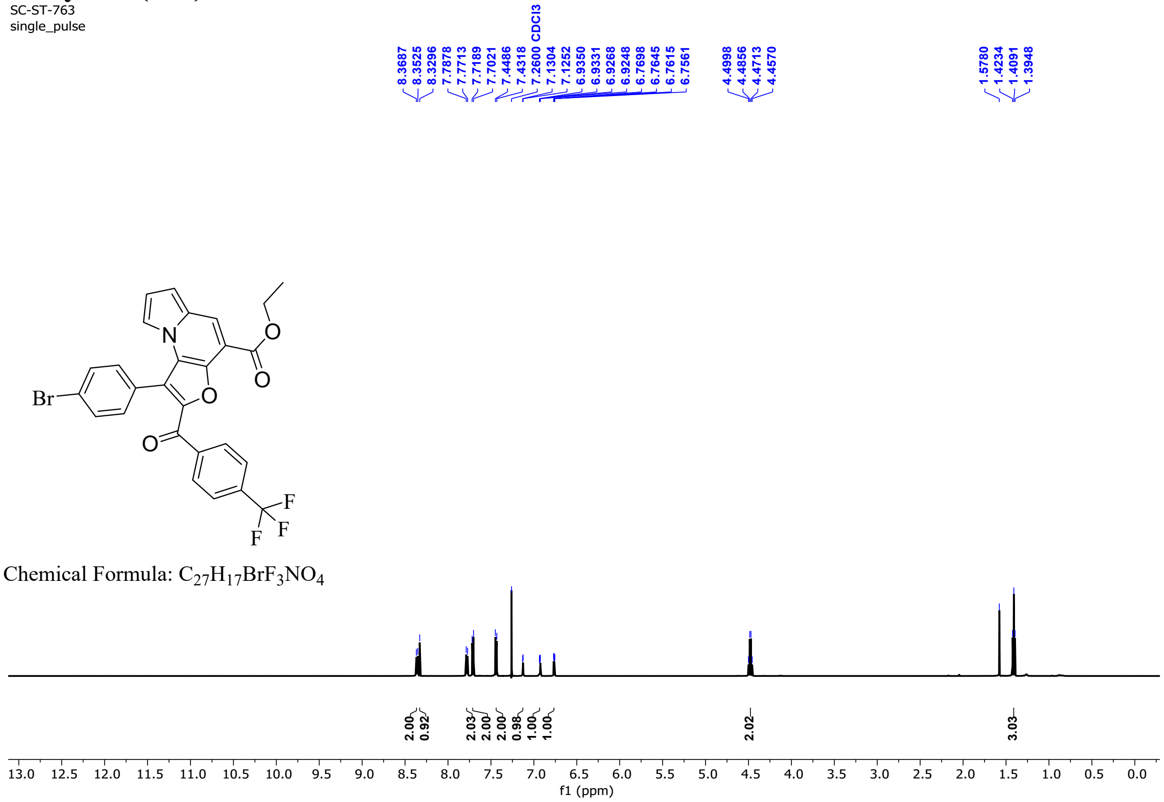

Figure S129. ^{1}H NMR Spectrum of Ethyl 1-(4-bromophenyl)-2-(4-(trifluoromethyl)benzoyl)furo[3,2-*e*]indolizine-4-carboxylate (6ac)

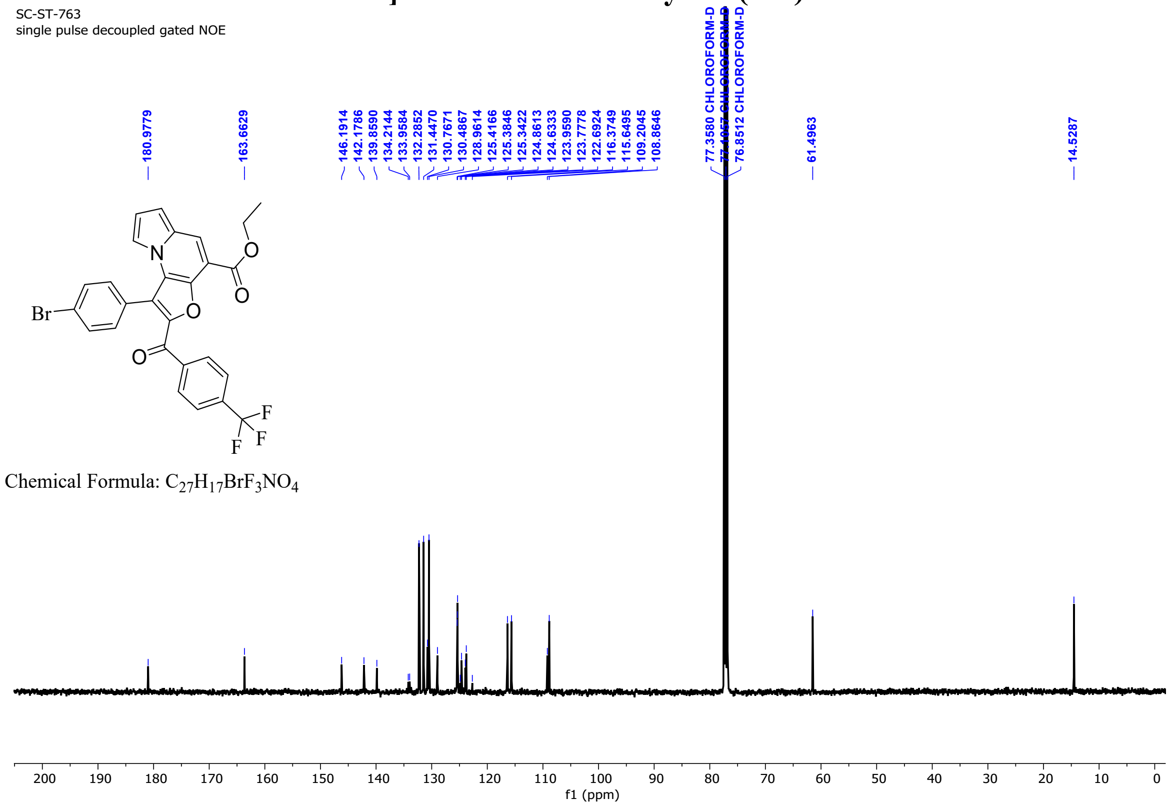

Figure S130. ^{13}C NMR Spectrum of Ethyl 1-(4-bromophenyl)-2-(4-(trifluoromethyl)benzoyl) furo[3,2-*e*]indolizine-4-carboxylate (6ac)

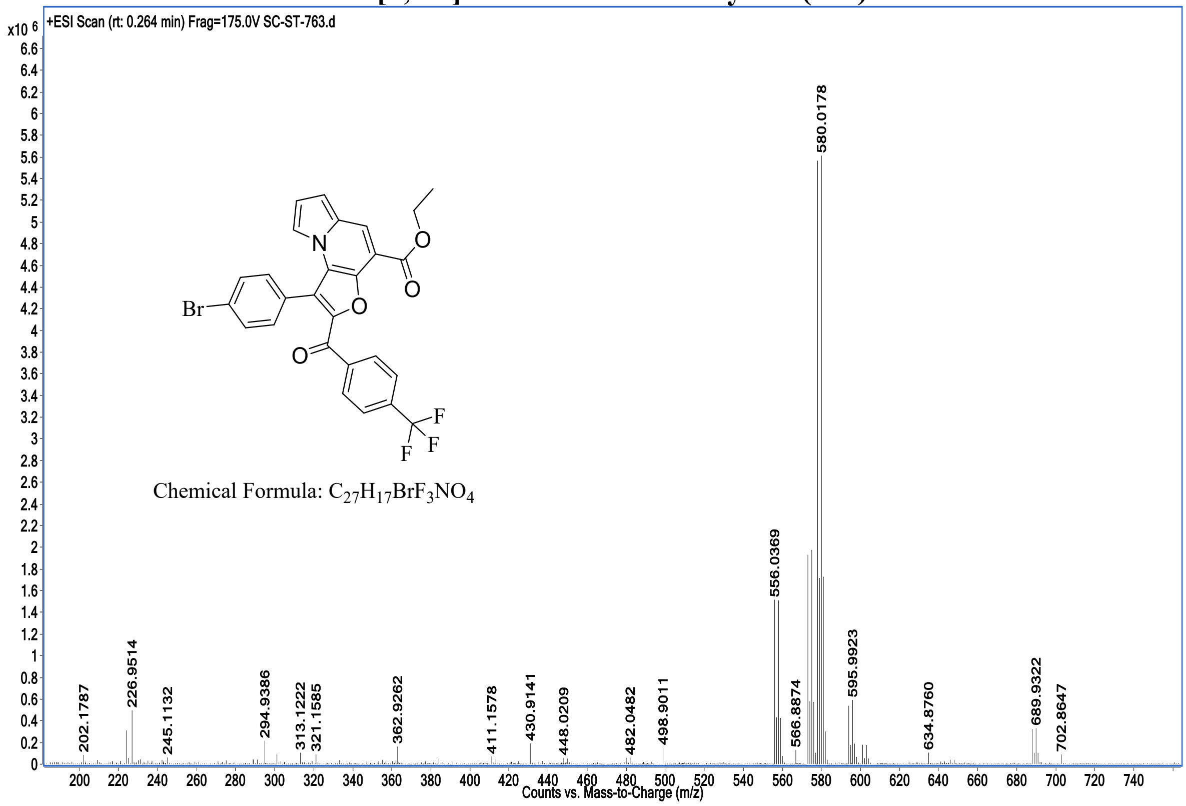

Figure S131. HRMS Spectrum of Ethyl 1-(4-bromophenyl)-2-(4-(trifluoromethyl)benzoyl) furo[3,2-*e*]indolizine-4-carboxylate (6ac)

52. Ethyl 2-(4-fluorobenzoyl)-1-(4-methoxyphenyl)-6,8-dimethylfuro[3,2-*e*]indolizine-4-carboxylate (8a)

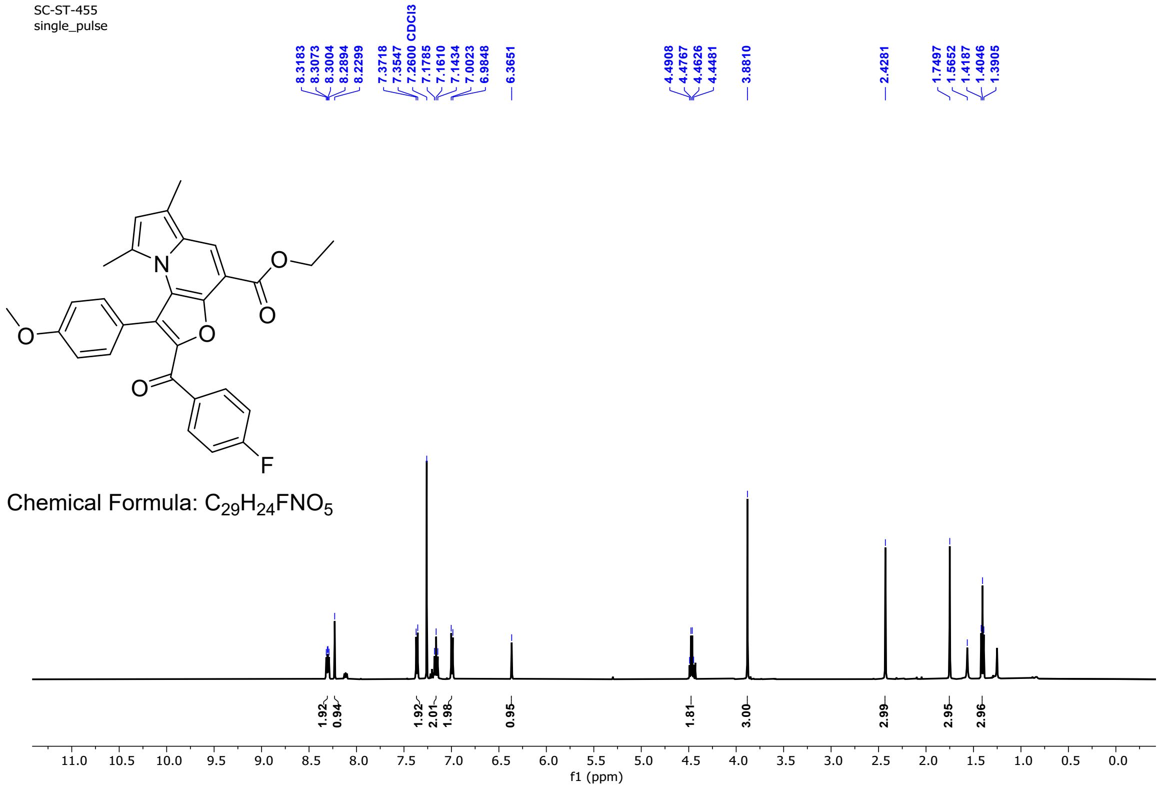

Figure S132. ^{1}H NMR Spectrum of Ethyl 2-(4-fluorobenzoyl)-1-(4-methoxyphenyl)-6,8-dimethylfuro[3,2-*e*]indolizine-4-carboxylate (8a)

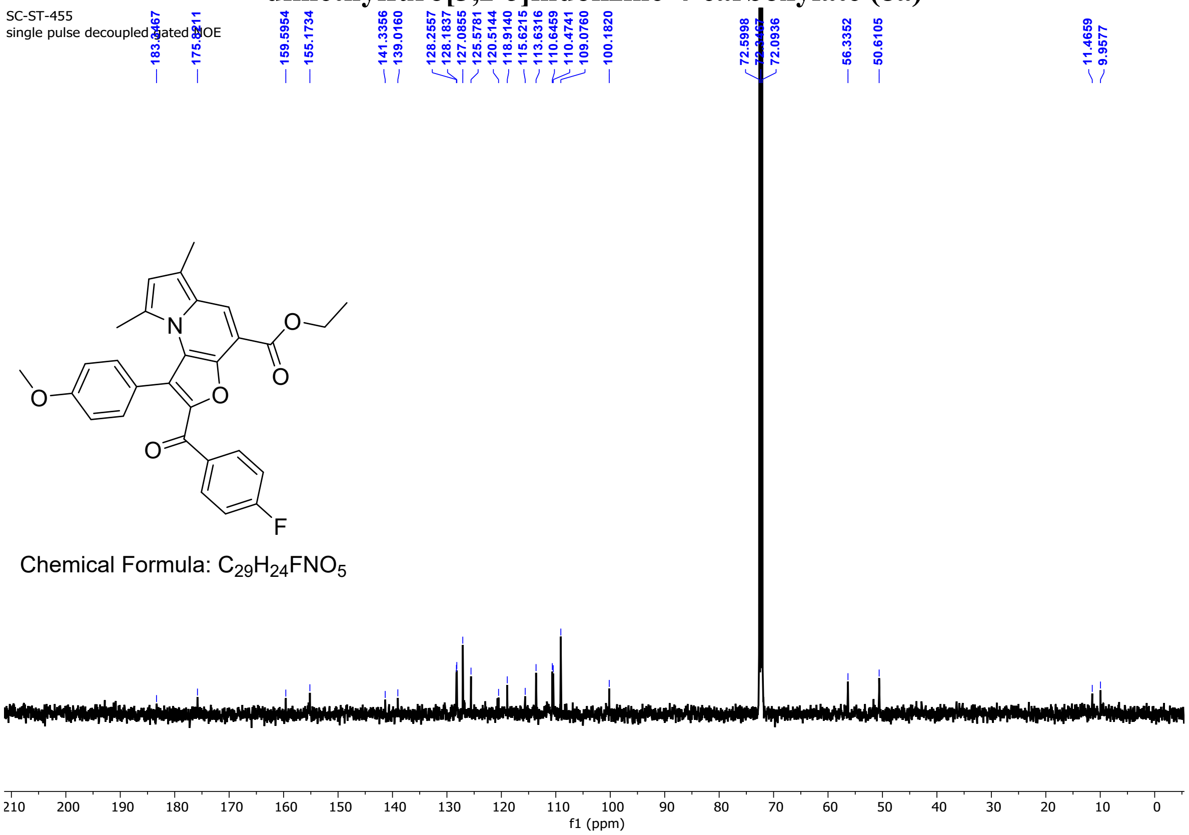

Figure S133. ^{13}C NMR Spectrum of Ethyl 2-(4-fluorobenzoyl)-1-(4-methoxyphenyl)-6,8-dimethylfuro[3,2-*e*]indolizine-4-carboxylate (8a)

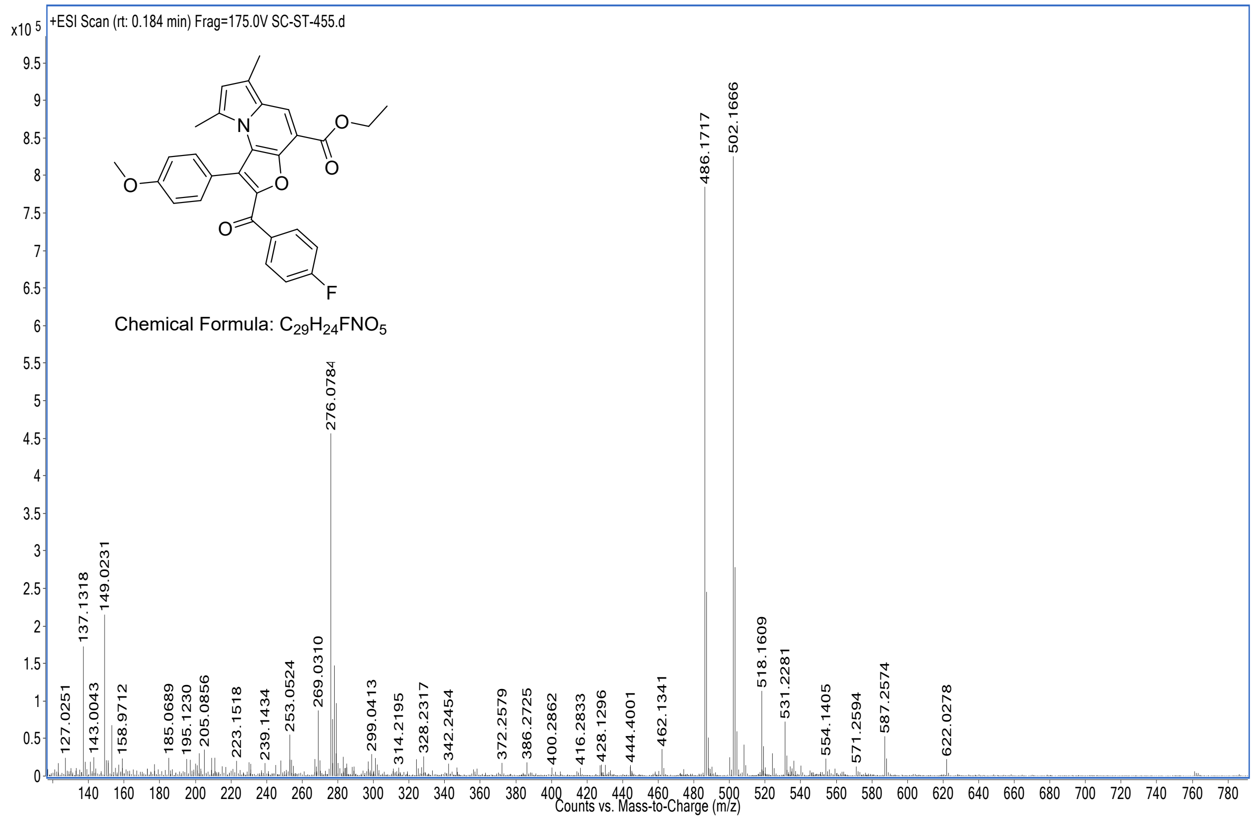

Figure S134. ^{13}C NMR Spectrum of Ethyl 2-(4-fluorobenzoyl)-1-(4-methoxyphenyl)-6,8-dimethylfuro[3,2-*e*]indolizine-4-carboxylate (8a)

53. Ethyl 2-(4-bromobenzoyl)-6,8-dimethyl-1-(p-tolyl)furo[3,2-*e*]indolizine-4-carboxylate (8b)

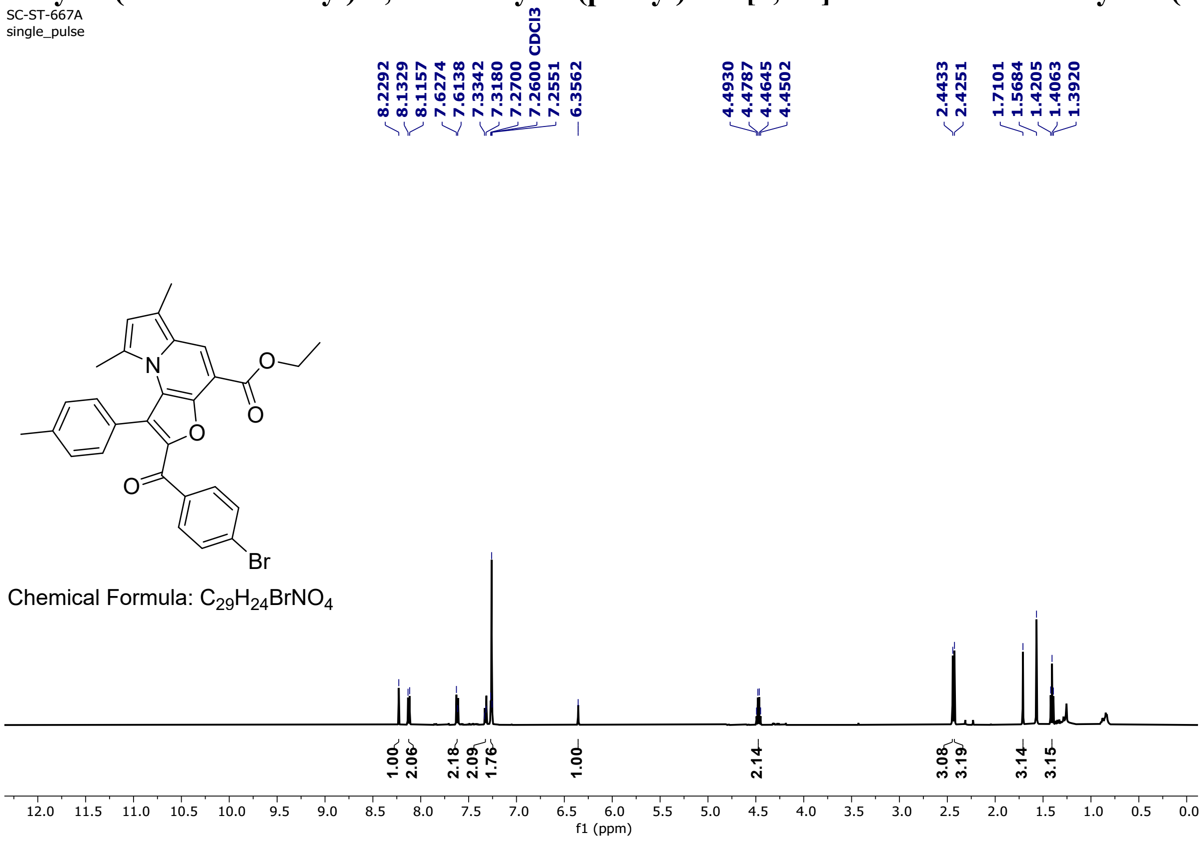

Figure S135. ^{1}H NMR Spectrum of Ethyl 2-(4-bromobenzoyl)-6,8-dimethyl-1-(p-tolyl)furo[3,2-*e*]indolizine-4-carboxylate (8b)

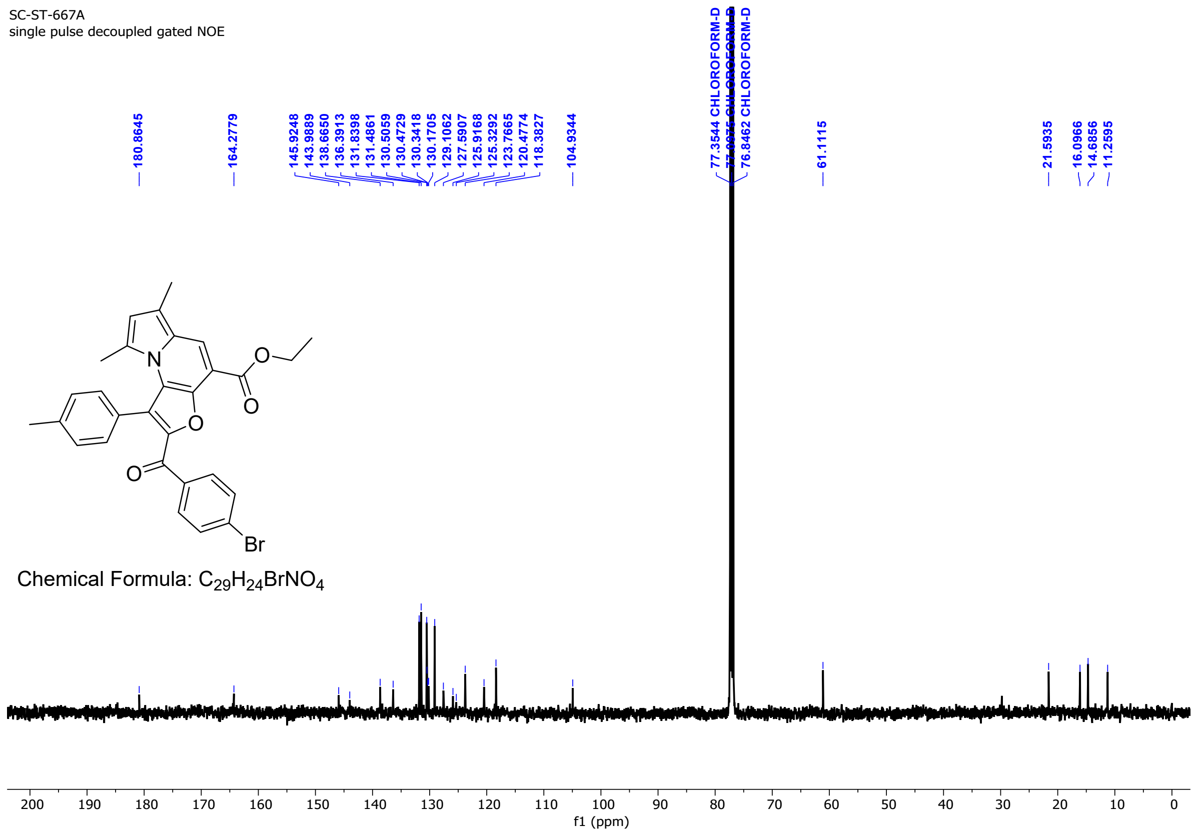

Figure S136. ^{13}C NMR Spectrum of Ethyl 2-(4-bromobenzoyl)-6,8-dimethyl-1-(p-tolyl)furo[3,2-*e*]indolizine-4-carboxylate (8b)

54. Ethyl 2-cyano-1-phenylfuro[3,2-*e*]indolizine-4-carboxylate (10a)

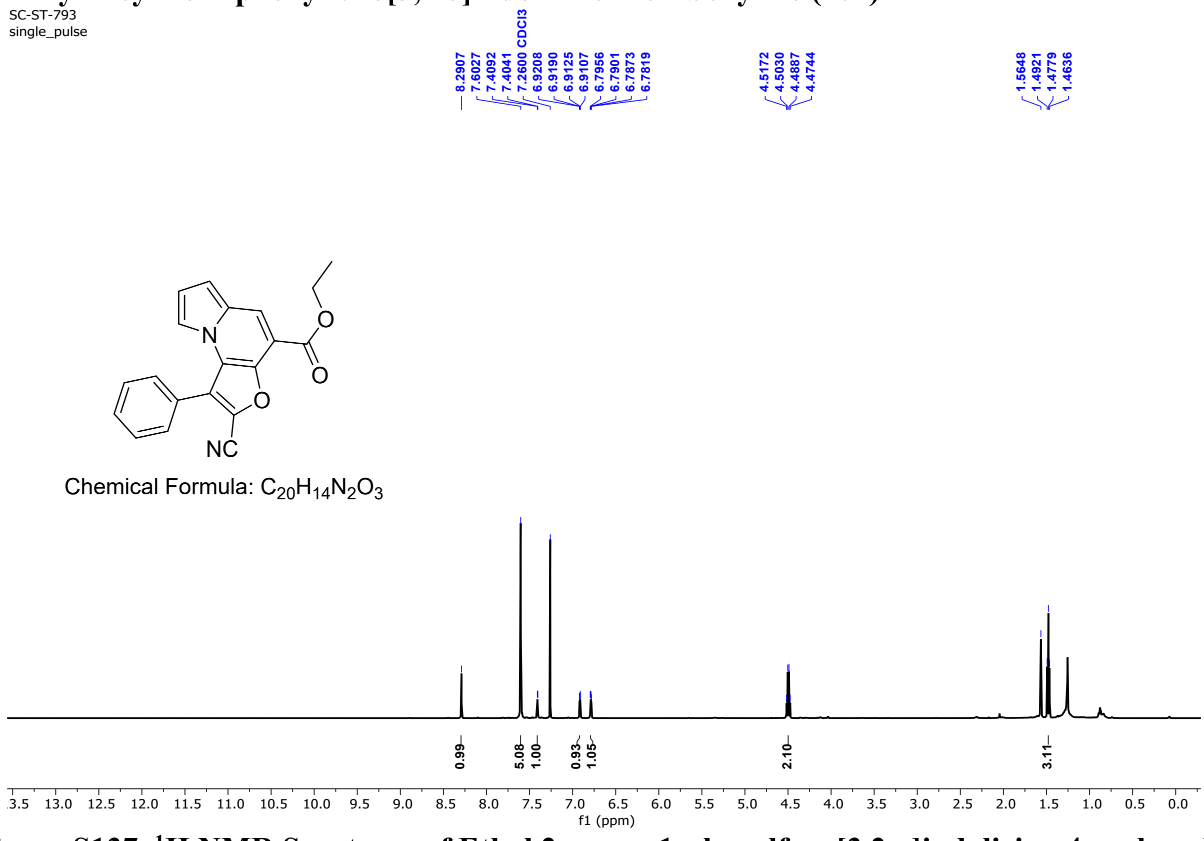

Figure S137. ^{1}H NMR Spectrum of Ethyl 2-cyano-1-phenylfuro[3,2-*e*]indolizine-4-carboxylate (10a)

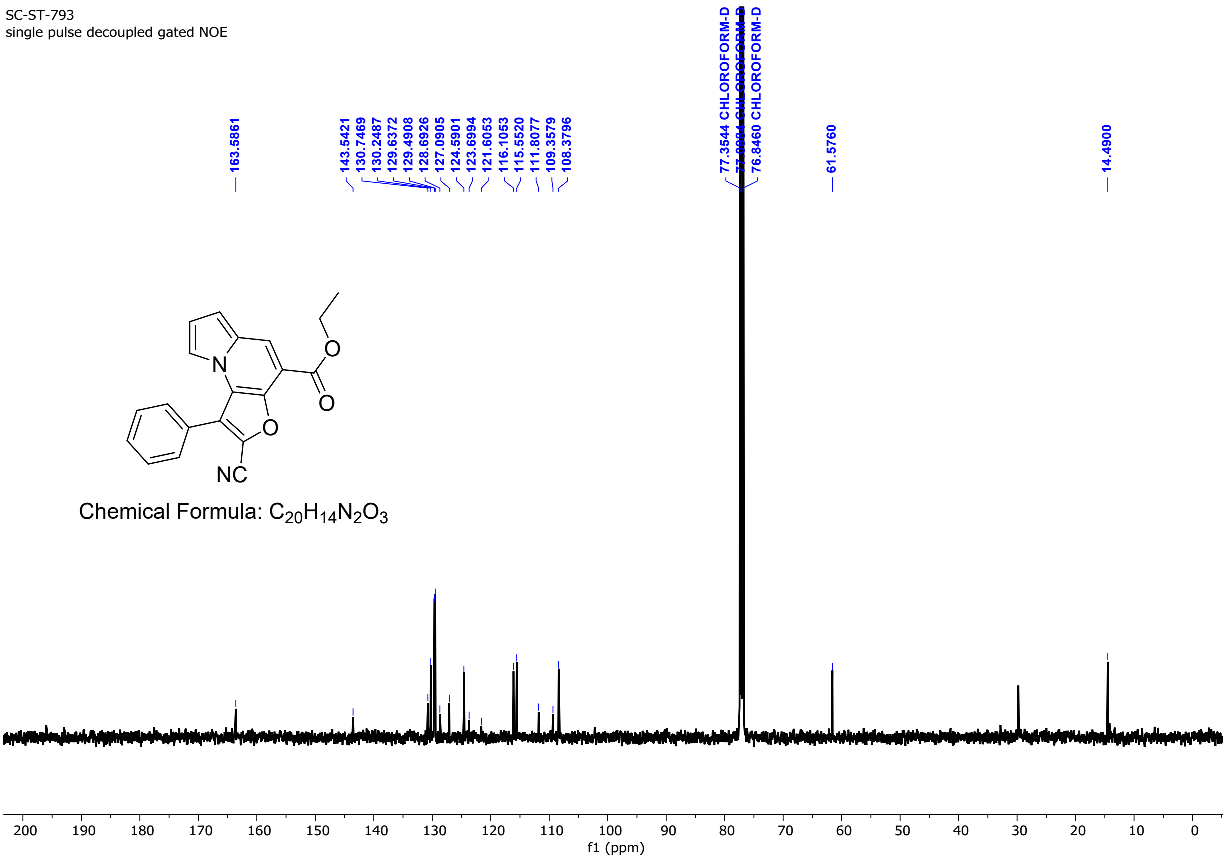

Figure S138. ^{13}C NMR Spectrum of Ethyl 2-cyano-1-phenylfuro[3,2-*e*]indolizine-4-carboxylate (10a)

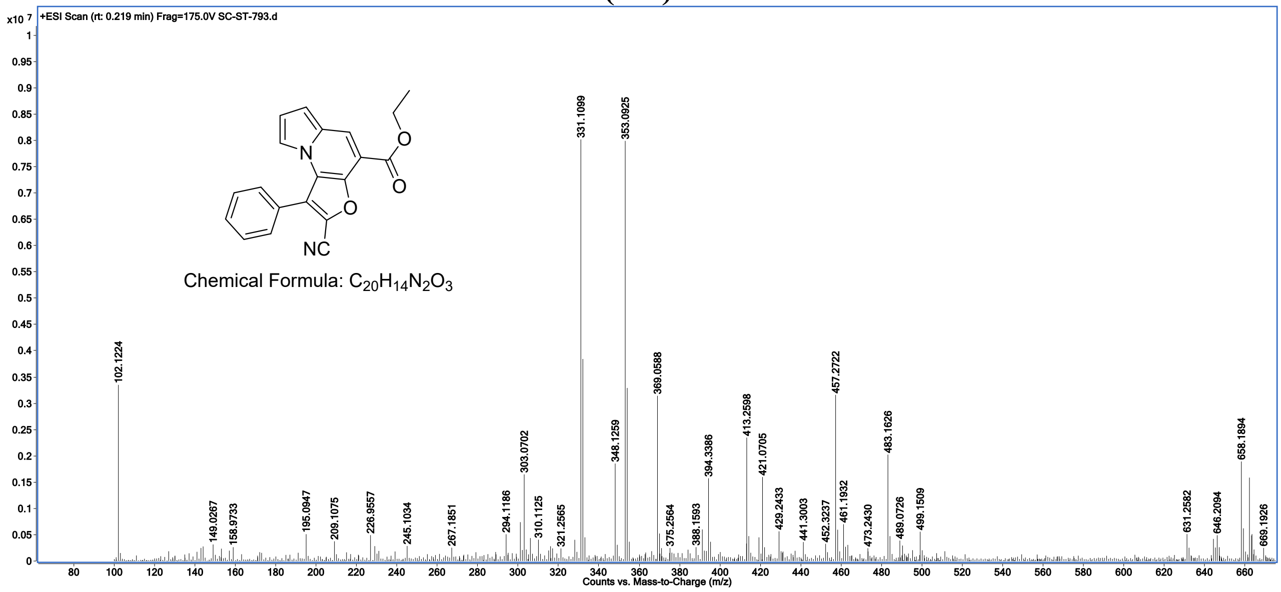

Figure S139. HRMS Spectrum of Ethyl 2-cyano-1-phenylfuro[3,2-*e*]indolizine-4-carboxylate (10a)
